# Programmable Monodisperse Glyco-Multivalency Using Self-Assembled Coordination Cages as Scaffolds

**DOI:** 10.1021/acsami.3c08666

**Published:** 2023-07-24

**Authors:** Callum Pritchard, Melissa Ligorio, Garrett D. Jackson, Matthew I. Gibson, Michael D. Ward

**Affiliations:** † Department of Chemistry, University of Warwick, Coventry CV47AL, U.K.; ‡ Division of Biomedical Sciences, Warwick Medical School, University of Warwick, Coventry CV47AL, U.K.

**Keywords:** glycans, lectins, self-assembly, coordination cages, multivalency

## Abstract

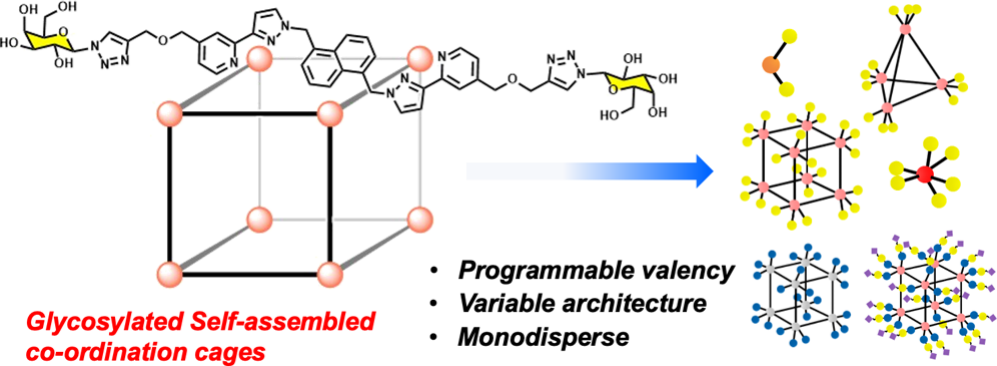

The multivalent presentation of glycans leads to enhanced binding avidity to lectins due to the cluster glycoside effect. Most materials used as scaffolds for multivalent glycan arrays, such as polymers or nanoparticles, have intrinsic dispersity: meaning that in any sample, a range of valencies are presented and it is not possible to determine which fraction(s) are responsible for binding. The intrinsic dispersity of many multivalent glycan scaffolds also limits their reproducibility and predictability. Here we make use of the structurally programmable nature of self-assembled metal coordination cages, with polyhedral metal-ion cores supporting ligand arrays of predictable sizes, to assemble a 16-membered library of perfectly monodisperse glycoclusters displaying valencies from 2 to 24 through a careful choice of ligand/metal combinations. Mono- and trisaccharides are introduced into these clusters, showing that the synthetic route is tolerant of biologically relevant glycans, including sialic acids. The cluster series demonstrates increased binding to a range of lectins as the number of glycans increases. This strategy offers an alternative to current glycomaterials for control of the valency of three-dimensional (3-D) glycan arrays, and may find application across sensing, imaging, and basic biology.

## Introduction

Protein-carbohydrate interactions are central to many recognition and signalling processes in nature, such as cell growth, immune function, and fertilization.^1−3^ All mammalian cells are coated with a complex glycan layer (the glycocalyx), which enables identification of self and signals the health of the cell, but is also exploited by pathogens in order to gain entry into the host. The protein “readers”^4^ of glycans are termed *lectins*: these engage in relatively weak interactions with glycans, with individual association constants of typically 10^3^ M^–1^. To overcome the intrinsically weak binding of an individual glycan with a lectin, multiple copies of each glycan are present in the glycocalyx, leading to a nonlinear increase in affinity termed the *cluster glycoside effect* arising from the presence of multiple interaction sites.^5−7^

There is a vast range of glycan-coated synthetic polymers, dendrimers, and nanoparticles that mimic this multivalent presentation to provide strong interactions with lectins: these have been used in a range of biomedical applications,^8−12^ and the strength of the interaction depends crucially on the arrangement of glycans on the exterior surface that is presented to the lectin.^13,14^ Such artificial, multivalent, glycan-functionalized materials are, however, generally not homogeneous but have intrinsic shape and size dispersity, meaning that the number and arrangement of glycans on each individual polymer strand or nanoparticle surface will be different. This in turn means that understanding and dissecting the specific interactions involved in binding of multivalent systems is difficult: it is not known which component in a distribution is the most avid binder to the substrate and which components are outliers. This uncertainty also limits reproducibility as small fractions of longer or shorter polymers, or smaller or larger nanoparticles, could be dominating the observed macroscopic interactions. Overall, this prevents the rational design of new multivalent glyco-mimetics, especially those whose interactions with proteins are to be used as the basis of biosensing or diagnostics.^15−17^ Considering this, the ability to pre-program multivalent glycan arrays that are monodisperse with a predictable three-dimensional (3-D) arrangement of glycans, and that are structurally identical each time they are made, is a challenging but important target.

A potentially useful platform for assembly of multi-glycan arrays, which has been exploited relatively rarely, is provided by supramolecular metal/ligand arrays in the form of coordination cages.^18−23^ These can be very large in conventional molecular terms (many tens of Å in diameter) with the largest examples (*e.g.*, Fujita’s pseudo-spherical Pd_30_L_60_ cage, with a diameter of 82 Å) comparable to small nanoparticles in size,^24^ and are based on the self-assembly of multiple copies of metal ions and (often relatively simple) ligands in a way that generates a single and highly symmetric product. Such cages provide clear benefits for use as scaffolds on which to base multi-glycan arrays. Firstly, they are monodisperse, with careful design of ligands and choice of metal ions leading to formation of a single product whose pendant glycan array is, accordingly, structurally highly predictable. Secondly, they contain multiple copies of (usually) just two units—metal ion and ligand—which self-assemble in a single, often trivially simple reaction: meaning that a large multi-glycan array only requires prior synthesis of a small glycan-substituted ligand, and the self-assembly process of multiple copies of this with metal ions in one step does the rest. Examples of self-assembled coordination cages (and related large metal complex assemblies) used in this way as a platform for self-assembled glycan arrays are rare, with notable examples provided by the groups of Fujita,^25^ Stauber,^26^ Stang,^27^ and Spokoyny^28^ (among others).

In this paper we report a systematic study into the use of metal complexes as scaffolds for formation of glycan arrays, and the resulting recognition processes of these glycoclusters with lectins, based on a family of coordination cages that we have studied extensively in recent years.^29,30^ Attachment of two glycan residues to each ligand, one at each of the pyridyl ring termini, is followed by assembly of tetrahedral M_4_L_6_ and cubic M_8_L_12_ cages generating an external array of twelve or twenty-four glycans, respectively, arranged in a predictable 3-D geometry defined by the underlying cage superstructure.

Firstly, to allow insights into the extent of the cluster glycoside effect in these systems—the increased binding strength with lectins associated with multivalency^5,6^—we have compared a series of glucose-substituted and galactose-substituted complexes for their binding to galactose-specific lectins (with the glucose analogues acting as controls). For this part of the work, in addition to the functionalized M_4_L_6_ and M_8_L_12_ cages, we have also used smaller mononuclear complexes bearing two or six pendant glycan units (from one or three bipyridyl-type ligands) for comparison with the 12- and 24-membered glycoclusters based on the coordination cages. Overall, this allows a comparison of the binding properties of a series of glycoclusters with 2, 6, 12, and 24 pendant glycan (glucose or galactose) units. Secondly, we have used the sialic acid derivatives 3′- and 6′-sialyllactose to prepare coordination-cage-based 12- and 24-component glycoclusters, given the particular importance of sialic acids in pathological processes such as the zoonosis (transfer between species) of a range of viruses, such as the transfer of influenza from avians to humans.^31^ This is the first such study using supramolecular methods to assemble arrays of sialic acid derivatives for lectin binding studies.

## Experimental Section

### Materials

All reagents and solvents used within the synthesis and purification were purchased from commercial sources (Sigma-Aldrich, Fischer-Scientific, Acros-Organics or Fluorochem Ltd.) and used without prior purification unless otherwise stated. Dry solvents (EtOH, tetrahydrofuran (THF), and MeOH) were transferred to Schlenk flasks and kept over predried 4 Å molecular sieves prior to use. 3′-Sialyllactose sodium salt and 6′-sialyllactose sodium salt were purchased from Biosynth. SBA, Jacalin, WGA, SNA, and EBL were purchased from Vector Laboratories. d-galactose and sheep blood in Alsever’s were purchased from Merck. Ultrahigh-quality water with a resistance of 18.2 MΩ·cm (at 25 °C) was obtained from a Millipore Milli-Q gradient machine fitted with a 0.22 μM filter.

### Techniques

Air-sensitive reactions were performed under nitrogen or argon atmospheres using typical Schlenk techniques. ^1^H NMR and ^13^C{^1^H} NMR spectra were recorded at 300, 400, or 500 MHz (^1^H) and 75 or 125 MHz (^13^C), respectively, using Bruker Avance (300 MHz), Bruker Avance III HD (400 MHz), or Bruker Avance III HD (500 MHz) spectrometers. ^19^F{^1^H} NMR were also recorded using a Bruker Avance III HD (400 MHz) spectrometer. All NMR spectra were measured at 25 °C in the indicated deuterated solvents unless stated otherwise. Proton and carbon chemical shifts (δ) are reported in ppm and coupling constants (*J*) are reported in Hertz (Hz). The resonance multiplicities in the ^1^H NMR spectra are described as “s” (singlet), “d” (doublet), “t” (triplet), “q” (quartet), “dd” (doublet of doublets), “ddd” (doublet of doublet of doublets), and “m” (multiplet), and broad resonances are indicated by “br”.

Two-dimensional (2D) homonuclear correlation ^1^H–^1^H COSY and 2D heteronuclear correlation ^1^H–^13^C HETCOR experiments (HMQC, HMBC) were used to confirm NMR peak assignments. Infrared spectra were recorded using a Bruker α IR-PLATINUM-ATR spectrophotometer with solid samples. Accurate mass measurements (ESI-HRMS) were performed using a Bruker maXis plus LC/ESI/MS instrument in positive-ion mode. Either protonated molecular ions [M + nH]^n+^ or sodium adducts [M + Na]^+^ were used for empirical formula confirmation. Elemental analyses for carbon, hydrogen, and nitrogen were performed using a FlashEA 1112 CH&N elemental analyzer from MEDAC Ltd. Chobham, Surrey GU24, 8JB, U.K.

Fluorescence measurements were collected using an Agilent Cary Eclipse fluorimeter and UV/Vis spectra were obtained using an Implen C40 Nanophotometer. Purifications by column chromatography were performed using either silica gel (Fluorochem Ltd, 60 Å, 40–63 μ) or Brockmann III aluminum oxide (Sigma-Aldrich). Size-exclusion chromatography was performed using Sephadex LH-20 or Sephadex G-50. The purities of the products were established by thin-layer chromatography (TLC) on either silica gel-coated aluminum plates (with F253 indicator; layer thickness, 200 μm; particle size, 2–25 μm; pore size 60 Å) or aluminum oxide-coated aluminum-backed plates (with F253 indicator, layer thickness, 1500 μm; particle size, pore size 150 Å).

Full details of the synthesis procedures are provided in the extensive Supporting Information, but an example cage synthesis is detailed here:

#### [Co_8_(L^15-Gal-Ac^)_12_(BF_4_)_16_] [Co_8_^Gal-Ac^] (**32**)

L^15-Gal-Ac^ (52 mg, 39 μmol, 1.5 equiv) was added to a 50 mL RBF solution and dissolved in CH_2_Cl_2_ (5 mL). A solution of Co(BF_4_)2·6H_2_O (9 mg, 26 μmol, 1.0 equiv) in MeOH (5 mL) was then added and a precipitate was immediately formed. The solution was heated to 40 °C and stirred for 24 h. The solution was cooled to RT, centrifuged, and the supernatant was washed sequentially with MeOH and CH_2_Cl_2_. Purification was then conducted on LH-20 Sephadex, with CH_3_CN as the eluent. Yield: 50 mg, 83%.

High-resolution ES-MS: *m*/*z* 3465.6056 ([Co_8_(L^15-Gal-Ac^)_12_(BF_4_)_11_]^5+^), 2873.8274 ([Co_8_(L^15-Gal-Ac^)_12_(BF_4_)_10_]^6+^), 2450.8634 ([Co_8_(L^15-Gal-Ac^)_12_(BF_4_)_9_]^7+^), 2133.6318 ([Co_8_(L^15-Gal-Ac^)_12_(BF_4_)_8_]^8+^), 1887.0001 ([Co_8_(L^15-Gal-Ac^)_12_(BF_4_)_7_]^9+^).

### Turbidimetry Experiments

Soybean agglutinin (SBA) was dissolved in HEPES buffer (10 mM HEPES, 0.15 M NaCl, 2 mM CaCl_2_, 0.2 mM MnCl_2_, pH 7.4).

Jacalin and wheat-germ agglutinin were dissolved in HEPES buffer (10 mM HEPES, 0.1 mM CaCl_2_, pH 8.5).

In a half-area flat-bottom 96-well plate, lectin (50 μL, 10 μM) and an aqueous solution of metal complex (5 μL, 500 μM) were quickly mixed and the absorbance was recorded at 420, 500, and 600 nm for 30 min every 60 s. A solution of free sugar (d-galactose, 3′-sialyllactose, or 6′-sialyllactose) (2 μL, 1 M) was added and the absorbance was recorded every 60 s for a further 30 min.

### Competition Experiments

Soybean agglutinin (SBA) was dissolved in HEPES buffer (10 mM HEPES, 0.15 M NaCl, 2 mM CaCl_2_, 0.2 mM MnCl_2_, pH 7.4).

Jacalin was dissolved in HEPES buffer (10 mM HEPES, 0.1 mM CaCl_2_, pH 8.5).

In a half-area flat-bottom 96-well plate, 20 μL of a serial dilution of d-galactose starting from 1 M, and 20 μL of lectin (40 μM) were incubated at room temperature for 1 h. An aqueous solution of glycan-appended metal complex (5 μL, 500 μM) was added to each well and the absorbance at 670, 700, and 750 nm was recorded every 60 s for 30 min.

## Results and Discussion

### Synthesis and Characterization: Mononuclear Complexes Bearing Glucose or Galactose Pendants

**Scheme 1:**
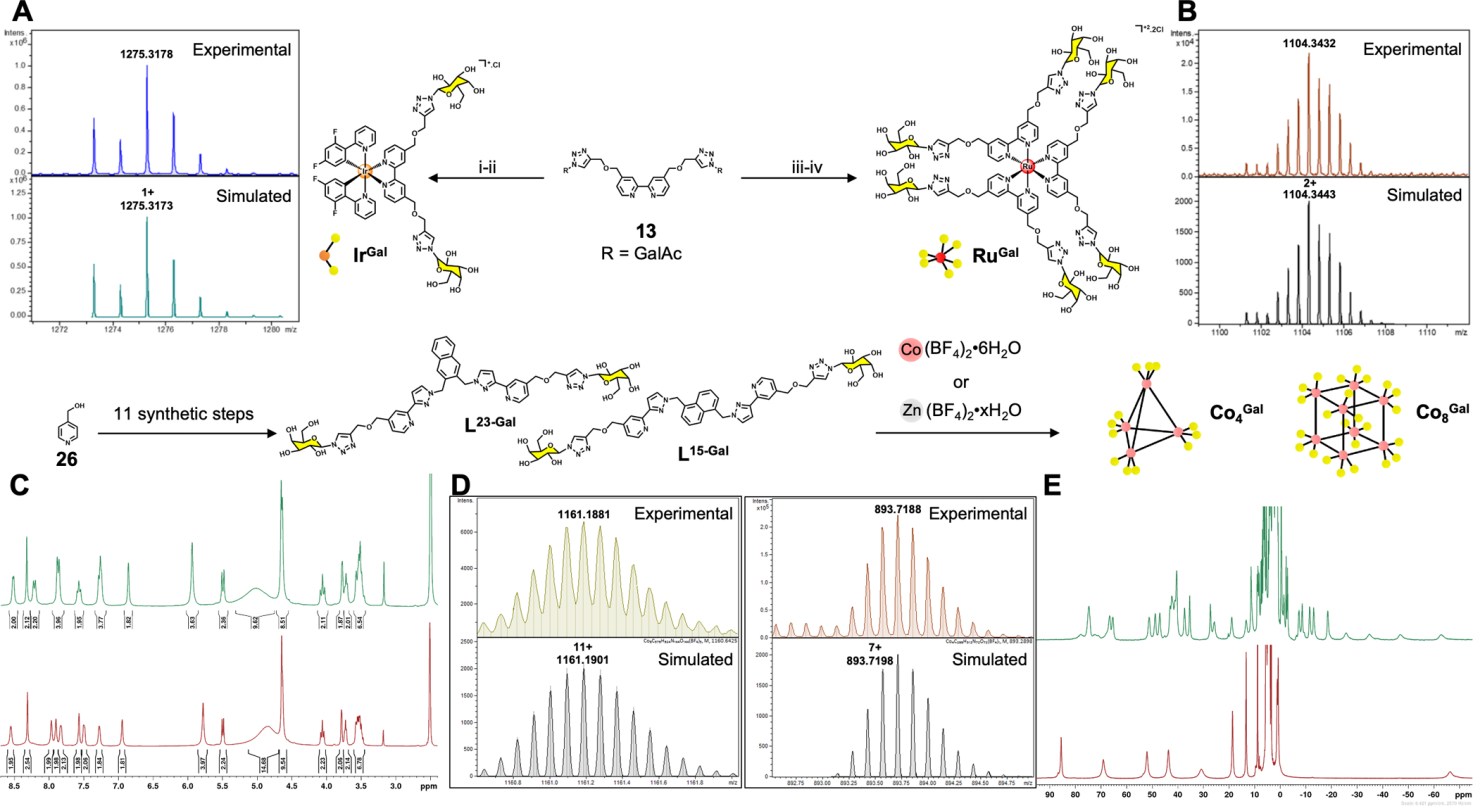
Synthesis Scheme and Analytical Data for the Preparation of Mononuclear Ir(III) (**Ir**^**Glu**^, **Ir**^**Gal**^) and Ru(II) (**Ru**^**Glu**^, **Ru**^**Gal**^) Complexes and Self-Assembled Tetrahedral Co_4_/Zn_4_ (**Co**_**4**_^**Glu**^/**Zn**_**4**_^**Gal**^) and Cubic Co_8_/Zn_8_ (**Co**_**8**_^**Glu**^/**Zn**_**8**_^**Gal**^) Cages Containing β-d-galactose and β-d-glucose Appendages: (i) [{Ir(F_2_ppy)_2_(μ-Cl)}_2_], MeOH/CH_2_Cl_2_ (1:1), r.t., 3 h; (ii) NaOMe (1 M in dry MeOH), r.t., 3 h; (iii) Ru(DMSO)_4_Cl_2_, EtOH, 78 °C, N_2_, 48 h, Darkness; (iv) MeOH/H_2_O/Et_3_N (4:2:1), 50 °C, N_2_, 18 h (A, B) HR-ESI-MS of **Ir**^**Gal**^ and **Ru**^**Gal**^; (C) ^1^H NMR Spectra of **L**^**23-Gal**^ and **L**^**15-Gal**^ (DMSO-*d*_6_, 25 °C, 400 MHz); (D) Selected Expansions of the HR-ESI-MS for **Co**_**4**_^**Gal**^ and **Co**_**8**_^**Gal**^; (E) ^1^H NMR Spectra of **Co**_**4**_^**Gal**^ and **Co**_**8**_^**Gal**^ (D_2_O, 90 °C, 400 MHz).

The set of complexes deployed here is shown in Scheme 1. It encompasses mononuclear complexes based on Ir(III) (complexes **Ir**^**Glu**^ and **Ir**^**Gal**^) and Ru(II) (complexes **Ru**^**Glu**^ and **Ru**^**Gal**^), to which are attached one or three (respectively) 2,2′-bipyridyl ligands, each with two pendant glycan units, and also encompasses the larger M_4_L_6_ and cubic M_8_L_12_ cages, in which all ligands again contain two pendant glycan units attached to their pyridyl termini. Thus, we have a set of complexes with glycan valencies of 2, 6, 12, 24 (for glucose and galactose), and 12 or 24 (for sialyllactose glycans).

The same general methodology was used in all cases for attachment of the glycan units to the pyridyl rings (Scheme 1). For the simple disubstituted bipy ligands **Bipy**^**Glu-Ac**^ (**9**) and **Bipy**^**Gal-Ac**^ (**13**) (where “Ac” denotes the presence of acetyl protecting groups on the glycan), we started with 4,4′-dimethyl-2,2′-bipyridine (**1**), which was converted to 4,4′-bis(hydroxymethyl)-2,2′-bipyridine (**4**) using a standard route.^32,33^ The hydroxyl groups were alkylated with propargyl bromide using NaH as the base in THF; we found that addition of 15-crown-5 to sequestrate the Na^+^ cations substantially improved the yield here. A Cu-AAC “Click” reaction^34^ with 1-azido-1-deoxy-β-d-glucopyranoside tetraacetate (**8**) or 1-azido-1-deoxy-β-d-galactopyranoside tetraacetate (**12**), using CuSO_4_/sodium l-ascorbate in a biphasic H_2_O/CH_2_Cl_2_ solvent system, resulted in two acetyl-protected glycan units being connected to the central bipy unit *via* 1,2,3-triazole spacers in reasonable yields (*ca.* 70%).

**Bipy**^**Glu-Ac**^ and **Bipy**^**Gal-Ac**^ were then reacted with [{Ir(F_2_ppy)_2_(μ–Cl)}_2_] [F_2_ppy = cyclometallated anion of 2-(2,4-difluorophenyl)pyridine] to afford mononuclear **Ir**^**Glu-Ac**^ (**16**) and **Ir**^**Gal-Ac**^ (**17**) as their chloride salts, which were purified by size-exclusion chromatography on Sephadex LH-20. Finally, deprotection under Zemplén conditions^35^—anhydrous MeOH and catalytic NaOMe—afforded the desired complexes **Ir**^**Glu**^ (**18**) and **Ir**^**Gal**^ (**19**). This deprotection step requires neutralization using the acidic ion-exchange resin Dowex 50W X8, and we found that leaving this step for too long resulted in the cation of the deprotected complexes **Ir**^**Glu**^ and **Ir**^**Gal**^ adhering to the resin, as shown by the loss of complex from solution and the appearance of green phosphorescence from the Dowex resin. This could be avoided by limiting the neutralization reaction time to 3 h.

To make 6-valent glycan complexes for comparison purposes, **Bipy**^**Glu-Ac**^ and **Bipy**^**Gal-Ac**^ were reacted with Ru(dmso)_4_Cl_2_ in EtOH at reflux (in darkness, under N_2_) in a 3:1 stoichiometric ratio to give the (protected) homoleptic Ru(II) *tris*-bipyridyl complexes. As with the Ir(III) complexes, purification was effected by chromatography on Sephadex LH-20 to give **Ru**^**Glu-Ac**^ (**20**) and **Ru**^**Gal-Ac**^ (**21**) in 75–80% yield. In these cases, glycan deprotection using MeOH/NaOMe clearly resulted in significant decomposition, so we found an alternative literature deprotection method involving Et_3_N in MeOH/H_2_O under N_2_ at 50 °C.^36^ Subsequent chromatographic purification required TOYOPEARL HW-40s, a hydroxylated methacrylate-based resin, as Sephadex LH-20 bound the deprotected hexa-glycan complexes **Ru**^**Glu**^ (**22**) and **Ru**^**Gal**^ (**23**) strongly. Elution on TOYOPEARL HW-40s with 0.01 M aqueous ammonium acetate afforded the pure bright orange products, which were separated from the excess ammonium acetate by precipitation with 2-propanol and then centrifugation.

All complexes were characterized by ^1^H NMR spectroscopy and high-resolution ES mass spectrometry (see SI).

### Synthesis and Characterization: Coordination-Cage-Based Glycoclusters Bearing Glucose or Galactose Pendants

The M_4_L_6_ tetrahedral cages^37−39^ (with 12 pendant glycans) and the M_8_L_12_ cubic cages^40,41^ (with 24 pendant glycans) are based on the bis(pyrazolyl-pyridine) ligands with 2,3-naphthyl^37−39^ and 1,5-naphthyl^40,41^ spacers, respectively. The nomenclature we use for these cages accordingly contains “23” or “15” to denote the ligand substitution pattern, “Glu”/“Gal” to denote the glycan type, and “Ac” (or not) to denote the presence of *O-*acetyl protecting groups.

To make the M_8_L_12_ cubic cages, we started with the known ligand L^15OH^ and alkylated it with 2 equiv of propargyl bromide.^42^ The subsequent Cu-AAC Click reactions with **8** and **12** then followed the methodology reported in the previous section to give the ligands **L**^**15-Glu-Ac**^ and **L**^**15-Gal-Ac**^, respectively, bearing two pendant acetyl-protected glycans connected to the terminal pyridyl rings *via* triazole spacers. These were deprotected using NaOMe/MeOH to give the ligands **L**^**15-Glu**^ (**30**) and **L**^**15-Gal**^ (**31**), which were converted to the M_8_L_12_ cages (M = Co, Zn) by reaction with M(BF_4_)_2_ in the appropriate 2M:3L ratio in MeOH at 50 °C for 24 h; after this time removal of solvent left a crude material which was purified by size-exclusion chromatography on Sephadex G-50, eluting with water. Note that removal of the *O-*acetyl protecting groups was performed on the ligands *before* the self-assembly step to prepare the cages, because (i) the deprotection conditions (NaOMe) were felt to be too harsh for use with relatively labile first-row transition metal complexes, and (ii) deprotection of a pre-assembled cube would require all 24 glycan units to fully deprotect, so deprotection and purification of the ligands (only 2 glycan units each) before the self-assembly step is more convergent. In all cases good yields were obtained for the desired products.

The M_4_L_6_ tetrahedral cages were prepared using the same methodology (Scheme 1). We needed first the (new) hydroxylated ligand skeleton **L**^**23OH**^ (**40**), which was prepared from 2 equiv of the same TIPS-protected hydroxylated pyrazolyl-pyridine unit^39^ that was used in the synthesis of **L**^**15OH**^. These were joined to a 2,3-naphthalene-diyl core *via* reaction with 2,3-bis(bromomethyl)naphthalene: removal of the TIPS groups liberated **L**^**23OH**^, which was then reacted further with (i) propargyl bromide and then (ii) **8** or **12***via* the Cu-AAC Click reaction, following the sequence described earlier, to give the ligands **L**^**23-Glu-Ac**^ (**42**) and **L**^**23-Gal-Ac**^ (**43**), respectively. Removal of the *O-*acetyl protecting groups, and then cage formation by reaction with the appropriate M(BF_4_)_2_ as described above for the cubic cages, afforded the desired M_4_L_6_ tetrahedral cages with 12 pendant glycan groups.

For simplicity, we adopt the following labelling scheme for the cages. The tetrahedral cages are labelled **Co**_**4**_ or **Zn**_**4**_, according to the metal used, with the glycan type as a superscript: hence we have **Co**_**4**_^**Glu**^ (**46**), **Co**_**4**_^**Gal**^ (**47**), **Zn**_**4**_^**Glu**^ (**48**), and **Zn**_**4**_^**Gal**^ (**49**), and the octanuclear cubic cages are labelled as **Co**_**8**_^**Glu**^ (**34**), **Co**_**8**_^**Gal**^ (**35**), **Zn**_**8**_^**Glu**^ (**36**), and **Zn**_**8**_^**Gal**^ (**37**). For any cages that retain their *O-*acetyl protecting groups (*e.g.*, for ease of spectroscopic characterization given their high solubility in organic solvents), we append the superscript “Ac,” to give *e.g.*, **Co**_**4**_^**Glu-Ac**^*etc*. All of the cages of both families were characterized by high-resolution ES mass spectrometry and ^1^H NMR spectroscopy: the detailed data are shown in the SI, but Scheme 1 illustrates the NMR and mass spectroscopic data for one member of each of the tetrahedral and cubic cage families.

### Interactions of Glucose and Galactose Glycoclusters with Lectins

**Figure 1:**
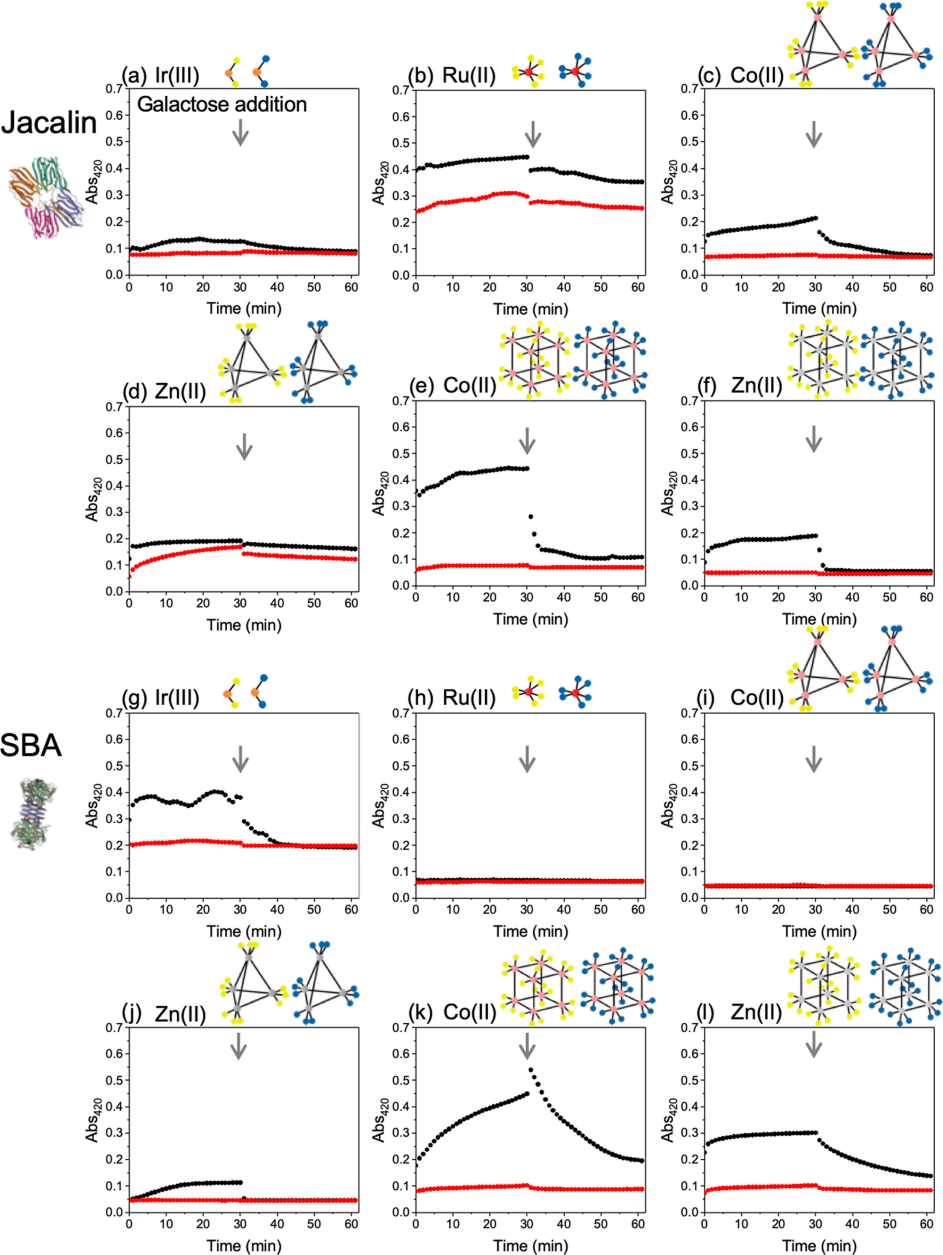
Lectin binding by turbidimetry. (Abs @ 420 nm) for a library of complexes/cages with indicated lectin. Red lines are glucose isomers, and black lines are galactose. (a–f) Jacalin; (g–l) SBA. [Lectin] = 0.01 mM, [Complex] = 0.5 mM. At the indicated point (gray arrow), a 1 M solution of free galactose was added to disrupt the binding. Complexes’ codes: (a/g) **Ir**^**Glu**^/**Ir**^**Gal**^, (b/h) **Ru**^**Glu**^/**Ru**^**Gal**^, (c/i) **Co**_**4**_^**Glu**^/**Co**_**4**_^**Gal**^; (d/j) **Zn**_**4**_^**Glu**^/**Zn**_**4**_^**Gal**^; (e/k) **Co**_**8**_^**Glu**^/**Co**_**8**_^**Gal**^; and (f/l) **Zn**_**8**_^**Glu**^/**Zn**_**8**_^**Gal**^.

To evaluate the ability of the glycan-appended cages to engage lectins, two model lectins were selected: Jacalin, which has a preference for β-d-galactose (Gal), and soybean agglutinin (SBA), which has a preference for β-d-*N*-acetyl-galactosamine (GalNAc). Initial screening using biolayer interferometry with the lectin immobilized onto the sensors^10,43^ revealed some nonspecific binding due to the high positive charge of the complexes (+2 per metal ion). Therefore, a solution-phase aggregation assay was deployed: a multivalent glycan probe, when mixed with a lectin bearing multiple binding sites, will aggregate, allowing association, which can be monitored by UV–Visible spectroscopy (turbidimetry).^44^ As these complexes/cages are colored, the optimal wavelength was first screened and it was observed that monitoring the absorbance changes at 420 nm was suitable for all of the materials explored. It should be noted that the baseline absorbance was not identical for all cages and that, *e.g.*, Ru(II) complexes have higher starting absorbance values at this wavelength from the ^1^MLCT transition: the change is what is important, and in this assay, an increase in *A*_420_ corresponds to binding. Thirty minutes after addition of the lectin, free β-d-galactose was added to disrupt the aggregates, reducing the *A*_420_ value and providing evidence of specific binding. The results are summarized for all 12 multivalent platforms in Figure 1. In all cases there was essentially zero binding to glucose-functionalized cages (red traces), which is expected as neither of the lectins used has a preference toward this monosaccharide. In contrast, the galactose-functionalized metal complex scaffolds displayed varied binding behavior according to the valency and nature of the cage/complex, leading to nonlinear responses, indicating the power of this self-assembly route to achieve multi-glycan arrays showing significant lectin binding. The larger complexes **Co**_**8**_^**Gal**^ and **Zn**_**8**_^**Gal**^ showed greater binding compared to smaller lower-valent complexes, as might be expected due to the increased number of glycans. With SBA binding, the lower-valency materials showed limited affinity, but the higher-valency M_8_ cages showed enhanced binding, which overcomes the intrinsically lower affinity, as SBA prefers GalNac to Gal. Co(II) cages showed increased responses to both lectins compared to the isostructural Zn(II) cages, demonstrating that simple metal-ion tuning can give a selective response, even with an identical number of glycans. Hence, this system demonstrates the tunability between isostructural analogues for generating a signal response to glycans.

**Figure 2:**
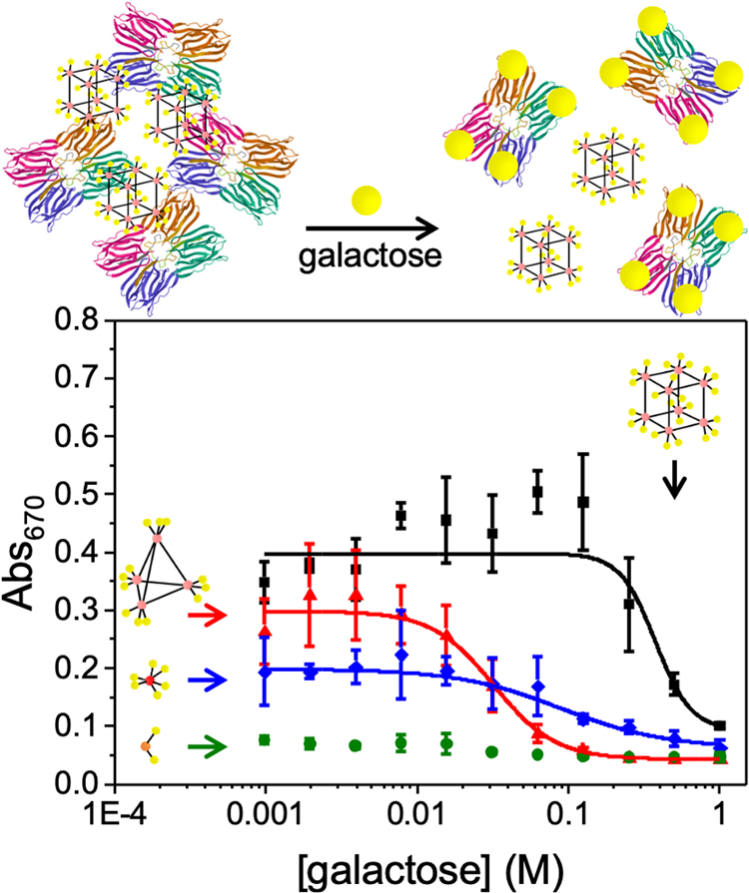
Competitive binding assay for Jacalin towards **Co**_**8**_^**Gal**^ (black), **Co**_**4**_^**Gal**^ (red), **Ru**^**Gal**^ (blue), and **Ir**^**Gal**^ (green). Monitored by *A*_670_. [Jacalin] = 0.04 mM, [Complex] = 0.5 mM. Values shown are the averages of 3 measurements.

To obtain more quantitative indications of affinity, competition experiments were performed with a subpanel of the glycosylated materials.^44^ In these assays, Jacalin was first incubated for 30 min with the indicated concentrations of competing galactose, and then the glycan-appended complexes were added and incubated for further 30 min. This allowed a dose–response curve to be generated, such that a higher concentration of galactose is required to break the complex-lectin interactions, which corresponds to higher affinity (Figure 2). This analysis showed that **Co**_**8**_^**Gal**^ required approximately 20-fold more galactose to break the interaction than **Co**_**4**_^**Gal**^, showing the multivalent enhancement in binding and the tuneable nature of this programmable assembly system. The extracted IC_50_ values from fitting to the Hill Equation are **Co**_**8**_^**Gal**^ = 0.38 M and **Co**_**4**_^**Gal**^ = 0.03 M.

### Synthesis and Characterization: Coordination-Cage-Based Glycoclusters Bearing Sialyllactose Pendants

In the previous section we demonstrated the benefit of using our self-assembly methodology to generate metal cages as scaffolds for glycoclusters with up to 24 components: the galactose-based clusters show clear evidence of affinity to galactose-binding lectins that scales with size and valency, arising from the cluster glycoside effect. However, the monosaccharides used have limited biological relevance on their own, and previous reports on Fe(II)-based supramolecular assemblies as glycocluster platforms likewise used glycans of limited biological relevance.^26^ Therefore, sialyllactose (SL) units were incorporated onto the cage exteriors. Sialic acids are of particular interest due to their role in viral adhesion/recognition processes including influenza and SARS-COV-2.^10,14,31,45^ We therefore prepared and studied members of both the M_4_L_6_ and M_8_L_12_ cage families containing, respectively, 12 or 24 SL pendant units, as one of two isomers, 3′-sialyllactose (3-SL) or 6′-sialyllactose (6-SL), to allow investigation of whether this synthesis route can tolerate increasingly complex glycans of higher biological significance.

**Figure 3:**
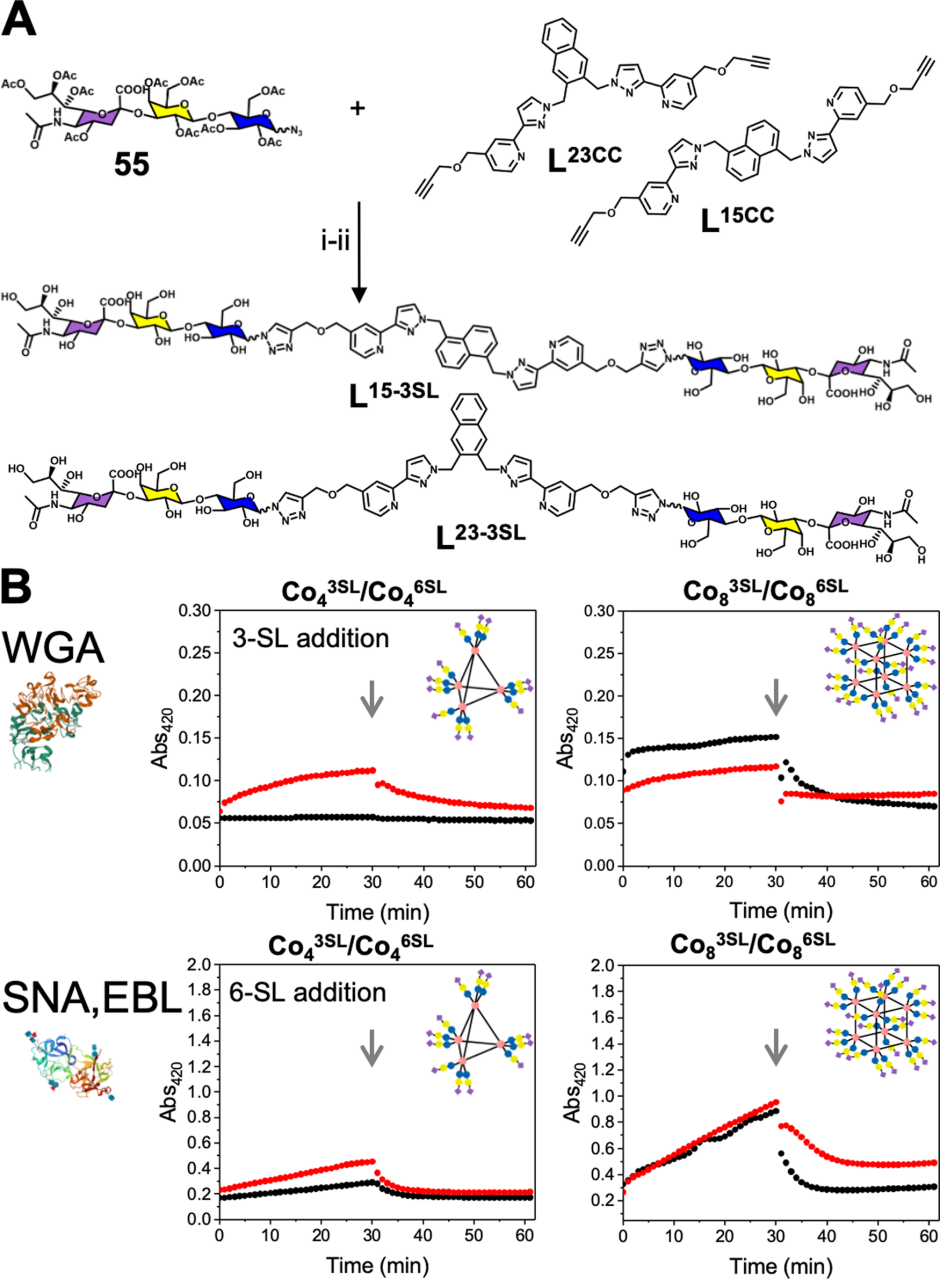
Sialyllactose-functionalized cages and their lectin binding. (A) Synthesis scheme for the preparation of ligands containing 3-SL and 6-SL pendants: **L**^**23-3SL**^ and **L**^**23-6SL**^ were used for the tetrahedral cages (**Co**_**4**_^**3SL**^, **Co**_**4**_^**6SL**^), and **L**^**15-6SL**^ and **L**^**15-3SL**^ were used for the cubic cages (**Co**_**8**_^**3SL**^, **Co**_**8**_^**6SL**^). (i) CuSO_4_·5H_2_O, sodium l-ascorbate, CH_2_Cl_2_/H_2_O (1:1), N_2_, 72 h; (ii) NaOMe (1 M in dry MeOH), r.t, 18 h. (B) Lectin binding by turbidimetry (Abs @ 420 nm) with the indicated lectins. Black lines are complexes with 3′-sialyllactose, and red lines are complexes with 6′-sialyllactose. [WGA] = 0.03 mM, [SNA, EBL] = 0.01 mM, [Complex] = 0.5 mM. At the indicated point (gray arrow), a 1 M solution of 3′ or 6′-sialyllactose was added to disrupt the binding.

The synthesis methods follow those reported previously for the mononuclear complexes: the glycan units are first connected to the ligand core *via* a Cu-AAC Click reaction between the pendant alkyne groups and acetylated 1-azido-1-deoxy-β-3′-sialyllactose (**55**) and acetylated 1-azido-1-deoxy-β-6′-sialyllactose (**56**) (Figure 3) to give ligands **L**^**15-3SL-Ac**^ (**63**), **L**^**15-6SL-Ac**^ (**64**), **L**^**23-3SL-Ac**^ (**70**), and **L**^**23-6SL-Ac**^ (**71**) (following the previous labelling scheme). Deprotection with NaOMe/MeOH removes the acetyl groups to give ligands **L**^**15-3SL**^ (**67**), **L**^**15-6SL**^ (**68**), **L**^**23-3SL**^ (**72**), and **L**^**23-6SL**^ (**73**) respectively, and combination with the relevant Co(BF_4_)_2_ generates the complete cages **Co**_**4**_^**3SL**^ (**78)**, **Co**_**4**_^**6SL**^ (**79**), **Co**_**8**_^**3SL**^ (**75**), and **Co**_**8**_^**6SL**^ (**76**). Full characterization data of the ligands (^1^H NMR and high-resolution ES-MS) are in the SI, along with the ^1^H NMR spectra of the cages.

To demonstrate that the sialic acids were available for binding to lectins, wheat-germ agglutinin (WGA) and *Sambucus nigra* agglutinin (Elderberry lectin) (SNA, EBL) were used as model lectins, as they allow cross-linking/turbidimetry experiments, similar to those performed for the monosaccharide-functionalized cages. WGA preferentially binds to *N*-acetyl-d-glucosamine units, but it can also bind to *N*-acetylneuraminic acid (Neu5Ac) units in oligosaccharides.^46^ SNA binds to Neu5Ac residues, with a preference for the disaccharide Neu5Ac(α-2,6)Gal/GalNAc over Neu5Ac(α-2,3)Gal/GalNAc.^47^

The use of **Co**_**8**_^**3SL**^ and **Co**_**8**_^**6SL**^ resulted in cross-linking (increased absorbance) with both SNA and WGA. Addition of free sialyllactose disrupted this binding, confirming a specific interaction. Overall, **Co**_**4**_^**3SL**^ and **Co**_**4**_^**6SL**^ bound the lectins equally, which was unexpected due to the lectins’ reported binding preferences. This could be due to the nature of the aggregation assay, which is semiquantitative, making it hard to extract subtle binding differences. There was, however, a clear difference in the magnitude of the response between the Co_4_ and Co_8_ cages (12 *vs* 24 glycans), with the higher-valency cubic cages showing larger responses than the smaller tetrahedral cages, again showing the cluster glycoside effect. As a further assay, the inhibition of WGA-triggered hemagglutination (erythrocyte aggregation)^48^ was probed (SI, Figure 149). This assay confirmed that the cages bearing 2,3-SL isomer pendants were more potent inhibitors of WGA-induced aggregation than cages bearing the 2,6-SL isomer pendants, agreeing with the expected selectivity,^46^ and demonstrating their potential in biological assays.

## Conclusions

We have used the self-assembly of multinuclear coordination cages to generate reproducible, perfectly monodisperse, and multivalent glycan assemblies containing 2 to 24 glycans, in well-defined three-dimensional arrays. In total, 16 glycosylated complexes/cages are reported here. Unlike other nanoscale or polymeric approaches, our assembly approach ensures that monodisperse and identical clusters can be obtained in a programmable manner, with complete control and predictability.

We first demonstrate that a series of complexes with 2, 6, 12, or 24 pendant galactose units show increased binding to target lectins as a function of the glycan valency. Using glycosylated control cages, no unspecific binding was observed. Quantitative inhibitory assays against free glycans showed that the cubic (24-galactose) cages required 20-fold greater concentrations of competing galactose to disrupt their binding compared to tetrahedral (12-galactose) cages. The synthetic methodology was further adapted to allow the incorporation of more biologically relevant trisaccharides based on sialyllactose isomers, which likewise showed a cluster glycoside effect with SL-bonding lectins, demonstrating that this methodology has broad applicability beyond simple monosaccharides.

Overall, this strategy has been shown to allow fully programmable introduction of multivalency with no dispersity, in contrast to conventional materials-chemistry approaches to glycomaterials. Small changes in glycan composition can have a major effect on the overall affinity/selectivity and hence, the cages presented here, which have no compositional dispersity, may be suitable for precision dissection of binding. These metal-containing cages also have highly characteristic photophysical properties and hence, in the future, can be developed for deployment in sensing paradigms.^30,38^

## Supporting Information Available

Full synthetic and characterization details; additional inhibition experiments (PDF)
