# Supplementary material for: Programmable Monodisperse Glyco-Multivalency Using Self-Assembled Coordination Cages as Scaffolds
Source: ACS Appl Mater Interfaces. 2023 Jul 24;15(30):36052–60. doi: 10.1021/acsami.3c08666 (PMC10401570; doi:10.1021/acsami.3c08666)
Supplement: Supplementary file 1 — am3c08666_si_001.pdf [file am3c08666_si_001.pdf]

Supporting Information For:

Programmable Monodisperse Glyco-Multivalency Using Self-Assembled Co-ordination Cages as Scaffolds

Callum Pritchard,[a,#] Melissa Ligorio,[a,#] Garrett D. Jackson,[a] Matthew I. Gibson*,[a,b] and Michael D. Ward*,[a]

[a] Department of Chemistry, University of Warwick, UK, CV47AL

[b] Division of Biomedical Sciences, Warwick Medical School, University of Warwick, UK, CV47AL

Corresponding Authors Email: m.d.ward@warwick.ac.uk, m.i.gibson@warwick.ac.uk

# Authors contributed equally

Contents

Materials and Methods 5

Synthesis of ligands for mononuclear complexes 7

Bipy^{Glu-Ac} (9) 13

Bipy^{Gal-Ac} (13) 16

Mononuclear complexes 17

Ir^{Glu-Ac} (16) 18

Ir^{Gal-Ac} (17) 20

Ir^{Glu} (18) 22

Ir^{Gal} (19) 24

Ru^{Glu-Ac} (20) 26

Ru^{Gal-Ac} (21) 28

Ru^{Glu} (22) 30

Ru^{Gal} (23) 32

NMR and MS figures 34

Synthesis of cubic cage ligands 58

L$^{15-Glu-Ac}$ (28) 58

L$^{15-Gal-Ac}$ (29) 60

L^{15-Glu} (30) 61

L^{15-Gal} (31) 62

Synthesis of cubic cages 63

[$Co_8(L^{15-Gal-Ac})_{12}(BF_4)_{16}$] [$Co_8^{Gal-Ac}$] (32) 63

[$Co_8(L^{15-Glu-Ac})_{12}(BF_4)_{16}$] [$Co_8^{Glu-Ac}$] (33) 64

[$Co_8(L^{15-Glu})_{12}(BF_4)_{16}$] [$Co_8^{Glu}$] (34) 65

[$Co_8(L^{15-Gal})_{12}(BF_4)_{16}$] [$Co_8^{Gal}$] (35) 66

[$Zn_8(L^{15-Glu})_{12}(BF_4)_{16}$] [$Zn_8^{Glu}$] (36) 67

[$Zn_8(L^{15-Gal})_{12}(BF_4)_{16}$] [$Zn_8^{Gal}$] (37) 67

[$Co_8(L^{15CC})_{12}(BF_4)_{16}$] [$Co_8^{CC}$] (38) 68

Synthesis of tetrahedral cage ligands 69

$L^{23OTIPS}$ (39) 69

L^{23OH} (40) 70

L^{23CC} (41) 71

$L^{23\text{-}Glu\text{-}Ac}$ (42) 72

$L^{23\text{-}Gal\text{-}Ac}$ (43) 73

$L^{23\text{-}Gal}$ (44) 74

$L^{23\text{-}Glu}$ (45) 75

Synthesis of tetrahedral cages 76

[$Co_4(L^{23\text{-}Glu})_6(BF_4)_8$] [$Co_4^{Glu}$] (46) 76

[$Co_4(L^{23\text{-}Gal})_6(BF_4)_8$] [$Co_4^{Gal}$] (47) 77

[$Zn_4(L^{23\text{-}Glu})_6(BF_4)_8$] [$Zn_4^{Glu}$] (48) 78

[$Zn_4(L^{23\text{-}Gal})_6(BF_4)_8$] [$Zn_4^{Gal}$] (49) 79

[$Co_4(L^{23CC})_6(BF_4)_8$] [Co_4^{CC}] (50) 80

Preparation of 1-azido-3’-sialyllactose and 1-azido-6’-sialyllactose 81

Acetylation protection 81

Bromination 82

Azido conversion 83

Esterification 84

Selected IR spectra of 3’-sialyllactose compounds and subsequent cubic cage ligands .. 87

Selected HRMS spectra of cubic cage ligands 92

Selected HRMS spectra of cages 98

Synthesis of cubic cage ligands (3’- and 6’-Sialyllactose pendants) 105

$L^{15\text{-}3SL\text{-}Ac}$ (63) 105

$L^{15\text{-}6SL\text{-}Ac}$ (64) 106

$L^{15\text{-}3SL\text{-}Ac\text{-}Me}$ (65) 107

$L^{15\text{-}6SL\text{-}Ac\text{-}Me}$ (66) 108

$L^{15\text{-}3SL}$ (67) 109

$L^{15\text{-}6SL}$ (68) 110

$L^{15\text{-}3SL\text{-}Me}$ (69) 111

Synthesis of tetrahedral cage ligands (3'- and 6'-Sialyllactose pendants) 112

$L^{23\text{-}3SL\text{-}Ac}$ (70) 112

$L^{23\text{-}6SL\text{-}Ac}$ (71) 113

$L^{23\text{-}3SL}$ (72) 114

$L^{23\text{-}6SL}$ (73) 114

Synthesis of sialyllactose-pendant cubic cages 115

$[Co_8(L^{15\text{-}3SL\text{-}Ac})_{12}(BF_4)_{16}]$ $[Co_8^{3SL\text{-}Ac}]$ (74) 115

$[Co_8(L^{15\text{-}3SL})_{12}(BF_4)_{16}]$ $[Co_8^{3SL}]$ (75) 115

$[Co_8(L^{15\text{-}6SL})_{12}(BF_4)_{16}]$ $[Co_8^{6SL}]$ (76) 115

$[Co_8(L^{15\text{-}3SL\text{-}Me})_{12}(BF_4)_{16}]$ $[Co_8^{3SL\text{-}Me}]$ (77) 116

Synthesis of sialyllactose-pendant tetrahedral cages 117

$[Co_4(L^{23\text{-}3SL})_6(BF_4)_8]$ $[Co_4^{3SL}]$ (78) 117

$[Co_4(L^{23\text{-}6SL})_6(BF_4)_8]$ $[Co_4^{6SL}]$ (79) 118

Selected NMR spectra Figures 119

Stability study of the Zn_4 tetrahedral and Zn_8 cubic cages 155

Aggregation assays 157

Turbidimetry experiments 157

Competition experiments 160

Haemagglutination inhibition assay 164

Haemolysis 165

References 166

Materials and Methods

Materials

All reagents and solvents used within the synthesis and purification were purchased from commercial sources (Sigma-Aldrich®, Fischer-Scientific®, Acros-Organics® or Fluorochem Ltd.) and used without prior purification unless otherwise stated. Dry solvents (EtOH, THF and MeOH) were transferred to Schlenk flasks and kept over pre-dried 4Å molecular sieves prior to use. 3'-Sialyllactose sodium salt and 6'-Sialyllactose sodium salt were purchased from Biosynth. SBA, Jacalin, WGA, SNA,EBL were purchased from Vector Laboratories. D-galactose and Sheep blood in Alsever's were purchased from Merck®. Ultrahigh quality water with a resistance of 18.2 MΩ.cm (at 25 ºC) was obtained from a Millipore Milli-Q gradient machine fitted with a 0.22 μM filter.

Techniques

Air sensitive reactions were performed under nitrogen or argon atmospheres using typical Schlenk techniques. ^{1}H NMR and $^{13}C\{^{1}H\}$ NMR spectra were recorded at 300, 400 or 500 MHz (^{1}H) and 75 or 125 MHz (^{13}C) respectively on Bruker Avance (300 MHz), Bruker Avance III HD (400 MHz) or Bruker Avance III HD (500 MHz) spectrometers. $^{19}F\{^{1}H\}$ NMR were also recorded on a Bruker Avance III HD (400 MHz). All NMR spectra were measured at 25°C in the indicated deuterated solvents unless stated otherwise. Proton and carbon chemical shifts (δ) are reported in ppm and coupling constants (*J*) are reported in Hertz (Hz). The resonance multiplicity in the ^{1}H NMR spectra are described as "s" (singlet), "d" (doublet), "t" (triplet), "q" (quartet), ''dd'' (doublet of doublets), ''ddd'' (doublet of doublet of doublets) and "m" (multiplet) and broad resonances are indicated by "br".

2D homonuclear correlation ^{1}H-^{1}H COSY and 2D heteronuclear correlation ^{1}H-^{13}C HETCOR experiments (HMQC, HMBC) were used to confirm NMR peak assignments. Infrared spectra were recorded on a Bruker Alpha IR-PLATINUM-ATR spectrophotometer using solid samples. Accurate mass measurements (ESI-HRMS) were performed on a Bruker maXis plus LC/ESI/MS instrument in positive-ion mode. Either protonated molecular ions $[M+nH]^{n+}$ or sodium adducts $[M+Na]^{+}$ were used for empirical formula confirmation. Elemental analyses for carbon, hydrogen and nitrogen were performed on a FlashEA 1112 CH&N elemental analyser by MEDAC Ltd. Chobham, Surrey GU24, 8JB, UK.

Fluorescence measurements were collected using an Agilent Cary Eclipse fluorimeter and UV/Vis spectra were obtained using an Implen C40 Nanophotometer. Purifications by column chromatography were performed using either silica gel (Fluorochem Ltd, 60Å, 40-63μ) or Brockmann III aluminium oxide (Sigma-Aldrich®). Size exclusion chromatography was performed on Sephadex LH-20® or Sephadex G-50®. The purities of the products were established by thin-layer chromatography (TLC) on either silica gel coated aluminium plates (with F253 indicator; layer thickness, 200 μm; particle size, 2-25 μm; pore size 60 Å) or aluminium oxide coated aluminium backed plates (with F253 indicator, layer thickness, 1500 μm; particle size, pore size 150 Å).

Synthesis of ligands for mononuclear complexes

[2,2'-Bipyridine]-4,4'-dicarboxylic acid (**2**)

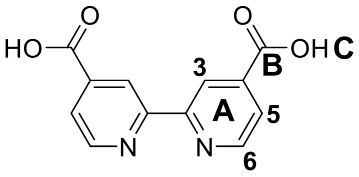

Compound **2** was synthesised according to a known literature method.[1]

A round bottomed flask charged with a solution of 4.4'-dimethyl-2,2'-bipyridine (**1**) (8.00 g, 43.4 mmol 1.0 equiv.) in concentrated H_2SO_4 (100 ml) was cooled to 0 °C in an ice bath for 30 mins. To this, $K_2Cr_2O_7$ (39.32 g, 130 mmol, 3.0 equiv.) was slowly added in 5 g portions over a 15 min period, producing a dark green solution overtime. Once complete, the solution was heated to 70 °C for 4 h before cooling to room temperature and pouring over an ice slurry. The white precipitate was collected, filtered and washed with acetone (250 ml) then dried under high vacuum. Data recorded was in good agreement with the reported literature.[1]

Yield: 10.25 g, 41.9 mmol, 97% (white powder).

^{1}H NMR (400 MHz, DMSO-d_6, 298K) δ_H: 13.29 (br(s), H^C, 2H), 8.92 (d, $^3J_{HH}$ = 4.9 Hz, H^{A6}, 2H), 8.85 (s, H^{A3}, 2H), 7.92 – 7.90 (dd, $^3J_{HH}$ = 4.9 Hz, $^4J_{HH}$ = 1.5 Hz, H^{A5}, 2H).

ESI-MS (+) (*m/z*): 245.3 $[M+H]^+$, 267.3 $[M+Na]^+$.

Diethyl [2,2'-bipyridine]-4,4'-dicarboxylate (**3**)

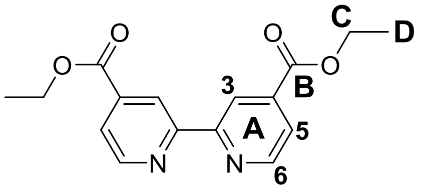

Compound **3** was synthesised according to a known literature method.[2]

2 (10.86 g, 44.5 mmol) was dissolved in EtOH (200 ml) producing a white suspension. To this, concentrated H_2SO_4 (20 ml) was added slowly, and the mixture was refluxed for 24 h, with a pink colour change being observed overtime.

Once cooled to room temperature, the solution was poured over an ice slurry, precipitating out a white powder. Product was collected by filtration and dried under high vacuum. Data recorded was in good agreement with the reported literature.[2]

Yield: 12.33 g, 41.1 mmol, 92% (white powder).

^{1}H NMR (400 MHz, DMSO-d_6, 298K) δ_H: 8.84 (d, $^3J_{HH}$ = 4.9 Hz, H^{A6}, 2H), 8.23 (s, H^{A3}, 2H), 7.89 (d, $^3J_{HH}$ = 4.9 Hz, H^{A5}, 2H), 4.45 (q, $^3J_{HH}$ = 7.2 Hz, $^4J_{HH}$ = 6.9 Hz, H^C, 4H), 1.42 (s, H^D, 6H).

ESI-MS (+) (*m/z*): 301.2 [M+H]$^+$, 323.2 [M+Na]$^+$.

4,4’-Bis(hydroxymethyl)-[2,2’-bipyridine] (**4**)

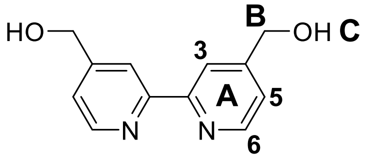

Compound **4** was synthesised according to a known literature method.[3]

$NaBH_4$ (18.01 g, 476 mmol, 22 equiv.) was slowly added in 3 g portions to a round-bottomed flask charged with **3** (6.50 g, 21.6 mmol, 1.0 equiv.) in dry EtOH (300 ml), with effervescence being observed. The mixture was then refluxed for 24 h under N_2, with a purple to orange colour change being observed overtime. Once cooled to room temperature, a satd. Solution of NH_4Cl (200 ml) and H_2O (200 ml) was added, and the mixture stirred under gentle heating until all the beige solid had dissolved. EtOAc (4 x 500 ml) was then added, and the product was extracted into the organic phase. Combined organic phases were dried over $MgSO_4$ and evaporated to give a light-yellow powder, which was further dried under high vacuum. Data recorded was in good agreement with the reported literature.[3]

Yield: 7.83 g, 36.2 mmol, 84% (light yellow powder).

^{1}H NMR (400 MHz, DMSO-d_6, 298K) δ_H: 8.60 (d, $^3J_{HH}$ = 4.9 Hz, H^{A6}, 2H), 8.39 (s, H^{A3}, 2H), 7.37 (d, $^3J_{HH}$ = 4.9 Hz, H^{A5}, 2H), 5.50 (t, $^3J_{HH}$ = 5.7 Hz, H^C, 2H), 4.64 (d, $^3J_{HH}$ = 5.6 Hz, H^B, 2H).

ESI-MS (+) (*m/z*): 217.2 [M+H]$^+$, 239.2 [M+Na]$^+$.

4,4'-Bis((prop-2-yn-1-yloxy)methyl)-2,2'-bipyridine (**5**)

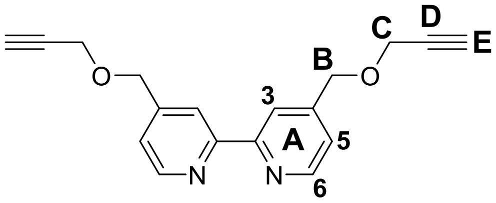

NaH (527 mg, 13.2 mmol, 2.5 equiv., 60 wt. % dispersion in mineral oil) was added to a flame-dried two necked round-bottomed flask charged with dry THF (250 ml) and stirred for 30 mins under N_2. To this grey suspension, 15-crown-5 ether (2.1 ml, 2.32 g, 10.5 mmol, 2.0 equiv.) was added and the resulting grey slurry was stirred for another 30 mins. A solution of **4** (1.14 g, 5.27 mmol, 1 equiv.) in dry THF (40 ml) was then added slowly, and the resulting beige suspension was refluxed for 1 h before the slow addition of propargyl bromide (1.70 ml, 15.8 mmol, 3.0 equiv., 80 wt. % in toluene) in dry THF (30 ml). Refluxing was then sustained for 24 h, with a beige to dark brown colour change being rapidly observed overtime. Once cooled to room temperature, the reaction was quenched with the dropwise addition of MeOH (10 ml) and the solvents evaporated to give a brown/black solid. Mineral oil was removed by filtration through a celite plug. The product was then purified by column chromatography on Al_2O_3, eluting with CH_2Cl_2/MeOH (98:2, v/v) to give a light brown powder.

Yield: 1.08 g, 3.69 mmol, 70% (brown powder).

^{1}H NMR (500 MHz, $CDCl_3$, 298K) δ_H: 8.67 – 8.65 (dd, $^3J_{HH}$ = 5.0 Hz, $^4J_{HH}$ = 1.0 Hz, H^{A6}, 2H), 8.34 (q, $^3J_{HH}$ = 1.0 Hz, H^{A3}, 2H), 7.35 - 733 (dt, $^3J_{HH}$ = 5 Hz, $^4J_{HH}$ = 1.0 Hz, H^{A5}, 2H), 4.70 (s, H^B, 2H), 4.26 (d, $^3J_{HH}$ = 2.4 Hz, H^C, 2H), 2.49 (t, $^3J_{HH}$ = 2.5 Hz, H^E, 2H).

^{13}C{^{1}H} NMR (125 MHz, $CDCl_3$, 298K) δ_C: 156.1 (C^{A4}), 149.4 (C^{A2}), 147.8 (C^{A6}), 122.1 (C^{A3}), 119.5 (C^{A5}), 77.0 (C^D), 75.2 (C^E), 70.0 (C^B), 57.9 (C^C).

ESI-HRMS (+) (*m/z*): 293.1285 calculated for $C_{18}H_{17}N_2O_2$ $[M+H]^+$, 293.1286 found for $[M+H]^+$.

IR (Solid/ cm^{-1}): $\tilde{\upsilon}$ 3237 (w), 2886 (w), 2101 (s), 1601 (s), 1352 (s), 1069 (s), 822 (s).

Elemental analysis. Calcd for $C_{18}H_{16}N_2O_2$: C 73.95, H 5.52, N 9.58. **Found**: C 73.66, H 5.34, N 9.54%.

2,3,4,6-Tetra-O-acetyl-α-D-glucopyranosyl bromide (**7**)

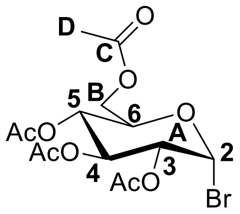

Compound **7** was synthesised according to a known literature method.[4]

To a stirred 0 °C solution of HBr (25 ml, 33 wt. % in acetic acid), *α*-D-glucose pentaacetate (**6)** (10.9 g, 28 mmol, 1.0 equiv.) was slowly added in 1 g portions over a 30 min period under N_2. The dark orange solution was then allowed to warm back up to room temperature then stirred for a further hour, until TLC confirmation of the total consumption of the starting material. The reaction mixture was then poured into ice-water and the resulting opaque goo extracted into CH_2Cl_2 (2 x 200 ml). The combined organic extracts were washed with a cold solution of sat. $NaHCO_3$ (2 x 100 ml) then dried over $MgSO_4$. Solvent was removed under low pressure, producing a sticky clear syrup which partially solidified under high vacuum and is essentially pure glucopyranosyl bromide. Data recorded was in good agreement with the reported literature.[4]

Yield: 9.51 g, 23.1 mmol, 82% (clear syrup).

^{1}H NMR (400 MHz, $CDCl_3$, 298K) δ_H: 6.58 (d, $^3J_{HH}$ = 4.2 Hz, H^{A2}, 1H), 5.53 (t, $^3J_{HH}$ = 9.9 Hz, H^{A3}, 1H), 5.17 (t, $^3J_{HH}$ = 9.6 Hz, H^{A5}, 1H), 4.84 – 4.82 (dd, $^3J_{HH}$ = 9.9, $^4J_{HH}$ = 4.2 Hz, H^{A6} 1H), 4.36 - 4.27 (m, H^B, 2H), 4.16 - 4.11 (m, H^{A6}, 1H), 2.11 - 2.04 (s, H^D, 12H).

ESI-MS (+) (*m/z*): 433.4 [*M*+Na]$^+$.

1-Azido-1-deoxy-β-D-glucopyranoside tetraacetate (**8**)

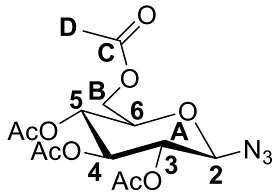

Compound **8** was synthesised according to a known literature method.[5]

To a round bottom flask (50 ml) containing **7** (2.70 g, 6.56 mmol, 1.0 equiv.), magnetic stirrer bar and rubber septum, a mixture of acetone/H_2O (20 ml, 5:1, v/v) was added to give a clear solution. NaN_3 (2.02 g, 31.1 mmol, 4.7 equiv.) was then slowly added portion wise over a 10 min period. Solution was left to stir over night under a N_2 atmosphere. After 18 hrs, TLC using hexanes/EtOAc (1:1, v/v) indicated a near complete conversion of starting material.

Acetone was then carefully removed under low pressure and the residual grey coloured goo partitioned between H_2O (50 ml) and EtOAc (50 ml). Organic layer was removed, and the aqueous layer extracted with EtOAc (2 x 50 ml). Combined organic extracts were dried over anhydrous Na_2SO_4, filtered and evaporated to give a sticky clear syrup. Recrystallization using MeOH produced a white crystalline solid. Data recorded was in good agreement with the reported literature.[5]

Yield: 2.25 g, 6.03 mmol, 92% (white crystalline solid).

^{1}H NMR (400 MHz, $CDCl_3$, 298K) δ_H: 5.21 (t, $^3J_{HH}$ = 9.4 Hz, H^{A3}, 1H), 5.09 (t, $^3J_{HH}$ = 9.8 Hz, H^{A4}, 1H), 4.95 (t, $^3J_{HH}$ = 9.0 Hz, H^{A2}, 1H), 4.64 (d, $^3J_{HH}$ = 8.9 Hz, H^{A1}, 1H), 4.29 – 4.24 (dd, $^3J_{HH}$ = 12.5 Hz, $^4J_{HH}$ = 4.8 Hz, H^{Bb}, 1H), 4.18 – 4.13 (dd, $^3J_{HH}$ = 12.5, $^4J_{HH}$ = 2.3 Hz, H^{Ba}, 1H), 3.82 – 3.76 (ddd, $^3J_{HH}$ = 9.9 Hz, $^4J_{HH}$ = 4.7 Hz, $^5J_{HH}$ = 2.4 Hz, H^{A5}, 1H), 2.09, 2.07, 2.02, 1.99 (s, H^D, 12H).

ESI-MS (+) (*m/z*): 373.3 $[M+H]^+$, 396.2 $[M+Na]^+$.

Bipy$^{Glu\text{-}Ac}$ (9)

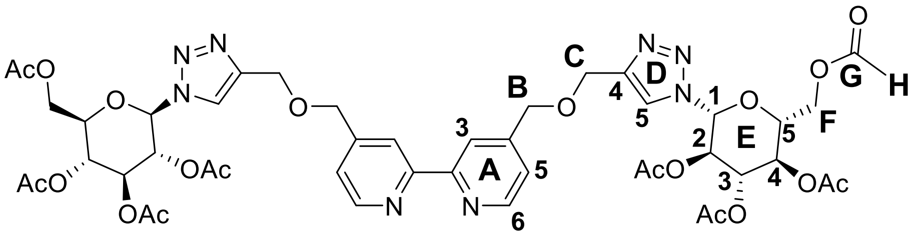

To a round bottom flask charged with **8** (232.9 mg, 0.62 mmol, 3.4 equiv.), **5** (52.1 mg, 0.18 mmol, 1.0 equiv.) in CH_2Cl_2 (10 ml) was added and the dark brown reaction mixture was left to stir for 10 mins. A solution of $CuSO_4$•$5H_2O$ (16 mg, 64 μmol, 0.35 equiv.) and NaAsc (31.4 mg, 0.16 mmol, 0.80 equiv.) in water (10 ml) was then added to this mixture, forming a biphasic system. The mixture was then heated continuously for 72 h at 60 °C, with a colour change from orange to dark red overtime.

The reaction was quenched by the addition of an $EDTA_{aq}$ (10 ml, 1.5M) solution with water (10 ml) and left to stir overnight. The organic phase was separated, and the aqueous phase extracted with CH_2Cl_2 (3 x 10 ml). Combined organic phases were dried over Na_2SO_4 and evaporated to give a dark yellow sticky solid. The product was purified on a short Sephadex LH-20 column using a $CHCl_3$/MeOH (99:1, v/v), eluting as the first fraction. Isolation yielded a pale-yellow sticky solid, which was further dried under vacuum to a flaky solid.

Yield: 122 mg, 0.12 mmol, 66% (yellow flaky solid).

^{1}H NMR (500 MHz, $CDCl_3$, 298K) δ_H: 8.64 (d, $^3J_{HH}$ = 3.7 Hz, H^{A6}, 2H), 8.35 (s, H^{A3}, 2H), 7.85 (s, H^{D5}, 2H), 7.35 (d, $^3J_{HH}$ = 3.4 Hz, H^{A5}, 2H), 5.90 – 5.86 (m, H^{E1}, 2H), 5.48 – 5.39 (dt, $^3J_{HH}$ = 15.3 Hz, J = 13.6 Hz, H^{E2} + H^{E3}, 4H), 5.25 – 5.21 (m, H^{E4}, 2H), 4.75 (s, H^C, 4H), 4.67 (s, H^B, 4H), 4.29 – 4.25 (dd, $^3J_{HH}$ = 12.7 Hz, $^4J_{HH}$ = 4.9 Hz, H^{Fb}, 2H), 4.14 – 4.10 (dd, $^3J_{HH}$ = 15 Hz , $^4J_{HH}$ = 3.3 Hz, H^{Fa}, 2H), 4.01 – 3.99 (m, H^{E5}, 2H), 2.06, 2.05, 2.02, 1.86 (s, H^H, 24H).

^{13}C{^{1}H} NMR (125 MHz, $CDCl_3$, 298K) δ_H: 170.5, 169.9, 169.4, 168.9 (C^G), 156.1 (C^{A2}), 149.4 (C^{A6}), 148.0 (C^{D5}), 145.5 (C^{A4}), 122.1 (C^{A5}), 121.6 (C^{D4}), 119.5 (C^{A3}), 85.8 (C^{E1}), 75.2 (C^{E5}), 72.6 (C^{E2}), 70.8 (H^C), 70.4 (C^{E3}), 67.7 (C^{E4}), 63.9 (C^B), 61.5 (C^F), 20.7, 20.5, 20.2 (C^H).

ESI-HRMS (m/z): 1039.3527 calculated for $C_{46}H_{55}N_8O_{20}$ [M+H]$^+$, 1039.3515 found for [M+H]$^+$.

IR (Solid/ cm^{-1}): $\tilde{\upsilon}$ 3140 (w), 2933 (w), 1743 (s), 1597 (w), 1208 (s), 1042 (s), 921 (m), 826 (m), 589 (m).

Elemental analysis. Calcd for $C_{46}H_{54}N_8O_{20}$: C 53.18, H 5.24, N 10.79. **Found**: C 53.34, H 5.16, N 10.70%.

2,3,4,6-Tetra-O-acetyl-α-D-galactopyranosyl bromide (**11**)

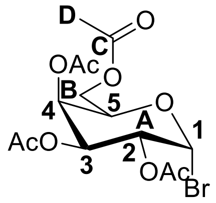

Compound **11** was synthesised using an identical method to **7**, substituting **6** for *α*-D-galactose pentaacetate **10** (5.01 g, 12.8 mmol, 1.0 equiv.). Data recorded was in good agreement with the reported literature.[6]

Yield: 5.27g, 12.8 mmol, 99% (beige solid).

^{1}H NMR (400 MHz, $CDCl_3$, 298K) δ_H: 6.69 (d, $^3J_{HH}$ = 3.9 Hz, H^{A1}, 1H), 5.51 – 5.47 (dd, $^3J_{HH}$ = 3.2 Hz, $^4J_{HH}$ = 1.1 Hz, H^{A4}, 1H), 5.42 – 5.38 (dd, $^3J_{HH}$ = 10.6 Hz, $^4J_{HH}$ = 3.3 Hz, H^{A2}, 1H), 5.07 – 5.02 (dd, $^3J_{HH}$ = 10.6 Hz, $^4J_{HH}$ = 3.9 Hz, H^{A3}, 1H), 4.48 (t, $^3J_{HH}$ = 6.5 Hz, H^{A5}, 1H), 4.21 – 4.07 (m, H^B, 2H), 2.15, 2.11, 2.05, 2.01 (s, H^D, 12H).

ESI-MS (+) (*m/z*): 433.2 [*M*+Na]$^+$.

1-Azido-1-deoxy-β-D-galactopyranoside tetraacetate (**12**)

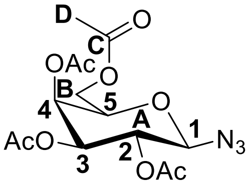

Compound **12** was synthesised using an identical method to **8**, substituting **7** for **11** (2.61 g, 6.34 mmol, 1.0 equiv.). Data recorded was in good agreement with the reported literature.[7]

Yield: 2.25 g, 6.03 mmol, 95% (white crystalline solid).

^{1}H-NMR (400 MHz, $CDCl_3$, 298K) δ_H: 5.41 – 5.38 (dd, $^3J_{HH}$ = 3.3 Hz, $^4J_{HH}$ = 1.0 Hz, H^{A3}, 1H), 5.18 – 5.12 (dd, $^3J_{HH}$ = 10.4 Hz, $^4J_{HH}$ = 8.7 Hz, H^{A4}, 1H), 5.05 – 5.00 (dd, $^3J_{HH}$ = 10.3 Hz, $^4J_{HH}$ = 3.3 Hz, H^{A2}, 1H), 4.59 (d, $^3J_{HH}$ = 8.6 Hz, H^{A1}, 1H), 4.17 – 4.14 (dd, $^3J_{HH}$ = 7.0 Hz, $^4J_{HH}$ = 2.5 Hz, H^B, 2H), 4.03 – 3.98 (m, H^{A5}, 1H), 2.16, 2.08, 2.05, 1.98 (s, H^D, 12H).

ESI-MS (+) (*m*/*z*): 396.2 [M+Na]$^+$.

Bipy^{Gal-Ac} (13)

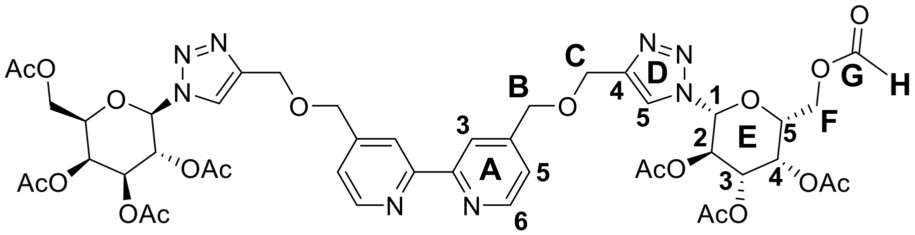

This compound was synthesised using an identical method to **Bipy^{Glu-Ac}**, substituting **8** for **12** (100 mg, 0.27 mmol, 3.4 equiv.).

Yield: 59 mg, 57 µmol, 72% (yellow glass like solid).

^{1}H NMR (500 MHz, $CDCl_3$, 298K) δ_H: 8.64 (d, $^3J_{HH}$ = 4.9 Hz, H^{A6}, 2H), 8.35 (s, H^{A3}, 2H), 7.90 (s, H^{D5}, 2H), 7.35 (d, $^3J_{HH}$ = 4.6 Hz, H^{A5}, 2H), 5.85 (d, $^3J_{HH}$ = 9.3 Hz, H^{E1}, 2H), 5.54 (t, $^3J_{HH}$ = 10.6 Hz, H^{E2} + H^{E4}, 4H), 5.25 – 5.20 (dd, $^3J_{HH}$ = 10.3 Hz, $^4J_{HH}$ = 3.3Hz, H^{E3}, 2H), 4.76 (s, H^C, 4H), 4.67 (s, H^B, 4H), 4.25 – 4.09 (m, H^{Fab} + H^{E5}, 6H), 2.20, 2.03, 1.99, 1.88 (s, H^H, 24H).

^{13}C{^{1}H} NMR (125 MHz, $CDCl_3$, 298K) δ_C: 170.3, 169.9, 169.8, 169.1 (C^G), 156.1 (C^{A2}), 149.4 (C^{A6}), 148.1 (C^{A4}), 145.4 (C^{D5}), 122.1 (C^{A5}), 121.3 (C^{D4}), 119.5 (C^{A3}), 86.3 (C^{E1}), 74.1 (C^{E5}), 70.8 (C^B), 70.7 (C^{E3}), 67.9 (C^{E2}), 66.9 (C^{E4}), 64.0 (C^C), 61.2 (C^F), 20.6, 20.5, 20.3 (C^H).

ESI-HRMS (+) (*m/z*): 1039.3527 calculated for $C_{46}H_{55}N_8O_{20}$ [M+H]$^+$, 1039.3526 found for [M+H]$^+$.

IR (Solid/ cm^{-1}): $\tilde{\upsilon}$ 3062 (w), 2942 (w), 1740 (s), 1597 (w), 1367 (m), 1212 (s), 1034 (s), 922 (m), 825 (m), 597 (m).

Elemental analysis. Calcd for $C_{46}H_{54}N_8O_{20}$: C 53.18, H 5.24, N 10.79. **Found**: C 53.40, H 5.18, N 10.74%.

Mononuclear complexes

[{Ir(F_2ppy)$_2$(μ–Cl)}$_2$] (**15**)

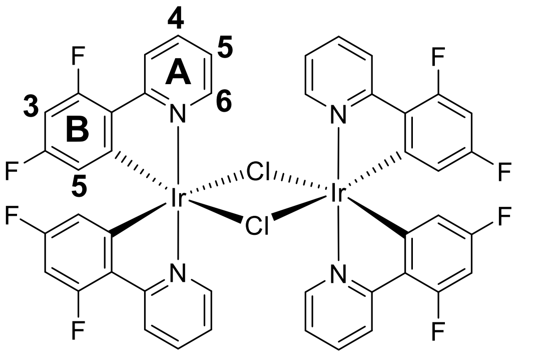

15 was synthesised according to a known literature method.[8]

2-(2,4-Difluorophenyl)pyridine (**14**) (325 mg, 1.70 mmol, 2.0 equiv.) and $IrCl_3.3H_2O$ (303 mg, 0.860 mmol, 1.0 equiv.) were added to a round bottomed flask and a N_2 degassed solution of 2-ethoxyethanol/H_2O (15 ml, 3:1, v/v) was added, forming a brown mixture. Mixture was then refluxed for 24 h under N_2, with an orange to yellow colour change being observed overtime, alongside the formation of a yellow precipitate. Reaction was then cooled to room temperature, filtered and washed successively with H_2O (30 ml) and Et_2O (30 ml). Fine yellow solid was isolated and dried under high vacuum. Data recorded was in good agreement with the reported literature.[8]

Yield: 419 mg, 0.345 mmol, 81% (yellow powder).

^{1}H NMR (400 MHz, $CDCl_3$, 298K) δ_H: 9.12 – 9.10 (dd, $^3J_{HH}$ = 5.8 Hz, $^4J_{HH}$ = 0.93 Hz, H^{A6}, 4H), 8.31 (d, $^3J_{HH}$ = 8.4 Hz, H^{A3}, 4H), 7.82 (t, $^3J_{HH}$ = 7.8 Hz, H^{A4}, 4H), 6.83 (dt, $^3J_{HH}$ = 7.2 Hz, $^4J_{HH}$ = 1.2 Hz, H^{A5}, 4H), 6.38 – 6.29 (m, H^{B3}, 4H), 5.29 (dd, $^3J_{HH}$ = 9.1 Hz, $^4J_{HH}$ = 2.3 Hz, H^{B5}, 4H).

ESI-MS (+) (*m/z*): 1217.8 [M+H]$^+$, 1239.8 [M+Na]$^+$.

Ir$^{Glu\text{-}Ac}$ (16)

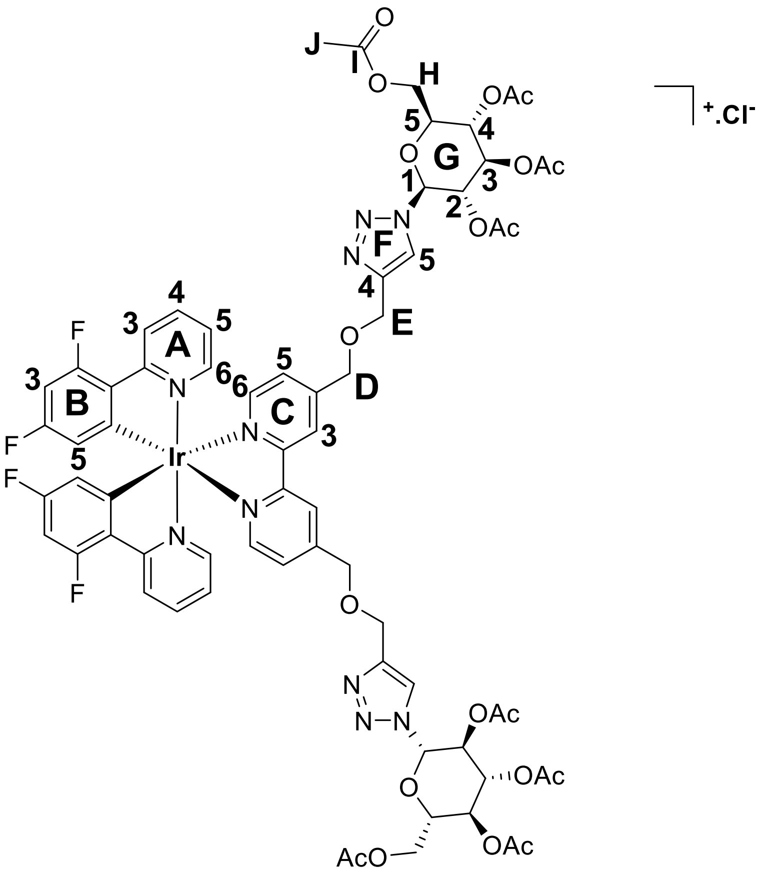

15 (58.5 mg, 48 µmol, 1.0 equiv.) and **Bipy$^{Glu\text{-}Ac}$** (**9**) (100 mg, 96 µmol, 2.0 equiv.) were added sequentially to a round bottom flask and subsequently dissolved up in mixture of MeOH/CH_2Cl_2 (10 ml, 1:1, v/v). This bright yellow mixture was then left to stir at room temperature for 3 h. Solvents were removed under low pressure to give a golden yellow precipitate, which was purified by size-exclusion chromatography using Sephadex LH-20, eluting with MeOH:CH_2Cl_2 (30:70, v/v) slowly to give a golden yellow powder.

Yield: 63 mg, 38 µmol, 80% (golden yellow powder).

^{1}H NMR (500 MHz, CD_3OD, 298K) δ_H: 8.75 (s, H^{C3}, 2H), 8.38 - 8.35 (m, H^{F5} + H^{A6}, 4H), 7.97 – 7.93 (m, H^{C6} + H^{A5}, 4H), 7.69 (d, $^3J_{HH}$ = 4.9 Hz, H^{A3}, 2H), 7.59 (s, H^{C5}, 2H), 7.14 (m, H^{A4}, 2H), 6.68 – 6.65 (ddd, $^3J_{HF}$ = 9.6, 9.2 Hz, H^{B3}, 2H), 6.19 – 6.16 (m, H^{G1}, 2H), 5.71 – 5.68 (dd, $^3J_{HF}$ = 9.2, $^4J_{HH}$ = 2.4 Hz, H^{B5}, 2H), 5.62 (t, $^3J_{HH}$ = 9.1 Hz, H^{G2}, 2H), 5.57 – 5.53 (m, H^{G3}, 2H), 5.30 – 5.26 (m, H^{G4}, 2H), 4.82 (s, E, 4H), 4.81 (s, D, 4H), 4.33 – 4.26 (m, H^H, 4H), 4.17 (m, H^{G5}, 2H), 2.06, 2.01, 1.99 and 1.78 (s, H^J, 24H).

^{13}C{^{1}H} NMR (125 MHz, CD_3OD, 298K) δ_C: 172.2, 171.4, 171.3, 170.5 (C^I), 164.7 - 163.5 (d, J_{CF} = 13.0 Hz, C^{B4}), 164.2 (d, J_{CF} = 7.0 Hz, C^{A2}), 161.7 - 160.0 (dd, J_{CF} = 21 Hz, 13.0 Hz, C^{B2}), 155.4 (C^{C2}), 154.0 (d, J_{CF} = 7.0 Hz, C^{C1}), 151.2 (C^{C6}), 150.3 (C^{A3}), 143.8 (C^{F4}), 140.5 (C^{A5}), 127.6 (C^{B6}), 127.4 (C^{C5}), 127.2 (C^{C4}), 124.8 (C^{A6}), 123.8 (C^{C3}), 123.2 (C^{A4}), 122.9 (C^{F5}), 113.4 (d, J_{CF} = 18.0 Hz, C^{B5}), 98.5 (t, J_{CF} = 27.0 Hz, C^{B3}), 86.7 (C^{G1}), 74.1 (C^{G3}), 72.1 (C^{G2}), 70.6 (E), 69.3 (C^{G4}), 64.8 (D), 64.6 (C^H), 63.7 (C^{G5}), 20.6, 20.58, 20.56, 20.2 (C^J).

^{19}F{^{1}H} NMR (400 MHz, CD_3OD, 298K) δ_F: (-108.7) – (-108.3) (m, 2F), -110.9 (d, J_{CF} = 12 Hz, 2F).

ESI-HRMS (+) (*m/z*): 1611.4021 calculated for $C_{68}H_{66}F_4IrN_{10}O_{20}$ $[M]^+$, found 1611.4044 for $[M]^+$.

IR (Solid/ cm^{-1}): $\tilde{\upsilon}$ 3294 (m)(br), 3201 (m), 2975 (w), 2812 (w), 1632 (m), 1602 (s), 1582 (s), 1472 (s), 1423 (m), 1231 (m), 1123 (m), 1075 (m), 990 (s).

Elemental analysis. Calcd for $C_{68}H_{66}ClF_4IrN_{10}O_{20}.H_2O$: C 49.05, H 4.12, N 8.41. **Found**: C 49.32, H 4.01, N 8.17%.

Ir$^{Gal\text{-}Ac}$ (17)

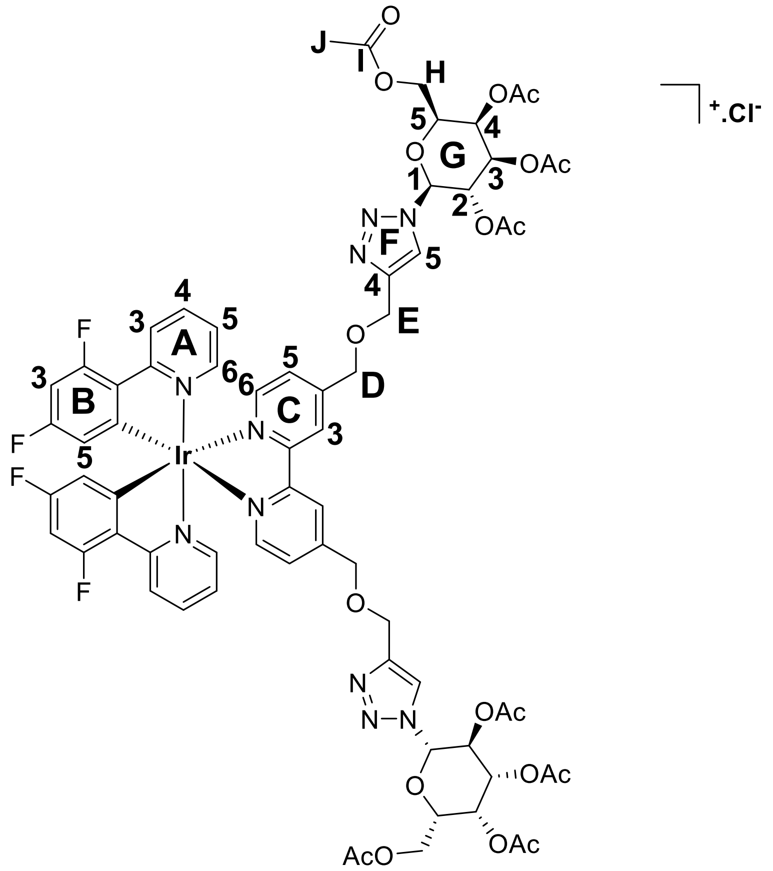

This complex was synthesised using an identical method to **Ir$^{Glu\text{-}Ac}$** (**16**), substituting **Bipy$^{Glu\text{-}Ac}$** (**9**) for **Bipy$^{Gal\text{-}Ac}$** (**13**) (50 mg, 48 μmol, 2.0 equiv.).

Yield: 32.0 mg, 19 μmol, 81% (golden yellow powder).

^{1}H NMR (500 MHz, CD_3OD, 298K) δ_H: 8.76 (d, $^3J_{HH}$ = 6.3 Hz, H^{C3}, 2H), 8.36 – 8.33 (m, H^{F5} + H^{A6}, 4H), 7.97 – 7.93 (m, H^{C6} + H^{A5}, 4H), 7.69 (t, $^3J_{HH}$ = 4.8 Hz, H^{A3}, 2H), 7.59 (d, $^3J_{HH}$ = 5.4 Hz, H^{C5}, 2H), 7.15 (t, $^3J_{HH}$ = 6.4 Hz, H^{A4}, 2H), 6.69 – 6.67 (ddd, $^3J_{HF}$ = 9.6, 9.2 Hz, $^4J_{HH}$ = 2.4 Hz, H^{B3}, 2H), 6.15 – 6.11 (m, H^{G1}, 2H), 5.71 (dd, J_{HF} = 9.2 Hz, J_{HH} = 2.4 Hz, H^{B5}, 2H), 5.65 (t, $^3J_{HH}$ = 10.5 Hz, H^{G2}, 2H), 5.56 (s, H^{G4}, 2H), 5.45 (dd, $^3J_{HH}$ = 10.0 Hz, $^4J_{HH}$ = 3.3 Hz, H^{G3}, 2H), 4.84 (s, E, 4H), 4.82 (s, D, 4H), 4.49 (q, $^3J_{HH}$ = 11.5 Hz, $^4J_{HH}$ = 5.6 Hz, H^{G5}, 2H), 4.24 – 4.09 (m, H^H, 4H), 2.19, 1.99, 1.98, 1.80 (s, H^J, 24H).

^{13}C{^{1}H} NMR (125 MHz, CD_3OD, 298K) δ_C: 170.6, 170.4, 169.8, 169.3 (H^I), 164.8 (d, $J_{C\text{-}F}$ = 12.8 Hz, C^{B4}), 164.0 (d, J_{CF} = 7.0 Hz, C^{A2}), 162.8 – 162.5 (dd, J_{CF} = 23 Hz, 12.0 Hz, C^{B2}), 160.5 (d, J_{CF} = 7.0 Hz, C^{B1}), 155.2 (C^{C2}), 151.6 (C^{A6}), 150.3 (C^{A3}), 144.6 (C^{F4}), 140.2 (C^{A5}), 127.8 (C^{B6}), 127.5 (C^{C5}), 127.4 (C^{C4}), 124.7 (C^{A6}), 124.0 (C^{C3}), 123.8 (C^{A4}), 122.1 (C^{F5}), 113.4

(d, J_{CF} = 18.0 Hz, C^{B5}), 98.5 (t, J_{CF} = 27.0 Hz, C^{B3}), 86.9 (C^{G1}), 75.2 (C^{G5}), 72.0 (C^{G3}), 70.6 (E), 69.8 (C^{G2}), 68.4 (C^{G4}), 64.4 (D), 62.5 (C^{H}), 19.2, 19.1, 19.0. 18.8 (H^{J}).

^{19}F{^{1}H} NMR (400 MHz, CD_3OD, 298K) δ_F: (-108.7) – (-108.3) (m, 2F), -110.9 (d, $^{4}J_{FF}$ = 12 Hz, 2F).

ESI-HRMS (+) (*m/z*): 1611.4021 calculated for $C_{68}H_{66}F_4IrN_{10}O_{20}$ $[M]^+$, 1611.3994 found for $[M]^+$.

IR (Solid/ cm^{-1}): $\tilde{\upsilon}$ 3310 (m)(br), 3205 (m), 3070 (m), 2922 (w), 2872 (w), 1659 (m), 1599 (s), 1573 (s), 1477 (s), 1403 (m), 1246 (m), 1163 (m), 1091 (m), 987 (s).

Elemental analysis. Calcd for $C_{68}H_{66}ClF_4IrN_{10}O_{20}.H_2O$: C 49.05, H 4.12, N 8.41. **Found**: C 49.44, H 4.10, N 8.29 %.

Ir^{Glu} (18)

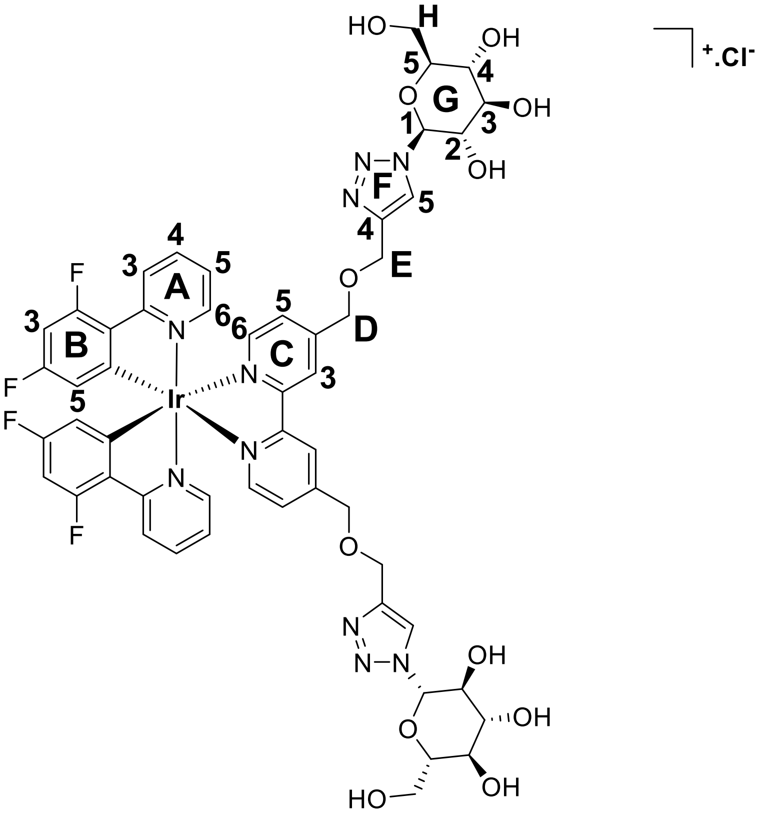

To a solution of **Ir^{Glu-Ac}** (**16**) (82 mg, 50 μmol, 1.0 equiv.) in dry MeOH (5 ml), a catalytic amount of NaOMe (220 μL, 0.44 equiv., 0.1 M in dry MeOH) was added until the pH was between 9-10. The bright yellow solution stirred at r.t. for 3 h, turning golden yellow overtime. The contents were then neutralized using DOWEX 50WX8 H^+ strong cation exchange resin and then filtered to remove the solid polymer resin. After removing the solvent under low pressure, the contents were dried under high vacuum and left without further purification to give a golden yellow powdery solid.

Yield: 39 mg, 30 μmol, 60% (golden yellow powder).

^{1}H NMR (500 MHz, CD_3OD, 298K) δ_H: 8.74 (s, H^{C3}, 2H) 8.35 – 8.31 (m, $H^{F5} + H^{A6}$, 4H), 7.94 – 7.93 (m, $H^{C6} + H^{A5}$, 4H), 7.69 (d, $^3J_{HH}$ = 5.0 Hz, H^{A3}, 2H), 7.58 (d, $^3J_{HH}$ = 5.3 Hz, H^{C5}, 2H), 7.15 (t, $^3J_{HH}$ = 6.3 Hz, H^{A4}, 2H), 6.69 – 6.66 (ddd, $^3J_{H\text{-}F}$ = 9.8 Hz, 9.1 Hz, $^4J_{HH}$ = 2.2 Hz, H^{B3}, 2H), 5.71 – 5.67 (dd, $^3J_{HF}$ = 8.4 Hz, $^4J_{HH}$ = 2.1 Hz, H^{B5}, 2H), 5.64 – 5.61 (m, H^{G1}, 2H), 4.83 (s, H^E, 4H), 4.80 (s, H^D, 4H), 3.93 – 3.84 (m, $H^{G2} + H^{Ha}$, 4H), 3.73 – 3.68 (m, H^{Hb}, 2H), 3.60 – 3.55 (m, $H^{G3} + H^{G5} + H^{G4}$, 6H).

$^{13}C\{^{1}H\}$ NMR (125 MHz, CD_3OD, 298K) δ_C: 167.7 - 166.5 (dd, J_{CF} = 21 Hz, 13.0 Hz, C^{B4}), 164.2 (d, J_{CF} = 7.0 Hz, C^{A2}), 163.4 - 162.2 (dd, J_{CF} = 23 Hz, 13.0 Hz, C^{B2}), 154.8 (C^{C2}), 153.1 (J_{CF} = 7.0 Hz, C^{B1}), 151.4 (C^{A6}), 150.4 (C^{A3}), 143.5 (C^{F4}), 141.3 (C^{A5}), 127.2 (C^{C5}), 126.9 (C^{C4}), 125.2 (C^{A6}), 125.1 (C^{A4}), 124.5 (C^{B6}), 124.3 (C^{C3}), 123.4 (C^{F5}), 113.6 (d, J_{CF} = 18.0 Hz, C^{B5}), 98.9 (t, J_{CF} = 27.0 Hz, C^{B3}), 88.1 (C^{G1}), 69.6 (E), 63.5 (D), 72.6 (C^{G2}), 61.1 (C^{H}), 79.8 (C^{G5}), 76.7 (C^{G3}), 69.4 (C^{G4}).

$^{19}F\{^{1}H\}$ NMR (400 MHz, CD_3OD, 298K) δ_F: (-108.6) – (-108.12) (m, 2F), (-110.47) – (-110.51) (m, 2F).

Emission (H_2O, λ_{max}(nm), λ_{exc} 290 nm) 550.

ESI-HRMS (+) (*m/z*): 1275.3174 calculated for $C_{68}H_{66}F_4IrN_{10}O_{20}$ $[M]^+$, 1275.3174 found for $[M]^+$.

IR (Solid/ cm^{-1}): $\tilde{\upsilon}$ 3283 (m)(br), 3212 (m), 3063 (m), 2942 (w), 2863 (w), 1643 (m), 1601 (s), 1583 (s), 1471 (s), 1400 (m), 1231 (m), 1151 (m), 1101 (m), 961 (s).

Elemental analysis. Calcd for $C_{52}H_{50}ClF_4IrN_{10}O_{12}.2H_2O$: C 46.38, H 4.04, N 10.40. **Found**: C 46.57, H 3.93, N 10.52 %.

Ir^{Gal} (19)

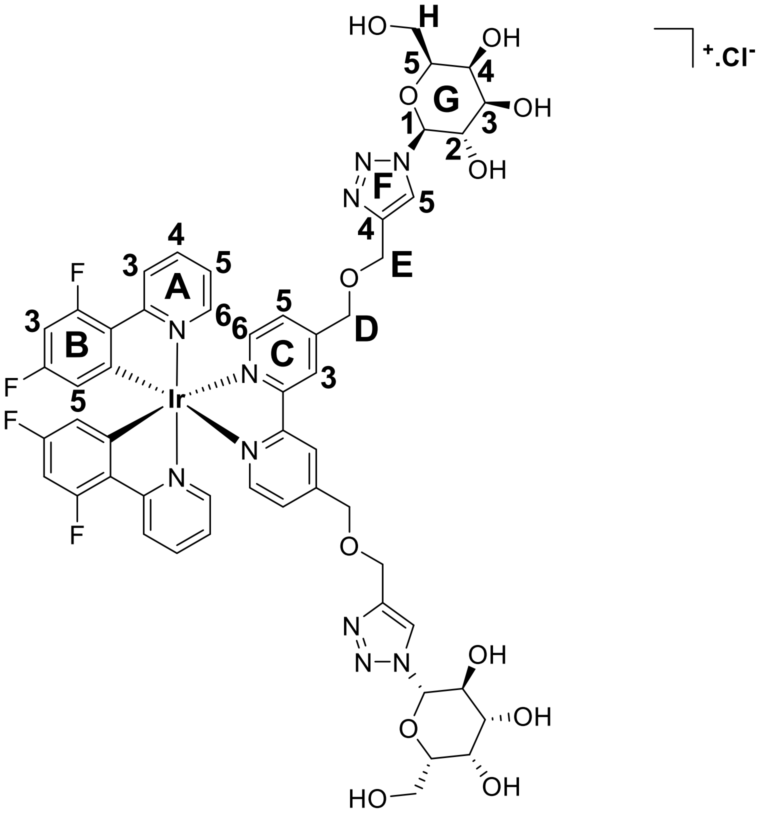

This complex was synthesised using an identical method to **Ir^{Glu}** (**18**), substituting **Ir$^{Glu\text{-}Ac}$** (**16**) for **Ir$^{Gal\text{-}Ac}$** (**17**) (102 mg, 62 μmol, 1.0 equiv.).

Yield: 53.5 mg, 40 μmol, 66% (golden yellow powder).

^{1}H NMR (500 MHz, CD_3OD, 298K) δ_H: 8.73 (s, H^{C3}, 2H), 8.38 - 8.32 (m, H^{F5} + H^{A6}, 4H), 7.97 – 7.93 (m, H^{C6} + H^{A5}, 4H), 7.68 (d, $^{3}J_{HH}$ = 5.0 Hz, H^{A3}, 2H), 7.58 (d, $^{3}J_{HH}$ = 5.7 Hz, H^{C5}, 2H), 7.15 (t(br), H^{A4}, 2H), 6.69 – 6.67 (ddd, $^{3}J_{HF}$ = 12.2 Hz, 9.3 Hz, $^{4}J_{HH}$ = 2.3 Hz, H^{B3}, 2H), 5.71 (dd, $^{3}J_{HF}$ = 8.4 Hz, $^{4}J_{HH}$ = 2.3 Hz, H^{B5}, 2H), 5.59 (d, $^{3}J_{HH}$ = 9.2 Hz, H^{G1}, 2H), 4.81 (s, H^{E}, 4H), 4.77 (s, H^{D}, 4H), 4.15 (t, $^{3}J_{HH}$ = 9.3 Hz, H^{G2}, 2H), 3.99 (d, $^{3}J_{HH}$ = H^{G4}, 2H), 3.83 (t, $^{3}J_{HH}$ = 5.0 Hz, H^{G5}, 2H), 3.74 – 3.69 (m, H^{H} + H^{G3}, 6H).

^{13}C{^{1}H} NMR (125 MHz, CD_3OD, 298K) δ_C: 164.85 – 164.79 (d, J_{CF} = 12.3 Hz, C^{B4}), 163.98 – 163.93 (d, J_{CF} = 12.3 Hz, C^{A2}), 162.8 – 162.5 (dd, J_{CF} = 23 Hz, 13.0 Hz, C^{B2}), 160.5 (d, J_{CF} = 7.0 Hz, C^{B1}), 155.6 (C^{C2}), 151.5 (C^{C6}), 150.4 (C^{A3}), 144.3 (C^{F4}), 140.3 (C^{A5}), 127.7 (C^{A6}), 127.3 (C^{C5}), 126.3 (C^{C4}), 124.9 (C^{A4}), 124.8 (C^{B6}), 123.5 (C^{C3}), 122.4 (C^{F5}), 113.5 (d, J_{CF} = 18.0 Hz, C^{B5}), 99.4 (t, J_{CF} = 27.0 Hz, C^{B3}), 88.8 (C^{G1}), 78.5 (C^{G3}), 70.1 (C^{G4}), 69.9 (C^{G5}), 69.3 (C^{E}), 63.5 (C^{D}), 61.0 (H^{H}), 55.1 (C^{G2}).

$^{19}F\{^1H\}$ NMR (400 MHz, CD_3OD, 298K) δ_F: (-108.6) – (-108.12) (m, 2F), (-110.47) – (-110.51) (m, 2F).

Emission [H_2O, λ_{max}(nm), λ_{exc} 290 nm] 550.

ESI-HRMS (+) (*m/z*): 1275.3173 calculated for $C_{52}H_{50}F_4IrN_{10}O_{12}^+$ $[M]^+$, found 1275.3179 for $[M]^+$.

IR (Solid/ cm^{-1}): $\tilde{\upsilon}$ 3294 (m)(br), 3201 (m), 2975 (w), 2812 (w), 1632 (m), 1602 (s), 1582 (s), 1472 (s), 1423 (m), 1231 (m), 1123 (m), 1075 (m), 990 (s).

Elemental analysis. Calcd for $C_{52}H_{50}ClF_4IrN_{10}O_{12}.3H_2O$: C 45.77, H 4.14, N 10.26. **Found**: C 45.49, H 4.21, N 10.12%.

Ru$^{Glu\text{-}Ac}$ (20)

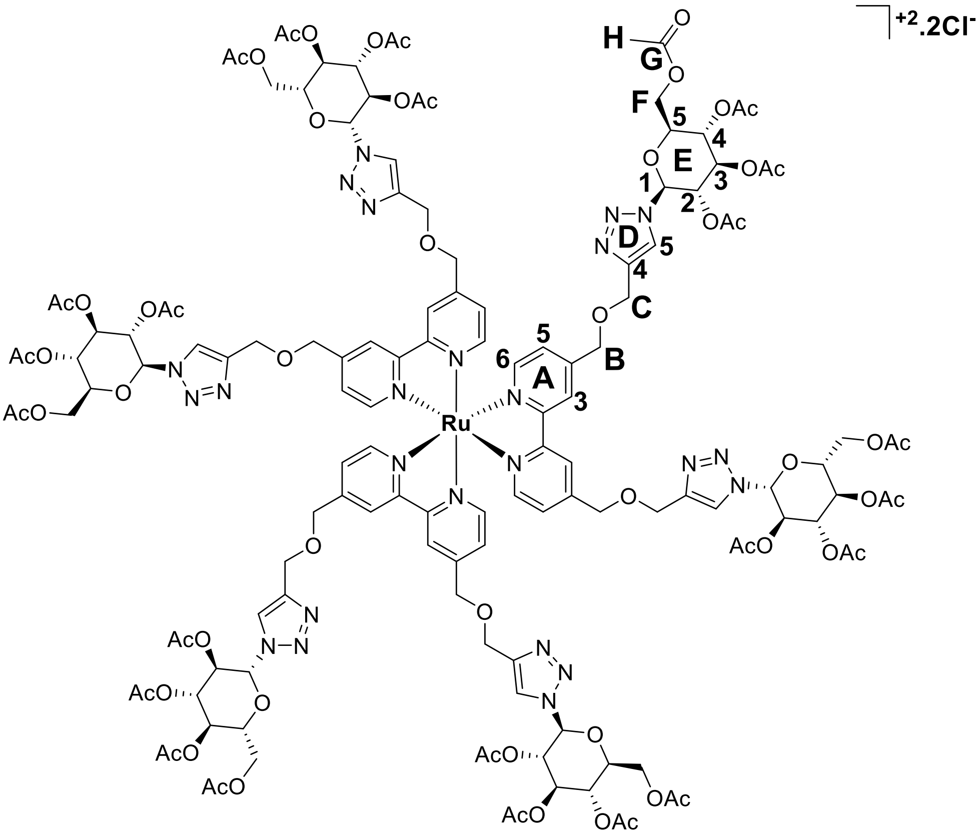

To a degassed solution of Ru(DMSO)$_4$Cl$_2$ (50 mg, 90 µmol, 1.0 equiv.) in EtOH (10 ml) was added **Bipy$^{Glu\text{-}Ac}$** (**9**) (299 mg, 0.29 mmol, 3.1 equiv.) and the orange suspension was refluxed under a N_2 atmosphere for 48 h in the dark. Once cooled, the solvent was removed under low pressure and a dark orange solid was obtained.

This solid was purified by size exclusion chromatography using Sephadex LH-20 (~ 1m column), eluting with CH_2Cl_2/MeOH (50:50, v/v) and dried under high vacuum for 4 h. The product was left as a mixture of its stereoisomers (Δ/Λ).

Yield: 229 mg, 70 µmol, 75% (dark red powder).

^{1}H NMR (500 MHz, CD$_3$OD, 298K) δ_H: 8.67 (s, H^{A3}, 6H), 8.37 (s, H^{D5}, 6H), 7.72 (d, $^3J_{HH}$ = 4.7 Hz, H^{A6}, 6H), 7.42 (t, $^3J_{HH}$ = 4.9 Hz, H^{A5}, 6H), 6.19 (dd, $^3J_{HH}$ = 9.2 Hz, $^4J_{HH}$ = 1.9 Hz, H^{E1}, 6H), 5.65 – 5.60 (td, $^3J_{HH}$ = 9.5 Hz, $^4J_{HH}$ = 3.8 Hz, H^{E2}, 6H), 5.56 (t, $^3J_{HH}$ = 9.2 Hz, H^{E3}, 6H), 5.28 (t, $^3J_{HH}$ = 9.3 Hz, H^{E4}, 6H), 4.79 (s, H^C + H^B, 24H), 4.29 – 4.26 (m, H^{E5} + H^{Fa}, 12H), 4.15 (d, $^3J_{HH}$ = 10.6 Hz, H^{Fb}, 6H), 2.07, 1.99, 1.78 (s, H^H, 72H).

^{13}C{^{1}H} NMR (125 MHz, CD$_3$OD, 298K) δ_C: 171.6, 170.4, 169.8, 169.2 (C^H), 158.2 (C^{A2}), 152.1 (C^{A6}), 144.7 (C^{D4}), 126.2 (C^{A5}), 125.7 (C^{A4}), 124.6 (C^{D5}), 123.3 (C^{A3}), 86.3 (C^{E1}), 75.8

(C^{E5}), 73.9 (C^{E3}), 71.8 (C^{E2}), 70.7 (C^{B}), 69.1 (C^{E4}), 64.5 (C^{C}), 62.8 (C^{F}), 19.21, 19.2, 19.1, 18.9 (C^{G}).

ESI-HRMS (+) (*m/z*): 1608.9631 calculated for $C_{138}H_{162}N_{24}O_{60}Ru$ $[M]^{2+}$, 1608.9648 found for $[M]^{2+}$.

IR (Solid/ cm^{-1}): $\tilde{\upsilon}$ 2963 (w), 2921 (w), 2861 (w), 1744 (m), 1598 (w), 1368 (s), 1211 (s), 1088 (s), 1041 (s), 922 (m).

Elemental analysis. Calcd for $C_{138}H_{162}Cl_2N_{24}O_{60}Ru.3H_2O$: C 49.58, H 5.07, N 10.06. **Found**: C 49.31, H 4.92, N 10.13%.

Ru$^{Gal\text{-}Ac}$ (21)

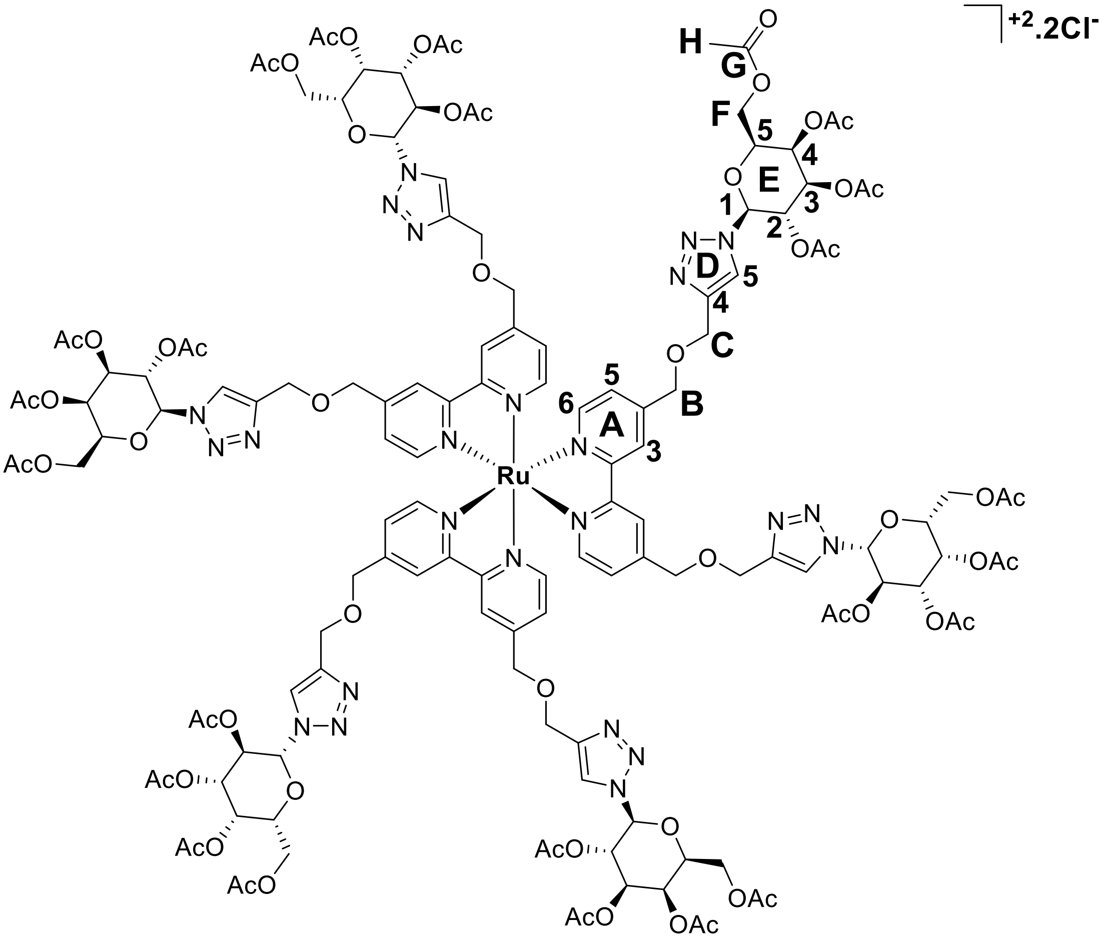

This complex was synthesised using an identical method to **Ru$^{Glu\text{-}Ac}$** (**21**), substituting **Bipy$^{Glu\text{-}Ac}$** (**9**) for **Bipy$^{Gal\text{-}Ac}$** (**13**) (332 mg, 0.32 mmol, 3.1 equiv.)

Yield: 272 mg, 83 µmol, 80% (dark red powder).

^{1}H NMR (500 MHz, CD_3OD, 298K) δ_H: 8.73 (d, $^{3}J_{HH}$ = 5.6 Hz, H^{A3}, 6H), 8.33 (s, H^{D5}, 6H), 7.74 (t, $^{3}J_{HH}$ = 5.4 Hz, H^{A6}, 6H), 7.46 (d, $^{3}J_{HH}$ = 5.2 Hz, H^{A5}, 6H), 6.13 (dd, $^{3}J_{HH}$ = 9.2 Hz, $^{4}J_{HH}$ = 2.7 Hz, H^{E1}, 6H), 5.64 (t, $^{3}J_{HH}$ = 9.7 Hz, H^{E2}, 6H), 5.56 (s, H^{E4}, 6H), 5.45 – 5.40 (dd, $^{3}J_{HH}$ = 10 Hz, $^{4}J_{HH}$ = 3.1 Hz, H^{E3}, 6H), 4.80 (s, H^{C} + H^{B}, 24H), 4.50 (q, $^{3}J_{HH}$ = 6.3 Hz, H^{E5}, 6H), 4.22 – 4.09 (m, H^{F}, 12H), 2.19, 2.00, 1.97, 1.81 (s, H^{H}, 72H).

^{13}C{^{1}H} NMR (125 MHz, CD_3OD, 298K) δ_C: 170.6, 170.4, 169.8, 169.3 (C^{G}), 156.2 (C^{A2}), 152.3 (C^{A6}), 144.5 (C^{D4}), 126.4 (C^{A5}), 125.3 (C^{A4}), 124.5 (C^{D5}), 123.4 (C^{A3}), 85.7 (C^{E1}), 73.6 (C^{E5}), 70.8 (C^{E3}), 69.2 (C^{C}), 68.3 (C^{E2}), 67.2 (C^{E4}), 63.2 (C^{B}), 61.1 (C^{F}), 19.2, 19.20, 19.1, 18.9 (C^{H}).

ESI-HRMS (+) (*m/z*): 1608.9725 calculated for $C_{138}H_{162}N_{24}O_{60}Ru$ $[M]^{2+}$, 1608.9732 found for $[M]^{2+}$.

IR (Solid/ cm^{-1}): $\tilde{\upsilon}$ 2963 (w), 2921 (w), 2861 (w), 1744 (m), 1598 (w), 1368 (s), 1211 (s), 1088 (s), 1041 (s), 922 (m).

Elemental analysis. Calcd for $C_{138}H_{162}Cl_2N_{24}O_{60}Ru.3H_2O$: 49.85, H 5.03, N 10.11. **Found**: C 49.70, H 4.85, N 10.20%.

Ru^{Glu} (22)

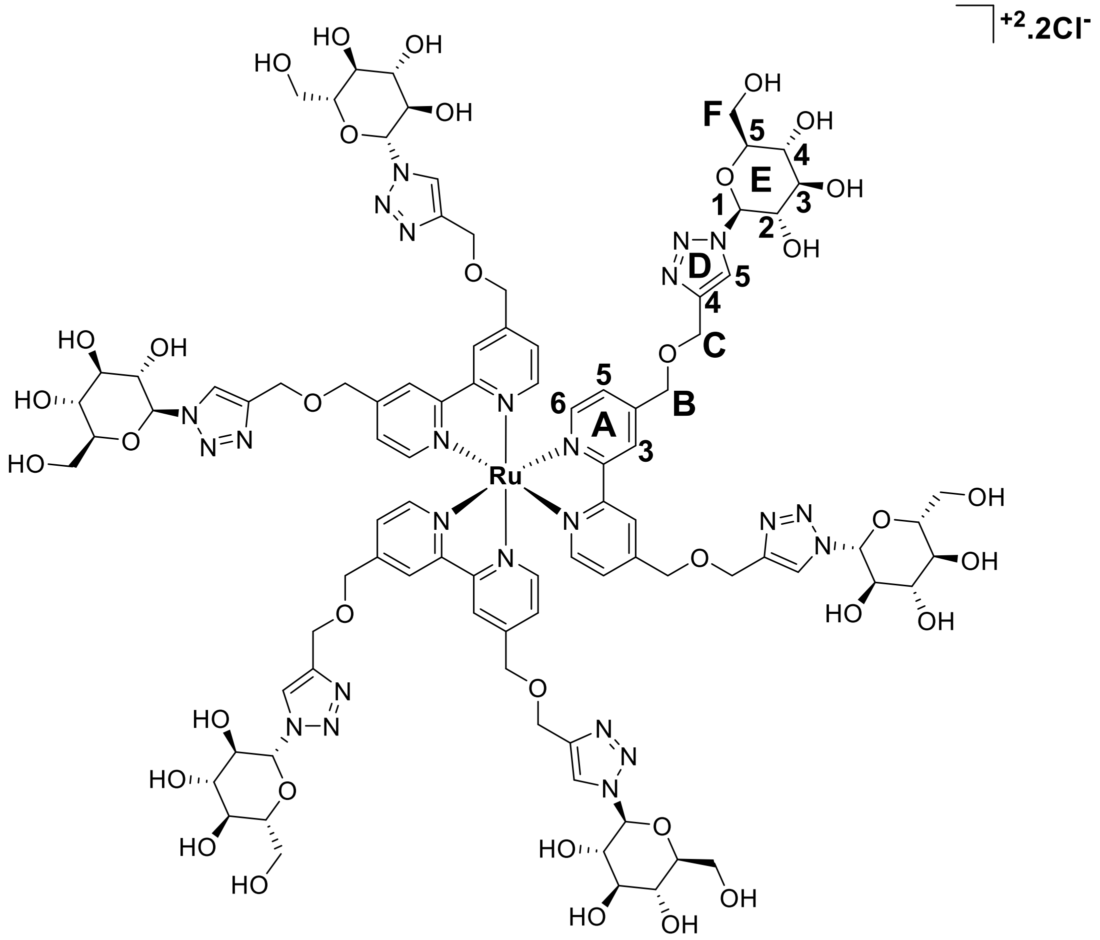

Ru^{Glu-Ac} (**20**) (80 mg, 24 µmol, 1.0 equiv.) was carefully added to a solution of MeOH (5 ml), creating a dark orange solution. A mixture of MeOH/H_2O/TEA (5 ml, 4:2:1, v/v) was added and the solution was heated to 50 ˚C for 18 h under N_2. Once cooled to r.t., the resulting dark red solution was evaporated to dryness and slowly purified on Toyo-Pearl HW40-S gel using a 0.01 M ammonium acetate eluent, eluting as a bright orange band, followed by a purple by-product.

Fractions were collected based on phosphorescence under a TLC lamp (observed on an aluminium backed silica gel 60 matrix plate) and were combined and evaporated to give a shiny red solid. This product was then carefully dissolved in the minimum amount of H_2O and titrated with IPA to give a bright orange precipitate. Centrifugation and evaporation of the solvent under high vacuum at 70 ˚C gave a powdery red solid.

Yield: 23 mg, 10 µmol, 42% (dark red powder).

^{1}H NMR (500 MHz, D_2O, 298K) δ_H: 8.49 (s, H^{A3}, 6H), 8.31 (s, H^{D5}, 6H), 7.67 – 7.65 (m, H^{A6}, 6H), 7.29 (d, $^3J_{HH}$ = 5.64 Hz, H^{A5}, 6H), 5.69 (t, $^3J_{HH}$ = 9.24 Hz, H^{E1}, 6H), 4.82 (s, H^C, 12H),

4.81 (s, H^B, 12H), 3.99 – 3.93 (td, $^3J_{HH}$ = 9.3 Hz, $^4J_{HH}$ = 2.7 Hz, H^{E2}, 6H), 3.85 – 3.78 (m, H^{Fa}, 6H), 3.75 – 3.66 (m, H^{E3} + H^{E4} + H^{Fb}, 18H), 3.62 – 3.57 (m, H^{E5}, 6H).

$^{13}C\{^1H\}$ NMR (125 MHz, D_2O, 298K) δ_C: 156.5 (C^{A2}), 150.9 (C^{A6}), 143.9 (C^{D1}), 125.5 (C^{A5}), 125.3 (C^{A4}), 124.2 (C^{D5}), 122.4 (C^{A3}), 87.4 (C^{E1}), 78.9 (C^{E4}) 76.0 (C^{E3}), 72.1 (C^{E2}), 69.8 (C^C), 68.9 (C^{E5}), 63.0 (C^B), 60.5 (C^F).

Emission [H_2O, λ_{max}(nm), λ_{exc} 290 nm] 632.

ESI-HRMS (+) (*m/z*): 1104.3443 calculated for $C_{90}H_{114}N_{24}O_{36}Ru$ $[M]^{2+}$, 1104.3432 found for $[M]^{2+}$.

IR (Solid/ cm^{-1}): $\tilde{\upsilon}$ 3288 (m), 3140 (m), 2870 (w), 1618 (m), 1561 (m), 1416 (m), 1368 (m), 1233 (m), 1091 (s), 1053 (s), 889 (m), 606 (m).

Elemental analysis. Calcd for $C_{90}H_{114}Cl_2N_{24}O_{36}Ru.6H_2O$: 45.27, H 5.32, N 14.08. **Found**: C 45.34, H 5.12, N 14.39%.

Ru^Gal (23)

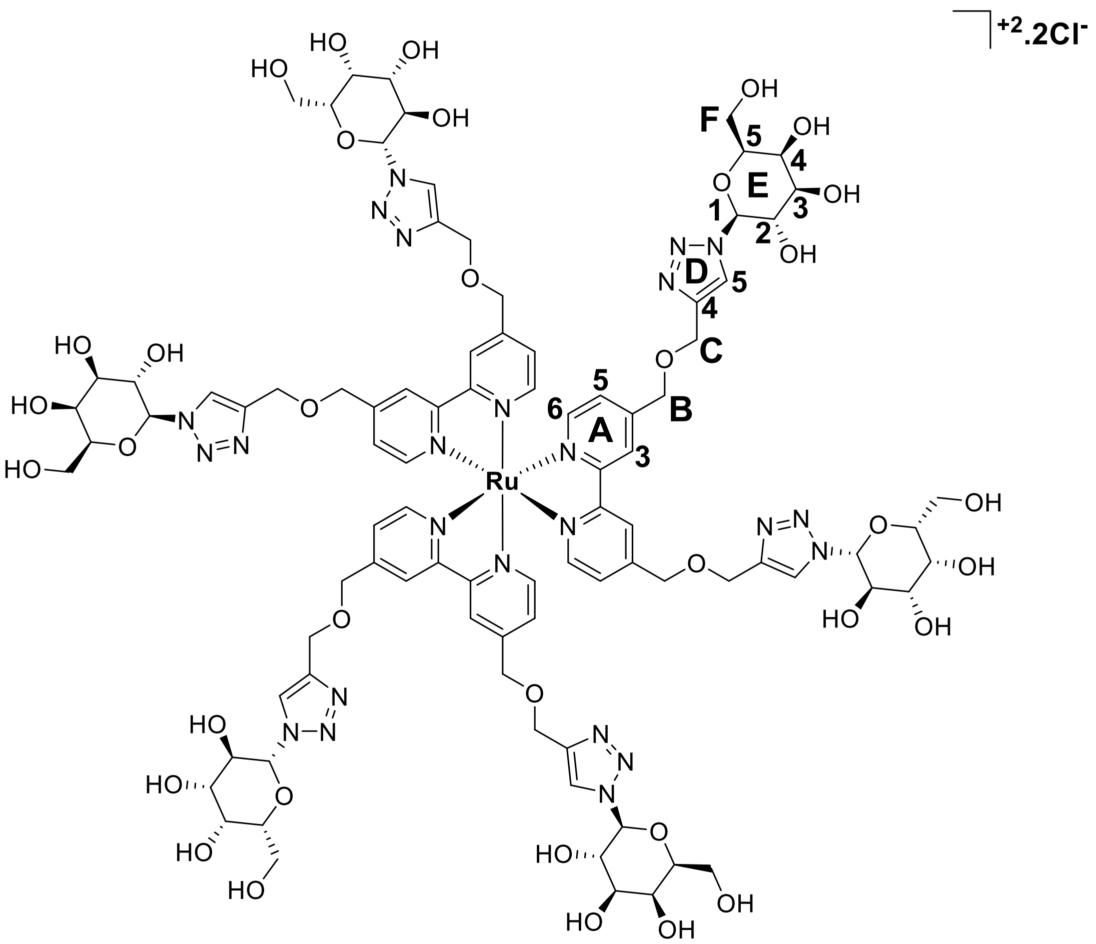

Ru^Gal (**23**) was synthesised using an identical method to **Ru^Glu** (**22**), substituting **Ru^Glu-Ac** (**20**) for **Ru^Gal-Ac** (**21**) (90 mg, 27 µmol, 3.1 equiv.).

Yield: 30 mg, 14 µmol, 50% (dark red powder).

^{1}H NMR (500 MHz, D_2O, 298K) δ_H: 8.48 (s, H^{A3}, 6H), 8.33 (s, H^{D5}, 6H), 7.69 – 7.61 (m, H^{A6}, 6H), 7.34 – 7.28 (m, H^{A5}, 6H), 5.66 – 5.62 (dd, $^3J_{HH}$ = 9.2 Hz, $^4J_{HH}$ = 3.9 Hz, H^{E1}, 6H), 4.82 (s, $H^C + H^B$, 24H), 4.21 – 4.16 (td, $^3J_{HH}$ = 9.5 Hz, $^4J_{HH}$ = 3.4 Hz, H^{E2}, 6H), 4.07 – 4.05 (m, H^{E4}, 6H), 3.97 – 3.83 (m, $H^{E3} + H^{E5}$, 12H), 3.75 – 3.52 – (m, H^{Fab}, 12H).

^{13}C{^{1}H} NMR (125 MHz, D_2O, 298K) δ_C: 156.6 (C^{A2}), 151.0 (C^{A6}), 143.9 (C^{D1}), 125.6 (C^{A5}), 125.2 (C^{A4}), 124.0 (C^{D5}), 122.2 (C^{A3}), 88.1 (C^{E1}), 78.2 (C^{E5}), 72.9 (C^{E3}), 69.9 (C^B), 69.8 (C^{E2}), 68.4 (C^{E4}), 63.2 (C^C), 60.7 (C^F).

Emission [H_2O, λ_{max}(nm), λ_{exc} 290 nm] 632.

ESI-HRMS (+) (*m/z*): 1104.3443 calculated for $C_{90}H_{114}N_{24}O_{36}Ru$ [*M*]$^{2+}$, 1104.3439 found for [*M*]$^{2+}$.

IR (Solid/ cm^{-1}): $\tilde{\upsilon}$ 3313 (m), 2919 (w), 2875 (w), 1563 (m), 1415 (m), 1386 (m), 1233 (m), 1091 (s), 1053 (s), 889 (m), 826 (m), 648 (m).

Elemental analysis Calcd for $C_{90}H_{114}Cl_2N_{24}O_{36}Ru.7H_2O$: 44.93, H 5.36, N 13.97. **Found**: C 45.12, H 5.20, N 14.05%.

NMR and MS figures

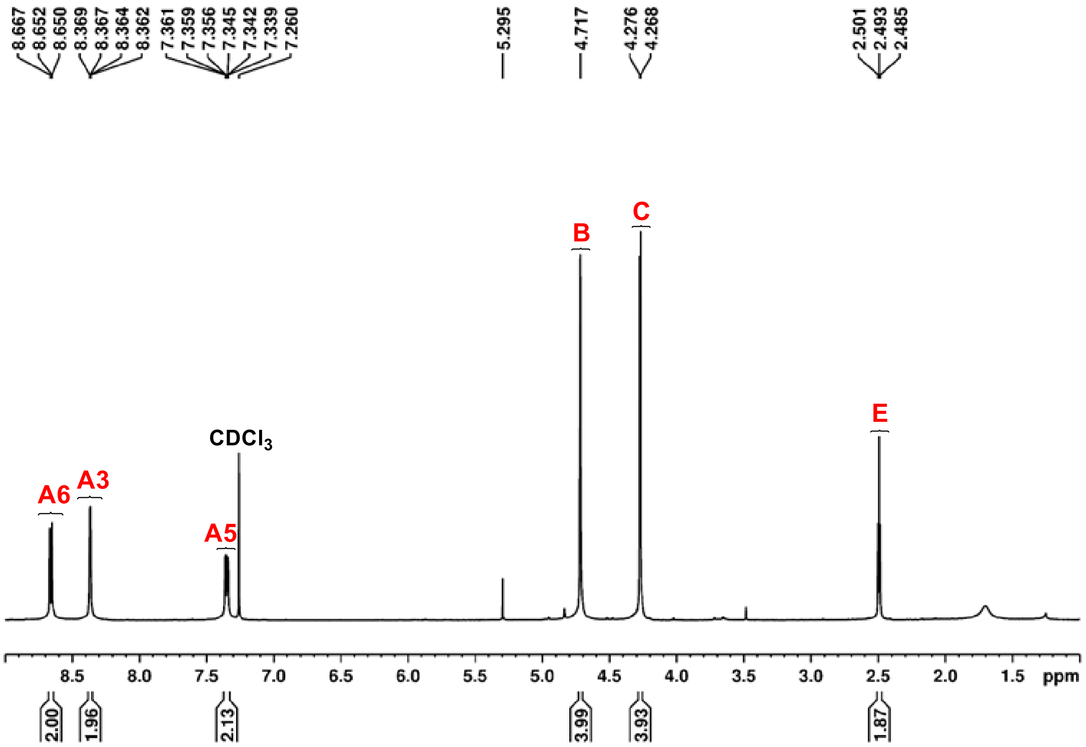

Figure S1 – ^{1}H NMR spectrum (500 MHz, $CDCl_3$, 298K) of compound **5**.

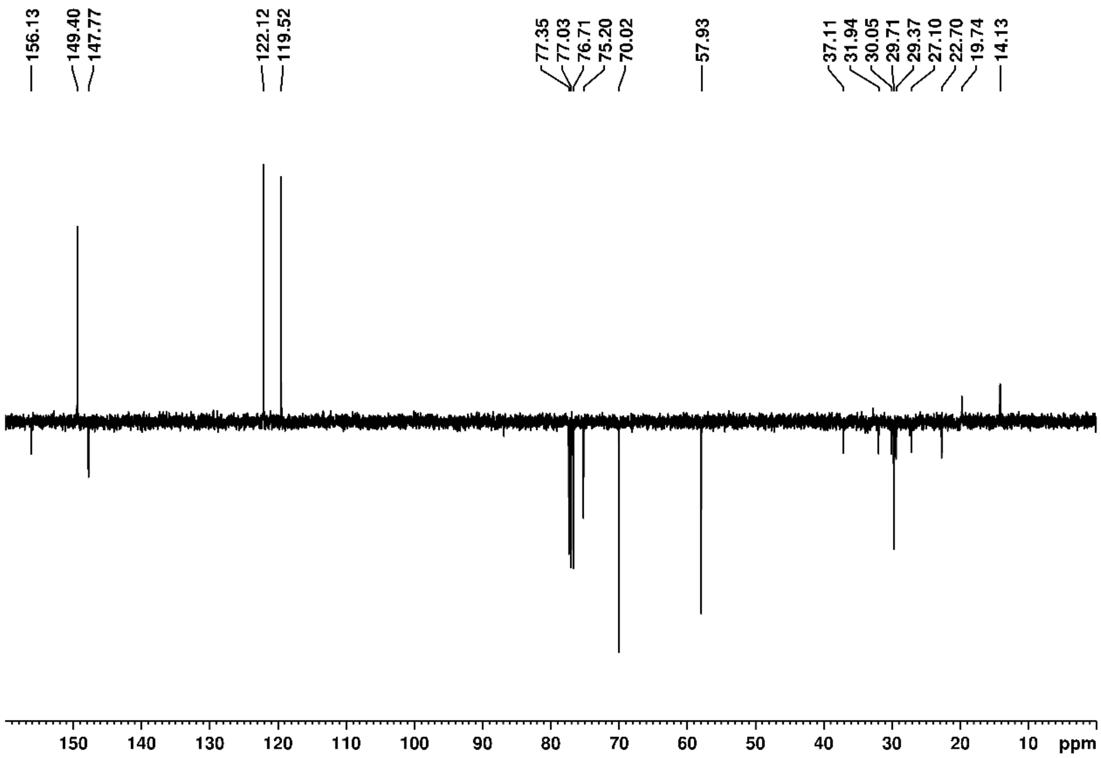

Figure S2 – $^{13}C\{^{1}H\}$ NMR spectrum (125 MHz, $CDCl_3$, 298K) of compound **5**.

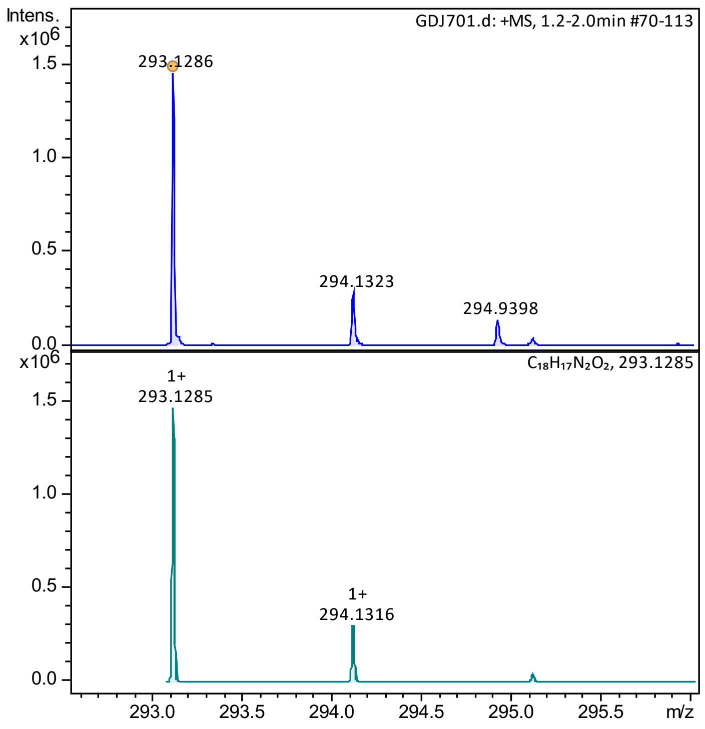

Figure S3 – ESI-HRMS of compound **5** ([*M*+H]$^+$).

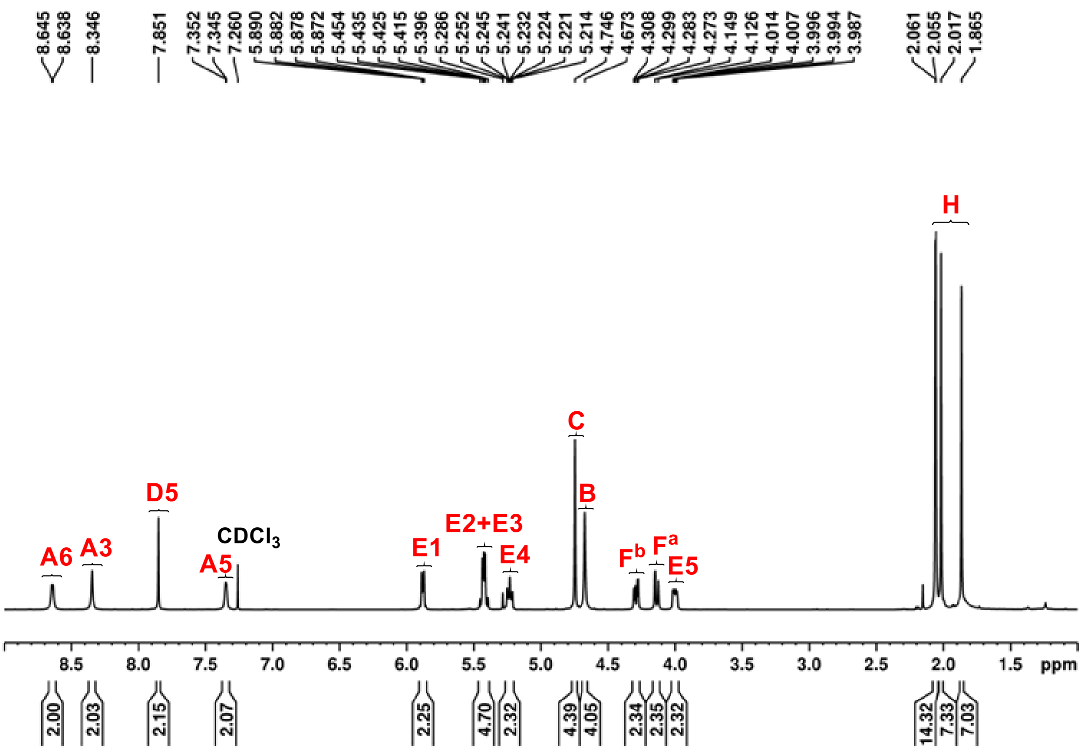

Figure S4 - ^{1}H NMR spectrum (500 MHz, $CDCl_3$, 298K) of **Bipy**$^{Glu\text{-}Ac}$ (**9**).

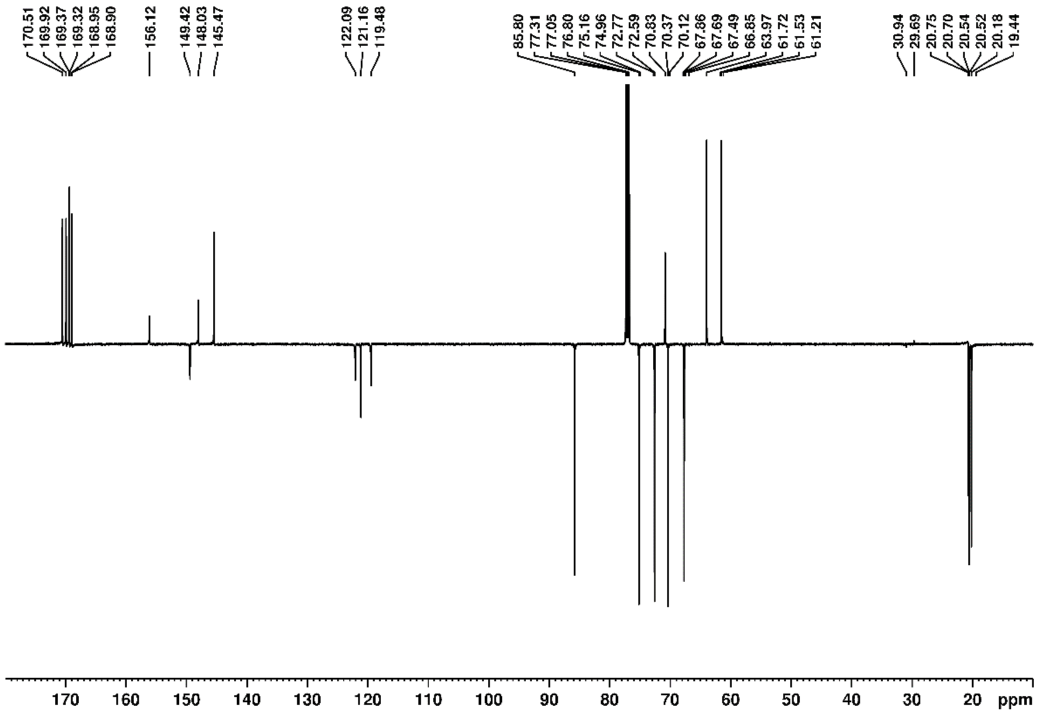

Figure S5 - $^{13}C\{^{1}H\}$ NMR spectrum (125 MHz, $CDCl_3$, 298K) of **Bipy**$^{Glu\text{-}Ac}$ (**9**).

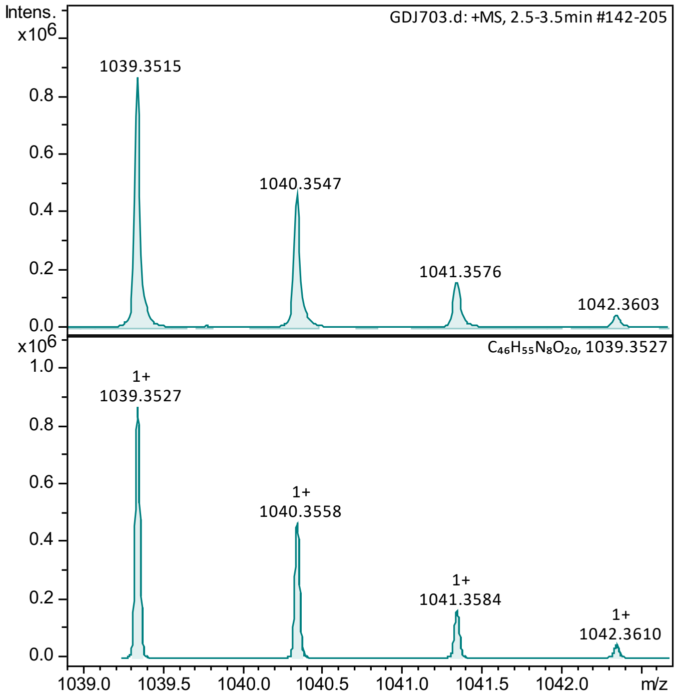

Figure S6 – ESI-HRMS of **Bipy$^{\mathbf{Glu\text{-}Ac}}$** (**9**) ([$M$+H]$^+$).

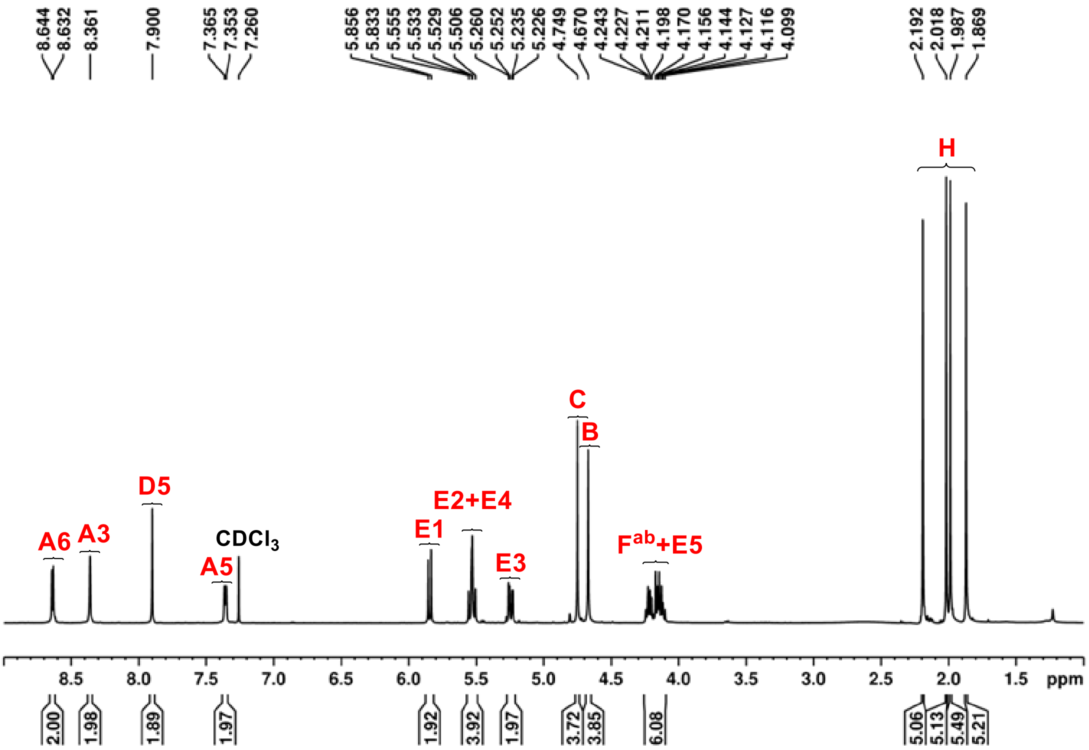

Figure S7 - ^{1}H NMR spectrum (500 MHz, CDCl$_3$, 298K) of **Bipy$^{\mathbf{Gal\text{-}Ac}}$** (**13**).

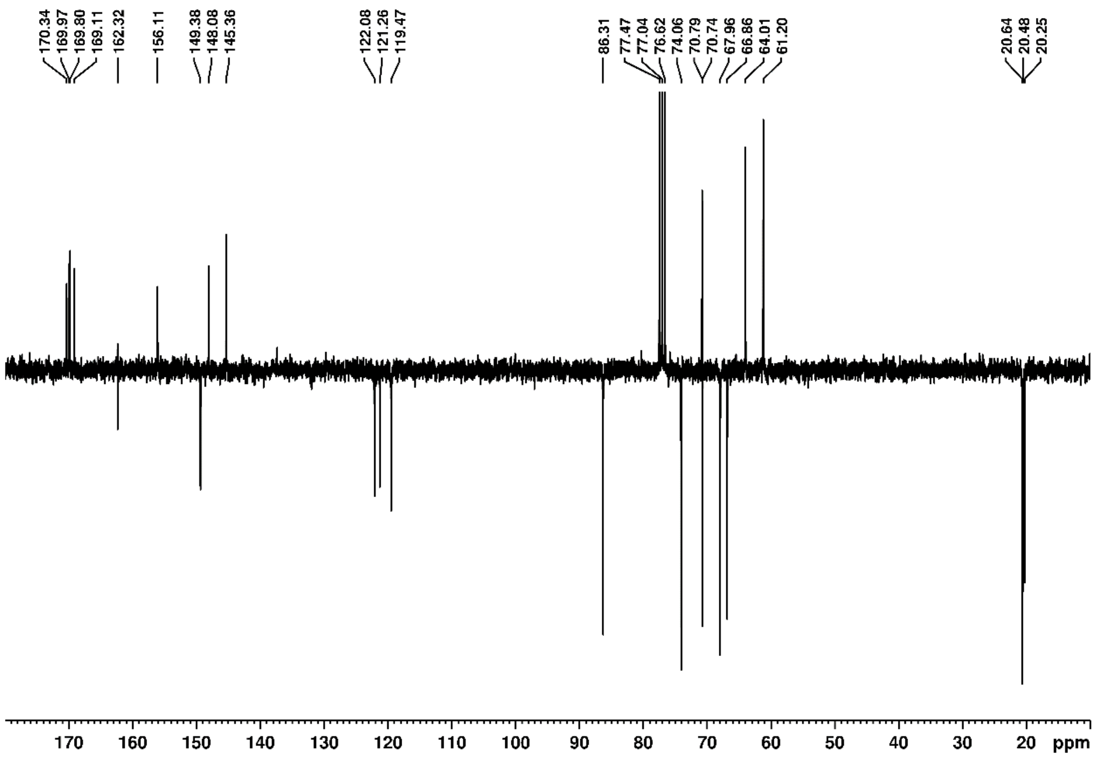

Figure S8 - $^{13}C\{^1H\}$ NMR spectrum (125 MHz, $CDCl_3$, 298K) of **Bipy$^{\text{Gal-Ac}}$** (**13**).

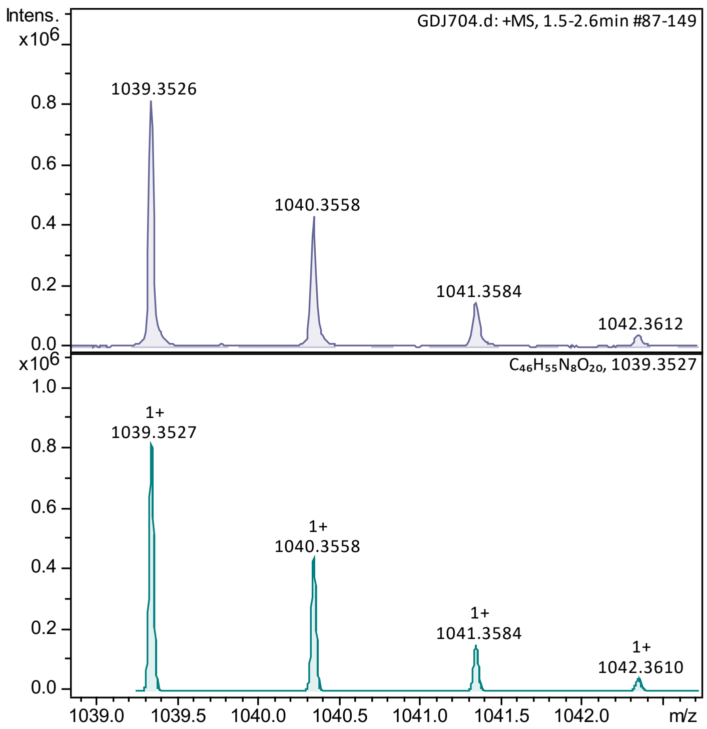

Figure S9 – ESI-HRMS of **Bipy$^{\text{Gal-Ac}}$** (**13**) ($[M+H]^+$).

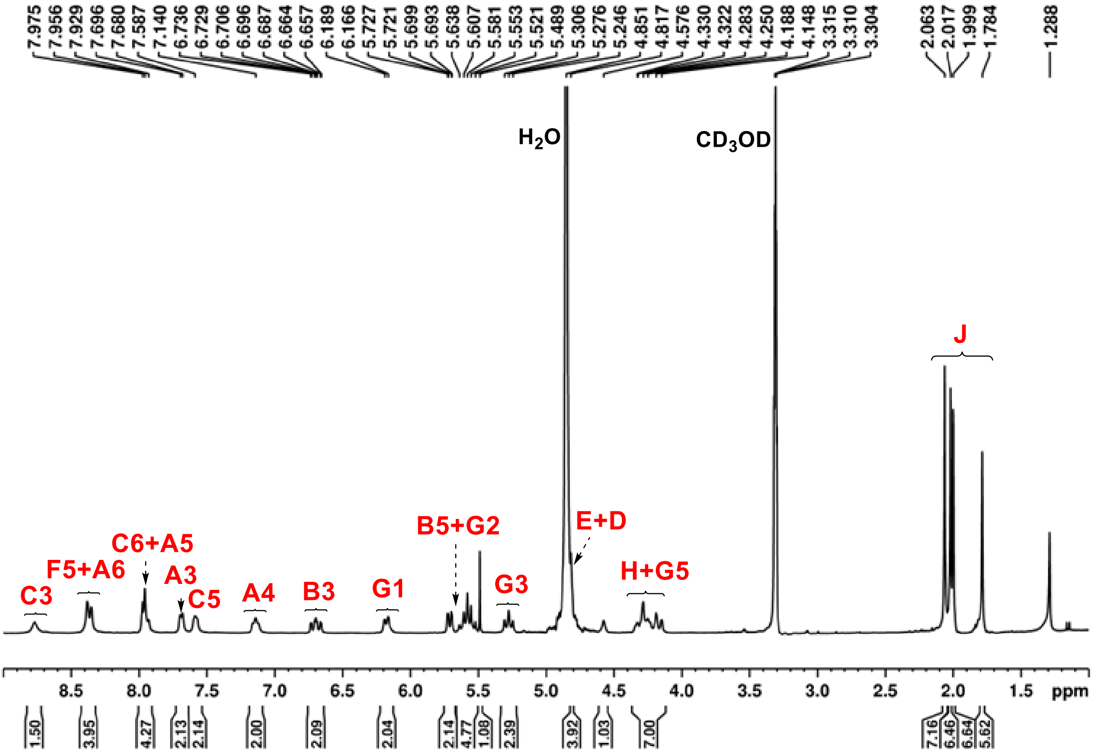

Figure S10 - ^{1}H NMR spectrum (500 MHz, CD3OD, 298K) of **Ir**$^{\mathbf{Glu\text{-}Ac}}$ (**16**).

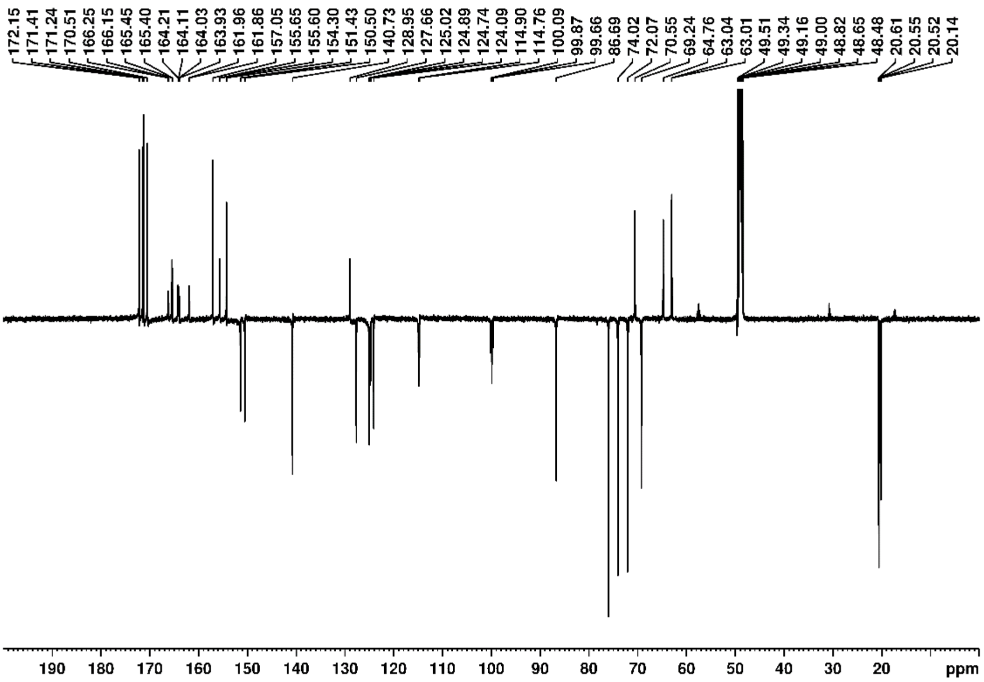

Figure S11 - ^{13}C{^{1}H} NMR spectrum (125 MHz, CD3OD, 298K) of **Ir**$^{\mathbf{Glu\text{-}Ac}}$ (**16**).

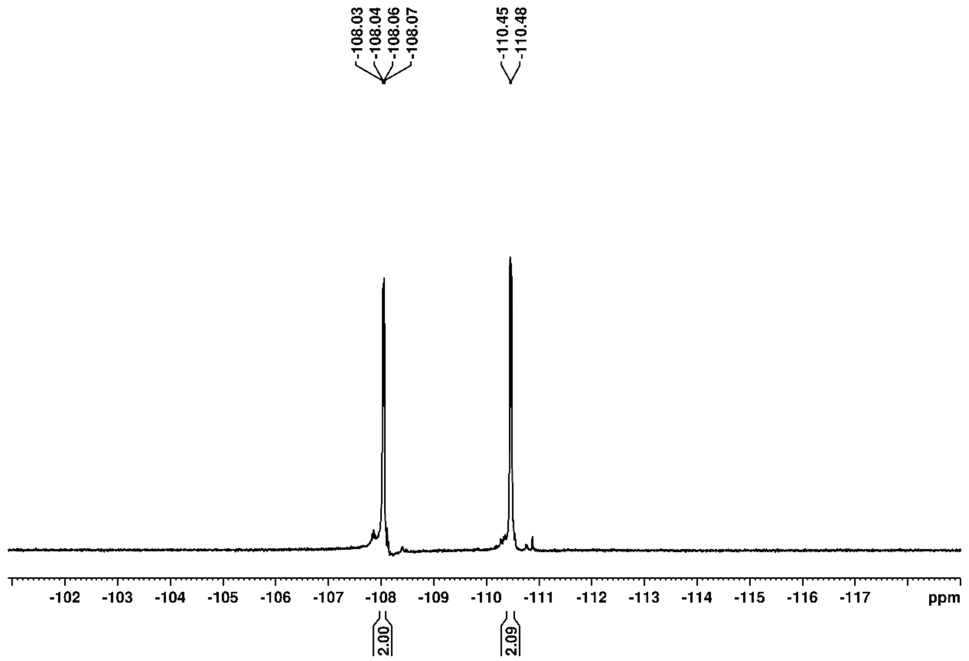

Figure S12 – $^{19}F\{^{1}H\}$ NMR spectrum (400 MHz, CD_3OD, 298K) for **Ir**$^{\mathbf{Glu\text{-}Ac}}$ (**16**).

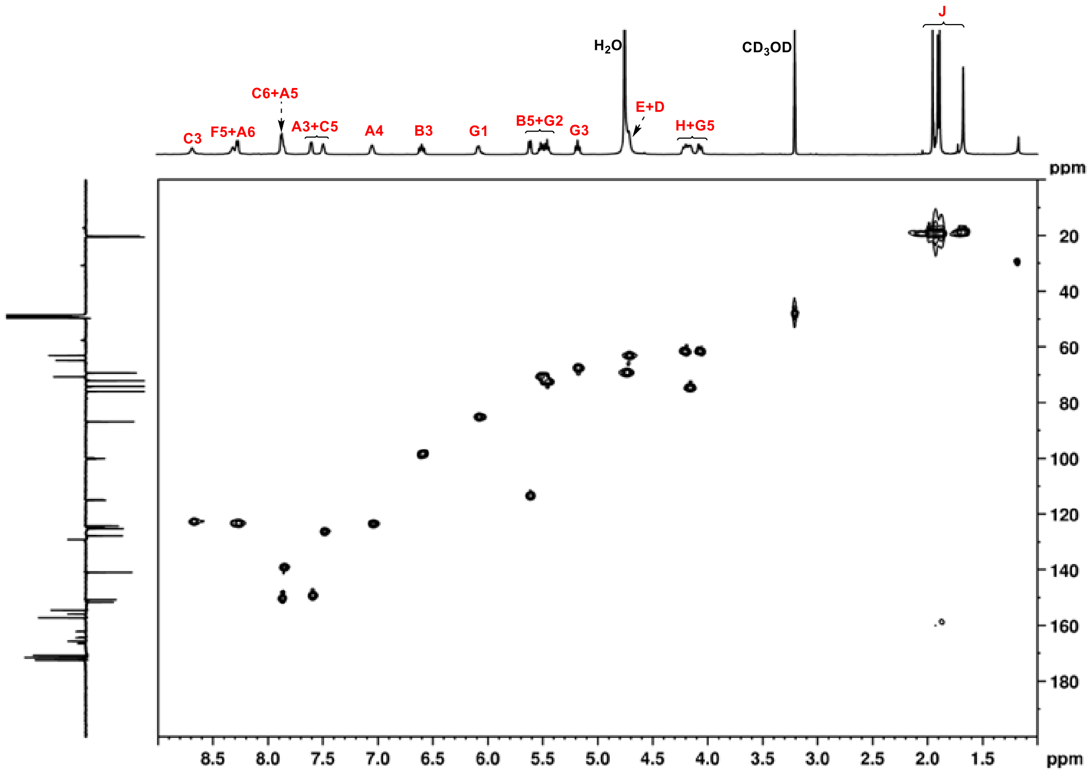

Figure S13 – $^{1}H\text{-}^{13}C\{^{1}H\}$ HSQC NMR spectrum for **Ir**$^{\mathbf{Glu\text{-}Ac}}$ (**16**).

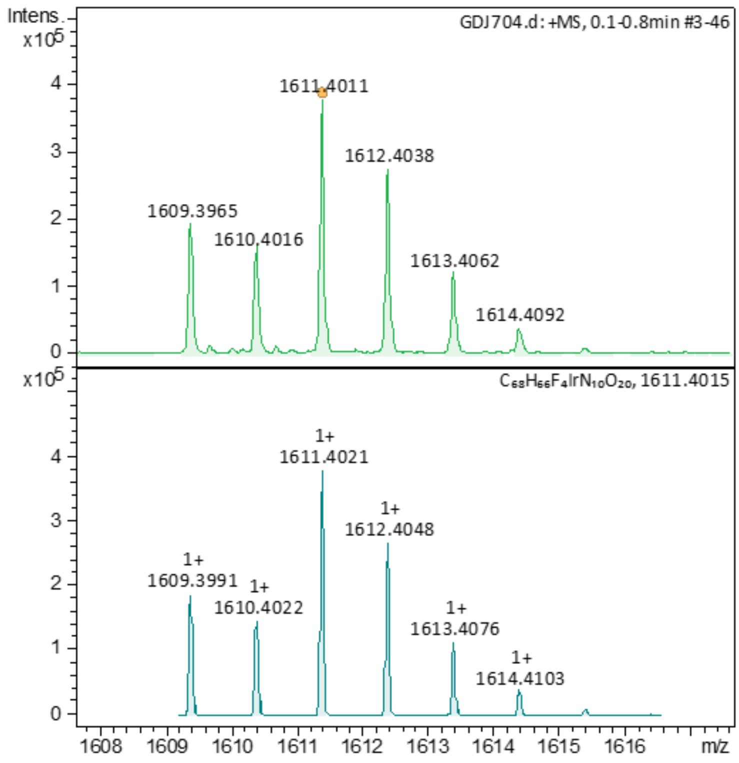

Figure S14 – ESI-HRMS of **Ir**$^{\mathbf{Glu\text{-}Ac}}$ (**16**) ([*M*]$^+$).

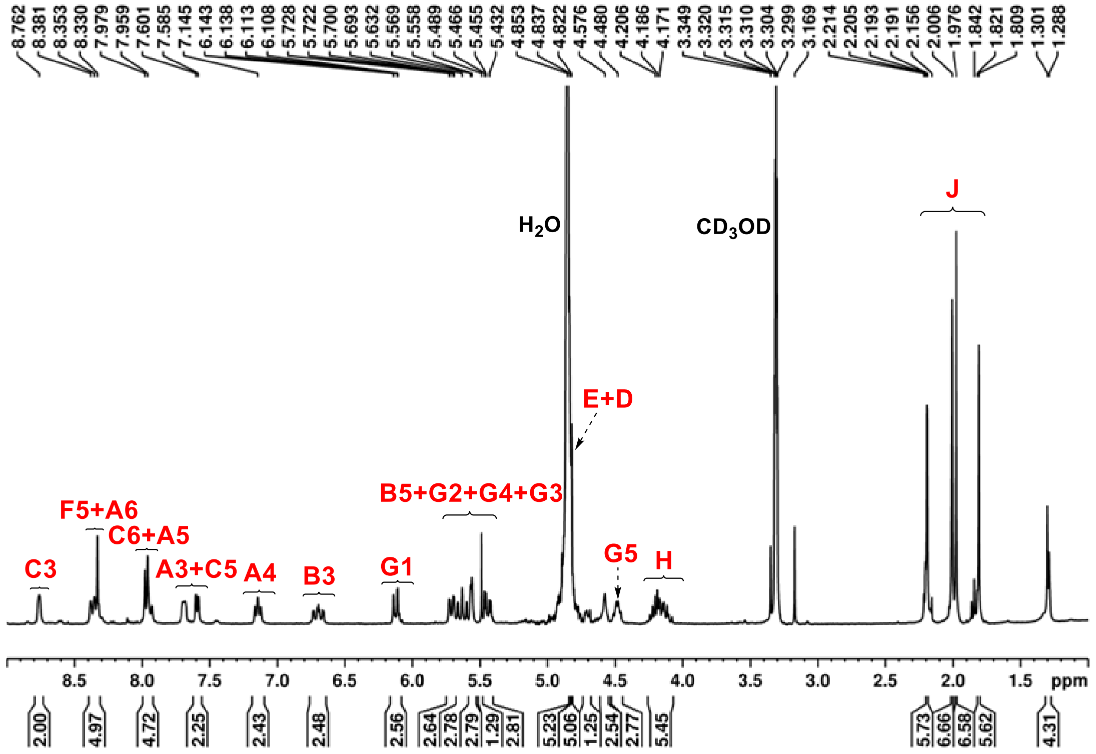

Figure S15 – ^{1}H NMR spectrum (500 MHz, CD$_3$OD, 298K) for **Ir**$^{\mathbf{Gal\text{-}Ac}}$ (**17**).

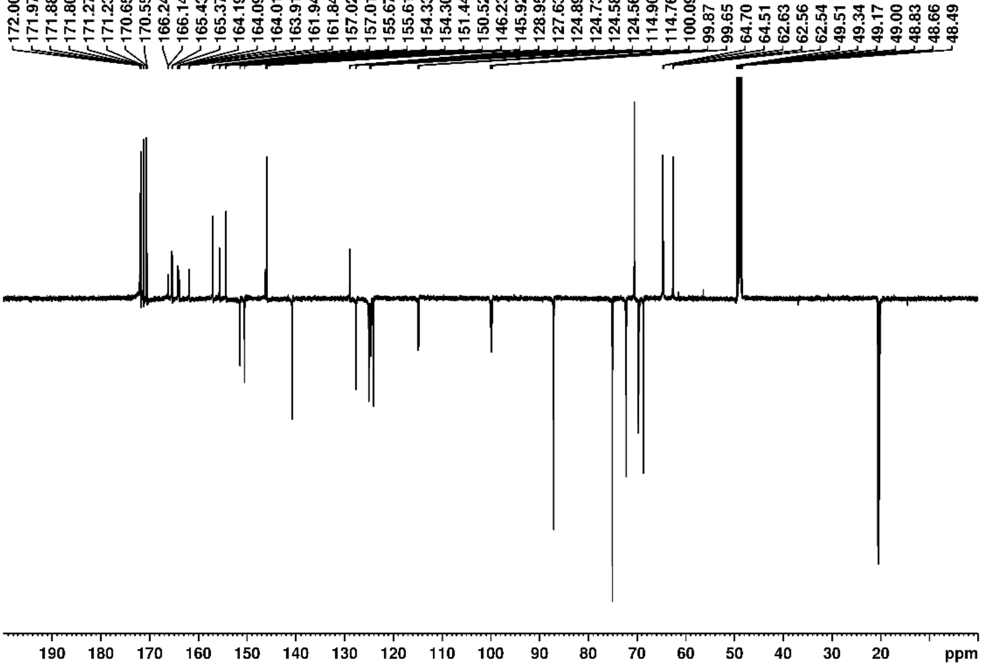

Figure S16 – $^{13}C\{^1H\}$ NMR spectrum (125 MHz, CD_3OD, 298K) for $\mathbf{Ir^{Gal\text{-}Ac}}$ (**17**).

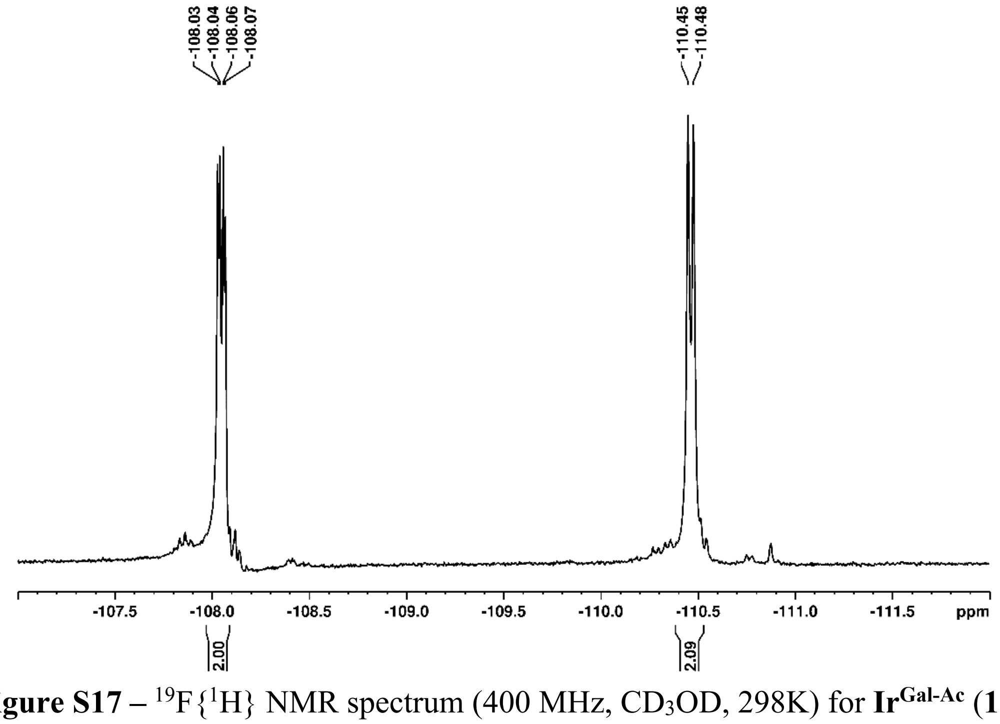

Figure S17 – $^{19}F\{^1H\}$ NMR spectrum (400 MHz, CD_3OD, 298K) for $\mathbf{Ir^{Gal\text{-}Ac}}$ (**17**).

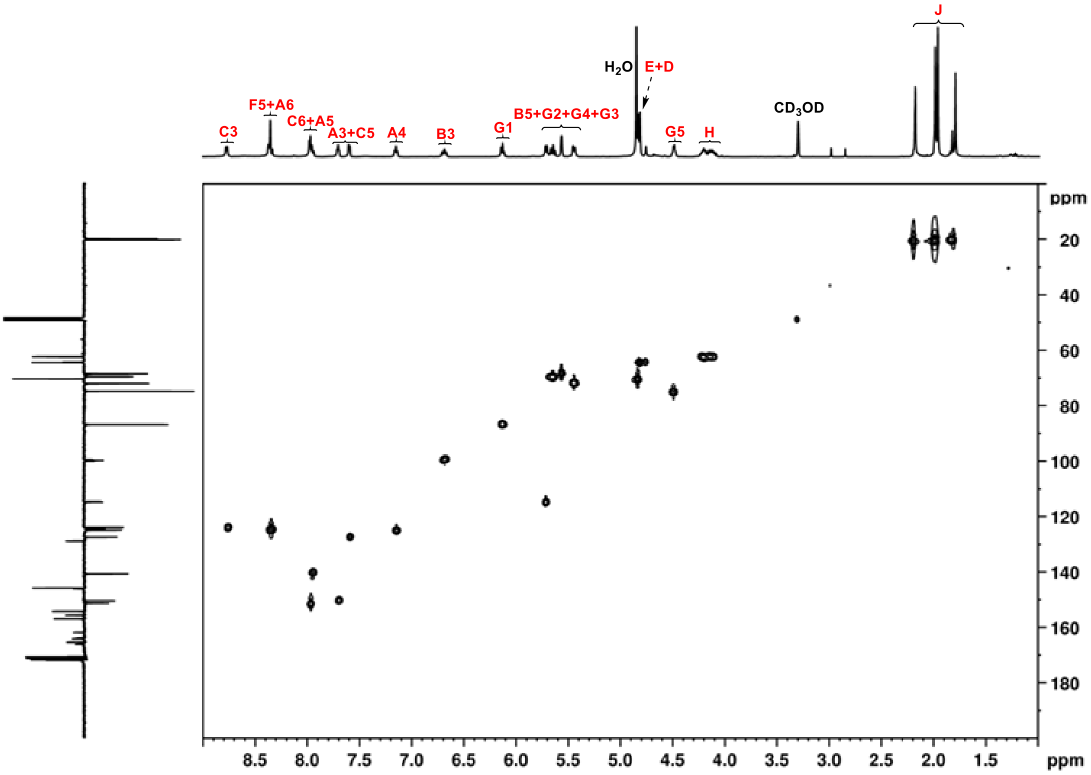

Figure S18 - ^{1}H-$^{13}C\{^{1}H\}$ HSQC NMR spectrum for **Ir$^{\text{Gal-Ac}}$** (**17**).

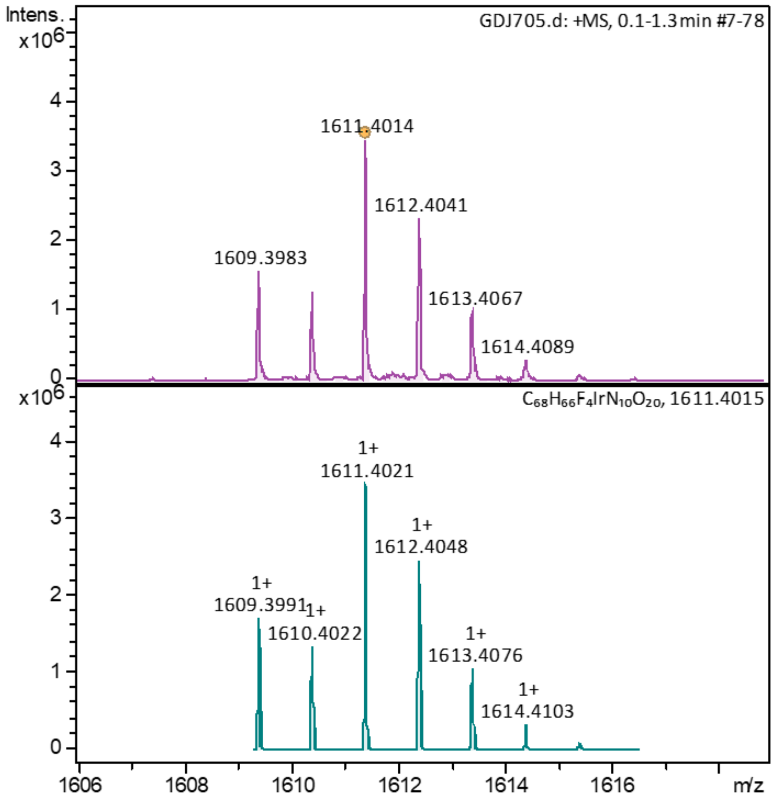

Figure S19 – ESI-HRMS of **Ir$^{\text{Gal-Ac}}$** (**17**) ($[M]^+$).

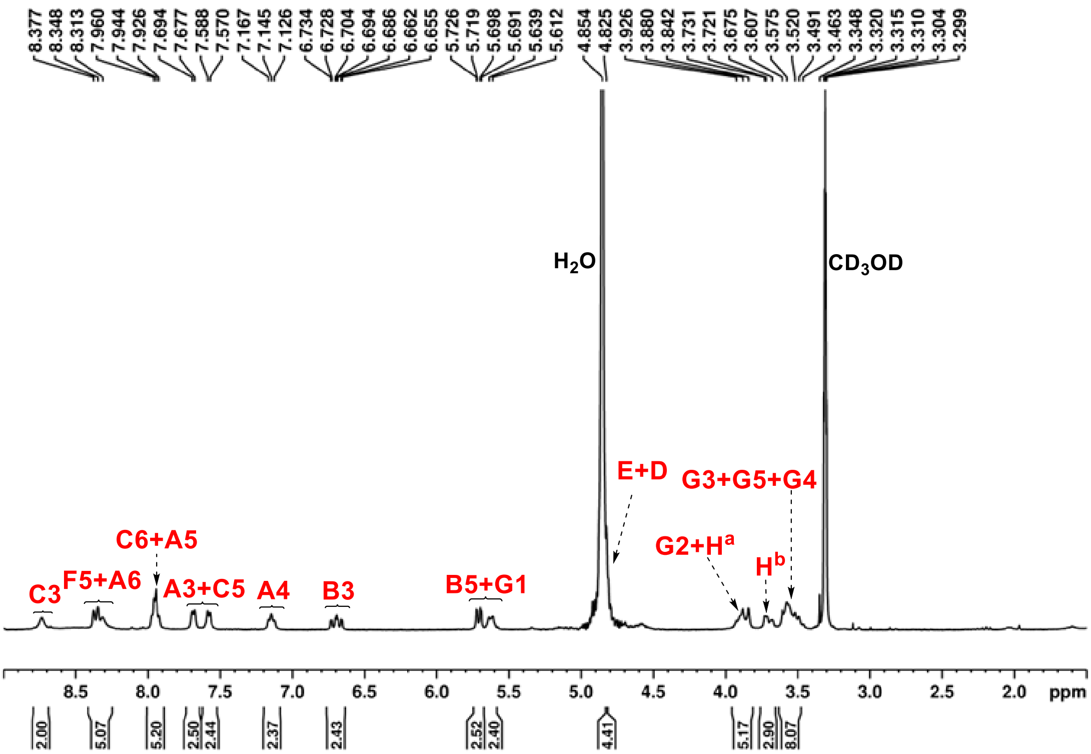

Figure S20 – ^{1}H NMR spectrum (500 MHz, CD_3OD, 298K) for **Ir^{Glu}** (**18**).

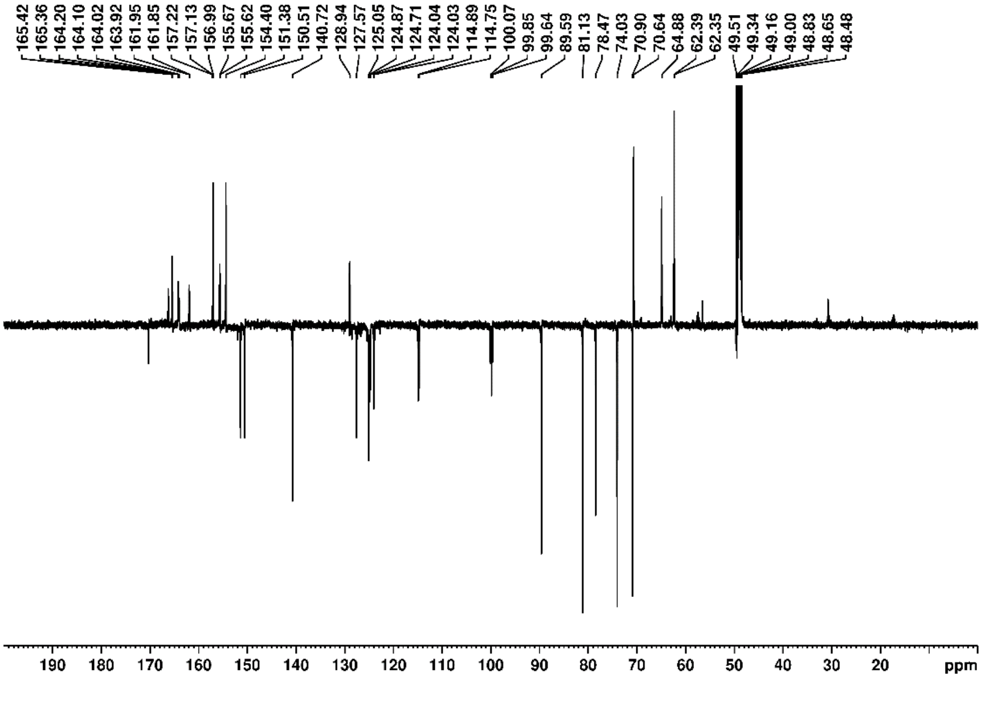

Figure S21 – ^{13}C{^{1}H} NMR spectrum (125 MHz, CD_3OD, 298K) for **Ir^{Glu}** (**18**).

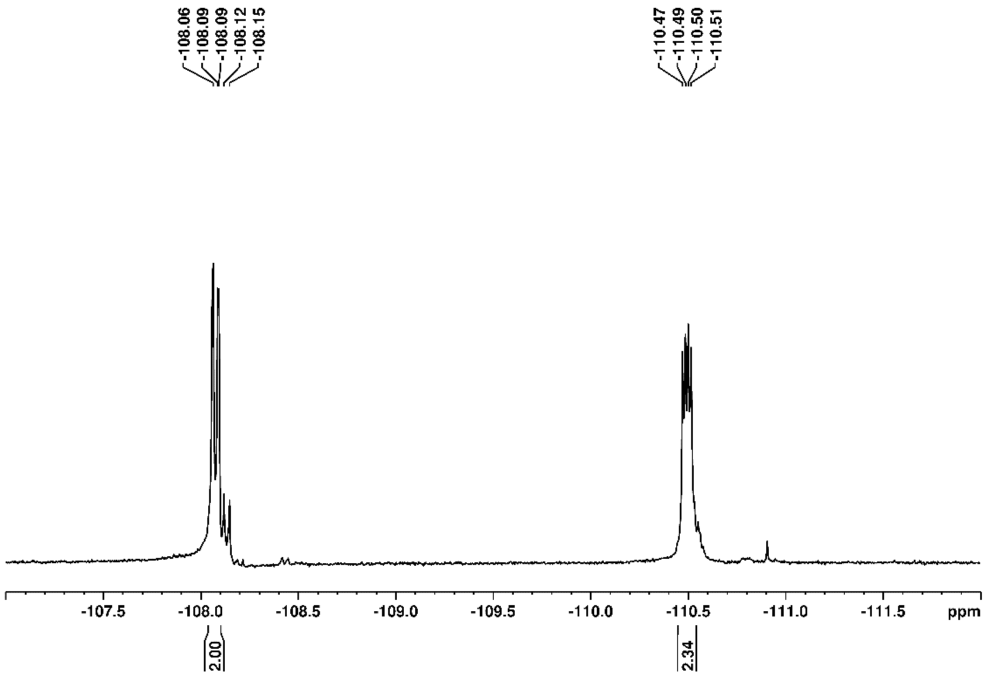

Figure S22 – ^{19}F{^{1}H} NMR spectrum (400 MHz, CD3OD, 298K) for **Ir[Glu]** (**18**).

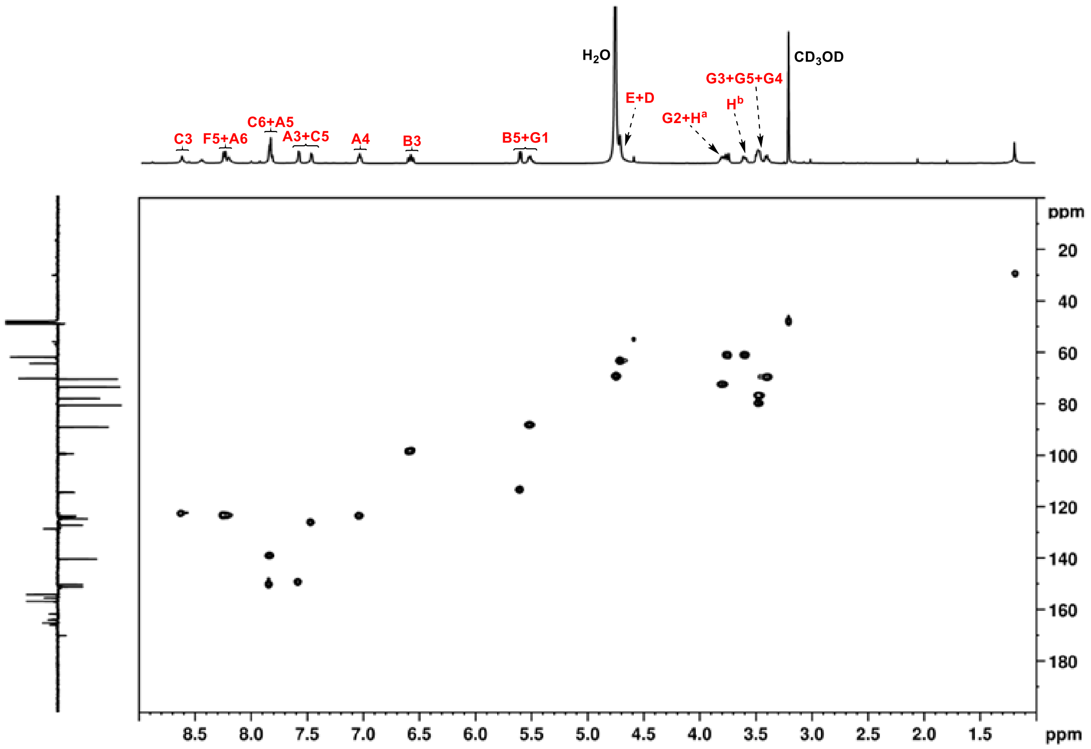

Figure S22 - ^{1}H-^{13}C{^{1}H} HSQC NMR spectrum for **Ir[Glu]** (**18**).

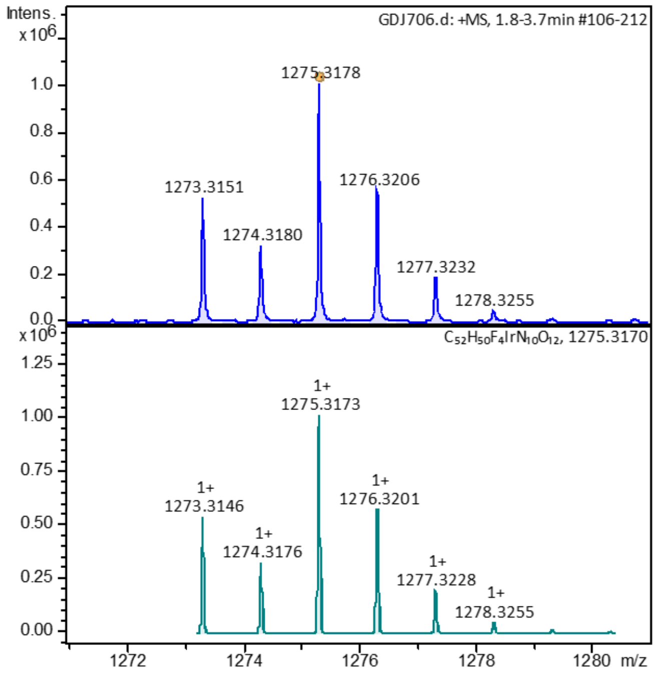

Figure S23 – ESI-HRMS of **Ir**$^{\mathbf{Glu}}$ (**18**) ([M]$^+$).

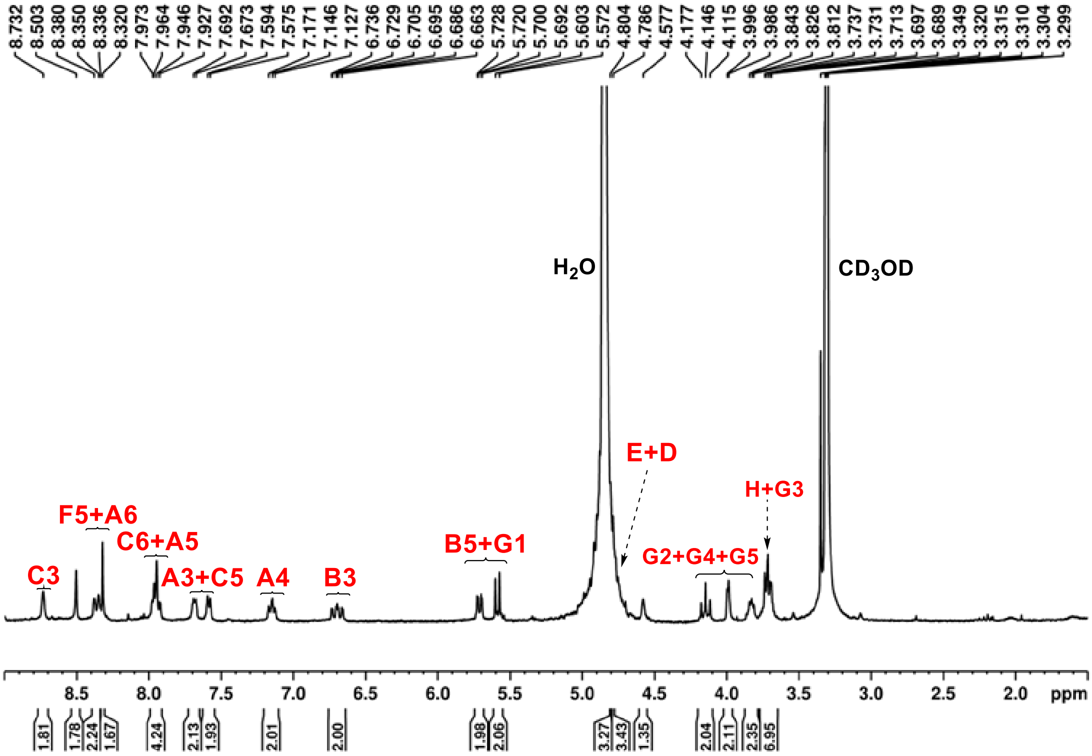

Figure S24 - ^{1}H NMR spectrum (500 MHz, CD_3OD, 298K) for **Ir**$^{\mathbf{Gal}}$ (**19**).

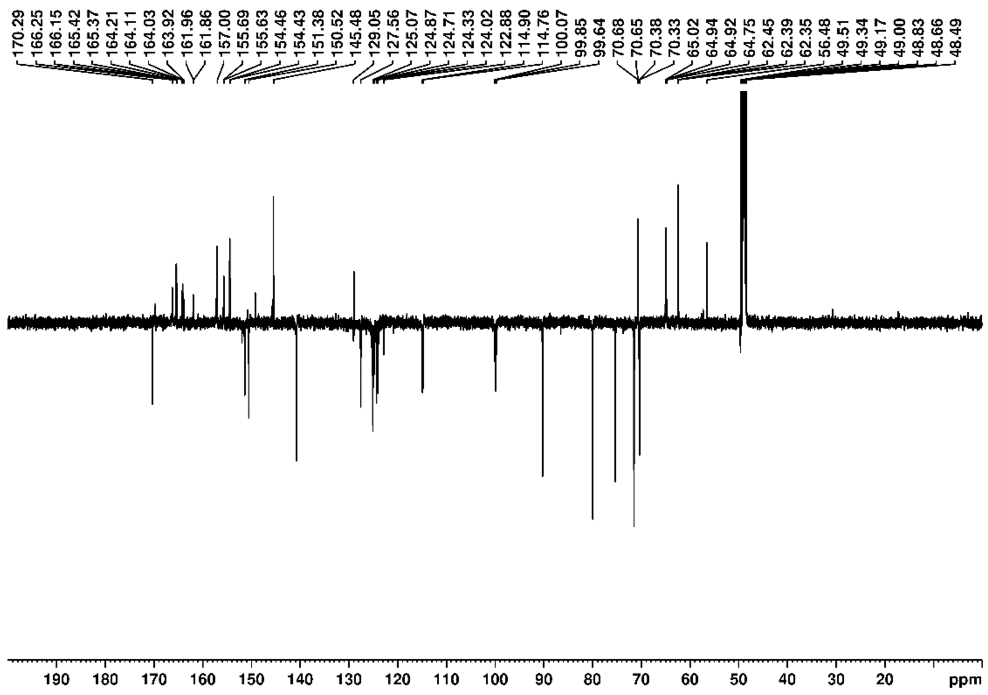

Figure S25 - $^{13}C\{^{1}H\}$ NMR spectrum (125 MHz, CD_3OD, 298K) for **Ir^{Gal}**.

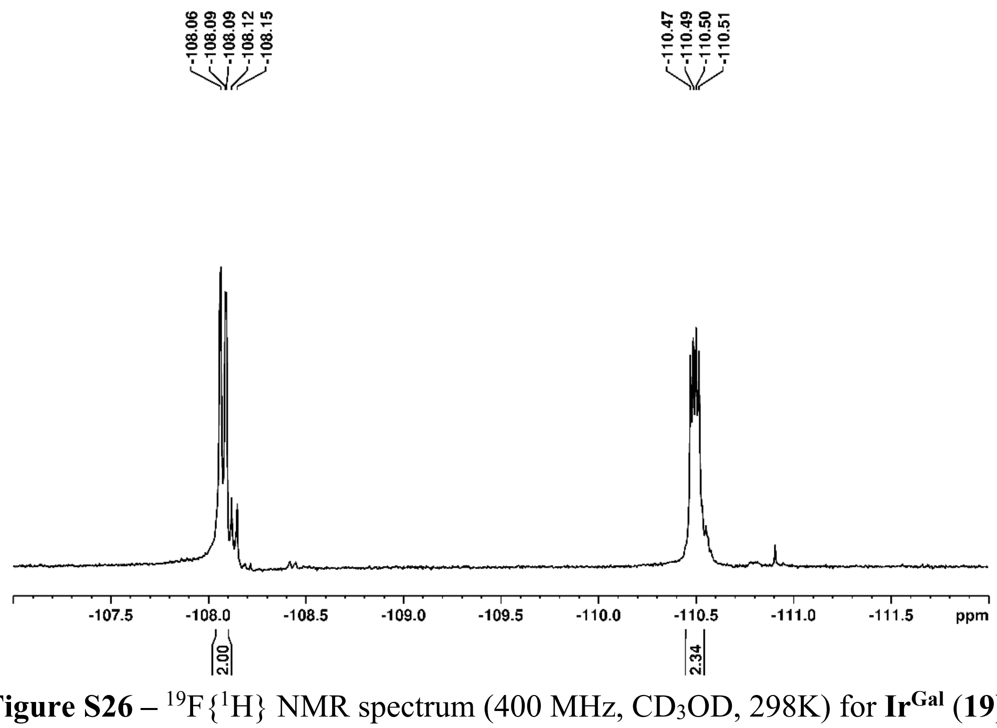

Figure S26 – $^{19}F\{^{1}H\}$ NMR spectrum (400 MHz, CD_3OD, 298K) for **Ir^{Gal}** (**19**).

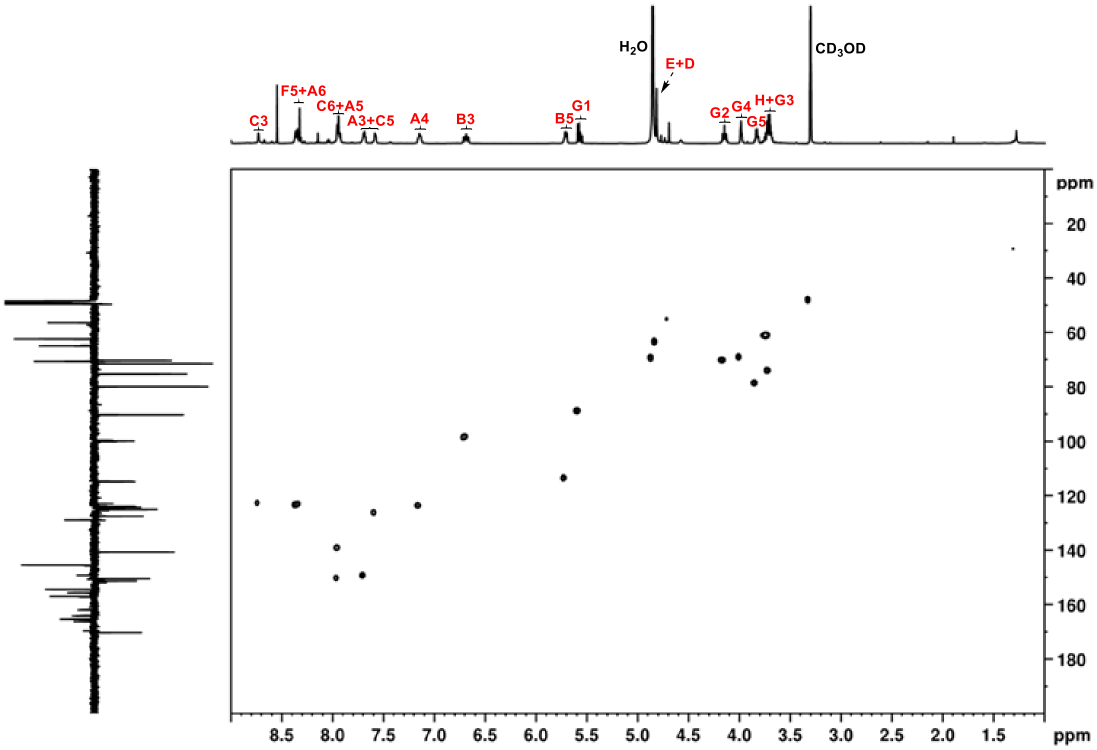

Figure S27 - ^{1}H-^{13}C{^{1}H} HSQC NMR spectrum for **Ir^{Gal}** (**19**).

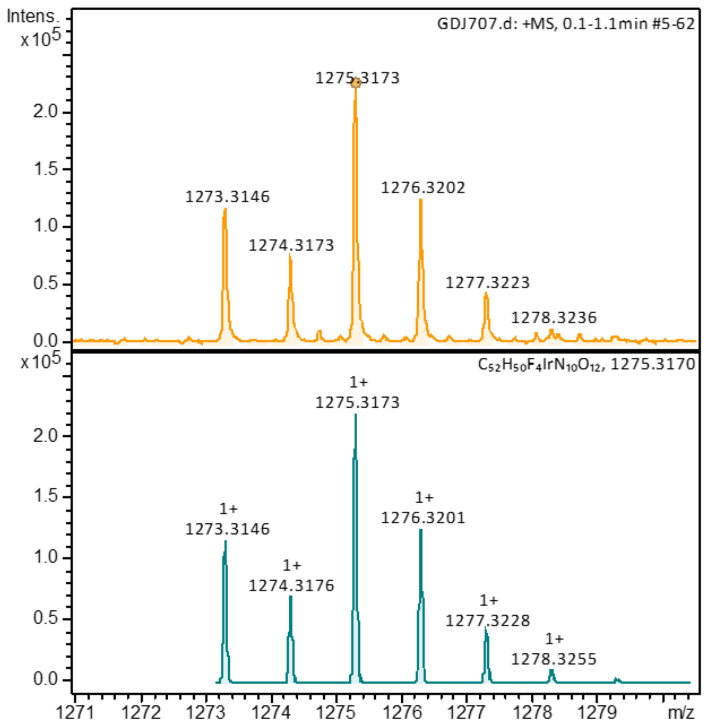

Figure S28 – ESI-HRMS of **Ir^{Gal}** (**19**) ([M]$^+$).

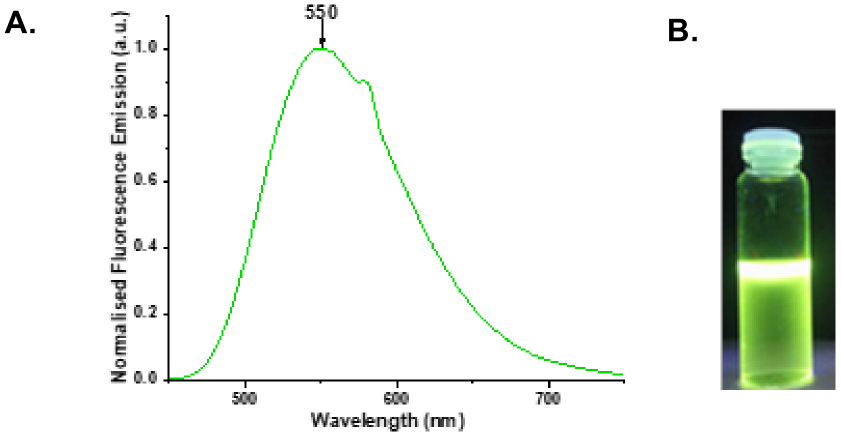

Figure S29 – Example emission spectrum of **Ir**$^{\mathbf{Gal}}$ (**19**) (H_2O, 10 µM) excited at 290 nm (A) (the small shoulder at 580 nm is a 2λ excitation artefact), and the resulting green colour observed when irradiated under a UV-lamp at 365 nm (B).

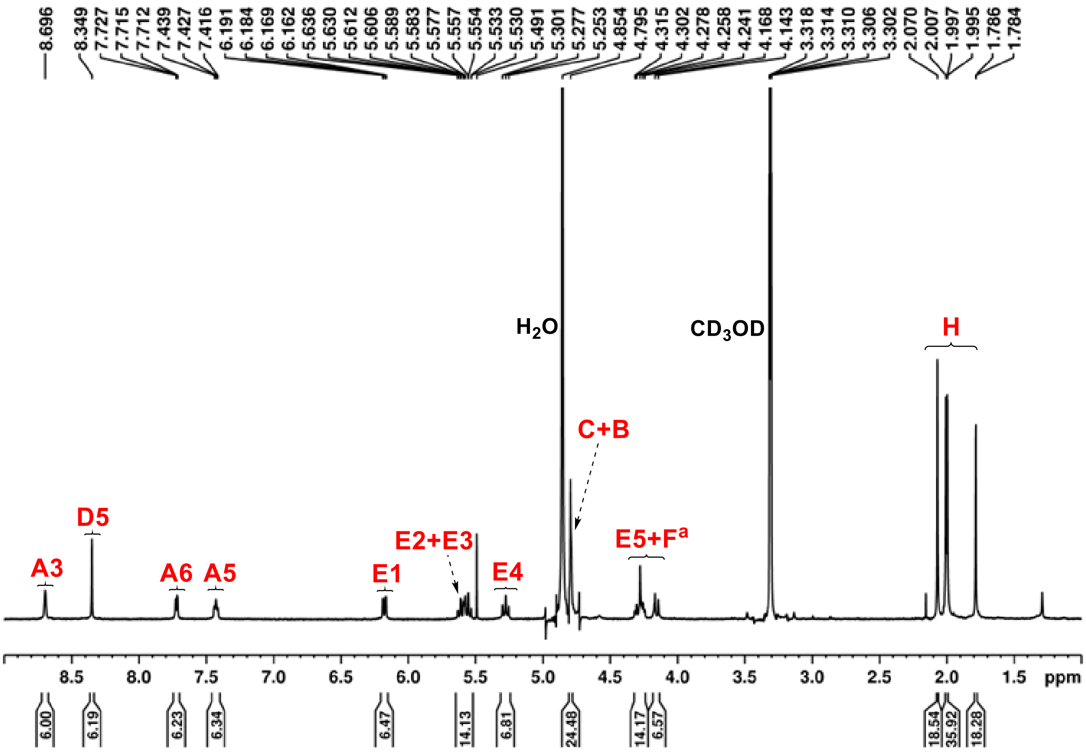

Figures S30 - ^{1}H NMR spectrum (500 MHz, CD_3OD, 298K) for **Ru**$^{\mathbf{Glu\text{-}Ac}}$ (**20**).

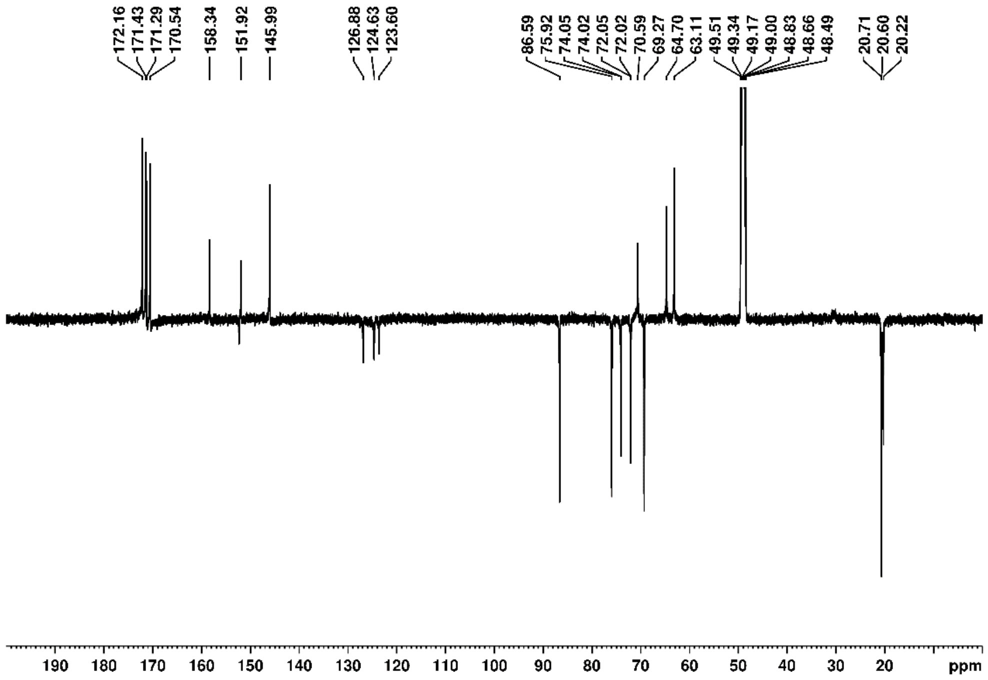

Figure S31 – $^{13}C\{^1H\}$ NMR spectrum (125 MHz, CD_3OD, 298K) for **Ru**$^{\mathbf{Glu\text{-}Ac}}$ (**20**).

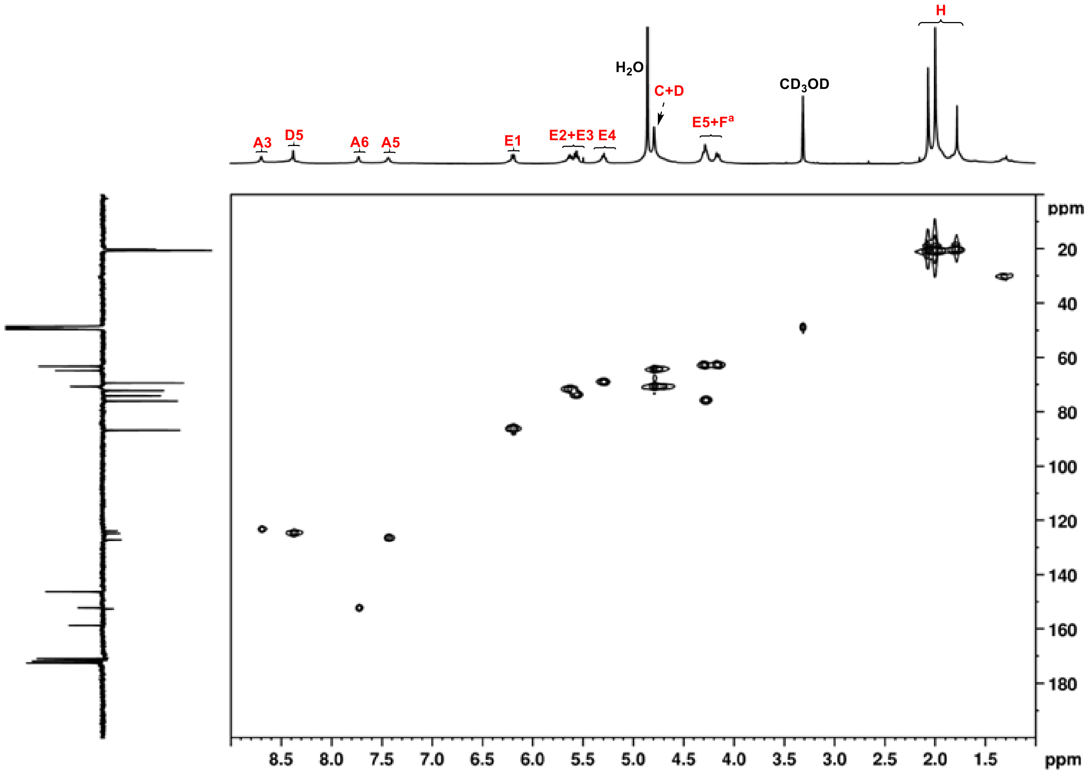

Figure S32 - ^1H-$^{13}C\{^1H\}$ HSQC NMR spectrum for **Ru**$^{\mathbf{Glu\text{-}Ac}}$ (**20**).

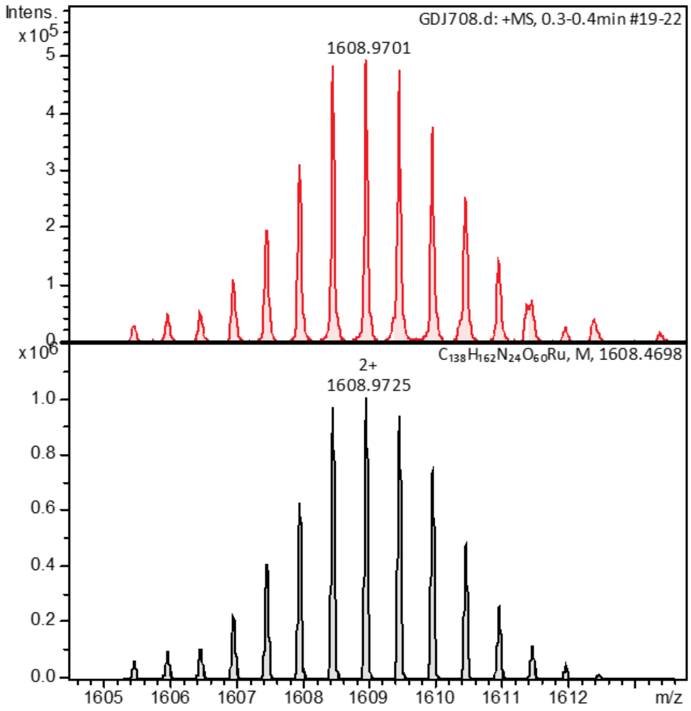

Figure S33 – ESI-HRMS of **Ru$^{\text{Glu-Ac}}$** (**20**) ([M]$^{2+}$)

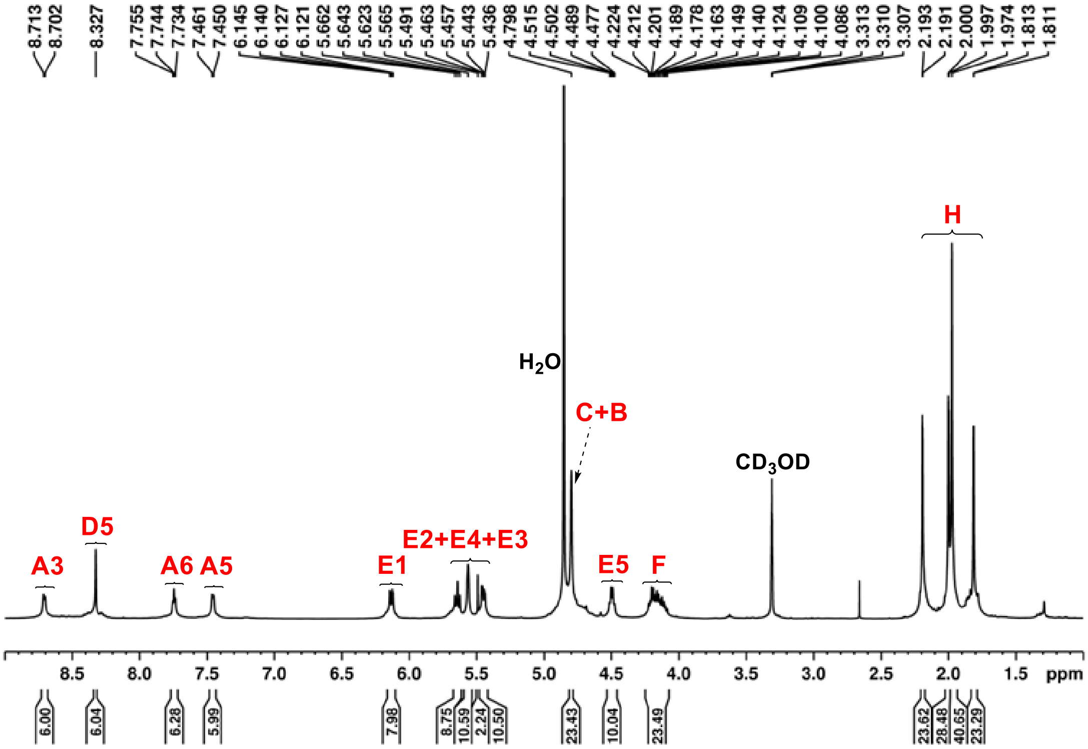

Figure S34 – ^{1}H NMR spectrum (500 MHz, CD$_3$OD, 298K) for **Ru$^{\text{Gal-Ac}}$** (**21**).

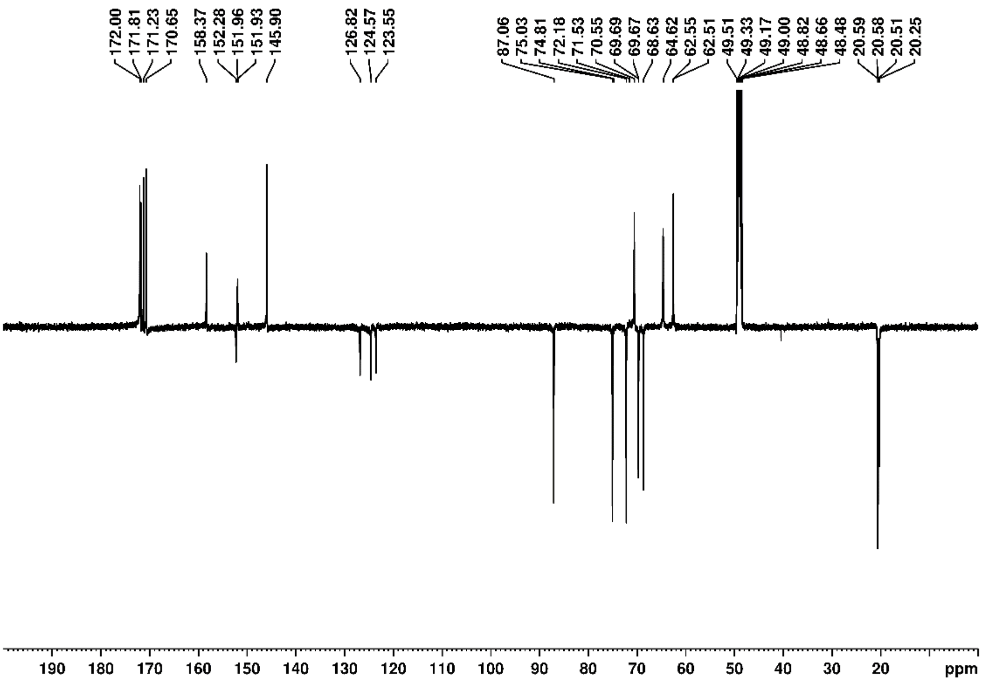

Figure S35 - $^{13}C\{^1H\}$ NMR spectrum (125 MHz, CD_3OD, 298K) for **Ru**$^{\text{Gal-Ac}}$ (**21**).

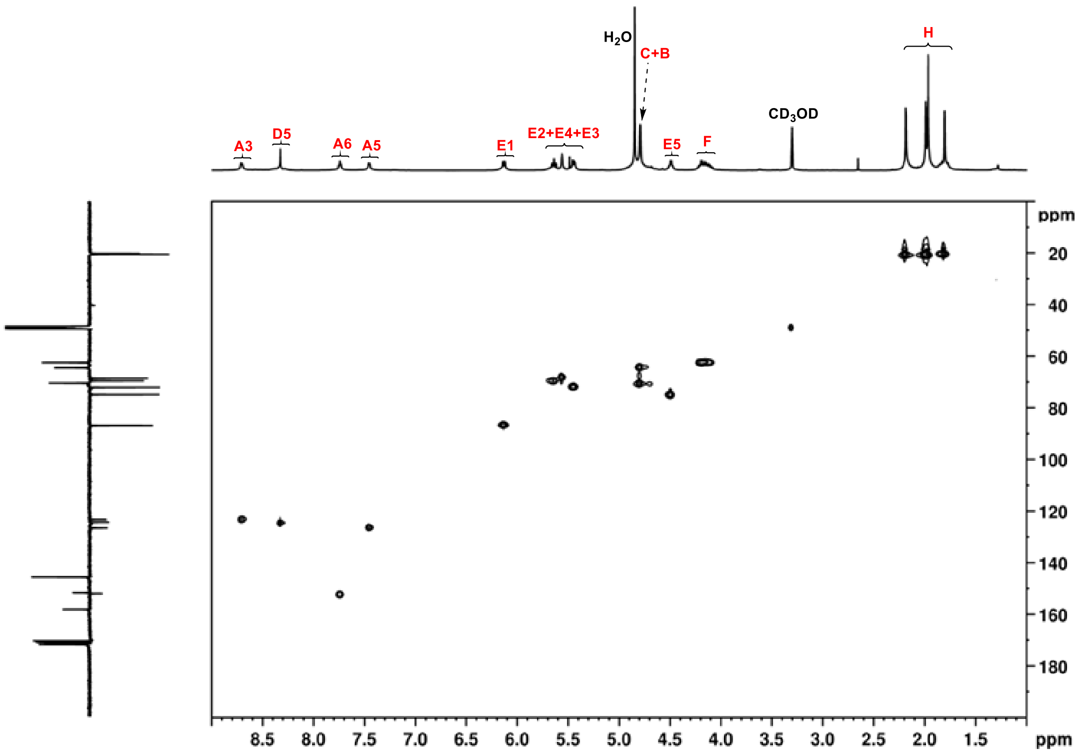

Figure S36 - ^1H-$^{13}C\{^1H\}$ HSQC NMR spectrum for **Ru**$^{\text{Gal-Ac}}$ (**21**).

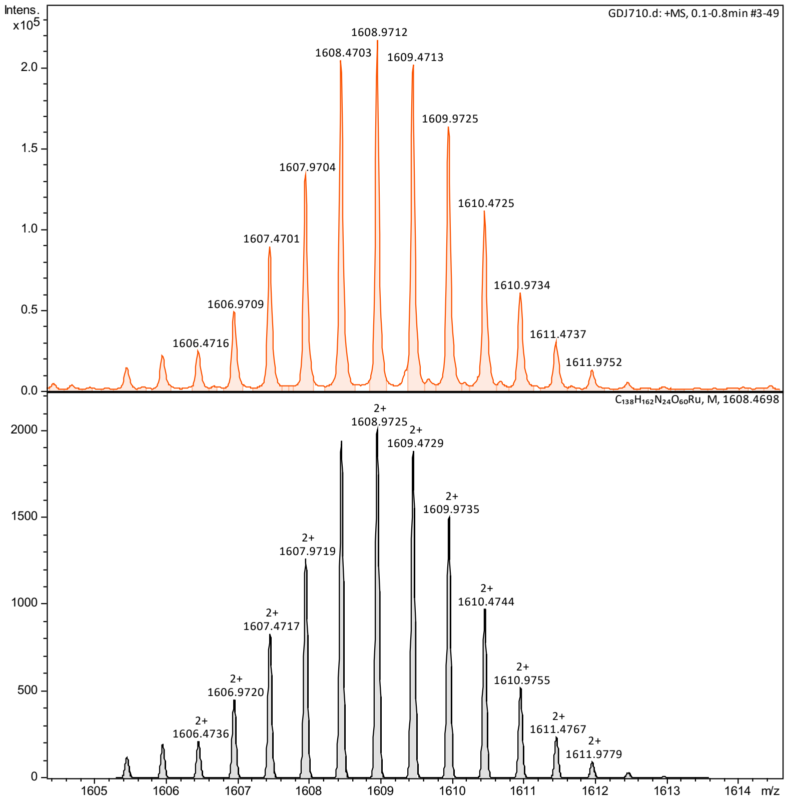

Figure S37 – ESI-HRMS of **Ru**$^{\mathbf{Gal\text{-}Ac}}$ (**21**) ([M]$^{2+}$).

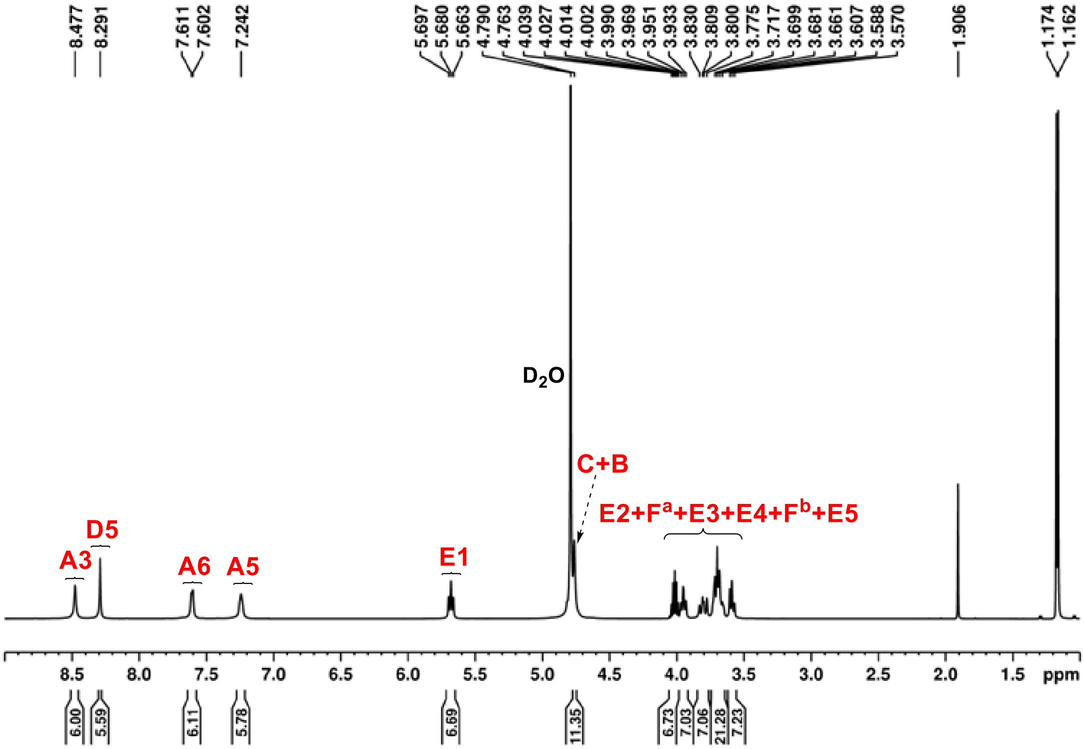

Figure S38 - ^{1}H NMR spectrum (500 MHz, D_2O, 298K) for **Ru**$^{\mathbf{Glu}}$ (**22**).

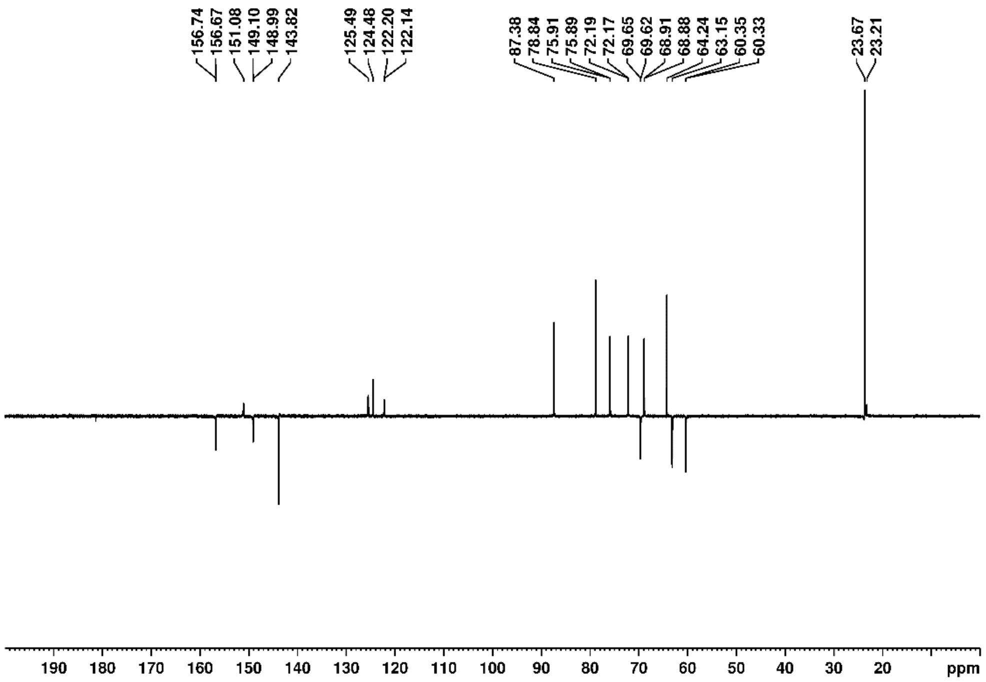

Figure S39 - $^{13}C\{^{1}H\}$ NMR spectrum (125 MHz, D_2O, 298K) for **Ru^{Glu}** (**22**).

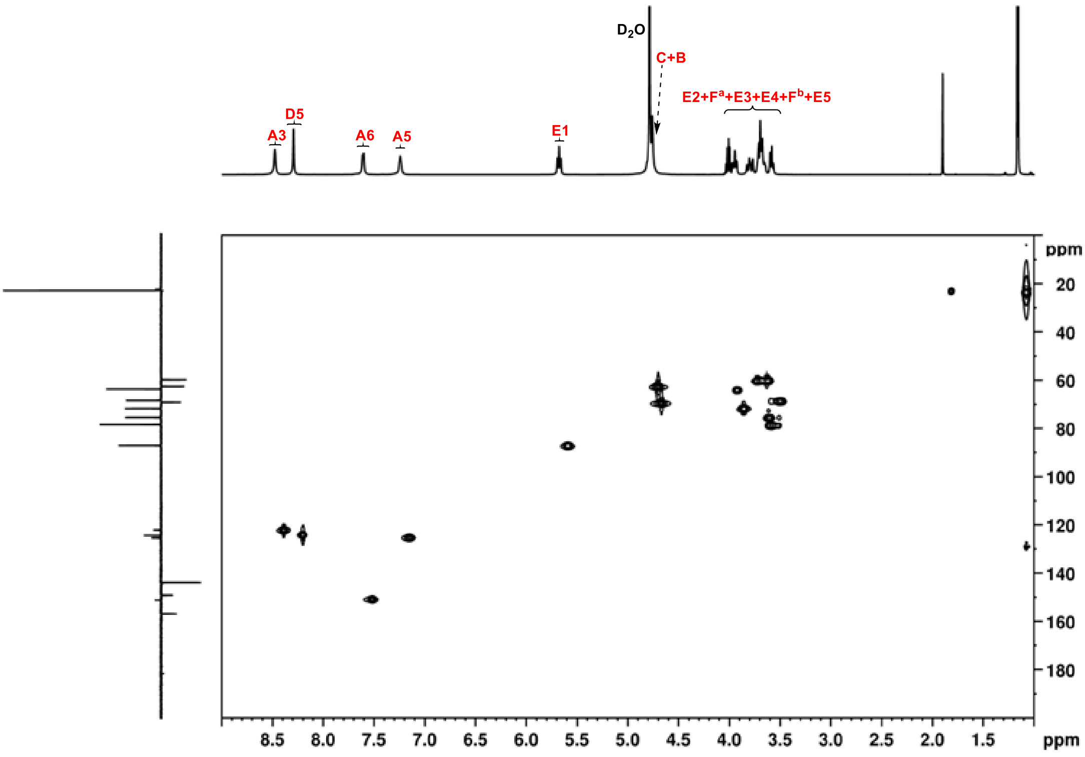

Figure S40 - ^{1}H-$^{13}C\{^{1}H\}$ HSQC NMR spectrum for **Ru^{Glu}** (**22**).

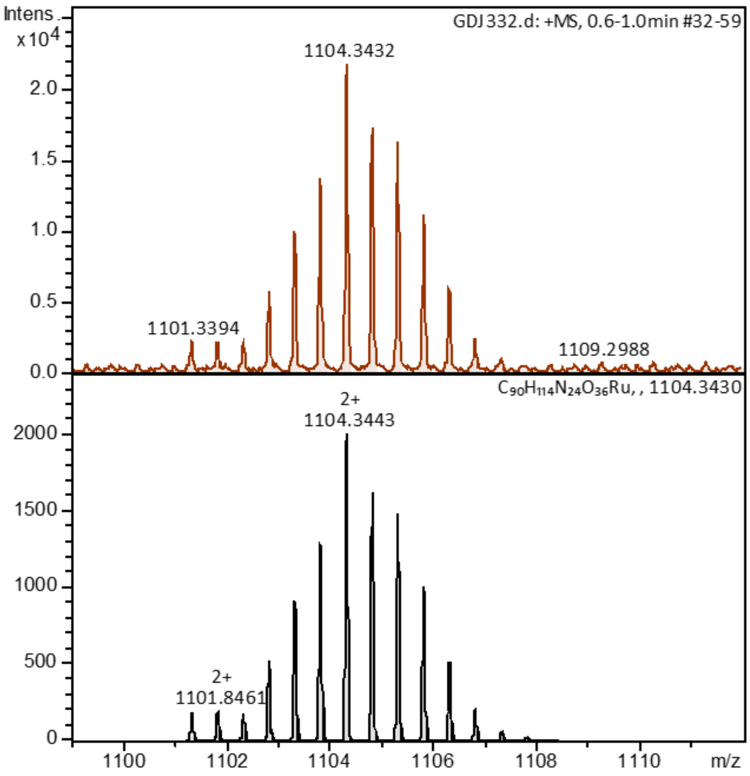

Figure S41 – ESI-HRMS of **Ru**$^{\mathbf{Glu}}$ (**22**) ([M]$^{2+}$).

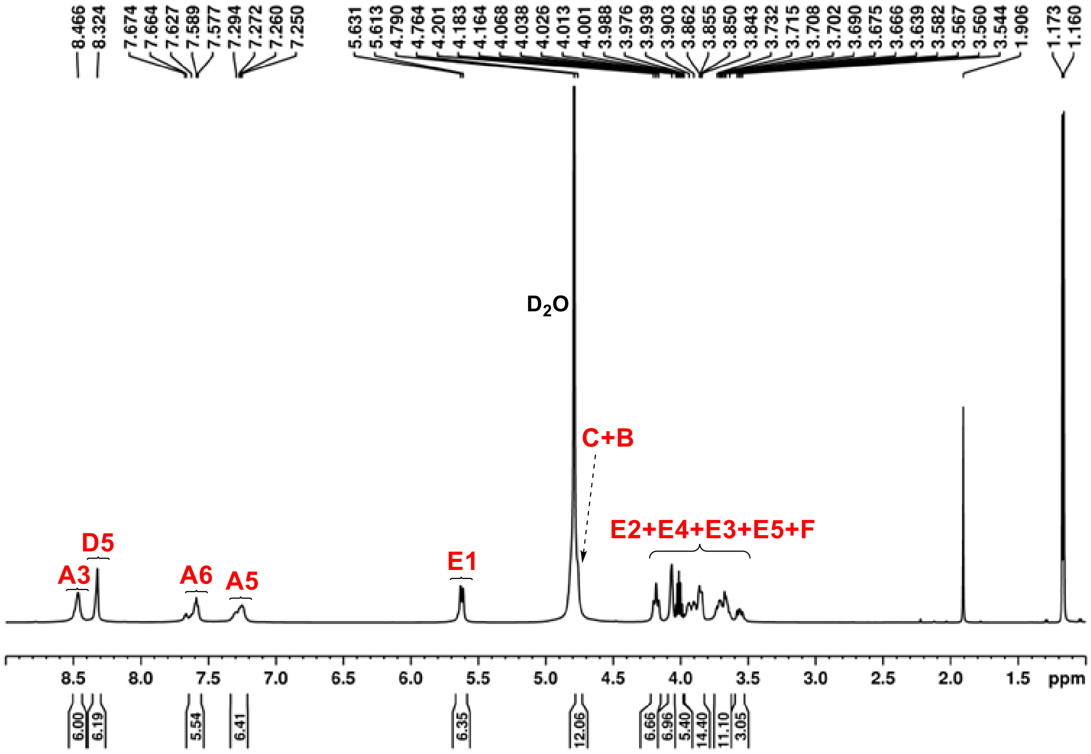

Figure S42 - ^{1}H NMR spectrum (500 MHz, D_2O, 298K) for **Ru**$^{\mathbf{Gal}}$ (**23**).

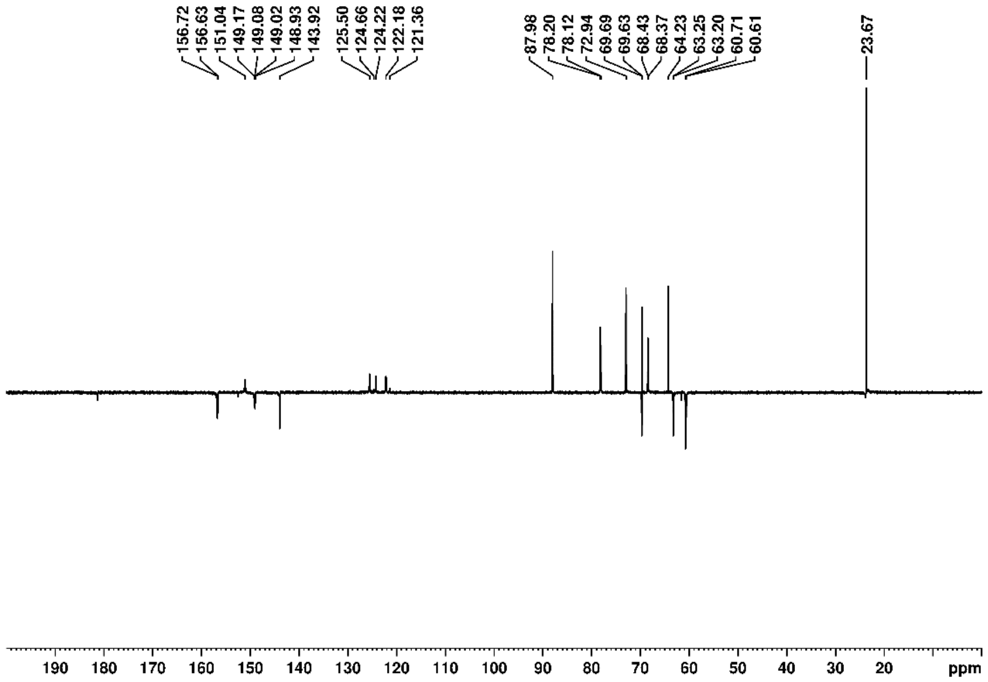

Figure S43 - ^{13}C{^{1}H} NMR spectrum (125 MHz, D_2O, 298K) for **Ru^{Gal}** (**23**).

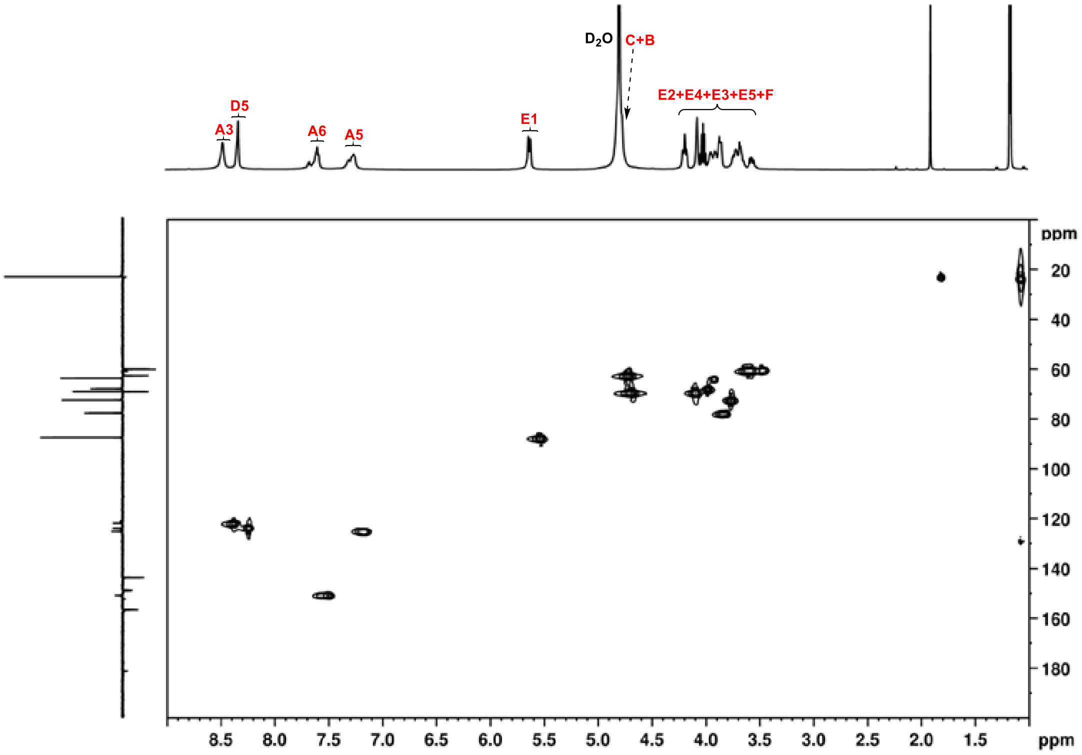

Figure S44 - ^{1}H-^{13}C{^{1}H} HSQC NMR spectrum for **Ru^{Gal}** (**23**).

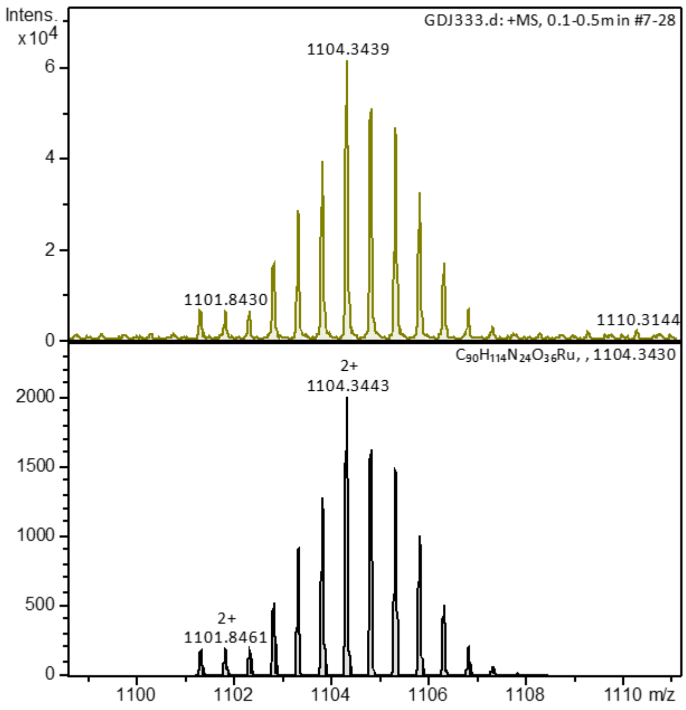

Figure S45 – ESI-HRMS of **Ru^{Gal}** (**23**) ([M]$^{2+}$).

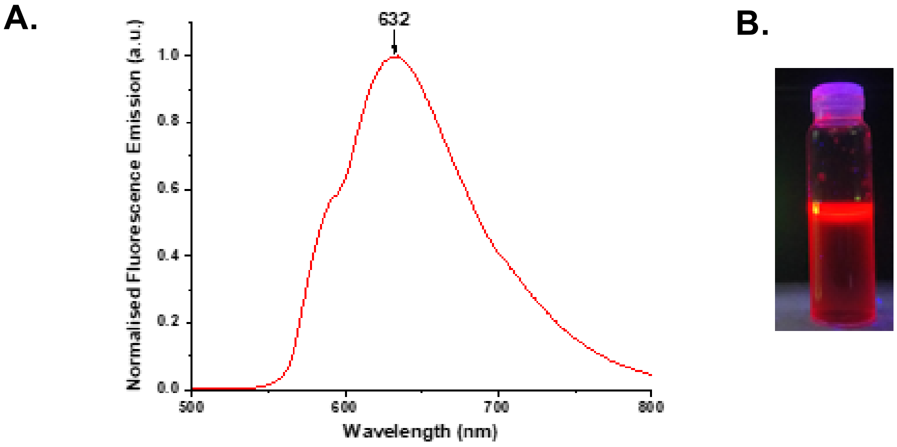

Figure S46 – Example emission spectrum of **Ru^{Gal}** (**23**) (H_2O, 10 μM) excited at 290 nm (A) (the small shoulder at 580 nm is a 2λ excitation artefact), and the resulting characteristic red colour observed when irradiated under a UV-lamp at 365 nm (B).

Synthesis of cubic cage ligands

L[15-Glu-Ac] (28)

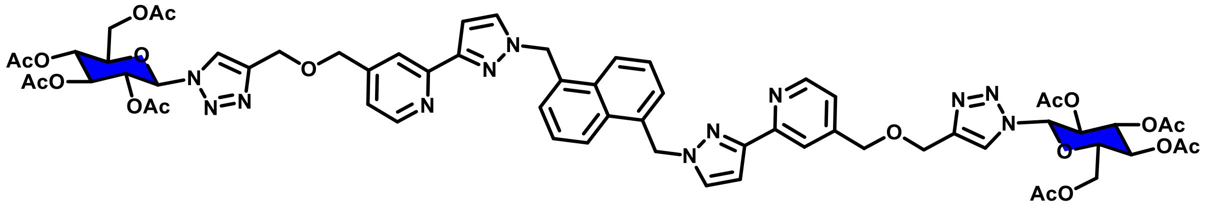

L[15CC (9)] (250 mg, 0.43 mmol) and **8** (403 mg, 1.08 mmol, 2.5 eq.) were added to a two-neck RBF under N_2, dissolved in CH_2Cl_2 (10 mL) and left to stir for 10 mins. A solution of $CuSO_4$•$5H_2O$ (78 mg, 0.31 mmol, 0.72 eq.) and NaAsc (86 mg, 0.43 mmol, 1.00 eq.) in H_2O (10 mL) was then added to this mixture, forming a biphasic orange and red coloured system. The mixture was then heated continuously for 72 h at 60 °C. TLC analysis after this time (Silica; CH_2Cl_2/MeOH - 9:1) indicated the presence of a new product at R_f: 0.44.

The reaction was then cooled to RT and quenched with the addition of an $EDTA_{aq}$ (5 mL, 1.5 M) solution in water (10 mL) and left to stir overnight with additional CH_2Cl_2 (10 mL). The organic phase was separated and the aqueous phase extracted with CH_2Cl_2 (3 x 20 mL). Combined organic phases were dried over Na_2SO_4 and evaporated to give an orange/brown crude solid. Product was purified on a long Sephadex® LH-20 column using a CH_2Cl_2/MeOH (70:30) eluent to yield a fine yellow powder. Yield: 485 mg, 0.37 mmol, 85%.

^{1}H-NMR (300 MHz, **$CDCl_3$**, 298 K) δppm: 8.61 (2H, d, J = 4.6 Hz, pyridyl H^6); 8.03 (2H, d, 8.3 Hz, naphthyl $H^{4/8}$); 7.93 (2H, s, pyridyl H^3); 7.84 (2H, s, triazole H^5); 7.48 (2H, t, J = 7.5 Hz, naphthyl $H^{3/7}$); 7.33 (2H, d, J = 6.8 Hz, naphthyl $H^{2/6}$); 7.26-7.20 (4H, m, pyrazole H^5 + pyridyl H^5); 6.86 (2H, s, J = 2.0 Hz, pyrazole H^4); 5.88-5.86 (2H, m, glucose H^1); 5.86 (4H, s, pyridyl-C**H_2**N); 5.46-5.39 (4H, dt, J = 9.6, 4.2 Hz, glucose H^{2+3}); 5.23 (2H, t, J = 9.4 Hz, glucose H^4); 4.74 (4H, s, pyridyl-C**H_2**O); 4.64 (4H, s, O-C**H_2**-CNR); 4.29 (2H, dd, J = 12.7, 4.4 Hz, glucose H^{6b}); 4.14 (2H, dd, J = 12.5, 1.9 Hz, glucose H^{6a}); 3.99 (2H, d, J = 6.1 Hz, glucose H^5); 2.06 (12H, s, methyl C**H_3**); 2.01 (6H, s, methyl C**H_3**); 1.86 (6H, s, methyl C**H_3**).

^{13}C-NMR (75 MHz, **$CDCl_3$**, 298 K) δppm: 170.5, 169.9, 169.3, 168.9 (**C**OCH$_3$); 145.4 (triazole C^4); 132.3 (naphthyl $C^{1/5}$ or $C^{9/10}$); 131.7 (naphthyl $C^{9/10}$ or $C^{1/5}$); 131.0 (pyrazole C^5), 127.4 (naphthyl $C^{2/6}$); 126.5 (naphthyl $C^{3/7}$); 124.4 (naphthyl $C^{4/8}$); 121.2 (triazole C^5); 120.8 (pyridyl C^5); 118.5 (pyridyl C^3); 105.4 (pyrazole C^4); 85.8 (glucose C^1); 75.2 (glucose C^5); 72.6

(glucose C^3); 70.7 (O-**C**H_2-CNR); 70.4 (glucose C^2); 67.7 (glucose C^4); 63.9 (pyridyl-**C**H_2O); 61.5 (glucose C^6); 54.7 (pyridyl-**C**H_2N); 20.7, 20.5, 20.5, 20.2 (CO**C**H_3).

High-resolution ES-MS: *m/z* observed, 1325.4772. Calculated for $C_{64}H_{69}N_{12}O_{20}^{+}$ [M + H^+], 1325.4746. Observed, 663.2400. Calculated for $C_{64}H_{70}N_{12}O_{20}^{2+}$ [M + $2H^+$], 663.2409.

ν_{max}/cm^{-1}: 2933w, 1744s, 1366m, 1210s, 1092m, 1034s.

$L^{15\text{-Gal-Ac}}$ (29)

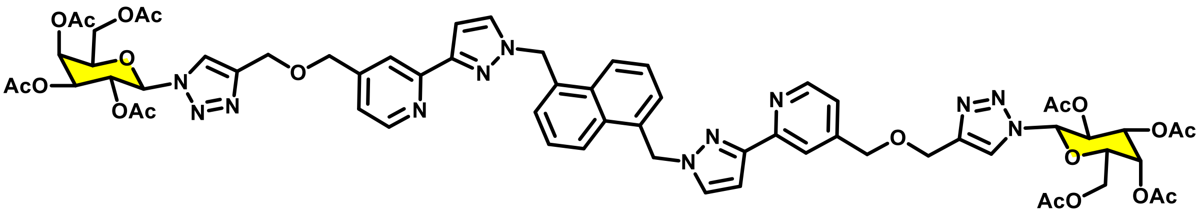

Compound **12** (403 mg, 1.08 mmol, 2.5 eq.) used with the same CuAAC reaction conditions described for **28**. Yield: 500 mg, 0.38 mmol, 88%.

^{1}H-NMR (300 MHz, **$CDCl_3$**, 298 K) δppm: 8.61 (2H, d, J = 5.0 Hz, pyridyl H^6); 8.03 (2H, d, 8.6 Hz, naphthyl $H^{4/8}$); 7.94 (2H, s, pyridyl H^3); 7.90 (2H, s, triazole H^5); 7.48 (2H, t, J = 8.5 Hz, naphthyl $H^{3/7}$); 7.32 (2H, d, J = 7.0 Hz, naphthyl $H^{2/6}$); 7.26-7.24 (4H, m, pyrazole H^5 + pyridyl H^5); 6.86 (2H, d, J = 2.4 Hz, pyrazole H^4); 5.86 (4H, s, pyridyl-C**H_2**N); 5.83 (2H, d, J = 9.0 Hz, galactose H^1); 5.54 (4H, dd, J = 10.6, 9.1 Hz, galactose H^{2+4}); 5.24 (2H, dd, J = 10.2, 3.4 Hz, galactose H^3); 4.75 (4H, s, pyridyl-C**H_2**O); 4.64 (4H, s, O-C**H_2**-CNR); 4.25-4.10 (6H, m, galactose $H^{6a+6b+5}$); 2.20 (6H, s, methyl C**H_3**); 2.03 (6H, s, methyl C**H_3**); 2.00 (6H, s, methyl C**H_3**); 1.88 (6H, s, methyl C**H_3**).

^{13}C-NMR (75 MHz, **$CDCl_3$**, 298 K) δppm: 170.3, 169.9, 169.8, 169.1 (**C**OCH_3); 152.1 (pyridyl C^2); 151.3 (pyrazole C^3); 149.4 (pyridyl C^6); 147.9 (pyridyl C^4); 145.3 (triazole C^4); 132.3 (naphthyl $C^{1/5}$ or $C^{9/10}$); 131.7 (naphthyl $C^{9/10}$ or $C^{1/5}$); 130.9 (pyrazole C^5); 127.4 (naphthyl $C^{2/6}$); 126.5 (naphthyl $C^{3/7}$); 124.4 (naphthyl $C^{4/8}$); 121.2 (triazole C^5); 120.7 (pyridyl C^5); 118.3 (pyridyl C^3); 105.0 (pyrazole C^4); 86.3 (galactose C^1); 74.1 (galactose C^5); 70.7 (O-**C**H_2-CNR + galactose C^3); 67.9 (galactose C^2); 66.8 (galactose C^4); 64.0 (pyridyl-**C**H_2O); 61.2 (galactose C^6); 54.7 (pyridyl-**C**H_2N); 20.6, 20.5, 20.3 (CO**C**H_3).

High-resolution ES-MS: *m/z* observed, 1325.4782. Calculated for $C_{64}H_{69}N_{12}O_{20}^+$ [M + H^+], 1325.4746. Observed, 663.2401. Calculated for $C_{64}H_{70}N_{12}O_{20}^{2+}$ [M + $2H^+$], 663.2409.

ν_{max}/cm^{-1}: 2962w, 1745s, 1367m, 1210s, 1089m, 1042s.

$L^{15\text{-}Glu}$ (30)

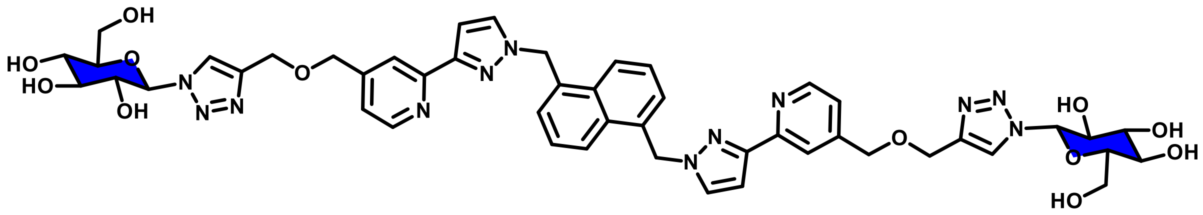

$L^{15\text{-}Glu\text{-}Ac}$ (93 mg, 70 μmol) was added to a 2-neck RBF under N_2, charged with dry MeOH (15 mL). NaOMe (1 M solution in dry MeOH) was then added until pH 9-10 was achieved and a precipitate was observed. The solution was left to stir for 18 h, neutralised with Amberlite® IR-120 (H) resin and then filtered. The solvent was evaporated to yield a fine yellow/brown solid and used without further purification. Yield: 66 mg, 67 μmol, 95%.

^{1}H-NMR (300 MHz, **DMSO-d_6**, 298 K) δppm: 8.52 (2H, d, J = 4.9 Hz, pyridyl H^6); 8.39 (2H, s, triazole H^5); 8.22 (2H, d, J = 8.4 Hz, naphthyl $H^{4/8}$); 7.89 (2H, s, pyridyl H^3); 7.86 (2H, s, pyrazole H^5); 7.58 (2H, t, J = 7.7 Hz, naphthyl $H^{3/7}$); 7.29-7.25 (4H, m, naphthyl $H^{2/6}$ + pyridyl H^5); 6.87 (2H, d, J = 2.0 Hz, pyrazole H^4); 5.94 (4H, s, pyridyl-C**H_2**N); 5.55 (2H, d, J = 9.2 Hz, glucose H^1); 4.97 (8H, brs, glucose-OH); 4.65 (4H, s, pyridyl-C**H_2**O); 4.64 (4H, s, O-C**H_2**-CNR); 3.79 (2H, t, J = 9.0 Hz, glucose H^2); 3.70 (2H, d, J = 9.8 Hz, glucose H^6); 3.51-3.39 (6H, m, glucose H^{3+4+6}); 3.25 (2H, t, J = 8.7 Hz, glucose H^5).

^{13}C-NMR (75 MHz, **DMSO-d_6**, 298 K) δppm: 151.7 (pyridyl C^2); 150.9 (pyrazole C^3); 149.2 (pyridyl C^6); 147.9 (pyridyl C^4) 143.4 (triazole C^4); 133.7 (naphthyl $C^{1/5}$ or $C^{9/10}$); 132.3 (pyrazole C^5), 130.9 (naphthyl $C^{9/10}$ or $C^{1/5}$); 126.3 (naphthyl $C^{2/6}$); 126.2 (naphthyl $C^{3/7}$); 123.8 (naphthyl $C^{4/8}$); 123.4 (triazole C^5); 120.5 (pyridyl C^5); 117.1 (pyridyl C^3); 104.4 (pyrazole C^4); 87.5 (glucose C^1); 79.9 (glucose C^4); 76.9 (glucose C^3); 72.1 (glucose C^2); 69.7 (O-**C**H_2-CNR); 69.5 (glucose C^5); 63.2 (pyridyl-**C**H_2O); 60.7 (glucose C^6); 53.3 (pyridyl-**C**H_2N).

High-resolution ES-MS: *m/z* observed, 989.3914. Calculated for $C_{48}H_{53}N_{12}O_{12}^+$ [M + H^+], 989.3900. Observed, 495.1984. Calculated for $C_{48}H_{54}N_{12}O_{12}^{2+}$ [M + $2H^+$], 495.1987.

ν_{max}/cm^{-1}: 3273br, 3143br, 2919w, 1604m, 1354m, 1258m, 1091s, 1014s

L[15-Gal] (31)

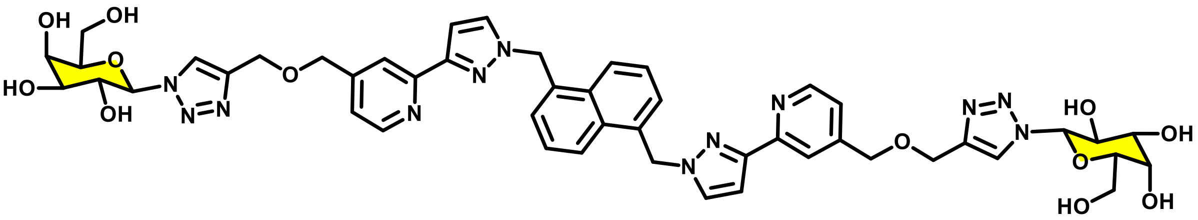

L[15-Gal-Ac] (94 mg, 71 µmol) used with the same deprotection conditions described for **30**. Yield: 66 mg, 67 µmol, 94%.

^{1}H-NMR (300 MHz, **DMSO-*d*$_6$**, 298 K) *δ*ppm: 8.52 (2H, d, J = 4.1 Hz, pyridyl H^6); 8.33 (2H, s, triazole H^5); 8.22 (2H, d, J = 8.3 Hz, naphthyl $H^{4/8}$); 7.89 (2H, s, pyridyl H^3); 7.86 (2H, s, pyrazole H^5); 7.58 (2H, t, J = 7.7 Hz, naphthyl $H^{3/7}$); 7.29-7.25 (4H, m, naphthyl $H^{2/6}$ + pyridyl H^5); 6.86 (2H, d, J = 2.0 Hz, pyrazole H^4); 5.94 (4H, s, pyridyl-C**H$_2$**N); 5.55 (2H, d, J = 9.1 Hz, galactose H^1); 5.04 (8H, brs, galactose-OH); 4.66 (4H, s, pyridyl-C**H$_2$**O); 4.64 (4H, s, O-C**H$_2$**-CNR); 4.06 (2H, t, J = 9.1 Hz, galactose H^2); 3.78 (2H, s, galactose H^5); 3.72 (2H, t, J = 6.2 Hz, galactose H^4); 3.58-3.46 (6H, m, galactose $H^{3+6a+6b}$).

^{13}C-NMR (75 MHz, **DMSO-*d*$_6$**, 298 K) *δ*ppm: 151.7 (pyridyl C^2); 150.9 (pyrazole C^3); 149.2 (pyridyl C^6); 147.9 (pyridyl C^4) 143.5 (triazole C^4); 133.6 (naphthyl $C^{1/5}$ or $C^{9/10}$); 130.9 (naphthyl $C^{9/10}$ or $C^{1/5}$); 126.3 (naphthyl $C^{2/6}$); 126.2 (naphthyl $C^{3/7}$); 123.8 (naphthyl $C^{4/8}$); 123.1 (triazole C^5); 120.5 (pyridyl C^5); 117.1 (pyridyl C^3); 104.4 (pyrazole C^4); 88.1 (galactose C^1); 78.4 (galactose C^4); 73.6 (galactose C^3); 69.7 (O-**C**H$_2$-CNR); 69.3 (galactose C^2); 68.4 (galactose C^5); 63.2 (pyridyl-**C**H$_2$O); 60.4 (galactose C^6); 53.3 (pyridyl-**C**H$_2$N).

High-resolution ES-MS: *m/z* observed, 989.3921. Calculated for $C_{48}H_{53}N_{12}O_{12}^{+}$ [M + H^+], 989.3900. Observed, 495.1985. Calculated for $C_{48}H_{54}N_{12}O_{12}^{2+}$ [M + $2H^+$], 495.1987.

ν_{max}/cm^{-1}: 3276br, 3140br, 2916w, 1611m, 1366m, 1327m, 1242m, 1122s, 1090s.

Synthesis of cubic cages

[Co_8($L^{15\text{-Gal-Ac}}$)$_{12}$(BF_4)$_{16}$] [$Co_8^{\text{Gal-Ac}}$] (32)

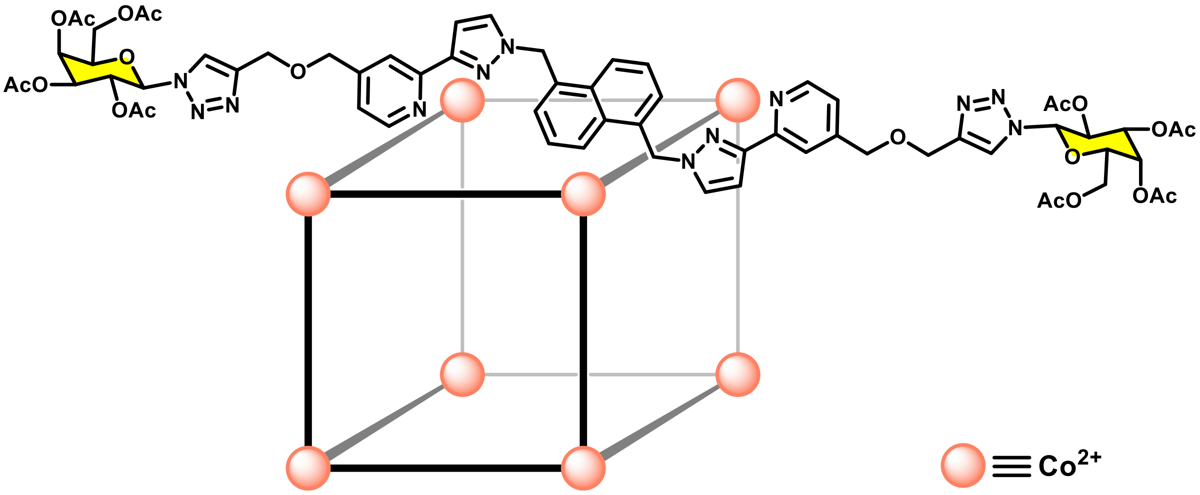

$L^{15\text{-Gal-Ac}}$ (52 mg, 39 µmol, 1.5 eq.) was added to a 50 mL RBF and dissolved in CH_2Cl_2 (5 mL). A solution of $Co(BF_4)_2$•$6H_2O$ (9 mg, 26 µmol, 1.0 eq.) in MeOH (5 mL) was then added and a precipitate was immediately formed. The solution was heated to 40 °C and stirred for 24 h. The solution was cooled to RT, centrifuged and the supernatant was washed sequentially with MeOH and CH_2Cl_2. Purification was then conducted on LH-20 Sephadex®, with CH_3CN as the eluent. Yield: 50 mg, 83%.

High resolution ES-MS: *m/z* 3465.6056 ([Co_8($L^{15\text{-Gal-Ac}}$)$_{12}$(BF_4)$_{11}$]$^{5+}$), 2873.8274 ([Co_8($L^{15\text{-Gal-Ac}}$)$_{12}$(BF_4)$_{10}$]$^{6+}$), 2450.8634 ([Co_8($L^{15\text{-Gal-Ac}}$)$_{12}$(BF_4)$_9$]$^{7+}$), 2133.6318 ([Co_8($L^{15\text{-Gal-Ac}}$)$_{12}$(BF_4)$_8$]$^{8+}$), 1887.0001 ([Co_8($L^{15\text{-Gal-Ac}}$)$_{12}$(BF_4)$_7$]$^{9+}$).

[$Co_8(L^{15\text{-}Glu\text{-}Ac})_{12}(BF_4)_{16}$] [$Co_8^{Glu\text{-}Ac}$] (33)

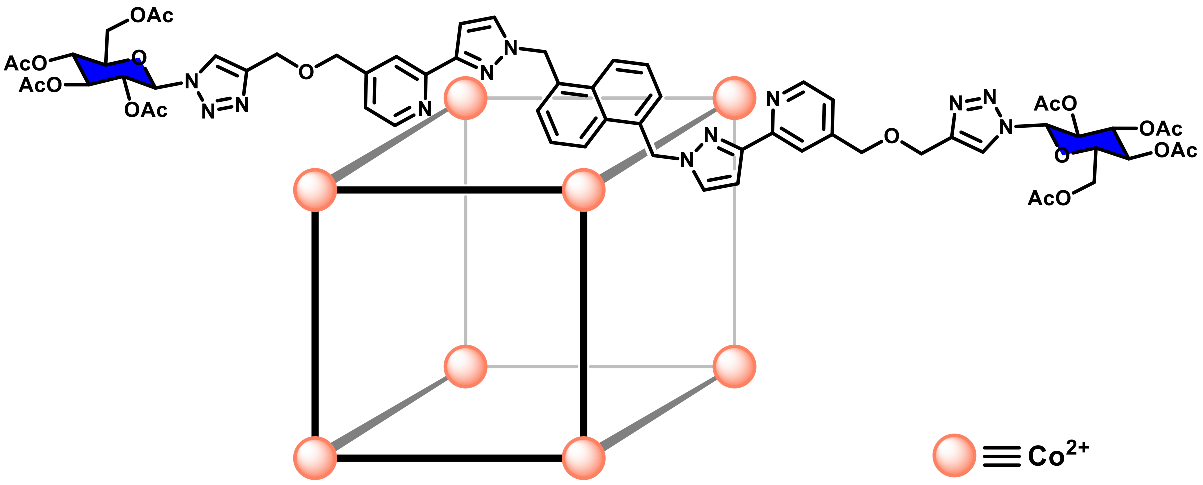

$L^{15\text{-}Glu\text{-}Ac}$ (84 mg, 63 μmol, 1.5 eq.) and $Co(BF_4)_2$•$6H_2O$ (14 mg, 42 μmol, 1.0 eq.) used with the same cage reaction conditions as **32**. Yield: 79 mg, 81%.

High resolution ES-MS: *m/z* 3465.8272 ([$Co_8(L^{15\text{-}Glu\text{-}Ac})_{12}(BF_4)_{11}]^{5+}$), 2873.6855 ([$Co_8(L^{15\text{-}Glu\text{-}Ac})_{12}(BF_4)_{10}]^{6+}$), 2450.7306 ([$Co_8(L^{15\text{-}Glu\text{-}Ac})_{12}(BF_4)_9]^{7+}$), 2133.6353 ([$Co_8(L^{15\text{-}Glu\text{-}Ac})_{12}(BF_4)_8]^{8+}$), 1886.8973 ([$Co_8(L^{15\text{-}Glu\text{-}Ac})_{12}(BF_4)_7]^{9+}$).

[Co_8($L^{15\text{-}Glu}$)$_{12}$(BF_4)$_{16}$] [Co_8^{Glu}] (34)

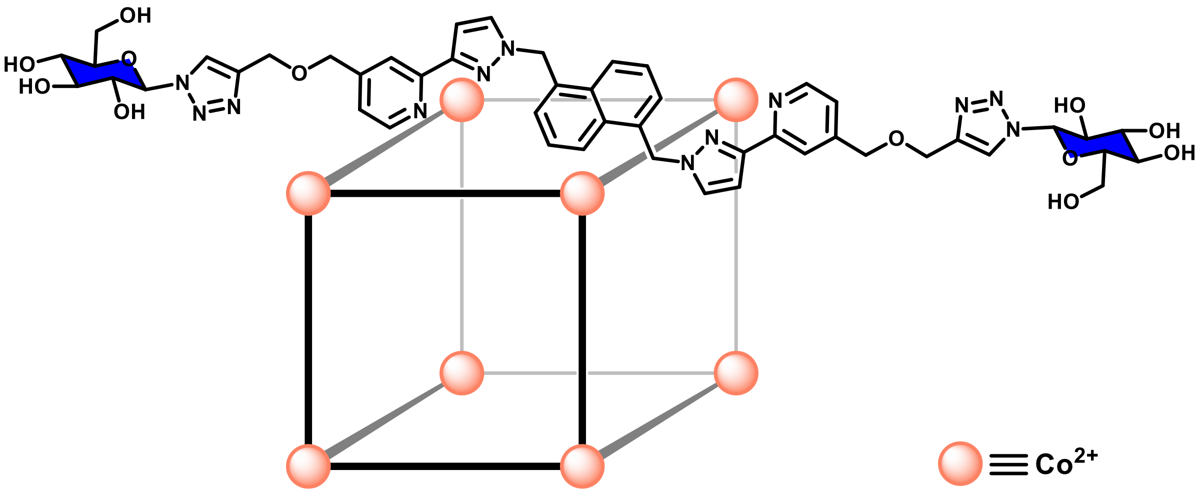

$L^{15\text{-}Glu}$ (66 mg, 67 μmol, 1.5 eq.) was added to a 50 mL RBF and dissolved in MeOH (5 mL); with gentle heating to encourage greater solubility. A solution of $Co(BF_4)_2$•$6H_2O$ (15 mg, 44 μmol, 1.0 eq.) in MeOH (5 mL) was then added and a precipitate was immediately formed. The solution was heated to 50 °C and stirred for 24 h. Once cooled to RT, the solvent was evaporated to leave a light pink solid, which was then re-dissolved in D_2O (~1 mL) for crude NMR analysis. The crude product was then immediately purified via G-50 Sephadex®, using H_2O as the eluent. Yield: 49 mg, 65.0%.

High resolution ES-MS: *m/z* 1874.2868 ([Co_8($L^{15\text{-}Glu}$)$_{12}$(BF_4)$_9$]$^{7+}$), 1629.1290 ([Co_8($L^{15\text{-}Glu}$)$_{12}$(BF_4)$_8$]$^{8+}$), 1438.4515 ([Co_8($L^{15\text{-}Glu}$)$_{12}$(BF_4)$_7$]$^{9+}$), 1286.0057 ([Co_8($L^{15\text{-}Glu}$)$_{12}$(BF_4)$_6$]$^{10+}$).

[$Co_8(L^{15\text{-}Gal})_{12}(BF_4)_{16}$] [$Co_8^{Gal}$] (35)

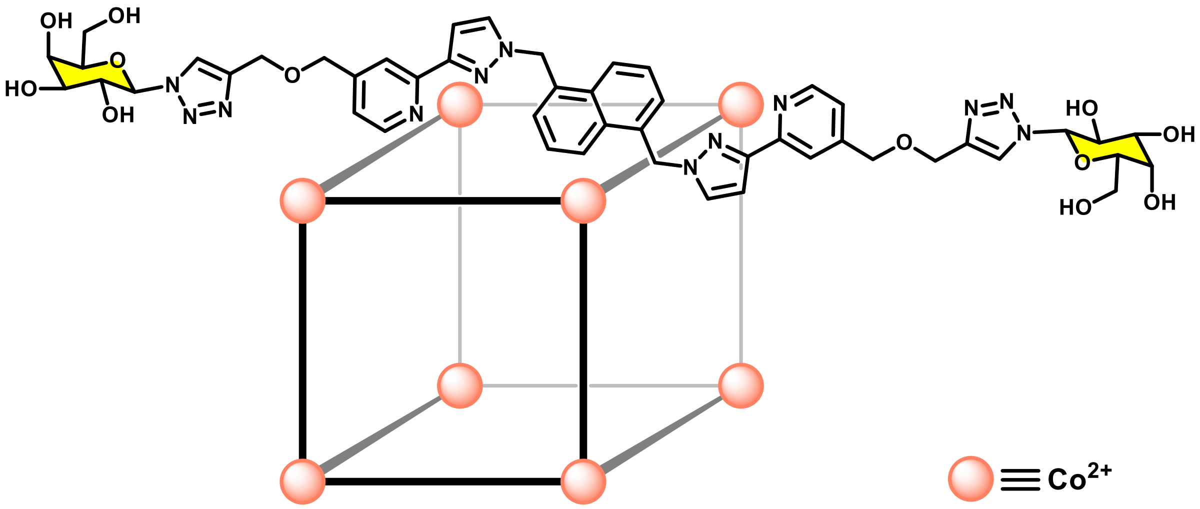

$L^{15\text{-}Gal}$ (134 mg, 0.135 mmol, 1.5 eq.) and $Co(BF_4)_2$•$6H_2O$ (31 mg, 90 µmol, 1.0 eq.) used with the same cage reaction conditions as **34**. Yield: 94mg, 61%.

High resolution ES-MS: *m/z* 1628.9975 ([$Co_8(L^{15\text{-}Gal})_{12}(BF_4)_8]^{8+}$), 1438.4469 ([$Co_8(L^{15\text{-}Gal})_{12}(BF_4)_7]^{9+}$), 1286.0058 ([$Co_8(L^{15\text{-}Gal})_{12}(BF_4)_6]^{10+}$), 1161.1881 [$Co_8(L^{15\text{-}Gal})_{12}(BF_4)_5]^{11+}$)

[Zn$_8$(L$^{15\text{-}Glu}$)$_{12}$(BF$_4$)$_{16}$] [Zn$_8$^{Glu}] (36)

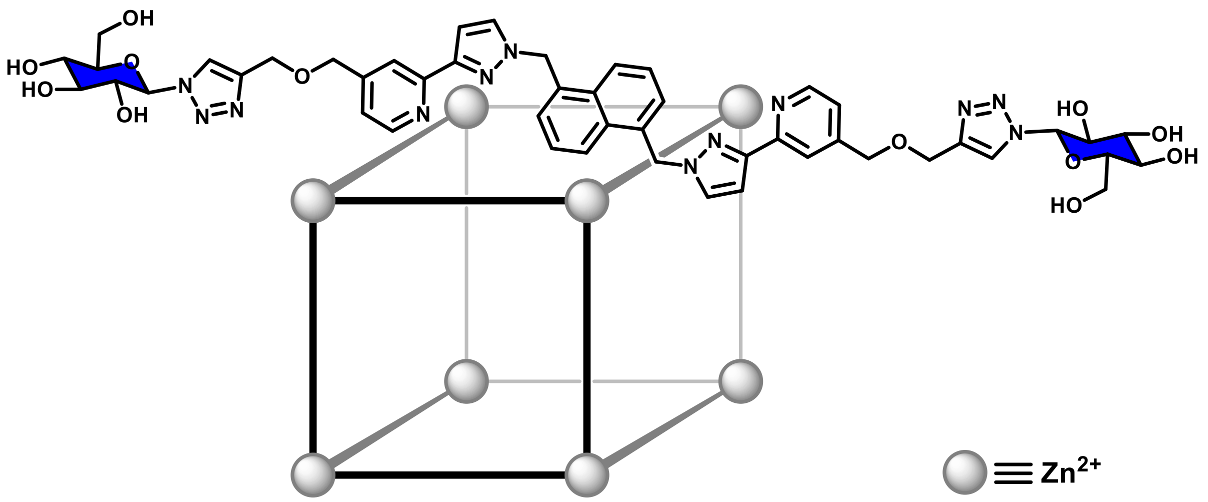

Prepared with Zn(BF$_4$)$_2$•xH$_2$O via the same procedure as the Co analogues **34** and **35** using ligand **30**. Yield: 62 mg, 93%.

High resolution ES-MS: *m/z* 1875.8543 ([Zn$_8$(L$^{15\text{-}Glu}$)$_{12}$(BF$_4$)$_9$]$^{7+}$), 1630.5005 ([Zn$_8$(L$^{15\text{-}Glu}$)$_{12}$(BF$_4$)$_8$]$^{8+}$), 1439.4473 ([Zn$_8$(L$^{15\text{-}Glu}$)$_{12}$(BF$_4$)$_7$]$^{9+}$).

[Zn$_8$(L$^{15\text{-}Gal}$)$_{12}$(BF$_4$)$_{16}$] [Zn$_8$^{Gal}] (37)

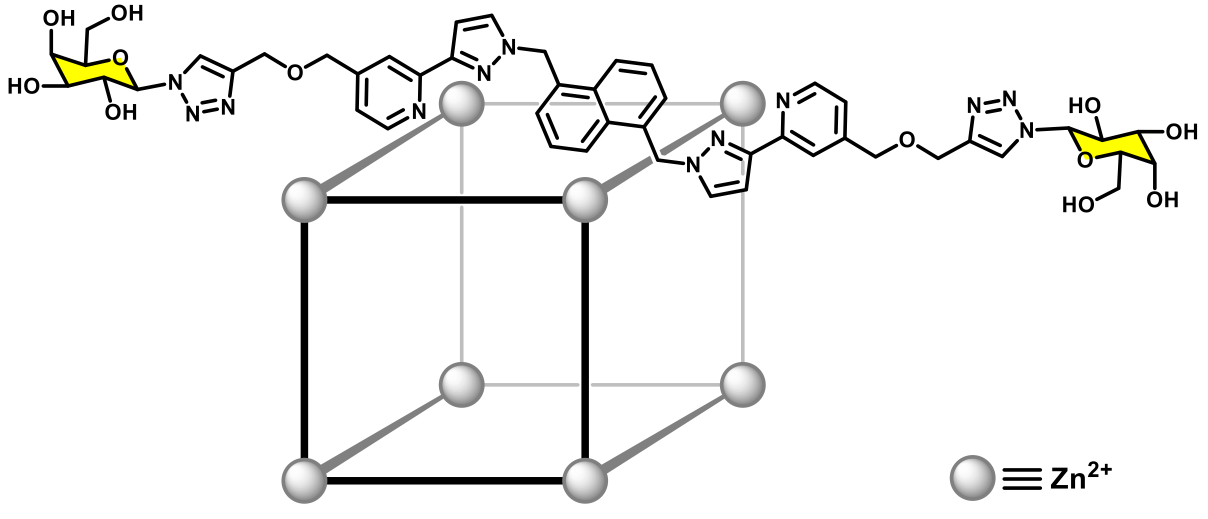

Prepared with Zn(BF$_4$)$_2$•xH$_2$O via the same procedure as the Co analogues **34** and **35** using ligand **31**. Yield: 48 mg, 72%.

High resolution ES-MS: *m/z* 1630.2397 ([Zn$_8$(L$^{15\text{-}Gal}$)$_{12}$(BF$_4$)$_8$]$^{8+}$), 1439.5514 ([Zn$_8$(L$^{15\text{-}Gal}$)$_{12}$(BF$_4$)$_7$]$^{9+}$), 1290.9992 ([Zn$_8$(L$^{15\text{-}Gal}$)$_{12}$(BF$_4$)$_6$]$^{10+}$).

Emission [H$_2$O, λ_{max}(nm), λ_{exc} 294 nm] 413.

[$Co_8(L^{15CC})_{12}(BF_4)_{16}$] [$Co_8^{CC}$] (38)

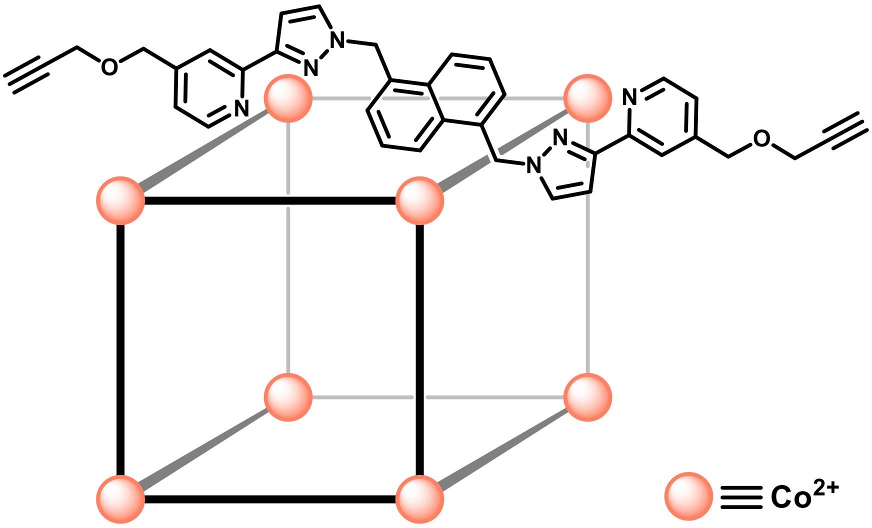

**For the purpose of comparing ^{1}H NMR spectra, this Co alkyne cage was also prepared via previously reported methods[9].*

Briefly, a glass vial was carefully charged with the alkyne-substituted ligand $\mathbf{L^{15CC}}$ (50 mg, 0.086 mmol, 1.5 equiv), $Co(BF_4)_2$•$6H_2O$ (19.6 mg, 0.058 mmol, 1.0 equiv) and MeOH (6 mL). This mixture was then placed within a Teflon liner and sealed within a solvothermal bomb apparatus, which was then heated to 120 °C at a rate of 0.1 °C min^{-1} and then sustained at this temperature over a 12 h period. The vessel was then cooled at a rate of 0.1 °C min^{-1} to RT. The resulting solution was then centrifuged to afford a salmon pink supernatant and washed sequentially with cold MeOH and CH_2Cl_2. The final solid was dried under high vacuum, dissolved in DMF-d_7 and a ^{1}H NMR spectrum was obtained. Yield: 55 mg, 87%.

High resolution ES-MS: *m/z* 1380.4030 ([$Co_8(L^{15CC})_{12}(BF_4)_6]^{6+}$), 1170.9168 ([$Co_8(L^{15CC})_{12}(BF_4)_7]^{7+}$), 1013.6673 ([$Co_8(L^{15CC})_{12}(BF_4)_8]^{8+}$)

Synthesis of tetrahedral cage ligands

$L^{23OTIPS}$ (39)

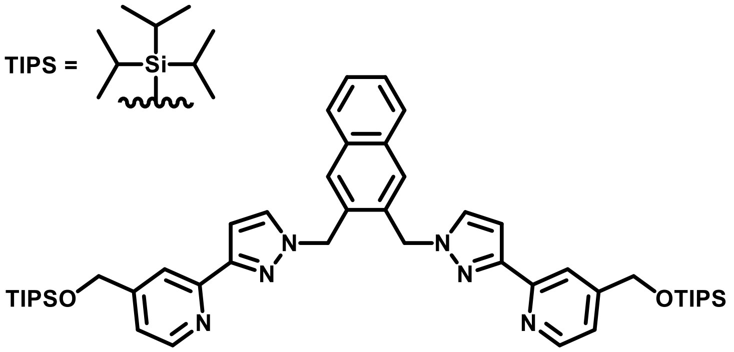

To a solution of 2-(1H-pyrazol-3-yl)-4-(((triisopropylsilyl)oxy)methyl)pyridine (3.0 g, 9.05 mmol, 2 eq.) in dry THF (300 mL) under N_2, was added NaH (60 wt. % dispersion in mineral oil, 0.724 g, 18.1 mmol, 4 eq.) and the mixture was stirred for 2 h at reflux. After 2 h, 2,3-bis(bromomethyl)naphthalene (1.43 g, 5.43 mmol, 1.2 eq.) and a catalytic amount of NaI was added and heating was continued for 36 h. The reaction was then quenched with MeOH at RT and the solvents were removed. The crude mixture was then redissolved in CH_2Cl_2 and filtered over celite. Solvents were then removed and the crude product was purified by an Al_2O_3 column (Brockmann activity III) using CH_2Cl_2/MeOH (98:2) as eluent to afford a yellow oil, which slowly becomes a yellow foam upon further removal of solvent. Yield: 3.05 g, 3.74 mmol, 83%.

^{1}H-NMR (300 MHz, **$CDCl_3$**, 298 K) δppm: 8.59 (2H, d, J = 5.2 Hz, pyridyl H^6); 7.89 (2H, s, pyridyl H^3); 7.78 (2H, dd, J = 6.2, 3.4 Hz, naphthyl $H^{5/8}$); 7.64 (2H, s, naphthyl $H^{1/4}$); 7.48 (2H, dd, J = 6.2, 3.3 Hz, napthyl $H^{6/7}$); 7.39 (2H, d, J = 2.3 Hz, pyrazole H^5); 7.29 (2H, d, J = 5.2 Hz, pyridyl H^5); 6.95 (2H, d, J = 2.3 Hz, pyrazole H^4); 5.56 (4H, s, pyridyl-C**H_2**N); 4.87 (4H, s, pyridyl-C**H_2**O), 1.23-1.01 (42H, m, OTIPS)

^{13}C-NMR (75 MHz, **$CDCl_3$**, 298 K) δppm: 152.2 (pyridyl C^2); 151.7 (pyrazole C^3); 149.2 (pyridyl C^6); 147.4 (pyridyl C^4); 133.1 (napthyl $C^{2/3}$); 132.3 (naphthyl $C^{5/10}$); 131.1 (pyrazole C^5); 129.2 (napthyl $C^{6/9}$); 127.7 (napthyl $C^{1/4}$); 126.9 (napthyl $C^{7/8}$); 120.8 (pyridyl C^5); 119.4 (pyridyl C^3); 105.2 (pyrazole C^4); 75.3 (**C**≡H), 63.8 (pyridyl-**C**H_2O), 54.1 (pyridyl-**C**H_2N), 18.0, 17.7, 12.3, 12.0 (OTIPS)

ESI-MS (+) *m/z* 815.4 $[M + H]^+$, 837.4 $[M + Na]^+$

L^{23OH} (40)

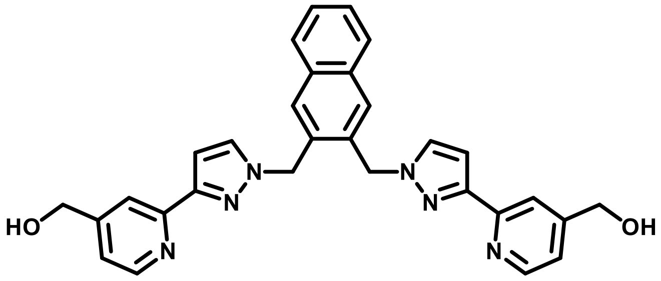

A solution of $\mathbf{L^{23OTIPS}}$ (3.05 g, 3.74 mmol) and tetrabutylammonium fluoride (1 M in dry THF, 3.25 mL, 11.22 mmol, 3 eq.) in dry THF (200 mL) was stirred at RT overnight. After 16 h, THF was removed and water was added to the residue, yielding a white solid precipitate. The bulk of the solid was collected via filtration, and any leftover solid collected with the addition of CH_2Cl_2 and sonication; followed by further filtration. Yield: 1.56 g, 3.10 mmol, 83%.

^{1}H-NMR (300 MHz, **DMSO-*d*$_6$**, 298 K) δppm: 8.50 (2H, d, J = 5.1 Hz, pyridyl H^6); 7.97 (2H, d, J = 2.0 Hz, pyrazole H^5), 7.93 (2H, s, pyridyl H^3); 7.82 (2H, dd, J = 6.1, 3.3 Hz, napthyl $H^{5/8}$), 7.58 (2H, s, naphthyl $H^{1/4}$); 7.48 (2H, dd, J = 6.3, 3.3 Hz, naphthyl $H^{6/7}$) 7.23 (2H, d, J = 4.9 Hz, pyridyl H^5); 6.93 (2H, d, J = 2.1 Hz, pyrazole H^4); 5.78 (4H, s, pyridyl-C**H_2**N); 4.56 (4H, s, pyridyl-C**H_2**O)

^{13}C-NMR (75 MHz, **DMSO-*d*$_6$**, 298 K) δppm: 152.9 (pyridyl C^2); 152.0 (pyrazole C^3); 149.4 (pyridyl C^6); 149.2 (pyridyl C^4); 134.1 (napthyl $C^{2/3}$); 133.2 (pyrazole C^5); 132.7 (naphthyl $C^{5/10}$); 128.3 (napthyl $C^{6/9}$); 127.7 (napthyl $C^{1/4}$); 126.8 (napthyl $C^{7/8}$); 120.7 (pyridyl C^5); 117.2 (pyridyl C^3); 105.2 (pyrazole C^4); 62.0 (pyridyl-**C**H_2O); 53.3 (pyridyl-**C**H_2N).

ESI-MS (+) *m/z* 503.2 $[M + H]^+$, 525.2 $[M + Na]^+$

ν_{max}/cm^{-1}: 3233br, 3145w, 2960w, 2926w, 2868w, 1608m, 1357m, 1049m, 755s.

L^{23CC} (41)

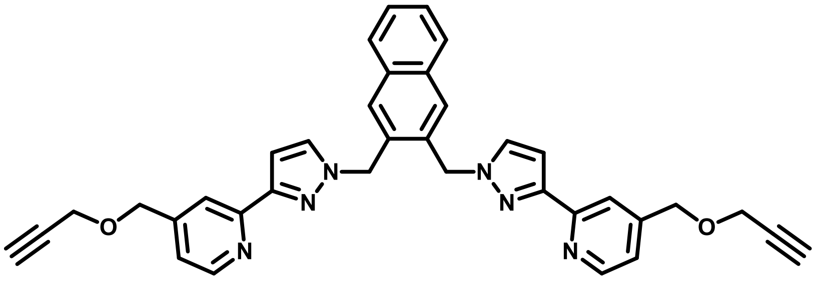

NaH (0.54 g, 13.43 mmol, 60 wt. % dispersion in mineral oil, 5 eq.) and 15-crown-5 ether (1.06 mL, 5.38 mmol, 2 eq.) were added to a flame dried two neck RBF charged with dry THF (50 mL): the mixture was stirred for 30 mins under N_2. To this suspension, a solution of **L^{23OH}** (1.35 g, 2.69 mmol) in dry THF (250 mL) was transferred via cannula and the resulting suspension was refluxed for 2 h before the addition of propargyl bromide (0.90 mL, 8.07 mmol, 80 wt. % in toluene, 3 eq.) with an instant colour change to brown observed. The mixture was refluxed for 72 h, with a darker brown colour developing over time. Once cooled to room temperature, the reaction was quenched with the addition of MeOH (50 mL) and the solvents evaporated to give a brown oil. The mineral oil was removed by filtration through a celite®/CH_2Cl_2 plug. The crude product was then purified through an Al_2O_3 column (Brockmann activity III) using CH_2Cl_2/MeOH (98:2) as eluent to remove unreacted starting material and a silica column using CH_2Cl_2/MeOH (96:4) as eluent to remove crown ether impurities. Yield: 1.16 g, 2.02 mmol, 75% (red solid).

^{1}H-NMR (300 MHz, **$CDCl_3$**, 298 K) *δ*ppm: 8.60 (2H, d, J = 5.1 Hz, pyridyl H^6); 7.93 (2H, m, pyridyl H^3); 7.77 (2H, dd, J = 6.2, 3.3 Hz, naphthyl H$^{5/8}$); 7.60 (2H, s, naphthyl H$^{1/4}$); 7.48 (2H, dd, J = 6.3, 3.4 Hz, napthyl H$^{6/7}$); 7.38 (2H, d, J = 2.4 Hz, pyrazole H^5); 7.22 (2H, d, J = 5.1, 1.0 Hz, pyridyl H^5); 6.96 (2H, d, J = 2.3 Hz, pyrazole H^4); 5.57 (4H, s, pyridyl-C**H_2**N); 4.65 (4H, s, pyridyl-C**H_2**O); 4.24 (4H, d, J = 2.4 Hz, CH_2-O-C**H_2**-C≡C); 2.48 (2H, t, J = 2.3 Hz, CH_2-C≡**H**)

^{13}C-NMR (100 MHz, **$CDCl_3$**, 298 K) *δ*ppm: 152.2 (pyridyl C^2); 151.7 (pyrazole C^3); 149.4 (pyridyl C^6); 147.4 (pyridyl C^4); 133.0 (napthyl C$^{2/3}$); 132.2 (naphthyl C$^{5/10}$); 131.2 (pyrazole C^5); 129.0 (napthyl C$^{6/9}$); 127.7 (napthyl C$^{1/4}$); 126.9 (napthyl C$^{7/8}$); 120.8 (pyridyl C^5); 118.4 (pyridyl C^3); 105.3 (pyrazole C^4); 75.3 (**C**≡H), 69.9 (pyridyl-**C**H_2O), 57.9 (CH_2-O-**C**H_2-C≡C), 54.0 (pyridyl-**C**H_2N).

High-resolution ES-MS: *m/z* observed, 579.2500. Calculated for $C_{36}H_{31}N_6O_2^+$ [*M* + H$^+$], 579.2503. Observed, 601.2315. Calculated for $C_{36}H_{30}N_6O_2Na^+$ [*M* + Na$^+$], 601.2322.

ν_{max}/cm^{-1}: 3284w, 2920w, 2851w, 2113w, 1606m, 1559m, 1087m, 765m, 752m.

$L^{23\text{-Glu-Ac}}$ (42)

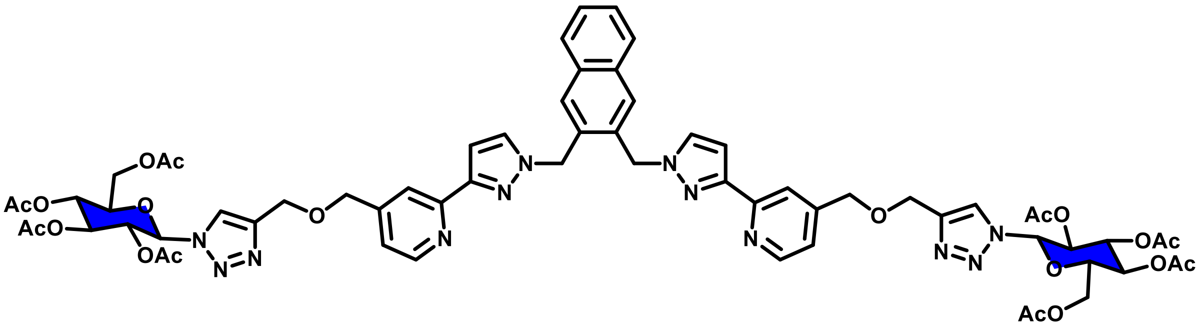

Prepared via the same CuAAC procedure as the cubic cage ligands: 250 mg of **L^{23CC}** used. Yield: 390 mg, 0.29 mmol, 68%.

^{1}H-NMR (400 MHz, **CDCl$_3$**, 298 K) *δ*ppm: 8.59 (2H, d, J = 4.8 Hz, pyridyl H^6); 7.91 (2H, s, pyridyl H^3); 7.85 (2H, s, triazole H^5); 7.77 (2H, dd, J = 6.2, 3.3 Hz, naphthyl H$^{5/8}$); 7.61 (2H, s, naphthyl H$^{1/4}$); 7.48 (2H, dd, J = 6.3, 3.3 Hz, napthyl H$^{6/7}$); 7.37 (2H, s, pyrazole H^5); 7.23 (2H, d, J = 4.7 Hz, pyridyl H^5); 6.96 (2H, s, pyrazole H^4); 5.88 (2H, d, J = 8.5 Hz, glucose H^1); 5.57 (4H, s, pyridyl-C**H$_2$**N); 5.46-5.39 (4H, m, glucose H^{2+3}); 5.23 (2H, t, J = 9.6 Hz, glucose H^4); 4.72 (4H, s, pyridyl-C**H$_2$**O); 4.61 (4H, s, O-C**H$_2$**-CNR); 4.29 (2H, dd, J = 12.8, 4.9 Hz, glucose H^{6b}); 4.12 (2H, d, J = 12.3 Hz, glucose H^{6a}); 3.99 (2H, dd, J = 10.4, 3.9 Hz, glucose H^5); 2.05 (6H, s, methyl C**H$_3$**); 2.04 (6H, s, methyl C**H$_3$**); 2.01 (6H, s, methyl C**H$_3$**); 1.84 (6H, s, methyl C**H$_3$**).

^{13}C-NMR (100 MHz, **CDCl$_3$**, 298 K) *δ*ppm: 170.5, 169.9, 169.4, 168.9 (**C**OCH$_3$); 152.0 (pyridyl C^2); 145.4 (triazole C^4); 133.0 (napthyl C$^{2/3}$); 132.1 (naphthyl C$^{5/10}$); 131.2 (pyrazole C^5); 129.1 (napthyl C$^{6/9}$); 127.7 (napthyl C$^{1/4}$); 126.9 (napthyl C$^{7/8}$); 121.2 (triazole C^5); 120.8 (pyridyl C^5); 118.4 (pyridyl C^3); 105.4 (pyrazole C^4); 85.8 (glucose C^1); 75.1 (glucose C^5); 72.6 (glucose C^3); 70.7 (O-CH$_2$-CNR); 70.4 (glucose C^2); 67.7 (glucose C^4); 63.9 (pyridyl-**C**H$_2$O); 61.5 (glucose C^6); 54.1 (pyridyl-**C**H$_2$N); 20.7, 20.5, 20.5, 20.2 (CO**C**H$_3$).

ESI-MS (+) *m/z* 1325.4 [*M* + H]$^+$, 1347.3 [*M* + Na]$^+$

ν_{max}/cm^{-1}: 2960w, 1745s, 1366m, 1211s, 1092m, 1035s.

L$^{23\text{-Gal-Ac}}$ (43)

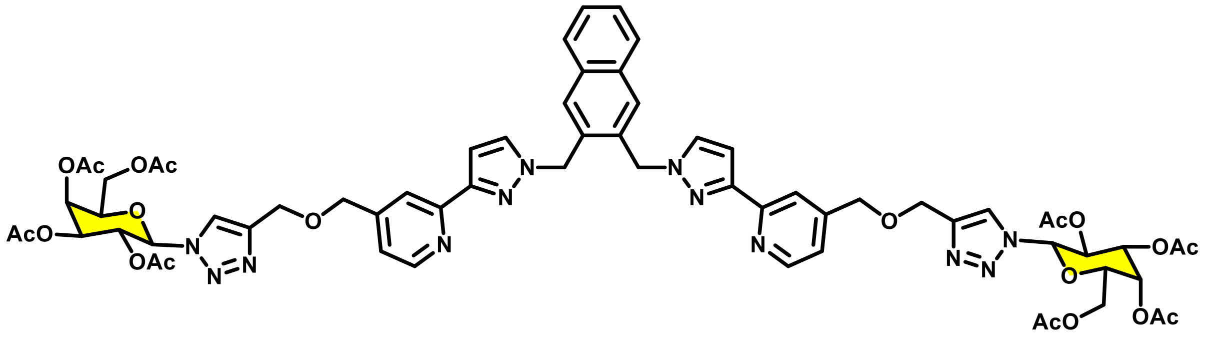

This was prepared via the same CuAAC procedure as used for the cubic cage ligands: 250 mg of **L^{23CC}** used. Yield: 416 mg, 0.31 mmol, 73%.

^{1}H-NMR (400 MHz, **$CDCl_3$**, 298 K) δppm: 8.60 (2H, d, J = 4.8 Hz, pyridyl H^{6}); 7.92 (2H, s, pyridyl H^{3}); 7.88 (2H, s, triazole H^{5}); 7.77 (2H, dd, J = 6.1, 3.2 Hz, naphthyl H$^{5/8}$); 7.60 (2H, s, naphthyl H$^{1/4}$); 7.48 (2H, dd, J = 6.1, 3.4 Hz, napthyl H$^{6/7}$); 7.37 (2H, s, pyrazole H^{5}); 7.25 (2H, d, J = 5.6 Hz, pyridyl H^{5}); 6.97 (2H, s, pyrazole H^{4}); 5.84 (2H, d, J = 9.4 Hz, galactose H^{1}); 5.56 (4H, s, pyridyl-C**H_2**N); 5.54 (4H, t, J = 9.8 Hz, galactose H^{2+4}); 5.24 (2H, dd, J = 10.3, 3.2 Hz, galactose H^{3}); 4.74 (4H, s, pyridyl-C**H_2**O); 4.62 (4H, s, O-C**H_2**-CNR); 4.24-4.10 (6H, m, galactose H$^{6a+6b+5}$); 2.19 (6H, s, methyl C**H_3**); 2.02 (6H, s, methyl C**H_3**); 1.99 (6H, s, methyl C**H_3**); 1.86 (6H, s, methyl C**H_3**).

^{13}C-NMR (100 MHz, **$CDCl_3$**, 298 K) δppm: 170.3, 169.9, 169.8, 169.1 (**C**OCH$_3$); 152.0 (pyridyl C^{2}); 151.5 (pyrazole C^{3}); 149.3 (pyridyl C^{6}); 145.3 (triazole C^{4}); 133.0 (napthyl C$^{2/3}$); 132.1 (naphthyl C$^{5/10}$); 131.2 (pyrazole C^{5}); 129.1 (napthyl C$^{6/9}$); 127.7 (napthyl C$^{1/4}$); 126.9 (napthyl C$^{7/8}$); 121.3 (triazole C^{5}); 120.8 (pyridyl C^{5}); 118.4 (pyridyl C^{3}); 105.5 (pyrazole C^{4}); 86.3 (galactose C^{1}); 74.1 (galactose C^{5}); 70.7 (O-**C**H$_2$-CNR + galactose C^{3}); 68.0 (galactose C^{2}); 66.9 (galactose C^{4}); 64.0 (pyridyl-**C**H$_2$O); 61.2 (galactose C^{6}); 54.1 (pyridyl-**C**H$_2$N); 20.7, 20.6, 20.5, 20.3 (CO**C**H$_3$).

ESI-MS (+) *m/z* 1325.4 [M + H]$^{+}$, 1347.3 [M + Na]$^{+}$

ν_{max}/cm^{-1}: 2962w, 1745s, 1367m, 1210s, 1089m, 1042s.

L$^{23\text{-Gal}}$ (44)

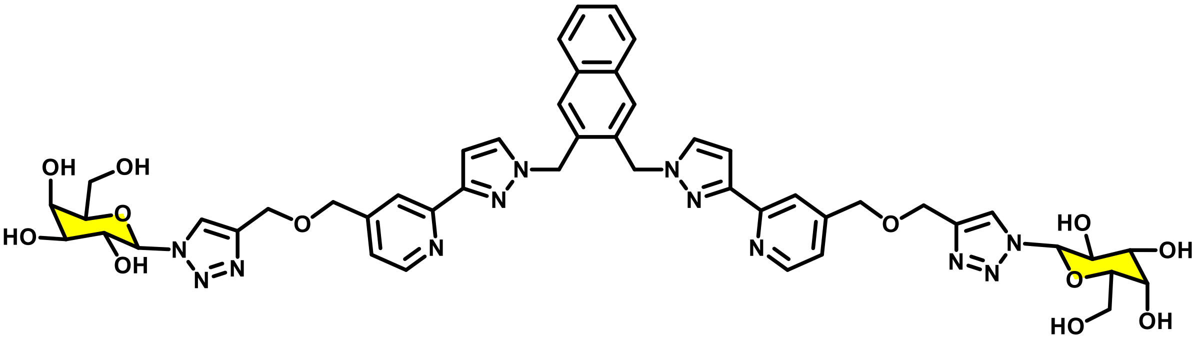

Prepared via the same deprotection procedure as used for the cubic cage ligands: 115 mg of **L$^{23\text{-Gal-Ac}}$** used. Yield: 80 mg, 81 μmol, 93%.

^{1}H-NMR (400 MHz, **DMSO-*d*$_6$**, 298 K) *δ*ppm: 8.55 (2H, d, J = 4.8 Hz, pyridyl H^6); 8.31 (2H, s, triazole H^5); 7.97 (2H, s, pyrazole H^5), 7.90 (2H, s, pyridyl H^3); 7.83 (2H, s, napthyl H$^{5/8}$), 7.57 (2H, s, naphthyl H$^{1/4}$); 7.49 (2H, s, naphthyl H$^{6/7}$) 7.28 (2H, d, J = 5.6 Hz, pyridyl H^5); 6.95 (2H, s, pyrazole H^4); 5.78 (4H, s, pyridyl-C**H$_2$**N); 5.49 (2H, d, J = 8.8 Hz, galactose H^1); 4.85 (8H, brs, galactose-OH); 4.65 (4H, s, pyridyl-C**H$_2$**O); 4.63 (4H, s, O-C**H$_2$**-CNR); 4.06 (2H, t, J = 9.2 Hz, galactose H^2); 3.78 (2H, s, galactose H^5); 3.72 (2H, t, J = 5.2 Hz, galactose H^4); 3.58-3.47 (6H, m, galactose H$^{3+6a+6b}$).

^{13}C-NMR (100 MHz, **DMSO-*d*$_6$**, 298 K) *δ*ppm: 152.2 (pyridyl C^2); 151.8 (pyrazole C^3); 149.7 (pyridyl C^6); 148.5 (pyridyl C^4); 144.0 (triazole C^4); 134.1 (napthyl C$^{2/3}$); 133.0 (pyrazole C^5); 132.7 (naphthyl C$^{5/10}$); 128.0 (napthyl C$^{6/9}$); 127.9 (napthyl C$^{1/4}$); 127.1 (napthyl C$^{7/8}$); 123.6 (triazole C^5); 121.1 (pyridyl C^5); 117.7 (pyridyl C^3); 105.1 (pyrazole C^4); 88.6 (galactose C^1); 78.9 (galactose C^4); 74.1 (galactose C^3); 70.2 (O-**C**H$_2$-CNR); 69.8 (galactose C^2); 68.9 (galactose C^5); 63.7 (pyridyl-**C**H$_2$O); 60.9 (galactose C^6); 53.3 (pyridyl-**C**H$_2$N).

High-resolution ES-MS: *m/z* observed, 989.3695. Calculated for $C_{48}H_{53}N_{12}O_{12}^{+}$ [*M* + H$^+$], 989.3900.

L$^{23\text{-Glu}}$ (45)

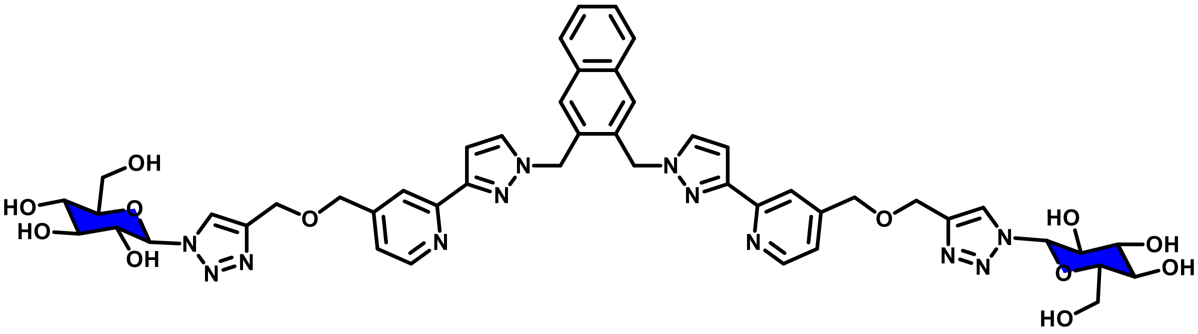

Prepared via the same deprotection procedure as used for the cubic cage ligands: 115 mg of **L$^{23\text{-Glu-Ac}}$** used. Yield: 79 mg, 80 μmol, 92%.

^{1}H-NMR (400 MHz, **DMSO-d_6**, 298 K) δppm: 8.54 (2H, d, J = 4.8 Hz, pyridyl H^6); 8.37 (2H, s, triazole H^5); 7.96 (2H, d, J = 1.3 Hz, pyrazole H^5), 7.89 (2H, s, pyridyl H^3); 7.83 (2H, dd, J = 6.0, 3.0 Hz, napthyl H$^{5/8}$), 7.56 (2H, s, naphthyl H$^{1/4}$); 7.49 (2H, dd, J = 6.0, 3.0 Hz, naphthyl H$^{6/7}$) 7.27 (2H, d, J = 5.0 Hz, pyridyl H^5); 6.93 (2H, d, J = 1.6 Hz, pyrazole H^4); 5.77 (4H, s, pyridyl-C**H$_2$**N); 5.52 (2H, d, J = 9.4 Hz, glucose H^1); 4.63 (8H, s, pyridyl-C**H$_2$**O and O-C**H$_2$**-CNR); 4.36 (8H, brs, glucose-OH); 3.77 (2H, t, J = 9.2 Hz, glucose H^2); 3.69 (2H, d, J = 10.0 Hz, glucose H^6); 3.51-3.38 (6H, m, glucose H^{3+4+6}); 3.24 (2H, t, J = 9.0 Hz, glucose H^5).

^{13}C-NMR (100 MHz, **DMSO-d_6**, 298 K) δppm: 152.2 (pyridyl C^2); 151.8 (pyrazole C^3); 149.8 (pyridyl C^6); 148.5 (pyridyl C^4); 143.9 (triazole C^4); 134.0 (napthyl C$^{2/3}$); 133.0 (pyrazole C^5); 132.7 (naphthyl C$^{5/10}$); 128.0 (napthyl C$^{6/9}$); 127.9 (napthyl C$^{1/4}$); 127.1 (napthyl C$^{7/8}$); 123.9 (triazole C^5); 121.1 (pyridyl C^5); 117.7 (pyridyl C^3); 105.1 (pyrazole C^4); 88.0 (glucose C^1); 80.4 (glucose C^4); 77.4 (glucose C^3); 72.5 (glucose C^2); 70.2 (O-**C**H$_2$-CNR); 70.0 (glucose C^5); 63.7 (pyridyl-**C**H$_2$O); 61.2 (glucose C^6); 53.3 (pyridyl-**C**H$_2$N)

High-resolution ES-MS: *m/z* observed, 989.3705. Calculated for $C_{48}H_{53}N_{12}O_{12}^+$ [M + H^+], 989.3900.

Synthesis of tetrahedral cages

[$Co_4(L^{23\text{-}Glu})_6(BF_4)_8$] [$Co_4^{Glu}$] (46)

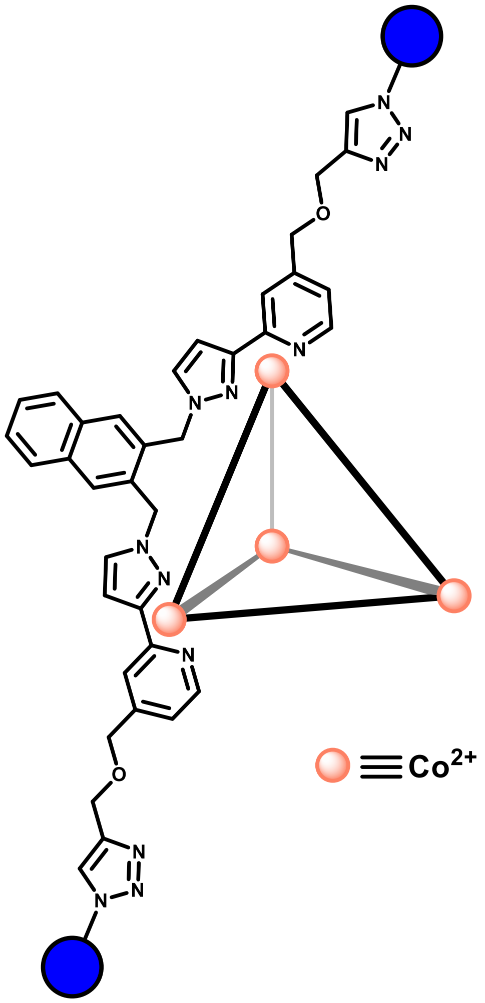

Prepared via the same procedure for the deprotected glycan cubic cages with a M:L ratio of 2:3 using ligand **45**. Yield: 99 mg, 79%.

High resolution ES-MS: *m/z* 1629.0092 ($[Co_4(L^{23\text{-}Glu})_6(BF_4)_4]^{4+}$), 1285.8069 ($[Co_4(L^{23\text{-}Glu})_6(BF_4)_3]^{5+}$), 1057.1715 ($[Co_4(L^{23\text{-}Glu})_6(BF_4)_2]^{6+}$), 893.7179 ($[Co_4(L^{23\text{-}Glu})_6(BF_4)]^{7+}$)

[Co$_4$(L$^{23\text{-Gal}}$)$_6$(BF$_4$)$_8$] [Co$_4^{Gal}$] (47)

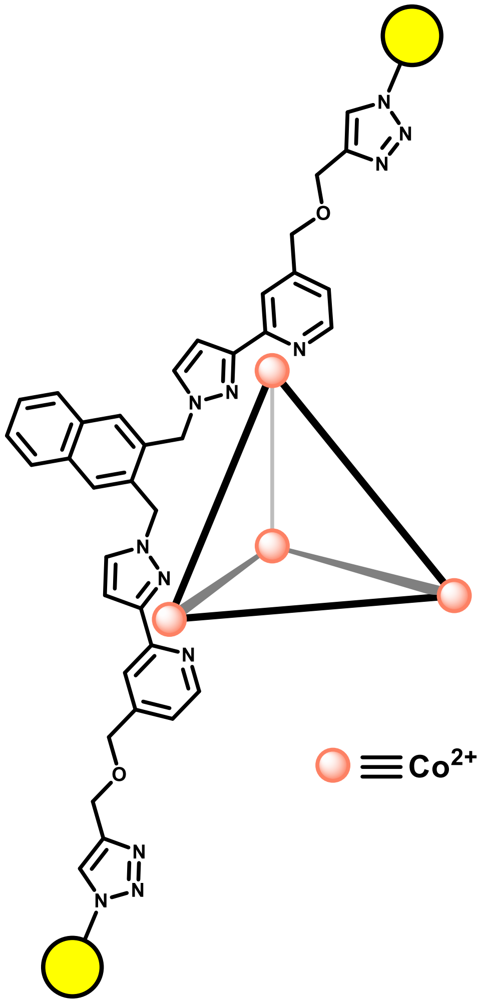

Prepared via the same procedure used for the deprotected glycan cubic cages with a M:L ratio of 2:3 using ligand **44**. Yield: 109 mg, 87%.

High resolution ES-MS: *m/z* 1629.0052 ([Co$_4$(L$^{23\text{-Gal}}$)$_6$(BF$_4$)$_4$]$^{4+}$), 1285.8054 ([Co$_4$(L$^{23\text{-Gal}}$)$_6$(BF$_4$)$_3$]$^{5+}$), 1057.1724 ([Co$_4$(L$^{23\text{-Gal}}$)$_6$(BF$_4$)$_2$]$^{6+}$), 893.7188 ([Co$_4$(L$^{23\text{-Gal}}$)$_6$(BF$_4$)]$^{7+}$)

[$Zn_4(L^{23\text{-}Glu})_6(BF_4)_8$] [$Zn_4^{Glu}$] (48)

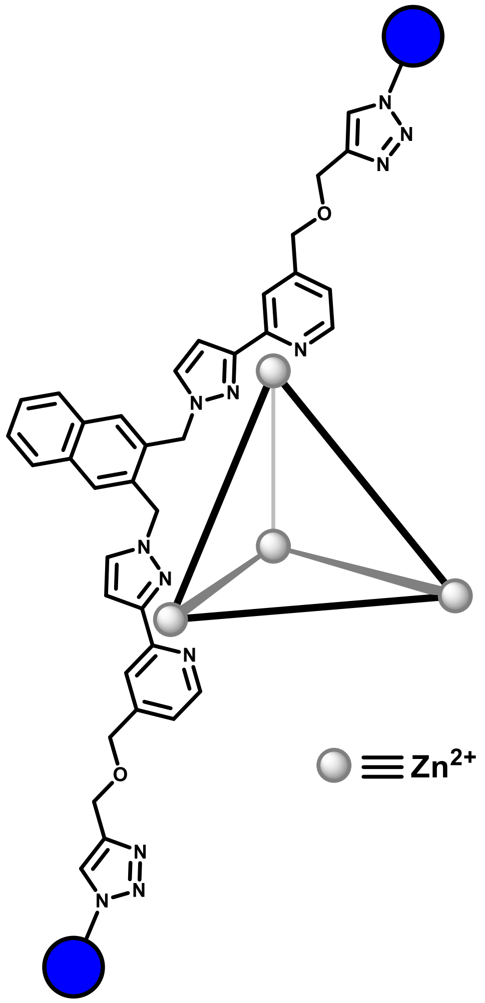

Prepared with $Zn(BF_4)_2{\bullet}xH_2O$ via the same procedure as used for the Co analogues **46** and **47** using ligand **45**. Yield: 60 mg, 82%.

High resolution ES-MS: *m/z* 1291.2037 ($[Zn_4(L^{23\text{-}Glu})_6(BF_4)_3]^{5+}$), 1061.3346 ($[Zn_4(L^{23\text{-}Glu})_6(BF_4)_2]^{6+}$)

[$Zn_4(L^{23\text{-}Gal})_6(BF_4)_8$] [$Zn_4^{Gal}$] (49)

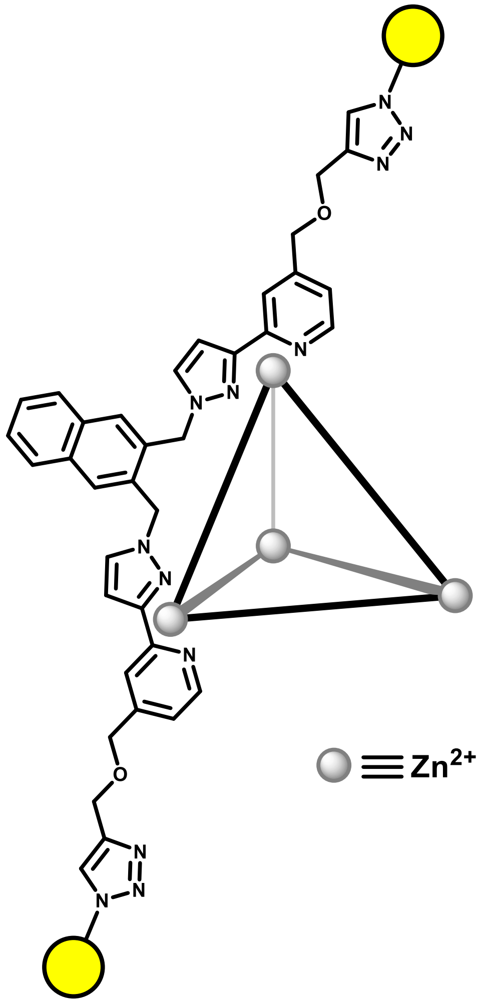

Prepared with $Zn(BF_4)_2$•xH_2O via the same procedure as the Co analogues **46** and **47** using ligand **44**. Yield: 88 mg, 78%.

High resolution ES-MS: *m/z* 1291.2013 ($[Zn_4(L^{23\text{-}Gal})_6(BF_4)_3]^{5+}$), 1061.3350 ($[Zn_4(L^{23\text{-}Gal})_6(BF_4)_2]^{6+}$)

Emission [H_2O, λ_{max}(nm), λ_{exc} 286 nm] 423.

[$Co_4(L^{23CC})_6(BF_4)_8$] [Co_4^{CC}] (50)

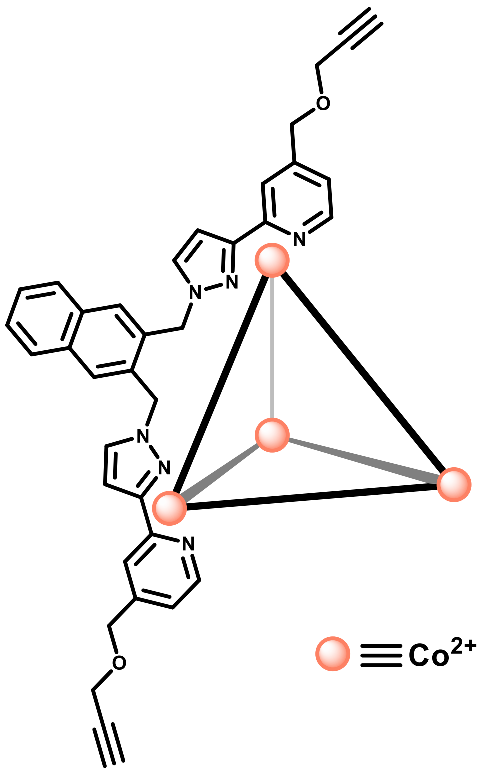

**For the purpose of comparing ^{1}H NMR spectra, this Co alkyne tetrahedral cage was also prepared via a previously reported method[9]. ESI-MS data were not collected and crystals were not grown.*

A glass vial was carefully charged with the alkyne-substituted ligand $\mathbf{L^{23CC}}$ (50 mg, 0.086 mmol, 1.5 equiv), $Co(BF_4)_2$•$6H_2O$ (19.6 mg, 0.058 mmol, 1.0 equiv) and MeOH (6 mL). This mixture was then placed within a Teflon liner and sealed within a solvothermal bomb apparatus, which was then heated to 120 °C at a rate of 0.1 °C min-1 and then sustained at this temperature over a 12 h period. The vessel was then cooled at a rate of 0.1 °C min^{-1} to RT. The resulting solution was then centrifuged to afford a crude black supernatant and a brown solute which was separated. The solution was concentrated *in vacuo* to afford a brown solid that was dried under high vacuum, dissolved in CD_3CN and a ^{1}H NMR spectrum was obtained. Yield: 58 mg, 92%.

Preparation of 1-azido-3’-sialyllactose and 1-azido-6’-sialyllactose

Acetylation protection

To an ice-cold solution of 3’-sialyllactose sodium salt (**24**) (200 mg, 0.31 mmol) in Ac_2O (5 mL) under N_2, a cold solution of conc. H_2SO_4 (0.5 mL) in Ac_2O (5 mL) was slowly added dropwise under stirring.[10] The reaction mixture was warmed to RT, then stirred at 40 °C for 1.5 h, then poured slowly with stirring into crushed ice (100 mL) and stirring continued for 2 h*. CH_2Cl_2 (60 mL) was added, organic extracted (3 x 30 mL), washed with H_2O (100 mL) and concentrated to give a white foam, which was confirmed by ESI-MS. Yield: 264 mg, 0.25 mmol, 79%.

***m/z* (ES$^-$)** 1094.2 [M - H]$^-$

Longer than 2 h gives an acidic red solution that upon extraction does not yield the desired product. The above procedure was also used with 6’-sialyllactose sodium salt (25**).*

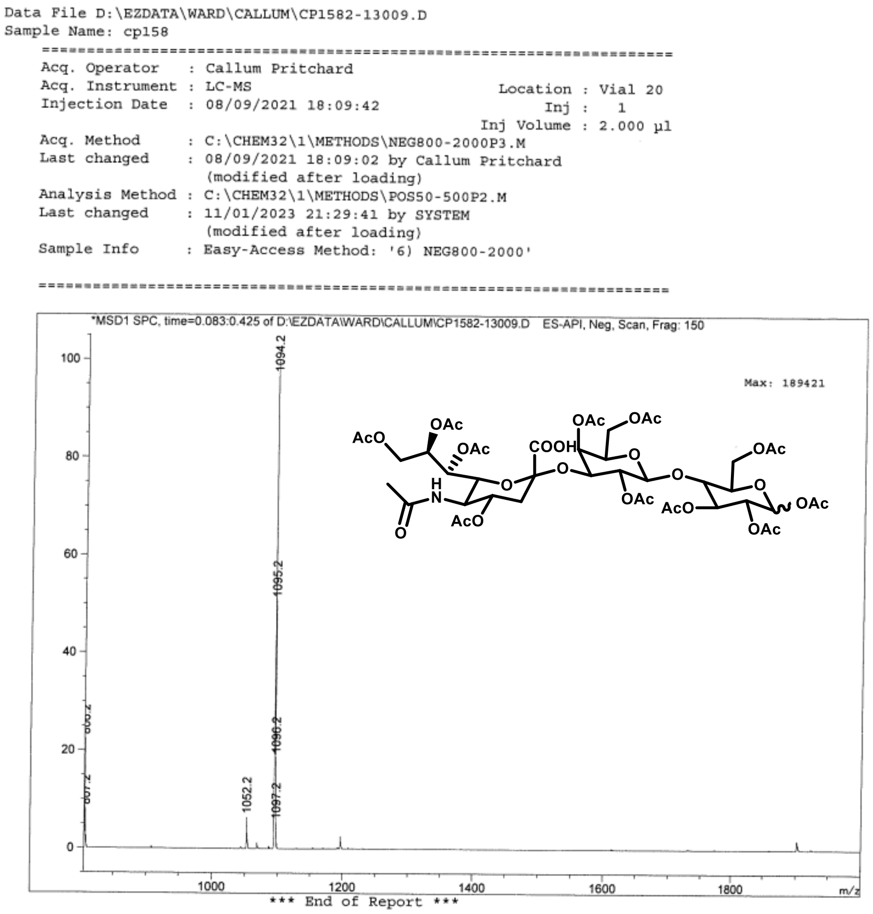

Figure S47. LR-ESI-MS spectrum of **3’-sialyllactose (Ac) (51)**

Bromination

To an oven-dried 50 mL two-neck RBF containing 3'-sialyllactose (Ac) (125 mg, 0.11 mmol) was added HBr solution (2 mL, 33 wt. % in acetic acid) at 0 °C and under N_2.[4] The resulting solution was allowed to warm back up to RT and stirred for 3 h. The reaction was then diluted with EtOAc (5 mL), cooled to 0 °C, and the solution neutralised carefully with 10 % aq. NaOH and sat. $NaHCO_3$. The mixture was then extracted with EtOAc (3 x 15 mL) and these organic extracts were dried with $MgSO_4$, filtered and evaporated under reduced pressure to leave a clear syrup; which was confirmed by ESI-MS. Yield: 90 mg, 80 µmol, 71%.

***m/z* (ES$^-$)** 1114.1 and 1116.1 [*M* - H]$^-$

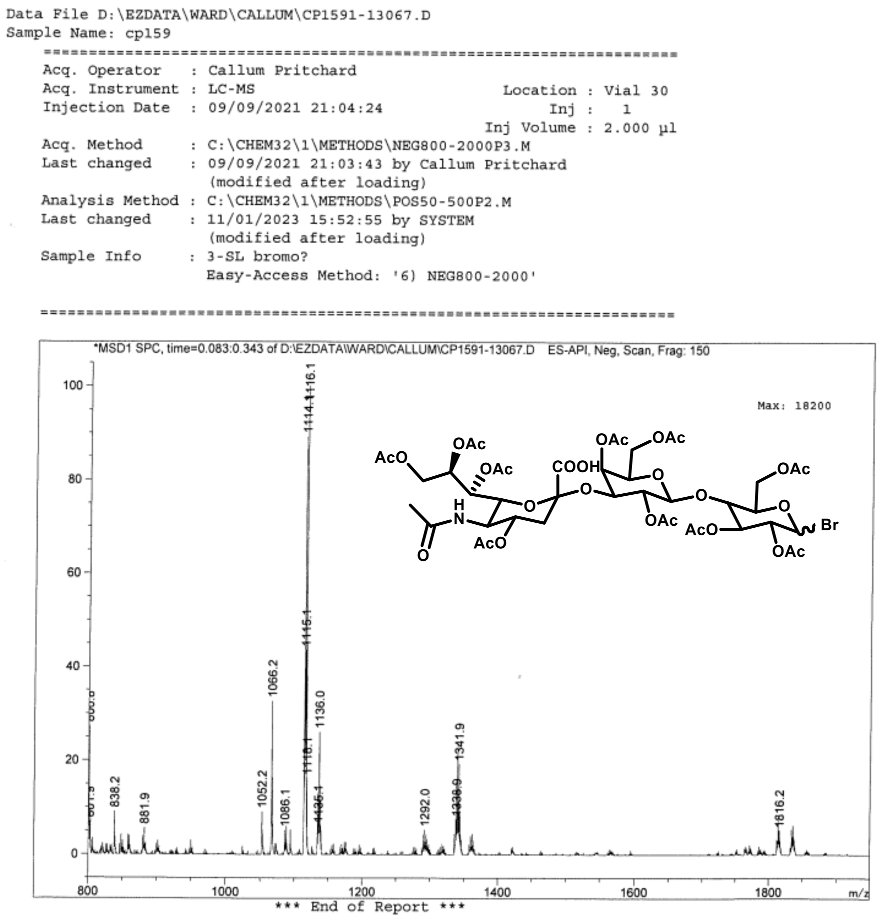

Figure S48. LR-ESI-MS spectrum of **1-bromo-3'-sialyllactose (Ac)** (**53**)

Azido conversion

1-Bromo-3'-sialyllactose (Ac) (62 mg, 56 μmol, 1 eq.) was added to a 50 mL two-neck RBF equipped with a rubber septum and magnetic stirrer bar and dissolved into a mixture of acetone/H_2O (5:1, 5 mL, 1 mL) to give a clear solution. NaN_3 (19 mg, 0.282 mmol, 5 eq.) was then added via a cool glass funnel and the resulting solution was left to stir for 24 h at RT[5,11].

Acetone was the carefully removed under low pressure at RT and the residual off-white slush was then portioned between H_2O and EtOAc (50:50, 20 mL). The organic layer was removed and the aqueous layer extracted with EtOAc (3 x 10 mL). Combined organic extracts were dried over $MgSO_4$ and concentrated *in vacuo* at 40 °C to give a clear syrup; which was confirmed by ESI-MS. Yield: 40 mg, 37 μmol, 66.6%.

***m/z* (ES$^-$)** 1077.2 [*M* - H]$^-$

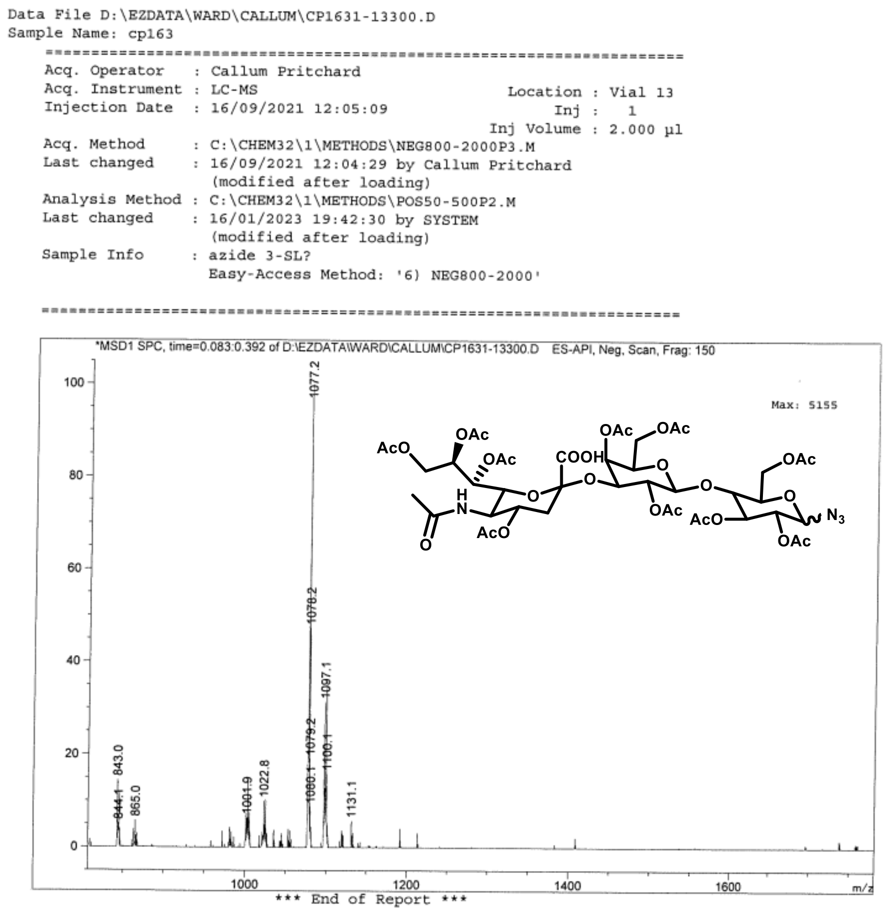

Figure S49. LR-ESI-MS spectrum of **1-azido-3'-sialyllactose (Ac)** (**55**)

Esterification

To a flame-dried 2-neck RBF under N_2 was added oven-dried 3'-sialyllactose (Ac) (350 mg, 0.32 mmol, 1 eq.) in dry MeOH (15 mL) and dry toluene (15 mL). The clear solution was then cooled to 0 °C. (Trimethylsilyl)diazomethane (1.8 to 2.4 M solution in hexanes) was added until the yellow colour change persisted and warmed to RT[10,12]. The mixture was stirred for 2 h, cooled back to 0 °C and the excess diazomethane quenched with glacial acetic acid until discolouration. The solvents were then evaporated and subsequently co-evaporated with toluene (50 mL) to afford a white foam; which was confirmed by ESI-MS. Yield: 343 mg, 0.31 mmol, 96.6%.

m/z **(ES$^+$)** 1132.2 $[M + Na]^+$

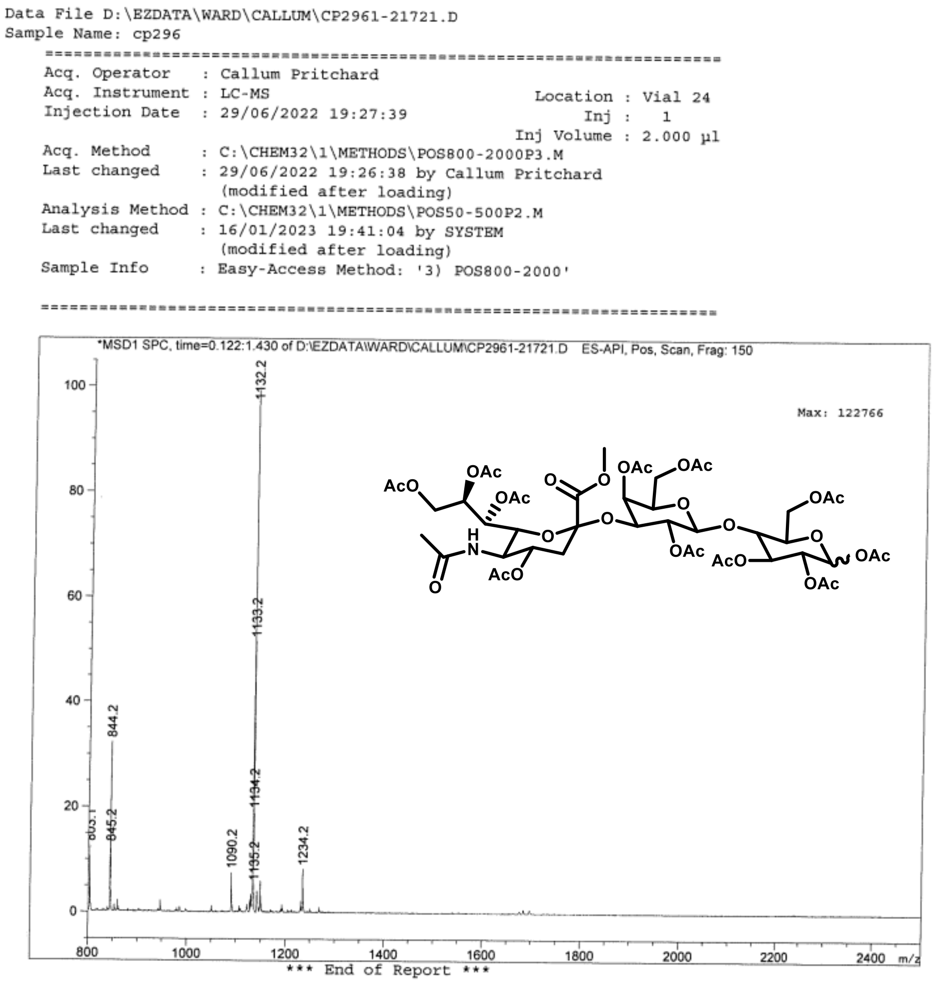

Figure S50. LR-ESI-MS spectrum of **3'-sialyllactose (Ac/Me*)** (**57**)

where **Me is used to denote the methyl ester form of the carboxylic acid group.*

**3'- and 6'-sialyllactose (Ac/Me) were then brominated and converted to their azido derivatives via the above methods.*

```
Data File D:\EZDATA\WARD\CALLUM\CP2991-21989.D
Sample Name: cp299
=================================================================
   Acq. Operator   : Callum Pritchard
   Acq. Instrument : LC-MS                      Location : Vial 14
   Injection Date  : 07/07/2022 13:14:55             Inj :   1
                                              Inj Volume : 2.000 µl
   Acq. Method     : C:\CHEM32\1\METHODS\POS800-2000P3.M
   Last changed    : 07/07/2022 13:14:10 by Callum Pritchard
                     (modified after loading)
   Analysis Method : C:\CHEM32\1\METHODS\POS50-500P2.M
   Last changed    : 16/01/2023 19:41:52 by SYSTEM
                     (modified after loading)
   Sample Info     : Easy-Access Method: '3) POS800-2000'

=================================================================
```

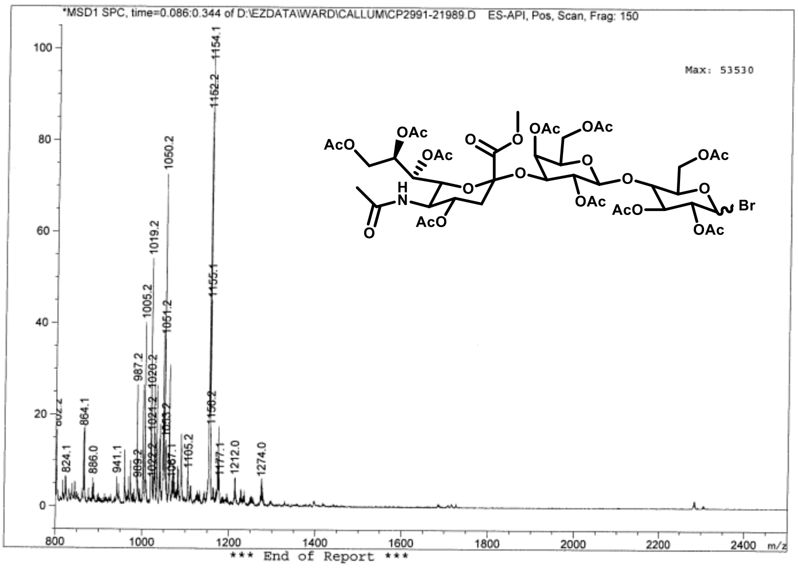


```
*** End of Report ***
```

***m/z* (ES$^+$)** 1152.2 and 1154.1 [M + Na]$^+$

Figure S51. LR-ESI-MS spectrum of **1-bromo-3'-sialyllactose (Ac/Me)** (**59**)

```
Data File D:\EZDATA\WARD\CALLUM\CP3061-22412.D
Sample Name: cp306
   ==================================================================
   Acq. Operator    : Callum Pritchard
   Acq. Instrument  : LC-MS                        Location : Vial 11
   Injection Date   : 20/07/2022 15:18:09               Inj :   1
                                                 Inj Volume : 2.000 µl
   Acq. Method      : C:\CHEM32\1\METHODS\POS800-2000P3.M
   Last changed     : 20/07/2022 15:17:25 by Callum Pritchard
                      (modified after loading)
   Analysis Method  : C:\CHEM32\1\METHODS\POS50-500P2.M
   Last changed     : 16/01/2023 19:42:02 by SYSTEM
                      (modified after loading)
   Sample Info      : azide 3sl me
                      Easy-Access Method: '3) POS800-2000'

   ==================================================================
```

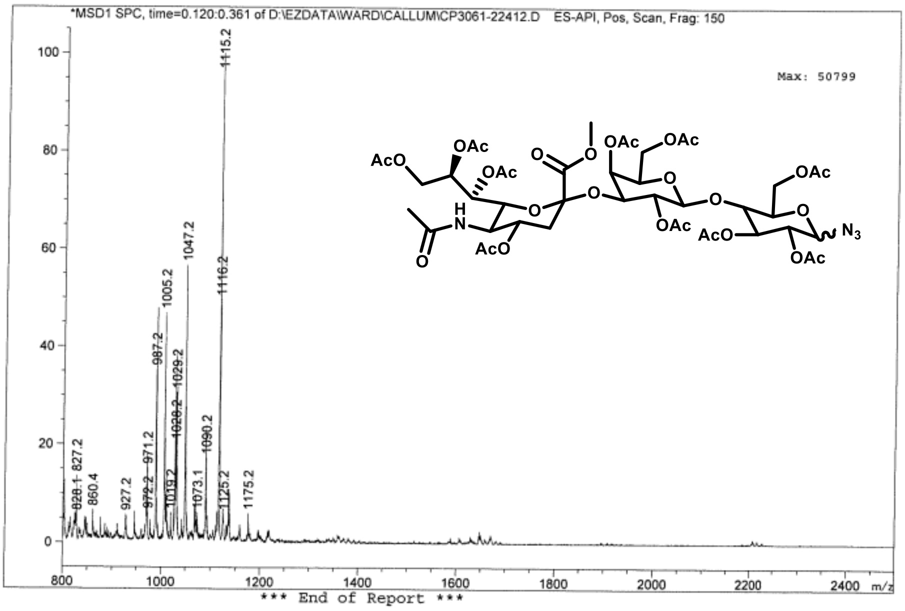

*** End of Report ***

m/z **(ES^+)** 1115.2 [*M* + Na]$^+$

Figure S52. LR-ESI-MS spectrum of **1-azido-3'-sialyllactose (Ac/Me) (61**)

Selected IR spectra of 3’-sialyllactose compounds and subsequent cubic cage ligands

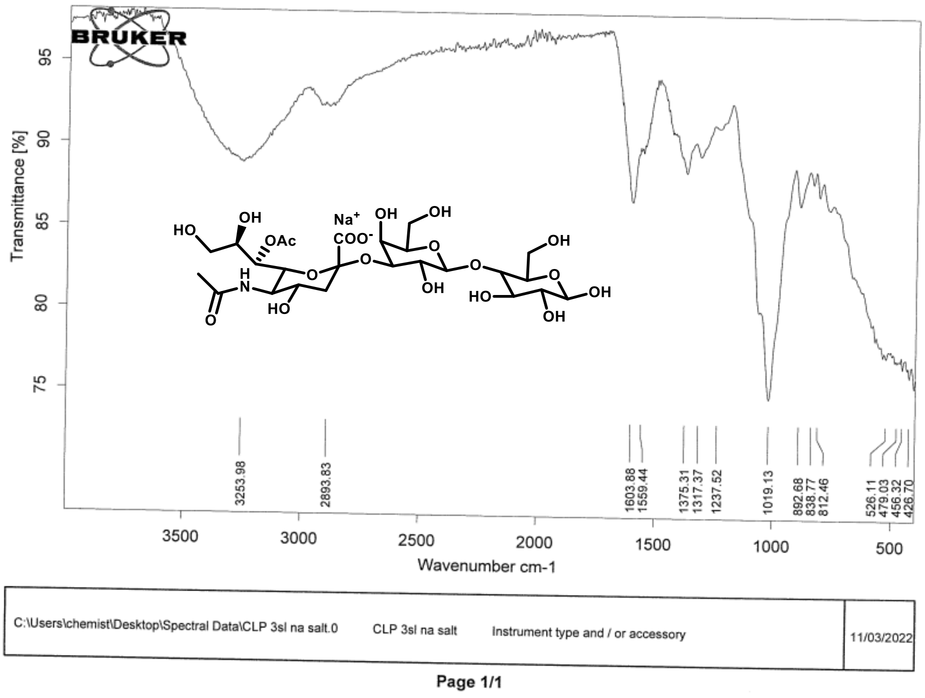

Figure S53. FTIR spectrum of **3’-sialyllactose sodium salt** (**24**)

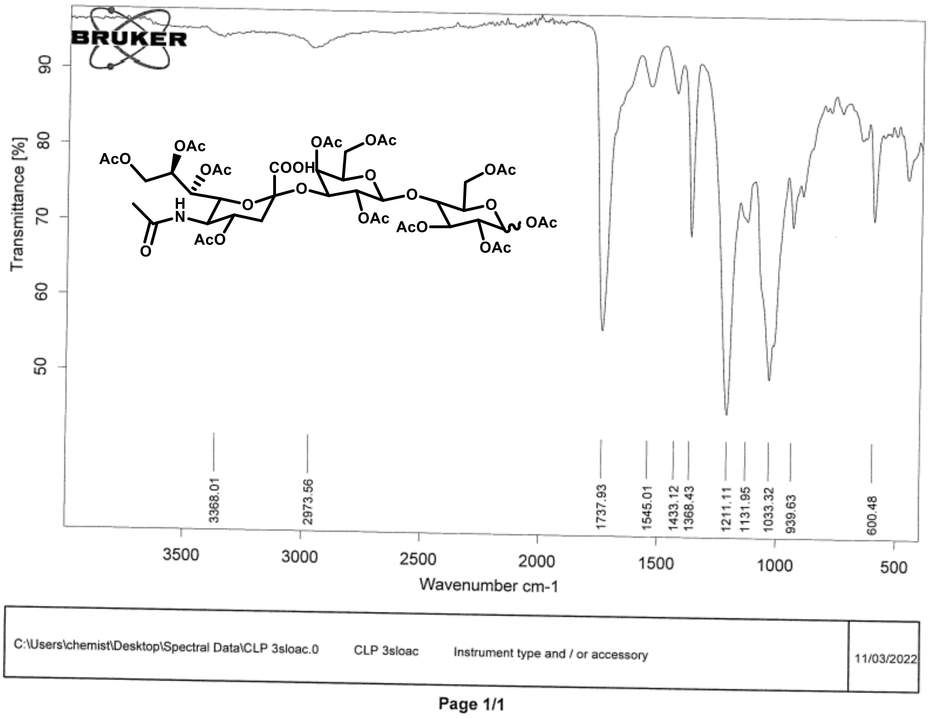

Figure S54. FTIR spectrum of **3’-sialyllactose(Ac)**

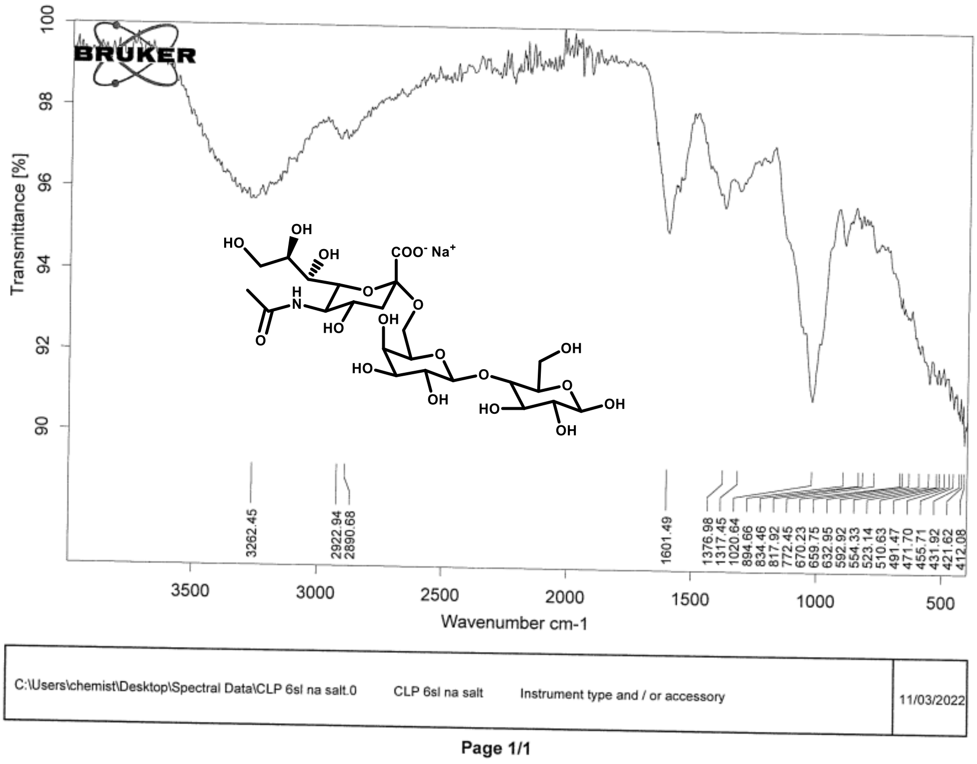

Figure S55. FTIR spectrum of **6'-sialyllactose sodium salt** (**25**)

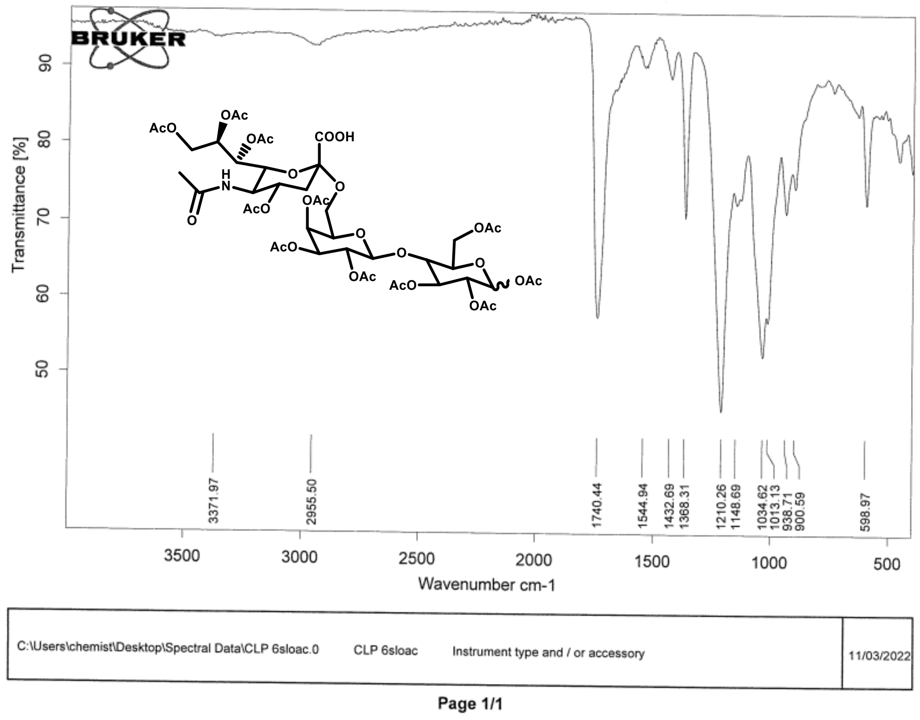

Figure S56. FTIR spectrum of **6'-sialyllactose(Ac)**

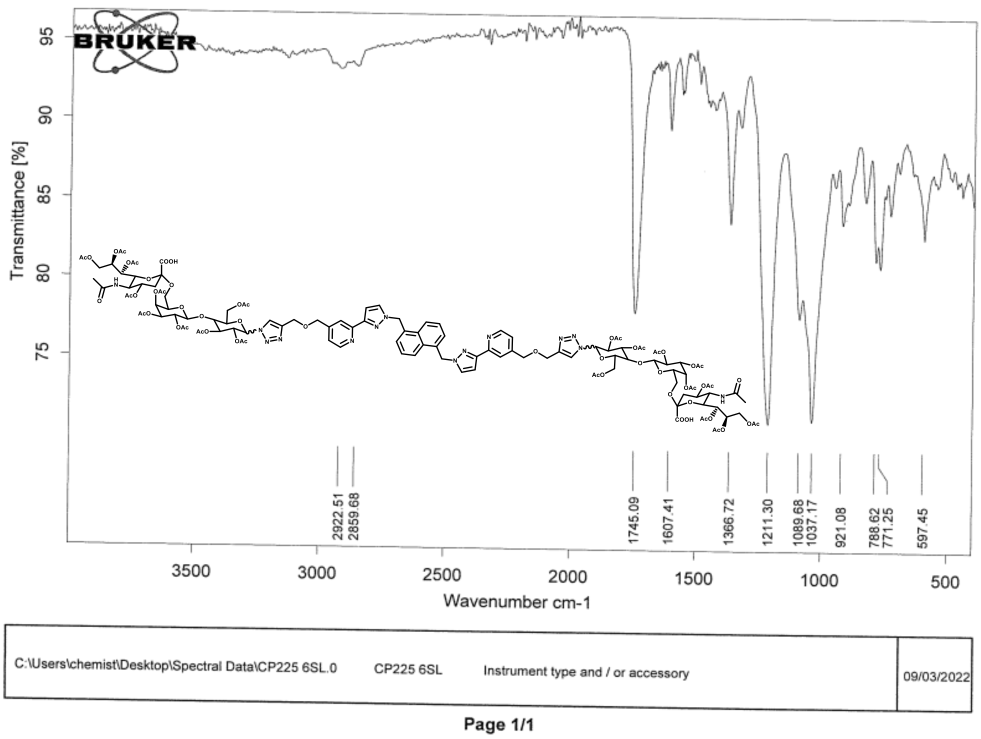

Figure S57. FTIR spectrum of $\mathbf{L^{15\text{-}6SL\text{-}Ac}}$

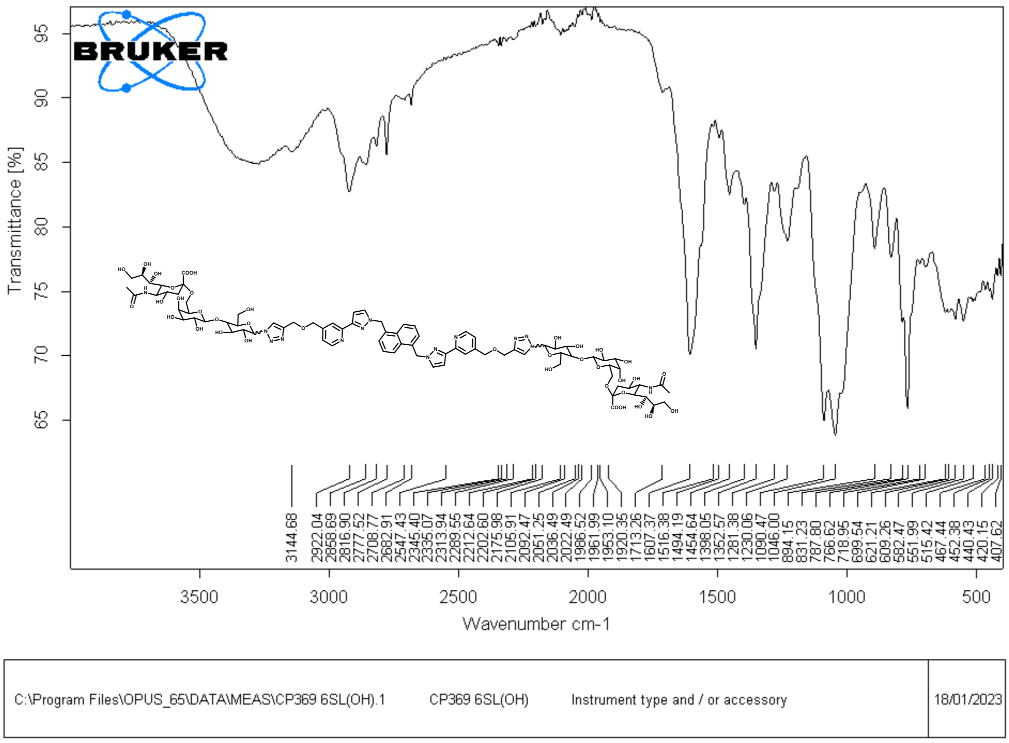

Figure S58. FTIR spectrum of $\mathbf{L^{15\text{-}6SL}}$

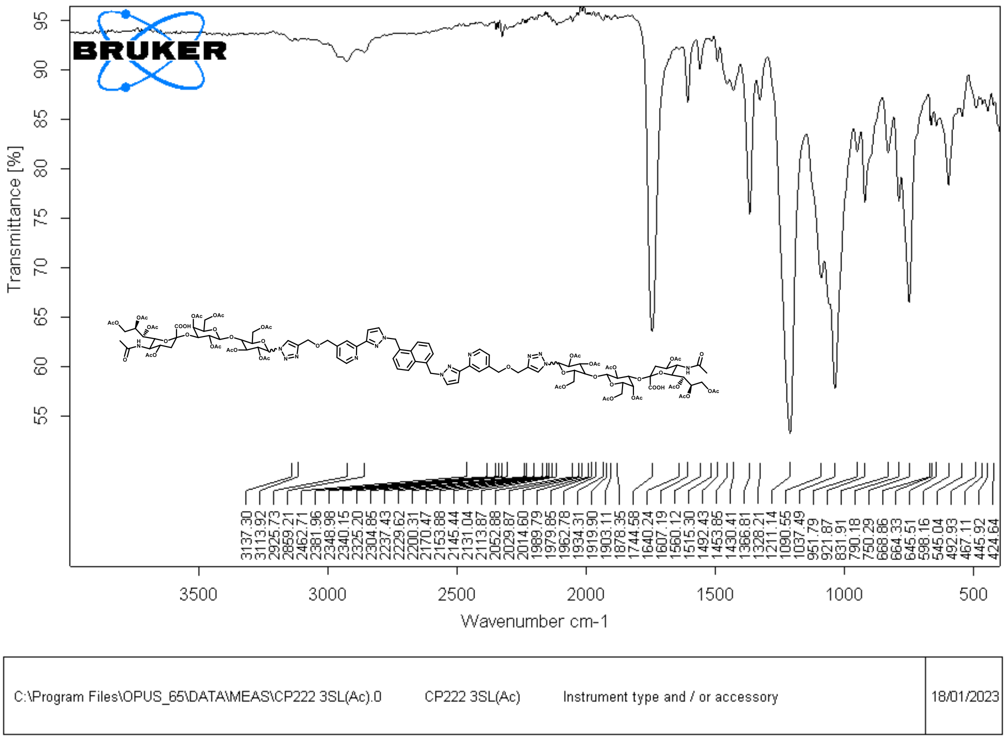

Figure S59. FTIR spectrum of $\mathbf{L^{15\text{-}3SL\text{-}Ac}}$

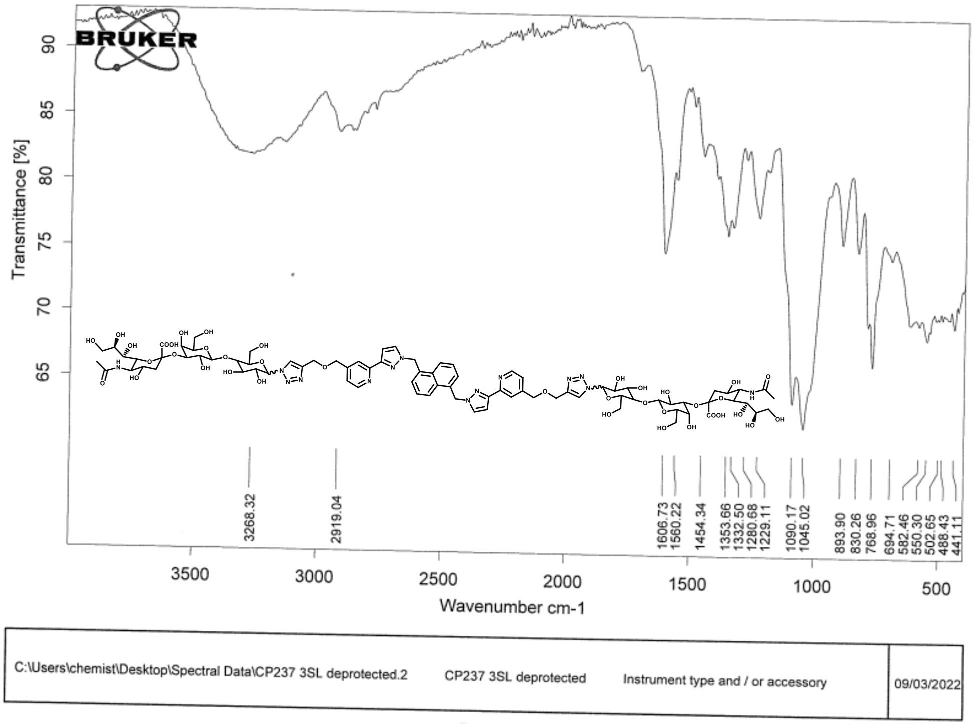

Figure S60. FTIR spectrum of $\mathbf{L^{15\text{-}3SL}}$

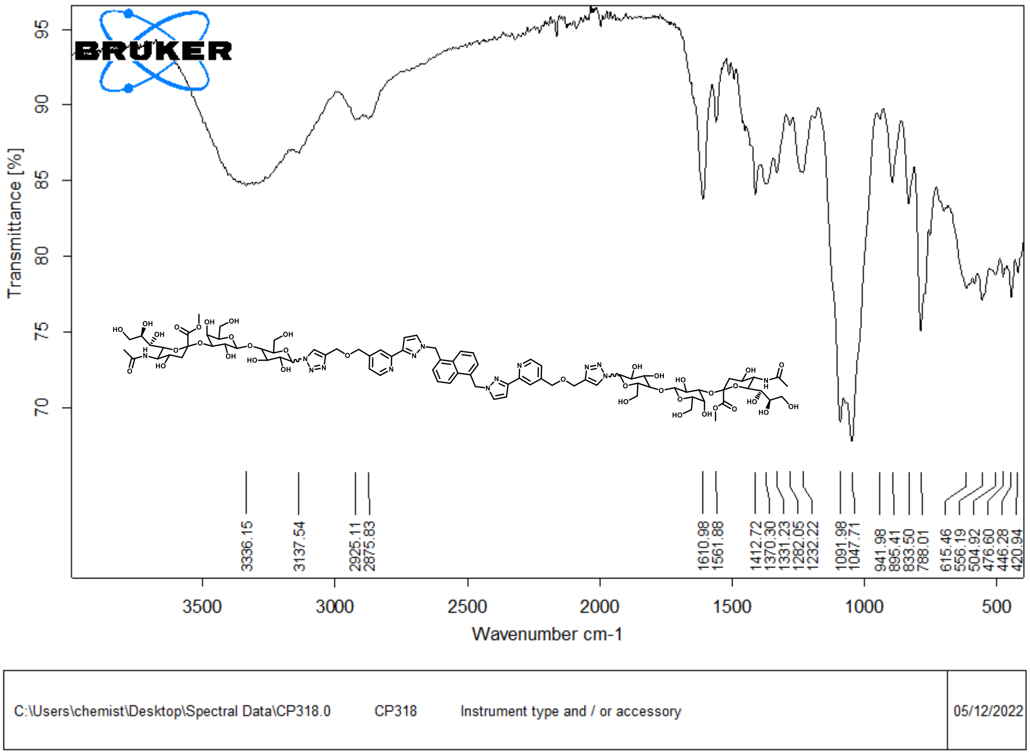

Figure S61. FTIR spectrum of **L**$^{\mathbf{15\text{-}3SL\text{-}Ac\text{-}Me}}$

Selected HRMS spectra of cubic cage ligands

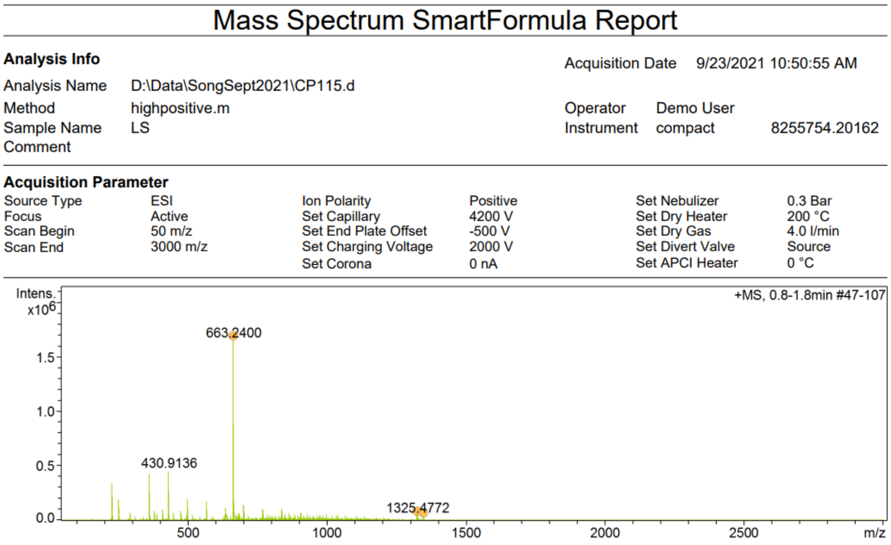

Figure S62. HR-ESI-MS spectrum of **L$^{15\text{-Glu-Ac}}$**

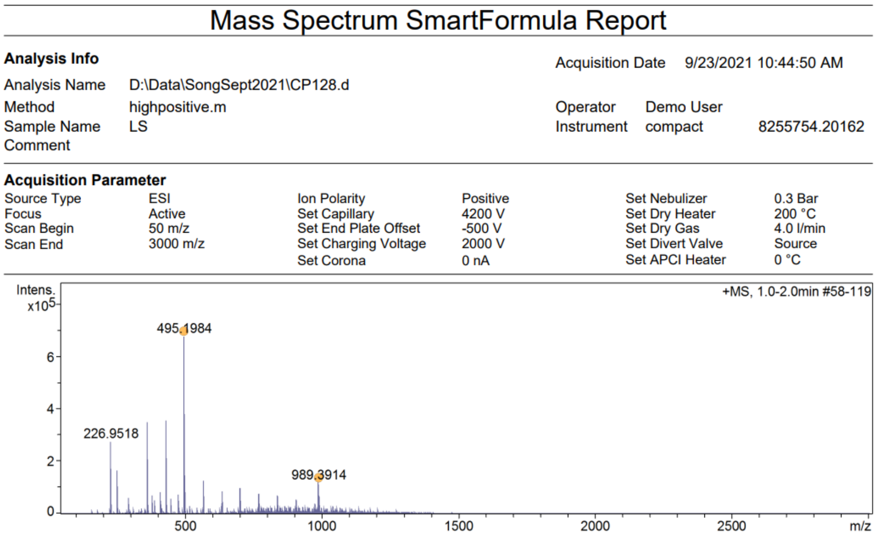

Figure S63. HR-ESI-MS spectrum of **L$^{15\text{-Glu}}$**

Mass Spectrum SmartFormula Report

Analysis Info

Analysis Name	D:\Data\SongSept2021\CP139.d	Acquisition Date	9/23/2021 11:20:05 AM
Method	highpositive.m	Operator	Demo User
Sample Name	LS	Instrument	compact 8255754.20162
Comment			

Acquisition Parameter

Source Type	ESI	Ion Polarity	Positive	Set Nebulizer	0.3 Bar
Focus	Active	Set Capillary	4200 V	Set Dry Heater	200 °C
Scan Begin	50 m/z	Set End Plate Offset	-500 V	Set Dry Gas	4.0 l/min
Scan End	3000 m/z	Set Charging Voltage	2000 V	Set Divert Valve	Source
		Set Corona	0 nA	Set APCI Heater	0 °C

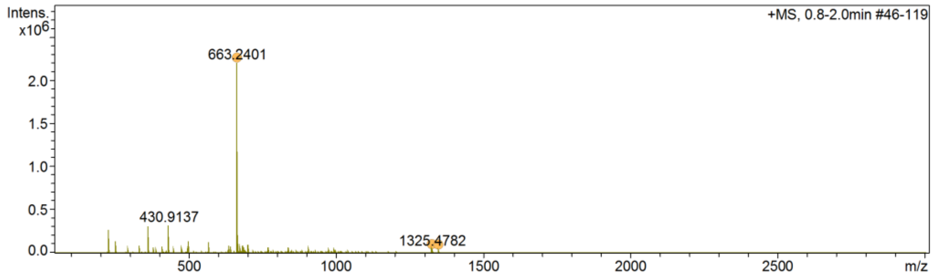

Figure S64. HR-ESI-MS spectrum of $\mathbf{L}^{\mathbf{15\text{-}Gal\text{-}Ac}}$

Mass Spectrum SmartFormula Report

Analysis Info

Analysis Name	D:\Data\SongSept2021\CP136.d	Acquisition Date	9/23/2021 11:08:52 AM
Method	highpositive.m	Operator	Demo User
Sample Name	LS	Instrument	compact 8255754.20162
Comment			

Acquisition Parameter

Source Type	ESI	Ion Polarity	Positive	Set Nebulizer	0.3 Bar
Focus	Active	Set Capillary	4200 V	Set Dry Heater	200 °C
Scan Begin	50 m/z	Set End Plate Offset	-500 V	Set Dry Gas	4.0 l/min
Scan End	3000 m/z	Set Charging Voltage	2000 V	Set Divert Valve	Source
		Set Corona	0 nA	Set APCI Heater	0 °C

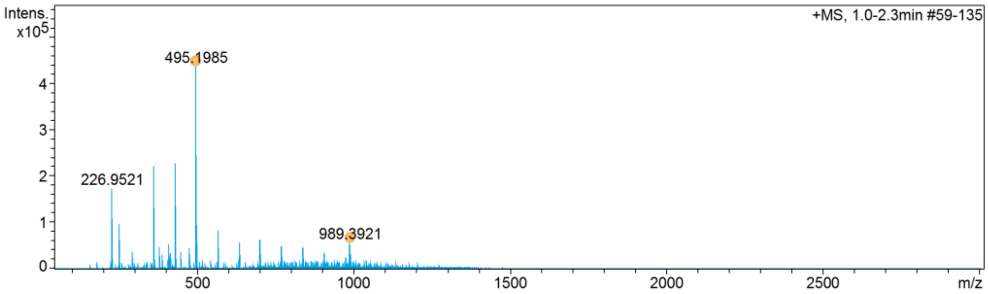

Figure S65. HR-ESI-MS spectrum of $\mathbf{L}^{\mathbf{15\text{-}Gal}}$

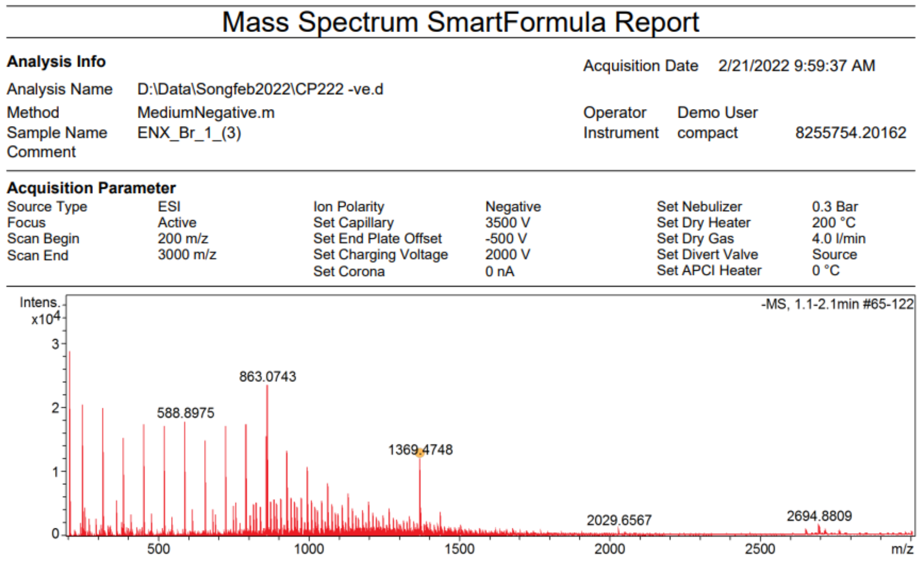

Figure S66. HR-ESI-MS (-ve) spectrum of **L**[15-3SL-Ac]

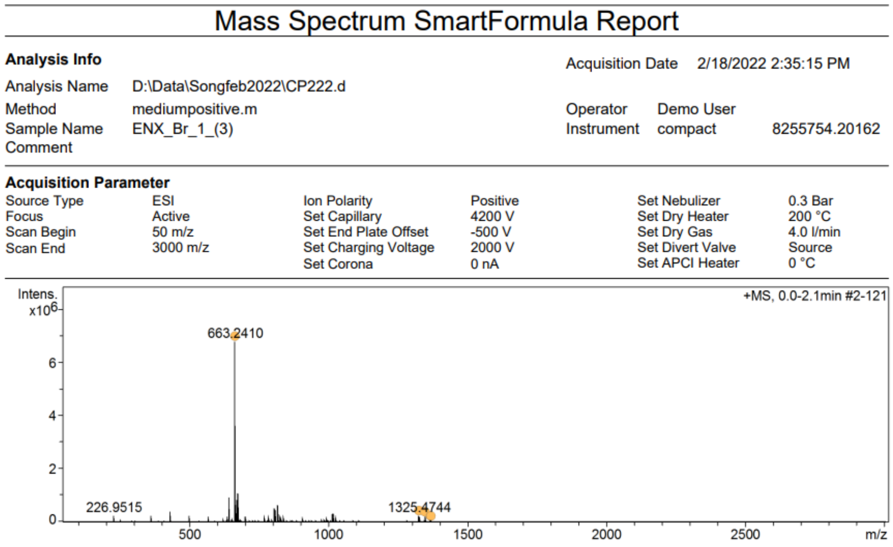

Figure S67. HR-ESI-MS (+ve) spectrum of **L**[15-3SL-Ac]

Mass Spectrum SmartFormula Report

Analysis Info

Analysis Name	D:\Data\Songfeb2022\CP225.d	Acquisition Date	2/23/2022 1:49:39 PM	
Method	mediumpositive.m	Operator	Demo User	
Sample Name		Instrument	compact	8255754.20162
Comment				

Acquisition Parameter

Source Type	ESI	Ion Polarity	Positive	Set Nebulizer	0.3 Bar
Focus	Active	Set Capillary	4200 V	Set Dry Heater	200 °C
Scan Begin	50 m/z	Set End Plate Offset	-500 V	Set Dry Gas	4.0 l/min
Scan End	3000 m/z	Set Charging Voltage	2000 V	Set Divert Valve	Source
		Set Corona	0 nA	Set APCI Heater	0 °C

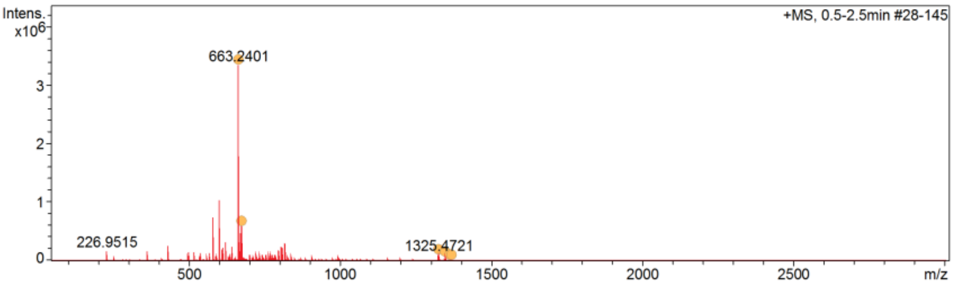

Figure S68. HR-ESI-MS (+ve) spectrum of $\mathbf{L^{15\text{-}6SL\text{-}Ac}}$

Mass Spectrum SmartFormula Report

Analysis Info

Analysis Name	D:\Data\SongNov2021\CP191.d	Acquisition Date	11/30/2021 9:38:07 AM	
Method	highPositve.m	Operator	Demo User	
Sample Name	Blank	Instrument	compact	8255754.20162
Comment				

Acquisition Parameter

Source Type	ESI	Ion Polarity	Positive	Set Nebulizer	1.2 Bar
Focus	Active	Set Capillary	4200 V	Set Dry Heater	200 °C
Scan Begin	50 m/z	Set End Plate Offset	-500 V	Set Dry Gas	8.0 l/min
Scan End	3000 m/z	Set Charging Voltage	2000 V	Set Divert Valve	Waste
		Set Corona	0 nA	Set APCI Heater	0 °C

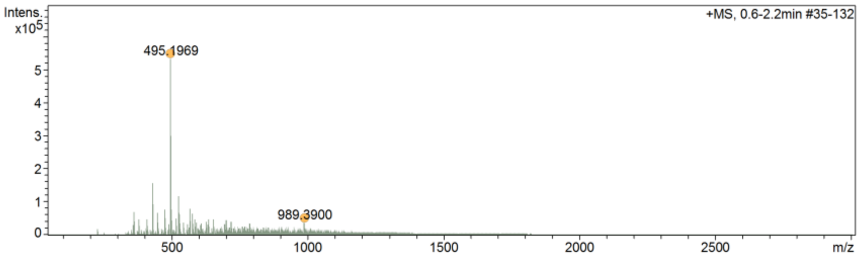

Figure S69. HR-ESI-MS (+ve) spectrum of $\mathbf{L^{15\text{-}3SL}}$

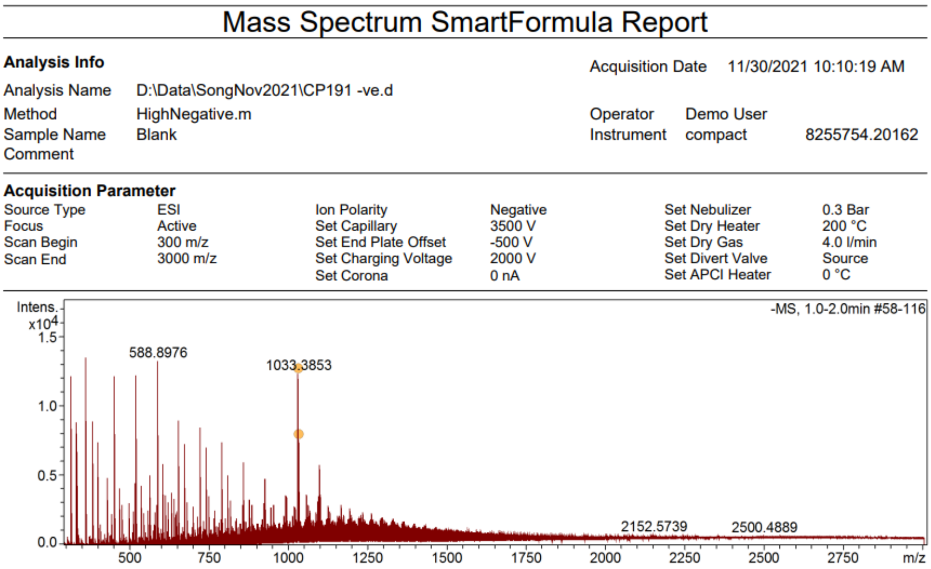

Figure S70. HR-ESI-MS (-ve) spectrum of **L$^{15\text{-}3SL}$**

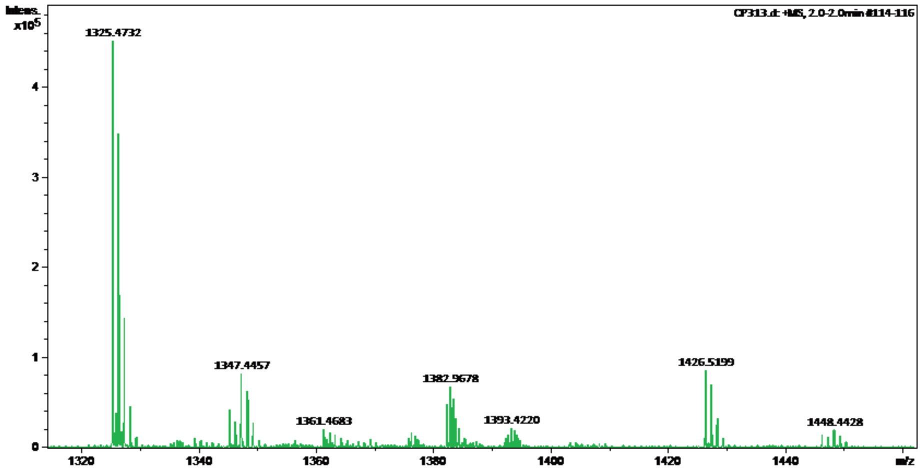

Figure S71. HR-ESI-MS (+ve) spectrum of **L$^{15\text{-}3SL\text{-}Ac\text{-}Me}$**

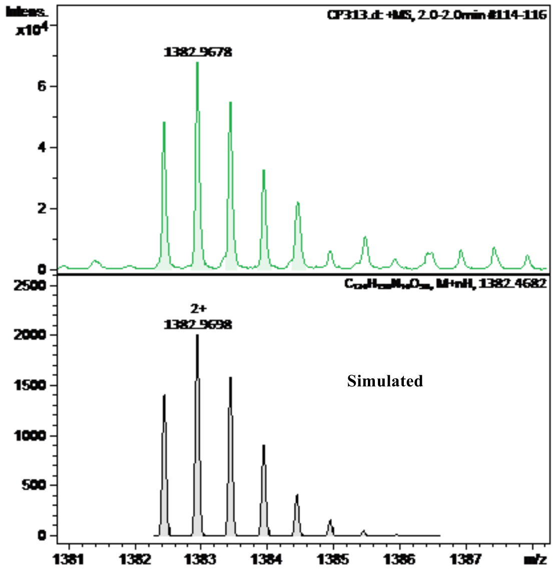

Figure S72. Zoom in of the HR-ESI-MS (+ve) spectrum of $\mathbf{L}^{\mathbf{15\text{-}3SL\text{-}Ac\text{-}Me}}$ at *m/z* 1382.9678 corresponding to $[M + 2H]^{2+}$

Selected HRMS spectra of cages

**All cage samples were analysed in H_2O or CH_3CN (for acetyl protected cages) at a concentration of 250 µM.*

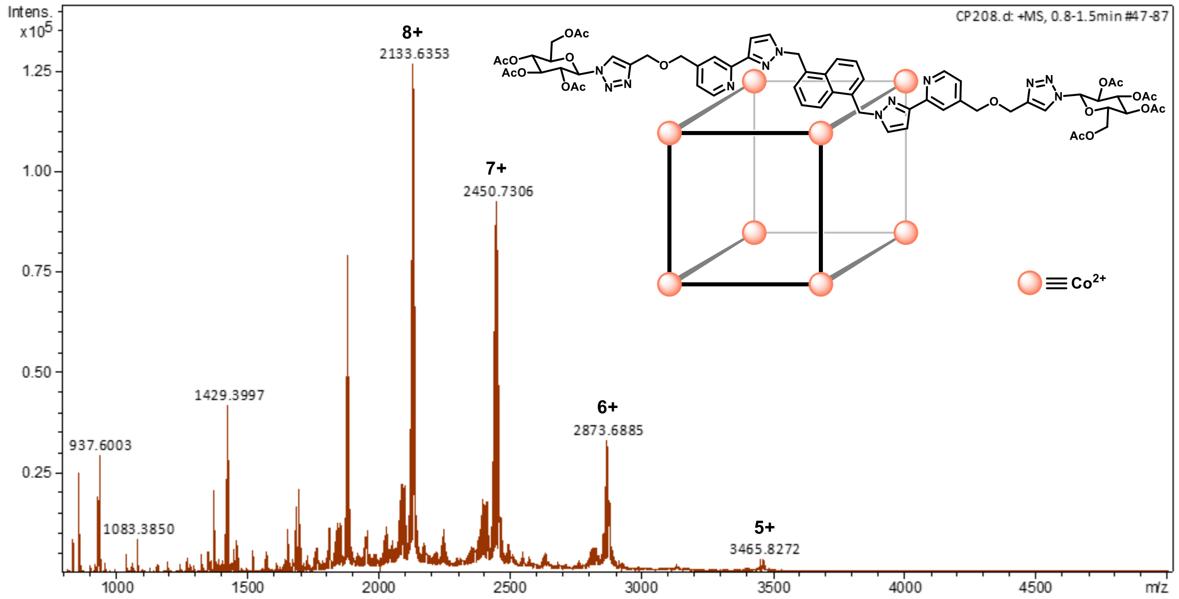

Figure S73. HR-ESI-MS of **$[Co_8(L^{15\text{-}Glu\text{-}Ac})_{12}(BF_4)_{16}]$ $[Co_8{}^{Glu\text{-}Ac}]$** in CH_3CN where n = number of BF_4 anions lost and hence the charge in the sequence $\{[Co_8(L^{15\text{-}Glu\text{-}Ac})_{12}](BF_4)_{16\text{-}n}\}^{n+}$

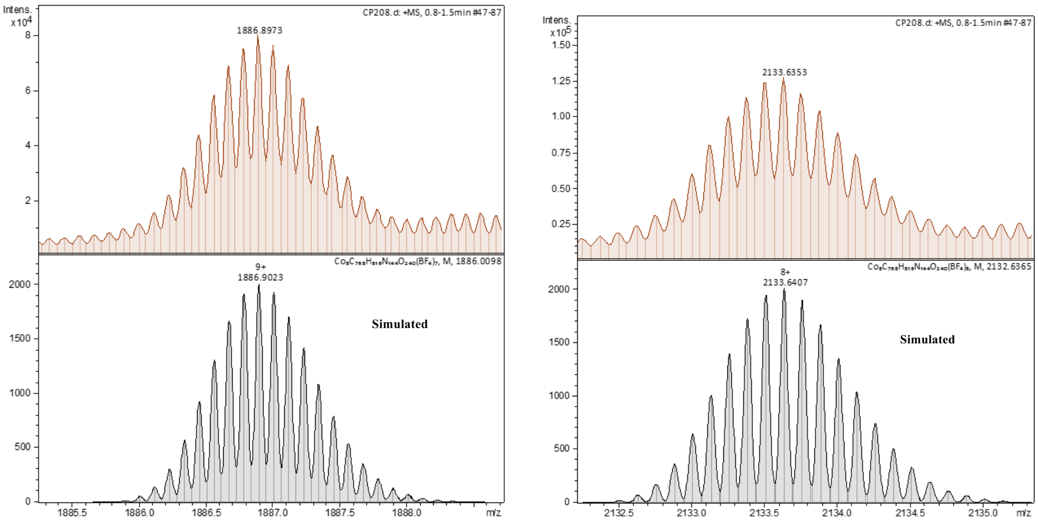

Figure S74. Selected expansions of Fig S73: **$([Co_8(L^{15\text{-}Glu\text{-}Ac})_{12}(BF_4)_7]^{9+}$** and **$([Co_8(L^{15\text{-}Glu\text{-}Ac})_{12}(BF_4)_8]^{8+}$**

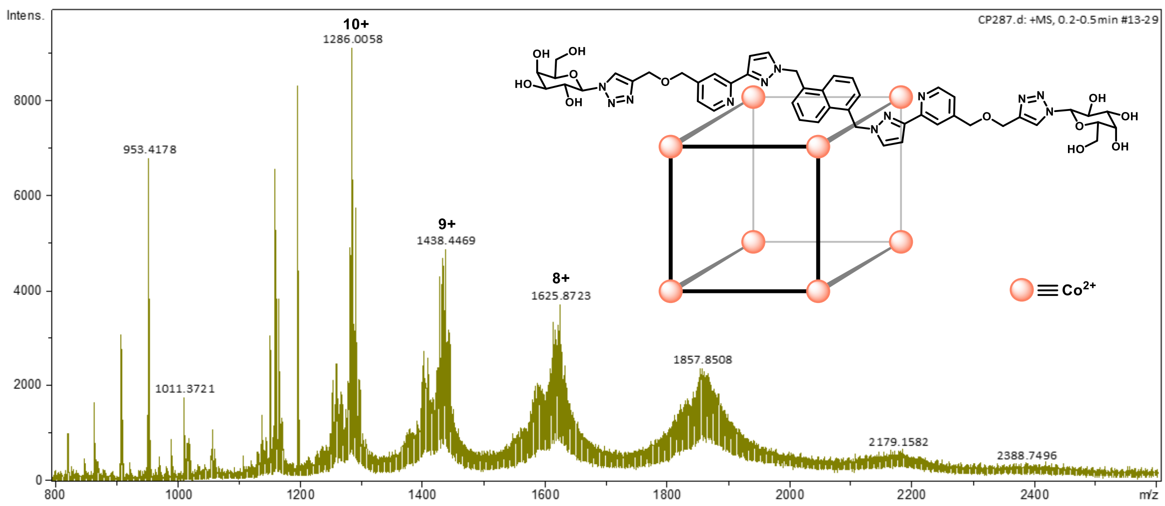

Figure S75. HR-ESI-MS of **$[Co_8(L^{15\text{-}Gal})_{12}(BF_4)_{16}]$ $[Co_8{}^{Gal}]$** in H_2O where n = number of BF_4 anions lost and hence the charge in the sequence $\{[Co_8(L^{15\text{-}Gal})_{12}](BF_4)_{16\text{-}n}\}^{n+}$

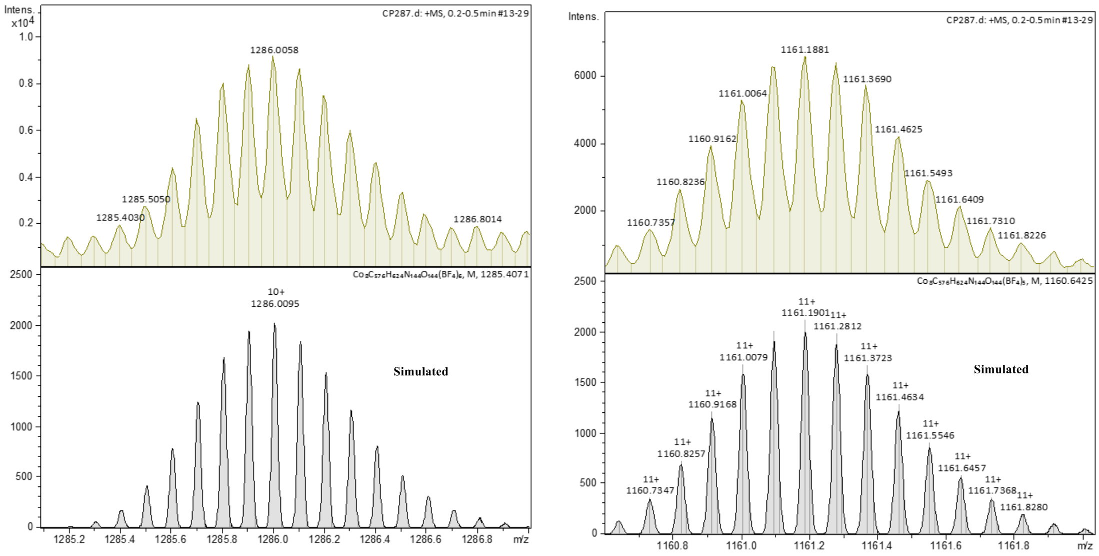

Figure S76. Selected expansions of Fig S75: **($[Co_8(L^{15\text{-}Gal})_{12}(BF_4)_6]^{10+}$** and **($[Co_8(L^{15\text{-}Gal})_{12}(BF_4)_5]^{11+}$**

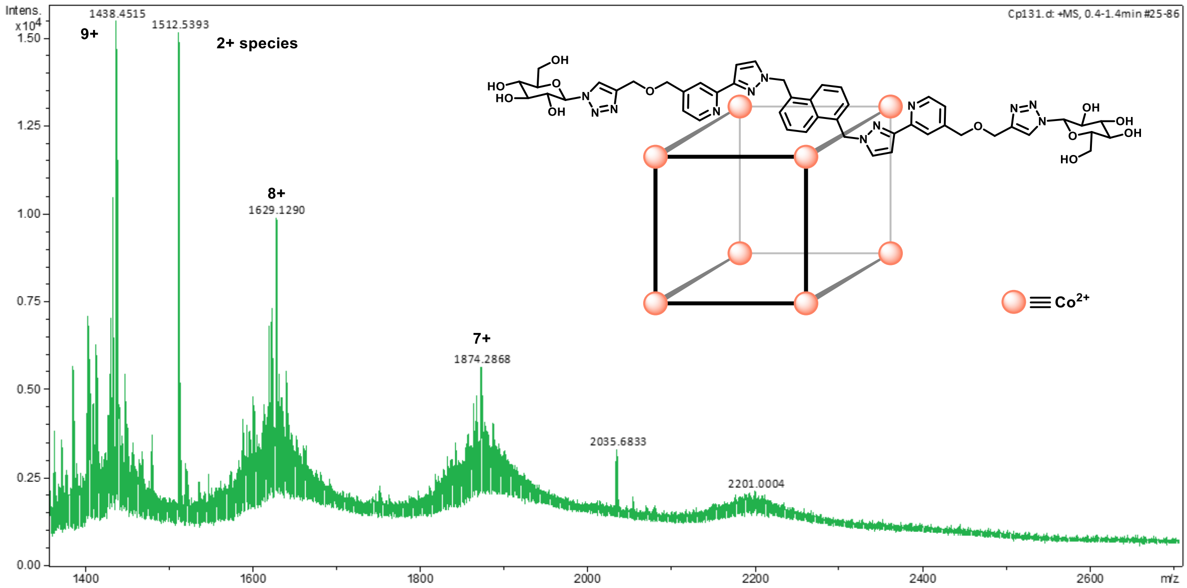

Figure S77. HR-ESI-MS of **[Co_8($L^{15\text{-}Glu}$)$_{12}$(BF_4)$_{16}$] [Co_8^{Glu}]** in H_2O where n = number of BF_4 anions lost and hence the charge in the sequence {[Co_8($L^{15\text{-}Glu}$)$_{12}$](BF_4)$_{16\text{-}n}$}$^{n+}$

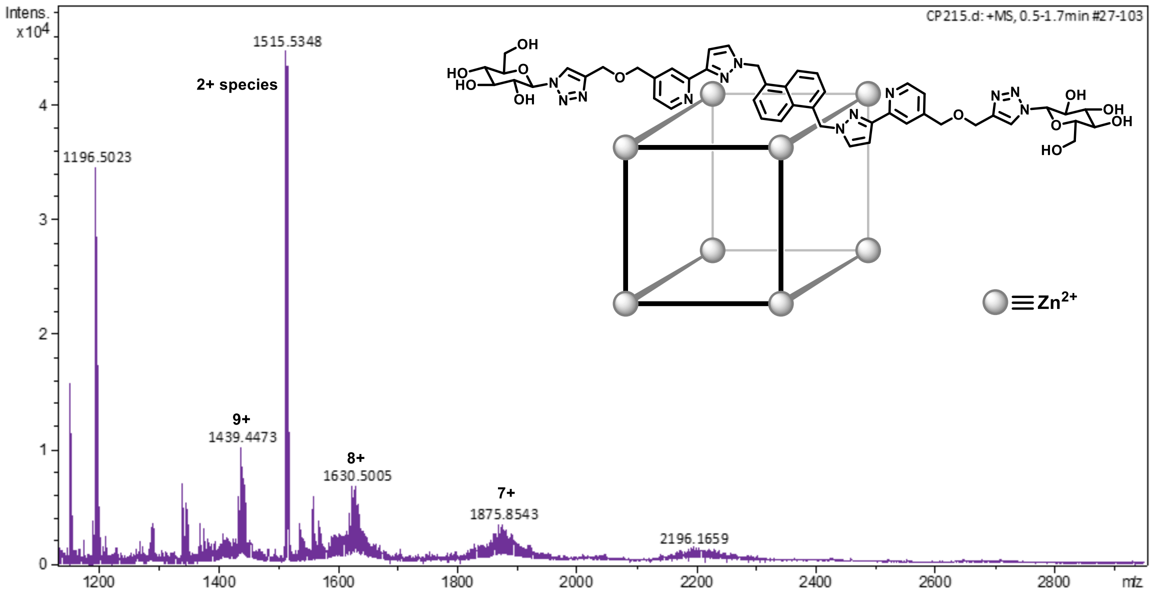

Figure S78. HR-ESI-MS of **[Zn_8($L^{15\text{-}Glu}$)$_{12}$(BF_4)$_{16}$] [Zn_8^{Glu}]** in H_2O where n = number of BF_4 anions lost and hence the charge in the sequence {[Zn_8($L^{15\text{-}Glu}$)$_{12}$](BF_4)$_{16\text{-}n}$}$^{n+}$

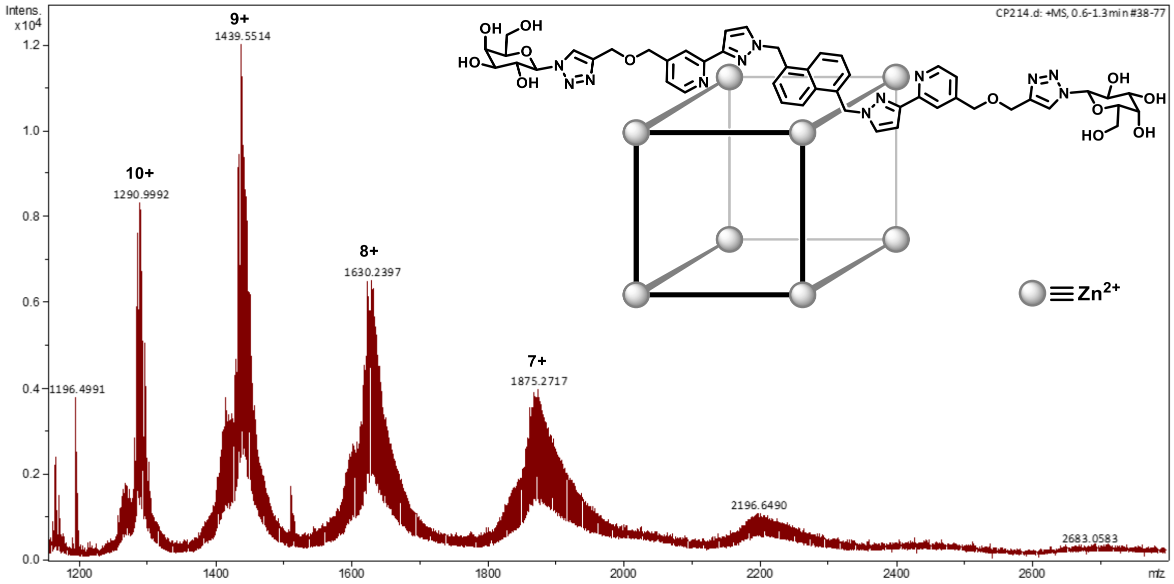

Figure S79. HR-ESI-MS of **$[Zn_8(L^{15\text{-}Gal})_{12}(BF_4)_{16}]$ $[Zn_8{}^{Gal}]$** in H_2O where n = number of BF_4 anions lost and hence the charge in the sequence $\{[Zn_8(L^{15\text{-}Gal})_{12}](BF_4)_{16\text{-}n}\}^{n+}$

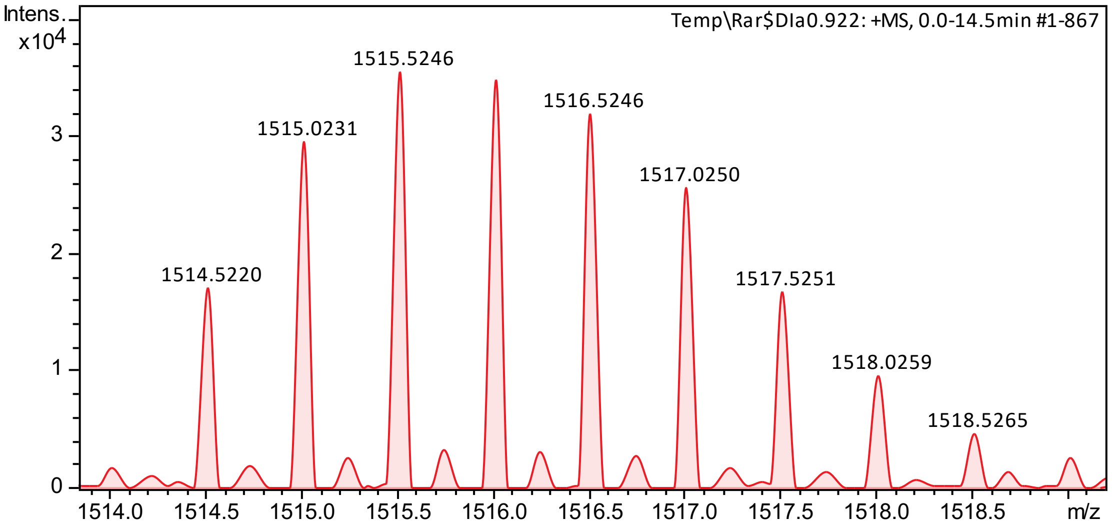

Figure S80. Selected expansion of Fig S78. The unknown species at *m/z* 1515.5246 works out to be a **$([Zn(L^{15\text{-}Glu})_3]^{2+}$** fragment. It is found in both glucose cubic cages as a major fragment and seen as a weak fragment in **$[Zn_8{}^{Gal}]$**

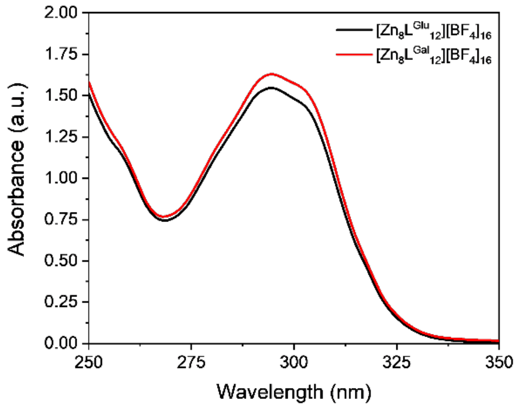

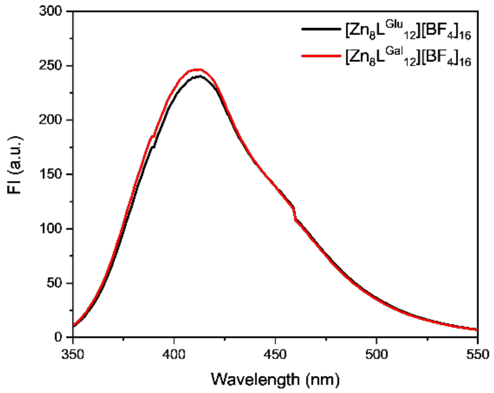

Figure S81. Absorption and emission spectra (λexc 294 nm) of **[Zn$_8$^{Gal}]** and **[Zn$_8$^{Glu}]** at 6.6 μM in H_2O

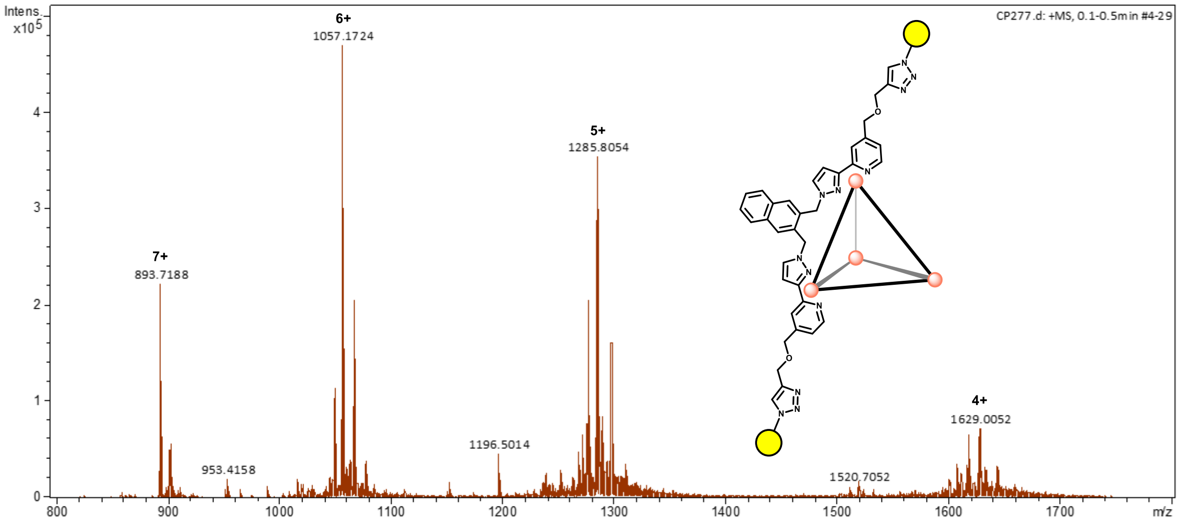

Figure S82. HR-ESI-MS of **[Co$_4$(L$^{23\text{-}Gal}$)$_6$(BF$_4$)$_8$]** in H_2O where n = number of BF_4 anions lost and hence the charge in the sequence $\{[Co_4(L^{23\text{-}Gal})_6](BF_4)_{8-n}\}^{n+}$

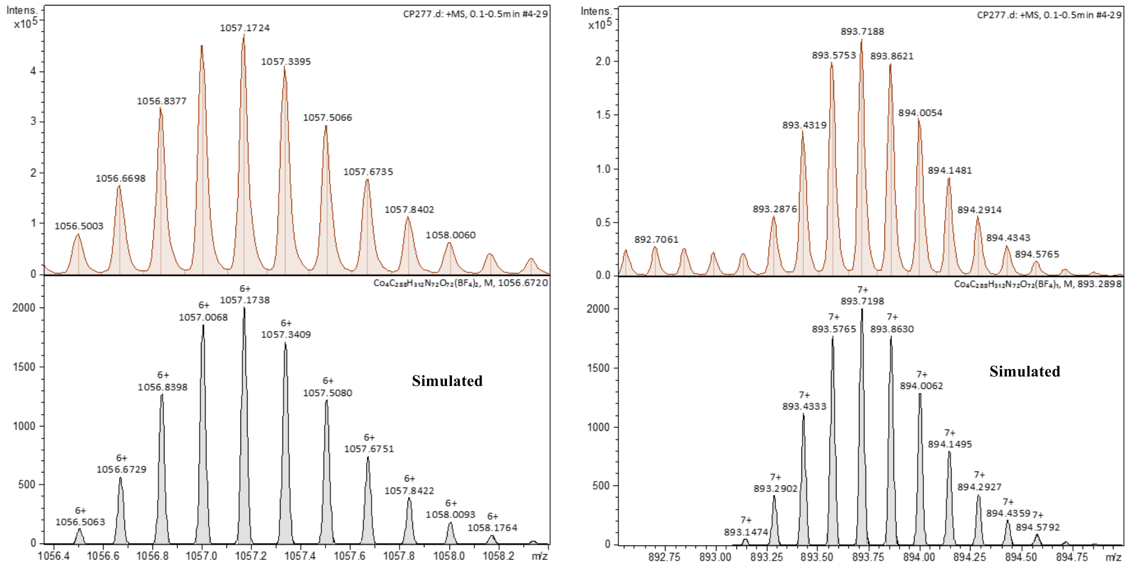

Figure S83. Selected expansions of Fig S82: **([$Co_4(L^{23\text{-}Gal})_6(BF_4)_2]^{6+}$** and **([$Co_4(L^{23\text{-}Gal})_6(BF_4)]^{7+}$**

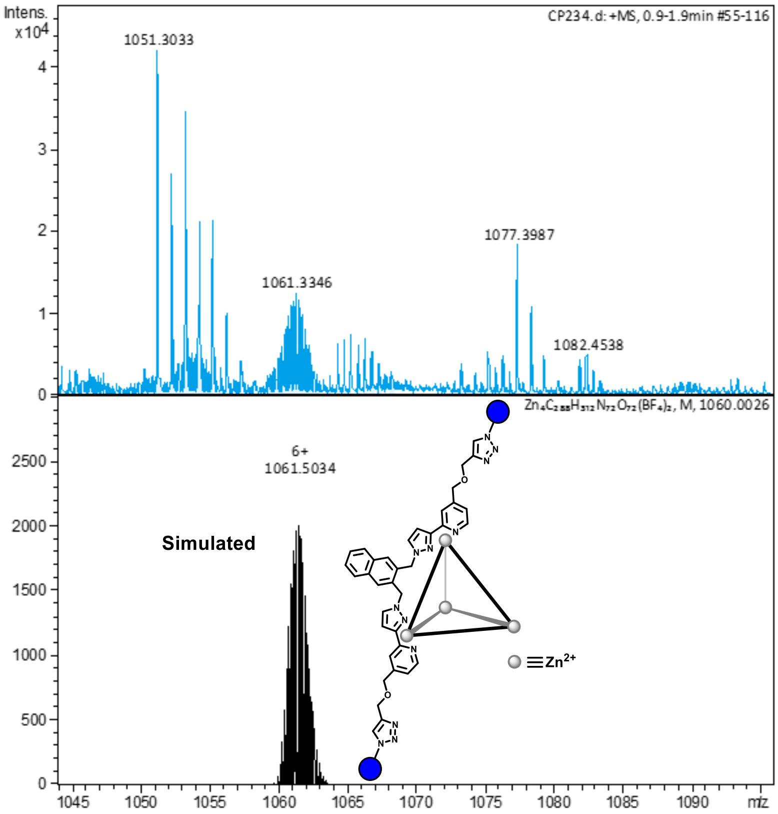

Figure S84. Selected expansion of HR-ESI-MS for **[$Zn_4(L^{23\text{-}Glu})_6(BF_4)_8$]**. The full spectra shows a sequence of varying charge states that correspond to {[$Zn_4(L^{23\text{-}Glu})_6$](Anion(s))$_{8\text{-}n}$}$^{n+}$ where n = number of anions lost.

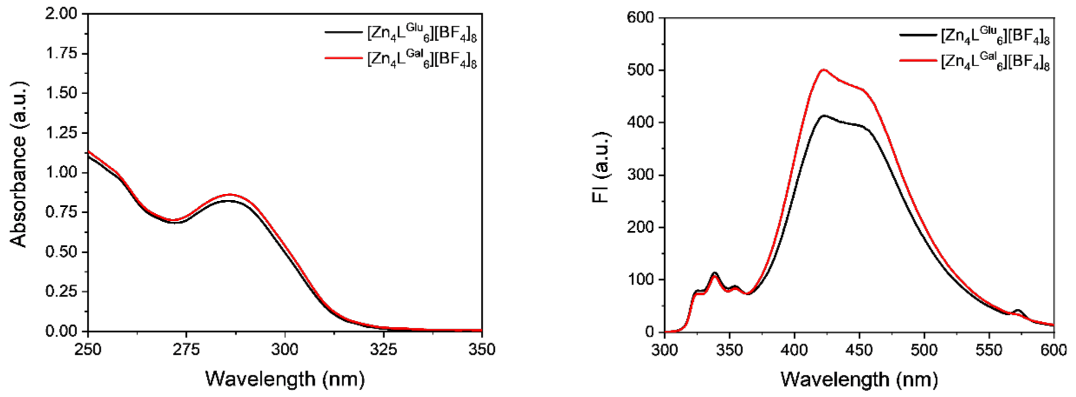

Figure S85. Absorption and emission spectra (λexc 286nm) of **[Zn$_4$^{Glu}]** and **[Zn$_4$^{Gal}]** at 6.6 µM in H_2O

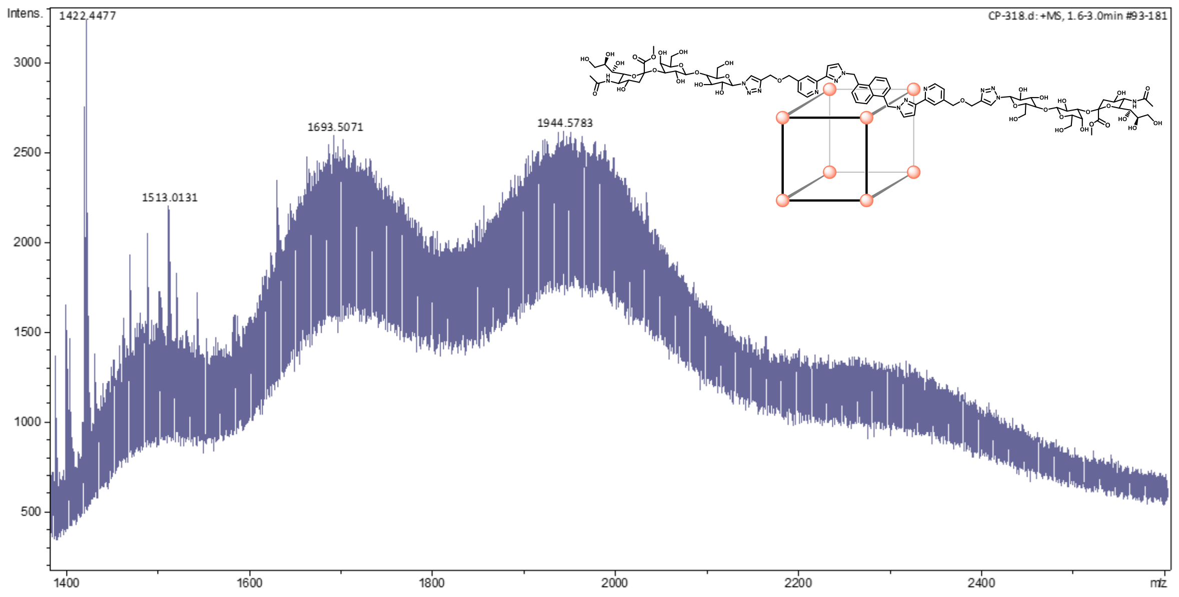

This appears to be something similar to what is expected, but signal is very weak even after optimisation of instrument parameters

Figure S86. Zoom in the region *m/z* 1400-2600 of the HR-ESI-MS of **[Co$_8$(L$^{15\text{-}3SL\text{-}Me}$)$_{12}$(BF$_4$)$_{16}$]** in H_2O; with a comment from the in-house MS professional. Out of the many samples run with the deprotected 3'-sialyllactose cage family, this was the only sample that showed any trace sign of the signals expected for the cubic cage: signals for the 3-SL pendant cages are very weak even at the same concentration as used for the monosaccharide cages.

Expected signals within this region: 1576.3060 ([Co$_8$(L$^{15\text{-}3SL\text{-}Me}$)$_{12}$(BF$_4$)$_1$]$^{15+}$), 1695.0996 ([Co$_8$(L$^{15\text{-}3SL\text{-}Me}$)$_{12}$(BF$_4$)$_2$]$^{14+}$), 1832.1692 ([Co$_8$(L$^{15\text{-}3SL\text{-}Me}$)$_{12}$(BF$_4$)$_3$]$^{13+}$), 1992.0837 ([Co$_8$(L$^{15\text{-}3SL\text{-}Me}$)$_{12}$(BF$_4$)$_4$]$^{12+}$). **Mw**: 24946.66 g/mol

Synthesis of cubic cage ligands (3'- and 6'-Sialyllactose pendants)

For all these ligands (apart from the **Me esters), no useful information was obtained from ESI-MS and MALDI-TOF-MS*

L$^{15\text{-}3SL\text{-}Ac}$ (63)

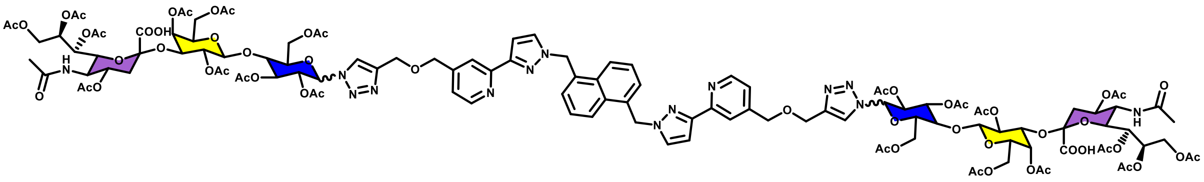

This was prepared via the same CuAAC procedure as used for the monosaccharide-pendant ligands: 134 mg of **L^{15CC}** used. Yield: 238 mg, 87 μmol, 38%.

^{1}H-NMR (400 MHz, **CDCl$_3$**, 298 K) *δ*ppm: 8.62 (2H, d, J = 5.0 Hz, pyridyl H^6); 8.03 (2H, d, 8.6 Hz, naphthyl H$^{4/8}$); 7.98 (2H, s, pyridyl H^3); 7.88 (1H, d, J = 5.7 Hz, triazole H^5), 7.85 (1H, s, triazole H^5); 7.48 (2H, t, J = 7.7 Hz, naphthyl H$^{3/7}$); 7.33 (2H, d, J = 6.7 Hz, naphthyl H$^{2/6}$); 7.28-7.24 (4H, pyrazole H^5 + pyridyl H^5); 6.97 (2H, s, pyrazole H^4); 5.89-5.83 (4H, m, glucose H^1 and galactose H^1); 5.86 (4H, s, pyridyl-C**H$_2$**N); 5.54 (4H, t, J = 9.1 Hz, galactose H^{2+4}); 5.45-5.36 (4H, m, glucose H^{2+3}); 5.29-5.21 (4H, m, glucose H^4 and galactose H^3); 4.76 (2H, s, pyridyl-C**H$_2$**O); 4.75 (2H, s, pyridyl-C**H$_2$**O); 4.66 (4H, s, O-C**H$_2$**-CNR); 4.32-4.09 (10H, m, glucose H^{6a+6b} and galactose H$^{6a+6b+5}$); 4.02-3.98 (2H, m, glucose H^5); 2.21 (6H, s, methyl C**H$_3$**); 2.20; 2.07; 2.06 (12H, s, methyl C**H$_3$**); 2.02 (6H, s, methyl C**H$_3$**); 2.01 (6H, s, methyl C**H$_3$**); 1.88 (6H, s, methyl C**H$_3$**); 1.87 (6H, s, methyl C**H$_3$**).

^{13}C-NMR (100 MHz, **CDCl$_3$**, 298 K) *δ*ppm: 170.5, 170.3, 170.0, 169.9, 169.8, 169.4, 169.1, 168.9 (**C**OCH$_3$); 152.3 (pyridyl C^2); 151.5 (pyrazole C^3); 149.6 (pyridyl C^6); 147.6 (pyridyl C^4); 145.4, 145.4 (triazole C^4); 132.3 (naphthyl C$^{1/5}$ or C$^{9/10}$); 131.7 (naphthyl C$^{9/10}$ or C$^{1/5}$); 130.9 (pyrazole C^5); 127.3 (naphthyl C$^{2/6}$); 126.5 (naphthyl C$^{3/7}$); 124.3 (naphthyl C$^{4/8}$); 121.3, 121.2 (triazole C^5); 120.7 (pyridyl C^5); 118.3 (pyridyl C^3); 105.0 (pyrazole C^4); 99.5; 86.3; 86.1 (galactose C^1); 85.7 (glucose C^1); 76.2; 75.9; 75.1 (glucose C^5); 74.0 (galactose C^5); 72.6 (glucose C^3); 70.8, 70.7 (O-**C**H$_2$-CNR + galactose C^3); 70.6; 70.4 (glucose C^2); 69.5; 68.3; 68.0 (galactose C^2); 67.8, 67.7 (glucose C^4); 67.3; 66.9 (galactose C^4); 63.9, 63.9 (pyridyl-**C**H$_2$O); 61.5 (glucose C^6); 61.2 (galactose C^6); 54.7 (pyridyl-**C**H$_2$N); 29.7; 26.9; 20.9; 20.7; 20.6; 20.5; 20.5; 20.3; 20.2; 20.2 (**C**H$_3$'s)

ν_{max}/cm^{-1}: 2927w, 2860w, 1743s, 1607m, 1367m, 1211s, 1090m, 1037s.

$L^{15\text{-}6SL\text{-}Ac}$ (64)

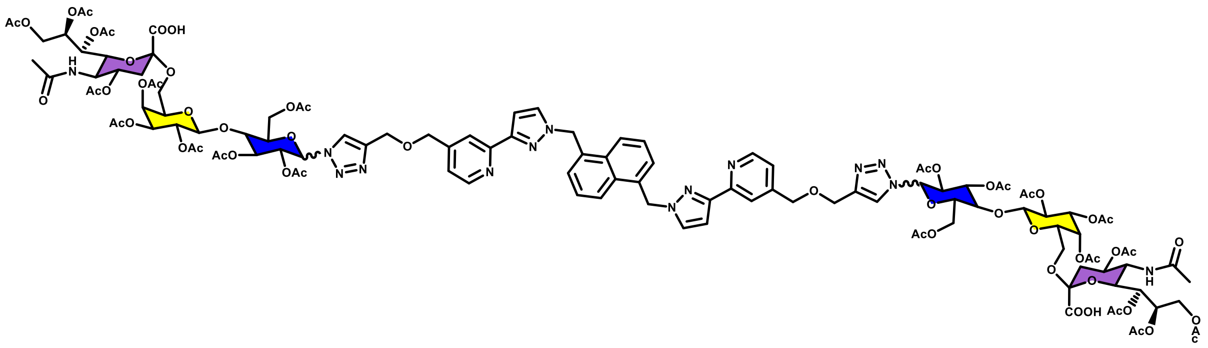

This was prepared via the same CuAAC procedure as used for the monosaccharide-pendant ligands: 120 mg of **L^{15CC}** used. Yield: 177 mg, 65 μmol, 31%.

^{1}H-NMR (400 MHz, **$CDCl_3$**, 298 K) *δ*ppm: 8.61 (2H, d, J = 5.0 Hz, pyridyl H^6); 8.03 (2H, d, 8.6 Hz, naphthyl $H^{4/8}$); 7.94 (2H, s, pyridyl H^3); 7.89 (2H, s, triazole H^5); 7.47 (2H, t, J = 8.0 Hz, naphthyl $H^{3/7}$); 7.32 (2H, d, J = 6.8 Hz, naphthyl $H^{2/6}$); 7.26 (2H, d, J = 1.8 Hz, pyrazole H^5); 7.23 (2H, d, J = 4.7 Hz, pyridyl H^5); 6.88 (2H, d, J = 1.5 Hz, pyrazole H^4); 5.91 (4H, t, J = 9.1 Hz, glucose H^1 and galactose H^1); 5.86 (4H, s, pyridyl-C**H_2**N); 5.56 (4H, dd, J = 10.0, 9.6 Hz, galactose H^{2+4}); 5.49-5.42 (4H, m, glucose H^{2+3}); 5.29-5.23 (4H, m, glucose H^4 and galactose H^3); 4.75 (2H, s, pyridyl-C**H_2**O); 4.74 (2H, s, pyridyl-C**H_2**O); 4.64 (4H, s, O-C**H_2**-CNR); 4.32-4.12 (10H, m, glucose H^{6a+6b} and galactose $H^{6a+6b+5}$); 4.05-4.00 (2H, m, glucose H^5); 2.21 (6H, s, methyl C**H_3**); 2.20; 2.07; 2.06 (12H, s, methyl C**H_3**); 2.02 (6H, s, methyl C**H_3**); 2.01 (6H, s, methyl C**H_3**); 1.88 (6H, s, methyl C**H_3**); 1.87 (6H, s, methyl C**H_3**).

^{13}C-NMR (100 MHz, **$CDCl_3$**, 298 K) *δ*ppm: 170.5, 170.3, 170.0, 169.9, 169.8, 169.8, 169.7 169.4, 169.1, 168.9 (**C**OCH_3); 152.3 (pyridyl C^2); 151.5 (pyrazole C^3); 149.6 (pyridyl C^6); 147.6, 147.5 (pyridyl C^4); 145.4, 145.3 (triazole C^4); 132.5 (naphthyl $C^{1/5}$ or $C^{9/10}$); 131.7 (naphthyl $C^{9/10}$ or $C^{1/5}$); 130.9 (pyrazole C^5); 127.3 (naphthyl $C^{2/6}$); 126.5 (naphthyl $C^{3/7}$); 124.3 (naphthyl $C^{4/8}$); 121.3, 121.2 (triazole C^5); 120.8 (pyridyl C^5); 118.3 (pyridyl C^3); 105.1 (pyrazole C^4); 86.2, 86.1 (galactose C^1); 85.7 (glucose C^1); 76.3; 76.2; 75.9; 75.1 (glucose C^5); 74.0 (galactose C^5); 72.6 (glucose C^3); 70.8, 70.7 (O-**C**H_2-CNR + galactose C^3); 70.4 (glucose C^2); 68.2 (galactose C^2); 67.7 (glucose C^4); 67.7; 67.3; 66.9 (galactose C^4); 63.9, 63.9 (pyridyl-**C**H_2O); 61.5 (glucose C^6); 61.2 (galactose C^6); 54.6 (pyridyl-**C**H_2N); 29.7; 26.9; 20.7; 20.6; 20.5; 20.5; 20.3; 20.2 (**C**H_3's)

ν_{max}/cm^{-1}: 2923w, 2860w, 1745s, 1607m, 1366m, 1211s, 1090m, 1037s.

L15-3SL-Ac-Me (65)

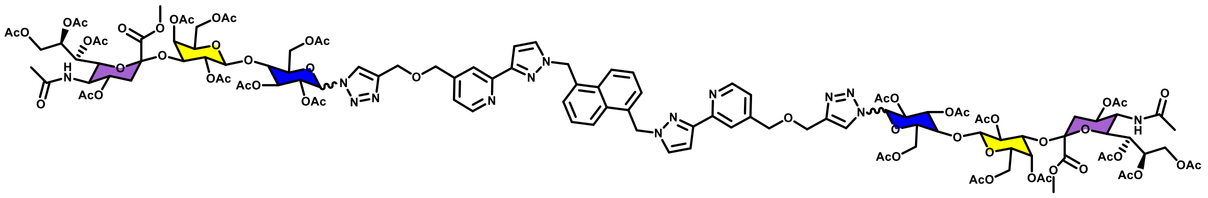

Prepared via the same CuAAC procedure as the monosaccharide-pendant ligands: 58 mg of **L15CC** used. Yield: 91 mg, 33 μmol, 33%.

¹H-NMR (400 MHz, **CDCl₃**, 298 K) δppm: 8.58 (2H, d, J = 4.9 Hz, pyridyl H^6); 8.01 (2H, d, 8.4 Hz, naphthyl $H^{4/8}$); 7.92 (2H, s, pyridyl H^3); 7.88 (1H, s, triazole H^5), 7.84 (1H, s, triazole H^5); 7.45 (2H, t, J = 7.5 Hz, naphthyl $H^{3/7}$); 7.30 (2H, d, J = 6.6 Hz, naphthyl $H^{2/6}$); 7.23-7.18 (4H, m, pyrazole H^5 + pyridyl H^5); 6.83 (2H, s, pyrazole H^4); 5.86 (4H, t, J = 9.1 Hz, glucose H^1 and galactose H^1); 5.84 (4H, s, pyridyl-C**H₂**N); 5.53 (4H, t, J = 9.6 Hz, galactose H^{2+4}); 5.45-5.34 (4H, m, glucose H^{2+3}); 5.25-5.19 (4H, m, glucose H^4 and galactose H^3); 4.73 (2H, s, pyridyl-C**H₂**O); 4.72 (2H, s, pyridyl-C**H₂**O); 4.62 (4H, s, O-C**H₂**-CNR); 4.30-4.10 (10H, m, glucose H^{6a+6b} and galactose $H^{6a+6b+5}$); 4.03-3.97 (2H, m, glucose H^5); 3.80 (6H, s, COO**Me**); 2.23, 2.19 (6H, s, methyl C**H₃**); 2.18; 2.04, 2.03 (12H, s, methyl C**H₃**); 2.00 (6H, s, methyl C**H₃**); 1.99 (6H, s, methyl C**H₃**); 1.98 (6H, s, methyl C**H₃**); 1.86 (6H, s, methyl C**H₃**); 1.85 (6H, s, methyl C**H₃**).

¹³C-NMR (100 MHz, **CDCl₃**, 298 K) δppm: 170.5, 170.3, 169.9, 169.8, 169.4, 169.1, 168.9 (**C**OCH₃); 152.3 (pyridyl C^2); 151.6 (pyrazole C^3); 149.7 (pyridyl C^6); 147.6, 147.5 (pyridyl C^4); 145.5, 145.4 (triazole C^4); 132.3 (naphthyl $C^{1/5}$ or $C^{9/10}$); 131.7 (naphthyl $C^{9/10}$ or $C^{1/5}$); 130.9 (pyrazole C^5); 127.4 (naphthyl $C^{2/6}$); 126.5 (naphthyl $C^{3/7}$); 124.3 (naphthyl $C^{4/8}$); 121.3, 121.2 (triazole C^5); 120.7 (pyridyl C^5); 118.3 (pyridyl C^3); 105.0 (pyrazole C^4); 86.3 (galactose C^1); 85.8 (glucose C^1); 75.1 (glucose C^5); 74.0 (galactose C^5); 72.6 (glucose C^3); 70.8, 70.7 (O-**C**H₂-CNR + galactose C^3); 70.4 (glucose C^2); 68.0 (galactose C^2); 67.7 (glucose C^4); 67.3; 66.9 (galactose C^4); 63.9; 63.9 (pyridyl-**C**H₂O); 61.5 (glucose C^6); 61.2 (galactose C^6); 54.7 (pyridyl-**C**H₂N); 52.8 (COO**Me** via HSQC); 29.7; 21.0; 20.8; 20.7; 20.6; 20.5; 20.5; 20.3; 20.2 (**C**H₃'s)

High-resolution ES-MS: *m/z* observed, 1382.9678. Calculated for $C_{124}H_{150}N_{14}O_{58}^{2+}$ [*M* + 2H]$^{2+}$, 1382.9698.

$L^{15\text{-}6SL\text{-}Ac\text{-}Me}$ (66)

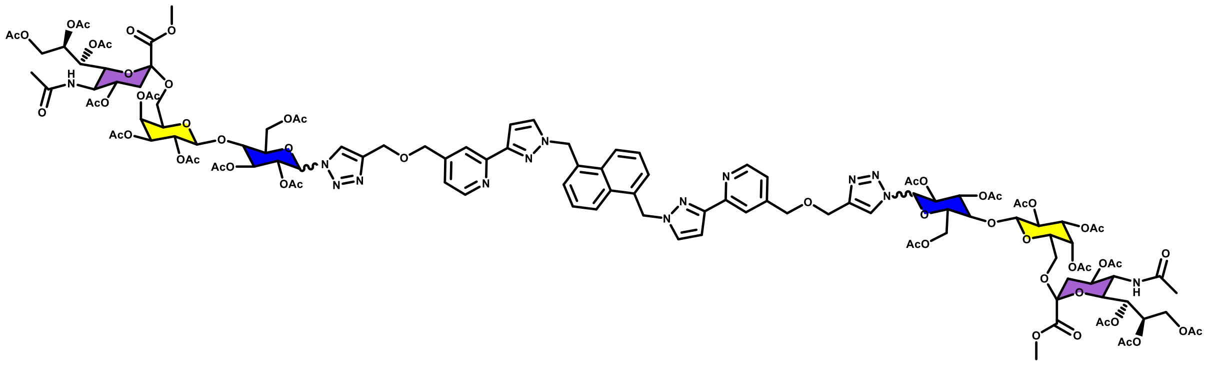

This was prepared via the same CuAAC procedure as used for the monosaccharide-pendant ligands: 66 mg of **L^{15CC}** (0.114 mmol) used. Yield: 100 mg, 36 μmol, 32%.

^{1}H-NMR (400 MHz, **$CDCl_3$**, 298 K) *δ*ppm: 8.58 (2H, d, J = 4.9 Hz, pyridyl H^6); 8.01 (2H, d, 8.3 Hz, naphthyl $H^{4/8}$); 7.91 (2H, s, pyridyl H^3); 7.87 (1H, d, J = 5.7 Hz, triazole H^5), 7.83 (1H, s, triazole H^5); 7.45 (2H, t, J = 7.4 Hz, naphthyl $H^{3/7}$); 7.30 (2H, d, J = 6.9 Hz, naphthyl $H^{2/6}$); 7.23-7.18 (4H, m, pyrazole H^5 + pyridyl H^5); 6.84 (2H, d, J = 1.9 Hz, pyrazole H^4); 5.86 (4H, t, J = 9.0 Hz, glucose H^1 and galactose H^1); 5.84 (4H, s, pyridyl-C**H_2**N); 5.53 (4H, td, J = 11.5, 2.4 Hz, galactose H^{2+4}); 5.45-5.35 (4H, m, glucose H^{2+3}); 5.25-5.19 (4H, m, glucose H^4 and galactose H^3); 4.73 (2H, s, pyridyl-C**H_2**O); 4.72 (2H, s, pyridyl-C**H_2**O); 4.62 (4H, s, O-C**H_2**-CNR); 4.29-4.09 (10H, m, glucose H^{6a+6b} and galactose $H^{6a+6b+5}$); 4.01-3.97 (2H, m, glucose H^5); 3.80 (6H, s, COO**Me**); 3.40-3.28, 2.19 (6H, s, methyl C**H_3**); 2.18; 2.04, 2.03 (12H, s, methyl C**H_3**); 2.00 (6H, s, methyl C**H_3**); 1.99 (6H, s, methyl C**H_3**); 1.98 (6H, s, methyl C**H_3**); 1.86 (6H, s, methyl C**H_3**); 1.85 (6H, s, methyl C**H_3**).

^{13}C-NMR (100 MHz, **$CDCl_3$**, 298 K) *δ*ppm: 170.5, 170.3, 170.0, 169.9, 169.8, 169.4, 169.1, 168.9 (**C**OCH_3); 152.4 (pyridyl C^2); 151.6 (pyrazole C^3); 149.7 (pyridyl C^6); 147.5, 147.5 (pyridyl C^4); 145.5, 145.4 (triazole C^4); 132.3 (naphthyl $C^{1/5}$ or $C^{9/10}$); 131.7 (naphthyl $C^{9/10}$ or $C^{1/5}$); 130.9 (pyrazole C^5); 127.4 (naphthyl $C^{2/6}$); 126.5 (naphthyl $C^{3/7}$); 124.3 (naphthyl $C^{4/8}$); 121.3, 121.2 (triazole C^5); 120.7 (pyridyl C^5); 118.3 (pyridyl C^3); 105.0 (pyrazole C^4); 86.3, 86.2 (galactose C^1); 85.8 (glucose C^1); 76.2, 75.9, 75.1 (glucose C^5); 74.0 (galactose C^5); 72.6 (glucose C^3); 70.8, 70.8, 70.7 (O-CH_2-CNR + galactose C^3); 70.4 (glucose C^2); 69.5, 68.3, 68.0 (galactose C^2); 67.7 (glucose C^4); 67.3; 66.9 (galactose C^4); 63.9; 63.9 (pyridyl-**C**H_2O); 61.5 (glucose C^6); 61.2 (galactose C^6); 54.7 (pyridyl-**C**H_2N); 53.2 (COO**Me** via HSQC); 29.7; 26.7 20.7; 20.6; 20.5; 20.5; 20.3; 20.2 (**C**H_3's)

High-resolution ES-MS: *m/z* observed, 1386.9253. $[M + 2H]^{2+}$

$L^{15\text{-}3SL}$ (67)

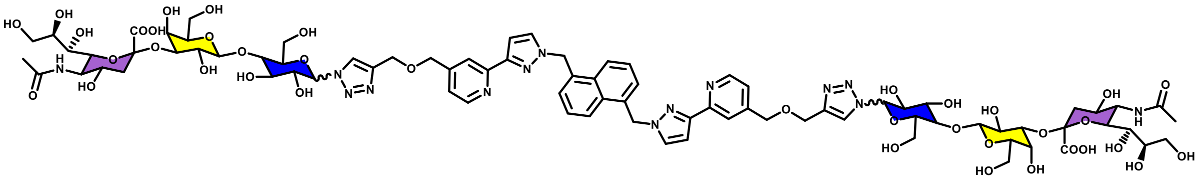

This was prepared via the same deprotection procedure as was used for the monosaccharide-pendant ligands: 55 mg of **$L^{15\text{-}3SL\text{-}Ac}$** used. Yield: 34 mg, 18 μmol, 89%.

^{1}H-NMR (300 MHz, **DMSO-*d*$_6$**, 298 K) δppm: 8.52 (2H, d, J = 4.9 Hz, pyridyl H^6); 8.39 (1H, s, triazole H^5), 8.32 (1H, s, triazole H^5); 8.22 (2H, d, J = 8.4 Hz, naphthyl H$^{4/8}$); 7.87 (2H, s, pyridyl H^3); 7.86 (2H, s, pyrazole H^5); 7.58 (2H, t, J = 8.0 Hz, naphthyl H$^{3/7}$); 7.29-7.27 (4H, m, naphthyl H$^{2/6}$ + pyridyl H^5); 6.86 (2H, d, J = 2.0 Hz, pyrazole H^4); 5.94 (4H, s, pyridyl-C**H$_2$**N); 5.58-5.47 (4H, m, glucose and galactose H^1); 4.97 (20H, brs, -O**H**); 4.65 (8H, s, pyridyl-C**H$_2$**O); 4.12-4.00 (2H, m, galactose H^2); 3.89-3.40 (20H, m, glucose H$^{2+3+4+6}$ and galactose H$^{3+4+5+6}$); 3.28-3.22 (2H, m, glucose H^5).

^{13}C-NMR (100 MHz, **DMSO-*d*$_6$**, 298 K) δppm: 167.4 (amide **C**OCH$_3$); 152.2 (pyridyl C^2); 151.5 (pyrazole C^3); 149.7 (pyridyl C^6); 148.5 (pyridyl C^4) 144.0, 143.9 (triazole C^4); 134.2 (naphthyl C$^{1/5}$ or C$^{9/10}$); 132.8 (pyrazole C^5), 131.4 (naphthyl C$^{9/10}$ or C$^{1/5}$); 126.8 (naphthyl C$^{2/6}$); 126.7 (naphthyl C$^{3/7}$); 124.3 (naphthyl C$^{4/8}$); 123.9 (triazole C^5); 123.6 (pyridyl C^5); 117.6 (pyridyl C^3); 105.0 (pyrazole C^4); 88.6 (galactose C^1); 88.0 (glucose C^1); 86.6; 79.9 (glucose C^4); 78.9 (galactose C^4); 77.4 (glucose C^3); 74.2 (galactose C^3); 73.1; 72.6 (glucose C^2); 70.2 (O-**C**H$_2$-CNR); 70.0 (glucose C^5); 69.8 (galactose C^2); 68.9 (galactose C^5); 63.7 (pyridyl-**C**H$_2$O); 61.2 (glucose C^6); 60.9 (galactose C^6); 53.8 (pyridyl-**C**H$_2$N).

ν_{max}/cm^{-1}: 3268br, 2916w, 1607m, 1560w, 1354m, 1333m, 1229m, 1090s, 1045s.

L$^{15\text{-}6SL}$ (68)

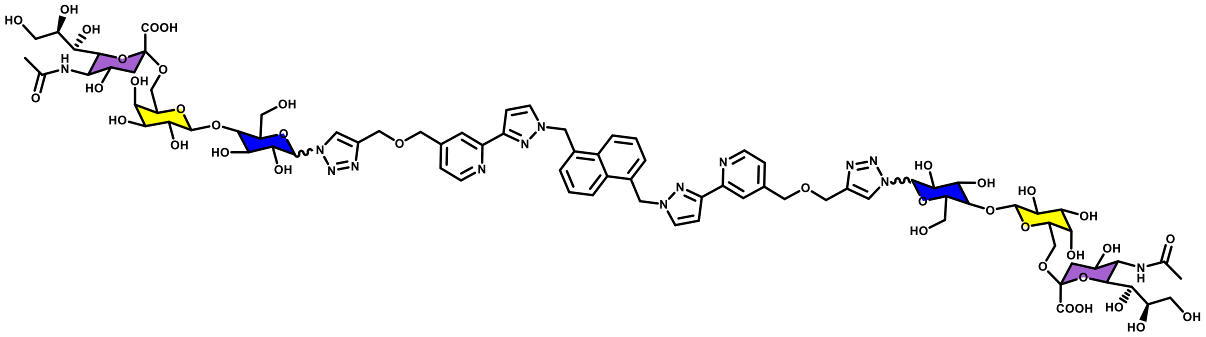

This was prepared via the same deprotection procedure as was used for the monosaccharide-pendant ligands: 90 mg of **L$^{15\text{-}6SL\text{-}Ac}$** used. Yield: 53 mg, 30 µmol, 92%.

^{1}H-NMR (400 MHz, **DMSO-*d*$_6$**, 298 K) *δ*ppm: 8.52 (2H, s, pyridyl H^6); 8.39 (3H, s, triazole H^5 + amide N**H**); 8.33 (1H, s, triazole H^5); 8.22 (2H, d, J = 8.4 Hz, naphthyl H$^{4/8}$); 7.89 (2H, s, pyridyl H^3); 7.86 (2H, s, pyrazole H^5); 7.58 (2H, t, J = 8.0 Hz, naphthyl H$^{3/7}$); 7.29-7.27 (4H, m, naphthyl H$^{2/6}$ + pyridyl H^5); 6.86 (2H, d, J = 2.0 Hz, pyrazole H^4); 5.94 (4H, s, pyridyl-C**H$_2$**N); 5.59-5.48 (4H, m, glucose and galactose H^1); 4.76 (20H, brs, -O**H**); 4.65 (8H, s, pyridyl-C**H$_2$**O); 4.12-4.00 (2H, m, galactose H^2); 3.89-3.37 (20H, m, glucose H$^{2+3+4+6}$ and galactose H$^{3+4+5+6}$); 3.28-3.23 (2H, m, glucose H^5).

^{13}C-NMR (100 MHz, **DMSO-*d*$_6$**, 298 K) *δ*ppm: 166.1 (amide **C**OCH$_3$ via HSQC); 152.2 (pyridyl C^2); 151.5 (pyrazole C^3); 149.7 (pyridyl C^6); 148.4 (pyridyl C^4) 144.1, 144.0 (triazole C^4); 134.2 (naphthyl C$^{1/5}$ or C$^{9/10}$); 132.8 (pyrazole C^5), 131.4 (naphthyl C$^{9/10}$ or C$^{1/5}$); 129.3; 126.8 (naphthyl C$^{2/6}$); 126.7 (naphthyl C$^{3/7}$); 124.3 (naphthyl C$^{4/8}$); 123.9 (triazole C^5); 123.6 (pyridyl C^5); 117.6 (pyridyl C^3); 115.9; 112.8; 112.1; 105.0 (pyrazole C^4); 88.6 (galactose C^1); 88.3; 88.0 (glucose C^1); 87.5; 80.5 (glucose C^4); 78.9 (galactose C^4); 78.2; 77.8; 77.4 (glucose C^3); 74.2 (galactose C^3); 73.7; 72.6 (glucose C^2); 70.2 (O-**C**H$_2$-CNR); 70.0 (glucose C^5); 69.8 (galactose C^2); 69.4; 68.9 (galactose C^5); 63.7 (pyridyl-**C**H$_2$O); 61.2 (glucose C^6); 60.9 (galactose C^6); 53.8 (pyridyl-**C**H$_2$N); 32.1 (amide CO**C**H$_3$)

ν_{max}/cm^{-1}: 3145w, 2922w, 1607m, 1516w, 1454w, 1352m, 1230m, 1090s, 1046s.

L$^{15\text{-}3SL\text{-}Me}$ (69)

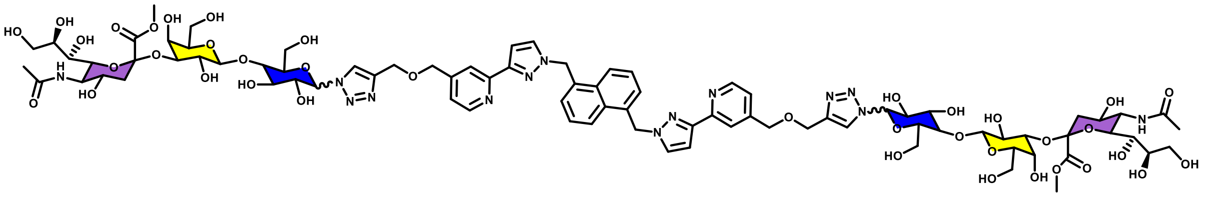

This was prepared via the same deprotection procedure as was used for the monosaccharide-pendant ligands: 91 mg of **L$^{15\text{-}3SL\text{-}Ac\text{-}Me}$** used. Yield: 56 mg, 29 µmol, 88%.

ν_{max}/cm^{-1}: 3336br, 3137w, 2925w, 2876w, 1611m, 1562w, 1413m, 1370m, 1331m, 1232m, 1092s, 1048s.

* *L$^{15\text{-}3SL\text{-}Me}$ was used directly in the next step, so an NMR sample was not prepared.*

Synthesis of tetrahedral cage ligands (3'- and 6'-Sialyllactose pendants)

**For all these ligands, no useful information was obtained from ESI-MS and MALDI-TOF-MS*

$L^{23\text{-}3SL\text{-}Ac}$ (70)

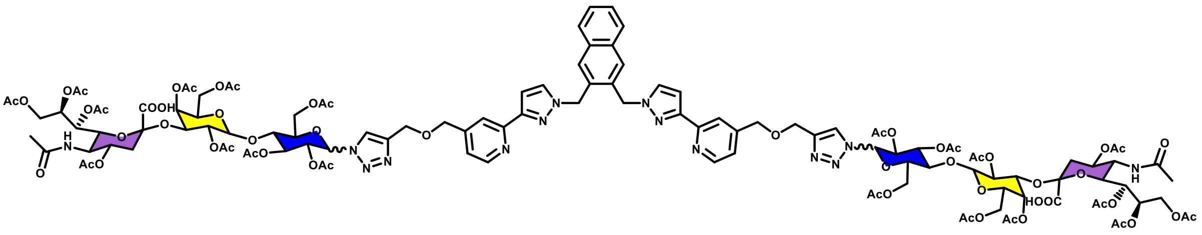

This was prepared via the same CuAAC procedure as was used for the cubic cage ligands: 71 mg of **L^{23CC}** used. Yield: 135 mg, 49 μmol, 40%.

^{1}H-NMR (400 MHz, **$CDCl_3$**, 298 K) *δ*ppm: 8.54 (2H, d, J = 4.8 Hz, pyridyl H^6); 7.88 (2H, s, pyridyl H^3); 7.87 (1H, s, triazole H^5); 7.85 (1H, s, triazole H^5); 7.72 (2H, dd, J = 6.1, 3.3 Hz, naphthyl $H^{5/8}$); 7.56 (2H, s, naphthyl $H^{1/4}$); 7.43 (2H, dd, J = 6.3, 3.2 Hz, napthyl $H^{6/7}$); 7.35 (2H, s, pyrazole H^5); 7.20 (2H, d, J = 4.9 Hz, pyridyl H^5); 6.95 (2H, s, pyrazole H^4); 5.88-5.81 (4H, m, glucose H^1 and galactose H^1); 5.53 (4H, s, pyridyl-C**H_2**N); 5.51-5.48 (4H, m, galactose H^{2+4}); 5.44-5.36 (4H, dt, 9.3, 7.3 Hz, glucose H^{2+3}); 5.22 (2H, d, J = 4.8 Hz, galactose H^3); 5.20 (2H, d, J = 9.8 Hz, glucose H^4); 4.69 (2H, s, pyridyl-C**H_2**O); 4.68 (2H, s, pyridyl-C**H_2**O); 4.57 (4H, s, O-C**H_2**-CNR); 4.28-4.06 (10H, m, glucose H^{6a+6b} and galactose $H^{6a+6b+5}$); 3.99-3.96 (2H, m, glucose H^5); 2.15 (6H, d, methyl C**H_3**); 2.11; 2.09; 2.05; 2.03 (6H, s, methyl C**H_3**); 2.01 (6H, s, methyl C**H_3**); 2.00 (6H, s, methyl C**H_3**); 1.97 (6H, s, methyl C**H_3**); 1.96 (6H, s, methyl C**H_3**); 1.82 (6H, s, methyl C**H_3**); 1.80 (6H, s, methyl C**H_3**).

^{13}C-NMR (100 MHz, **$CDCl_3$**, 298 K) *δ*ppm: 170.5, 170.3, 170.0, 169.9, 169.8, 169.8, 169.7, 169.5, 169.4, 169.1, 168.9 (**C**OCH_3); 151.8 (pyridyl C^2); 151.2 (pyrazole C^3); 149.0 (pyridyl C^6); 148.3, (pyridyl C^4); 145.3, 145.2 (triazole C^4); 133.0 (napthyl $C^{2/3}$); 132.1 (naphthyl $C^{5/10}$); 131.3 (pyrazole C^5); 129.1 (napthyl $C^{6/9}$); 127.7 (napthyl $C^{1/4}$); 126.9 (napthyl $C^{7/8}$); 121.3 (triazole C^5); 120.8 (pyridyl C^5); 118.4 (pyridyl C^3); 105.5 (pyrazole C^4); 98.4; 86.2, 86.0 (galactose C^1); 85.7 (glucose C^1); 76.3; 76.1; 75.9; 75.0 (glucose C^5); 74.0 (galactose C^5); 72.6 (glucose C^3); 70.8; 70.7; 70.6 (O-**C**H_2-CNR + galactose C^3); 70.4 (glucose C^2); 70.0; 69.5; 68.2; 68.0 (galactose C^2); 67.8, 67.7 (glucose C^4); 67.3; 66.9 (galactose C^4); 63.9, 63.9 (pyridyl-

CH2O); 61.9; 61.5 (glucose C[6]); 61.2 (galactose C[6]); 54.0 (pyridyl-**C**H2N); 48.0; 29.6, 27.0, 20.8, 20.7, 20.7, 20.5, 20.5, 20.2, 20.2, 20.1 (**C**H3's).

ν_{max}/cm^{-1}: 2962w, 1744s, 1607m, 1367m, 1211s, 1089m, 1036s.

L$^{23\text{-}6SL\text{-}Ac}$ (71)

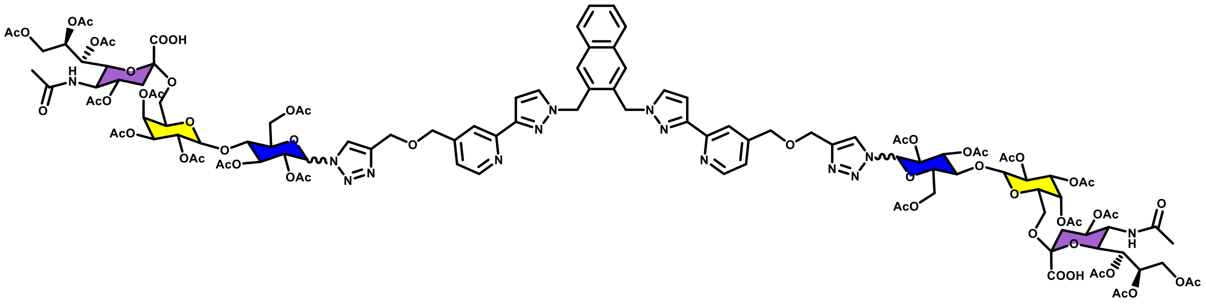

This was prepared via the same CuAAC procedure as was used for the cubic cage ligands: 78 mg of **L^{23CC}** used. Yield: 135 mg, 43 μmol, 32%.

^{1}H-NMR (400 MHz, **CDCl$_3$**, 298 K) *δ*ppm: 8.55 (2H, d, J = 4.4 Hz, pyridyl H^6); 7.87 (2H, s, pyridyl H^3); 7.85 (2H, s, triazole H^5); 7.73 (2H, dd, J = 6.1, 3.3 Hz, naphthyl H$^{5/8}$); 7.56 (2H, s, naphthyl H$^{1/4}$); 7.44 (2H, dd, J = 6.1, 3.2 Hz, napthyl H$^{6/7}$); 7.35 (2H, s, pyrazole H^5); 7.19 (2H, d, J = 4.6 Hz, pyridyl H^5); 6.90 (2H, s, pyrazole H^4); 5.85 (4H, t, J = 9.2 Hz, glucose H^1 and galactose H^1); 5.53 (4H, s, pyridyl-C**H$_2$**N); 5.53-5.49 (4H, m, galactose H^{2+4}); 5.45-5.36 (4H, dt, J = 9.5, 7.8 Hz, glucose H^{2+3}); 5.23 (2H, d, J = 6.6 Hz, galactose H^3); 5.20 (2H, d, J = 9.4 Hz, glucose H^4); 4.70 (2H, s, pyridyl-C**H$_2$**O); 4.69 (2H, s, pyridyl-C**H$_2$**O); 4.57 (4H, s, O-C**H$_2$**-CNR); 4.28-4.08 (10H, m, glucose H^{6a+6b} and galactose H$^{6a+6b+5}$); 4.00-3.96 (2H, m, glucose H^5); 2.16 (6H, d, methyl C**H$_3$**); 2.13; 2.06; 2.03 (12H, s, methyl C**H$_3$**); 2.01 (6H, s, methyl C**H$_3$**); 1.98 (6H, s, methyl C**H$_3$**); 1.97 (6H, s, methyl C**H$_3$**); 1.83 (6H, s, methyl C**H$_3$**); 1.81 (6H, s, methyl C**H$_3$**).

^{13}C-NMR (100 MHz, **CDCl$_3$**, 298 K) *δ*ppm: 170.5, 170.3, 170.0, 169.9, 169.8, 169.8, 169.7, 169.5, 169.4, 169.0, 168.9 (**C**OCH$_3$); 152.2 (pyridyl C^2); 151.8 (pyrazole C^3); 149.6 (pyridyl C^6); 147.6, (pyridyl C^4); 145.4, 145.3 (triazole C^4); 133.0 (napthyl C$^{2/3}$); 132.2 (naphthyl C$^{5/10}$); 131.2 (pyrazole C^5); 129.0 (napthyl C$^{6/9}$); 127.7 (napthyl C$^{1/4}$); 126.9 (napthyl C$^{7/8}$); 121.3 (triazole C^5); 120.7 (pyridyl C^5); 118.3 (pyridyl C^3); 105.2 (pyrazole C^4); 86.2, 86.1 (galactose C^1); 85.7 (glucose C^1); 76.2; 75.9; 75.1 (glucose C^5); 74.0 (galactose C^5); 72.6 (glucose C^3); 70.8, 70.7 (O-**C**H$_2$-CNR + galactose C^3); 70.4 (glucose C^2); 68.0 (galactose C^2); 67.7, 67.7 (glucose C^4); 67.3; 66.9 (galactose C^4); 63.9, 63.9 (pyridyl-**C**H$_2$O); 61.5 (glucose C^6); 61.2 (galactose C^6); 54.0 (pyridyl-**C**H$_2$N); 26.9, 20.9, 20.7, 20.5, 20.5, 20.2, 20.2 (**C**H$_3$'s).

ν_{max}/cm^{-1}: 2970w, 1743s, 1608m, 1366m, 1210s, 1087m, 1037s.

L$^{23\text{-}3SL}$ (72)

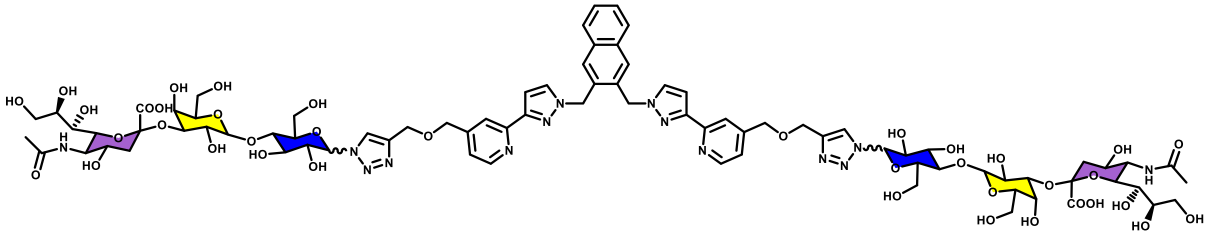

This was prepared via the same deprotection procedure as was used for the monosaccharide ligands: 135 mg of **L$^{23\text{-}3SL\text{-}Ac}$** used. Yield: 81.8 mg, 43 µmol, 88%.

ν_{max}/cm^{-1}: 3284br, 3146w, 2920w, 2862w, 1635w, 1609m, 1559w, 1456m, 1330m, 1230m, 1090s, 1046s.

L$^{23\text{-}6SL}$ (73)

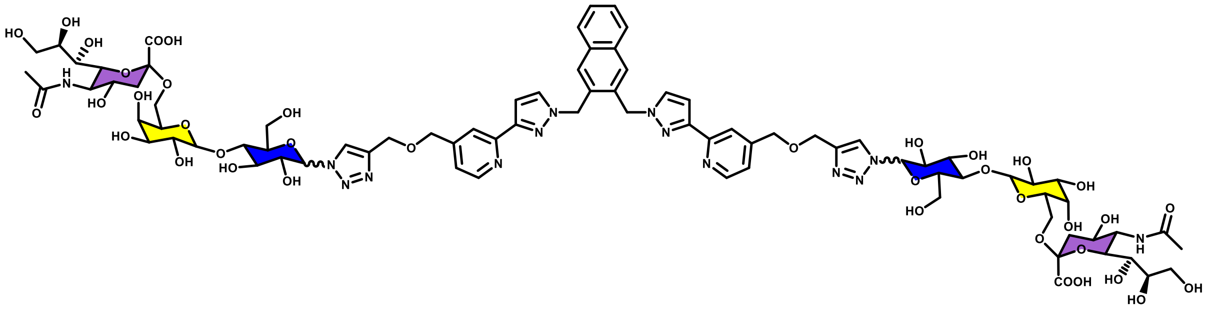

This was prepared via the same deprotection procedure as was used for the monosaccharide ligands: 135 mg of **L$^{23\text{-}6SL\text{-}Ac}$** used. Yield: 87.6 mg, 46 µmol, 94%.

ν_{max}/cm^{-1}: 3257br, 3141w, 2921w, 2858w, 2824w, 1706, 1589w, 1454m, 1348m, 1230m, 1090s, 1046s.

**$L^{23\text{-}3SL}$ and $L^{23\text{-}6SL}$ were used directly in the next step, so NMR samples were not prepared.*

Synthesis of sialyllactose-pendant cubic cages

[$Co_8(L^{15\text{-}3SL\text{-}Ac})_{12}(BF_4)_{16}$] [$Co_8^{3SL\text{-}Ac}$] (74)

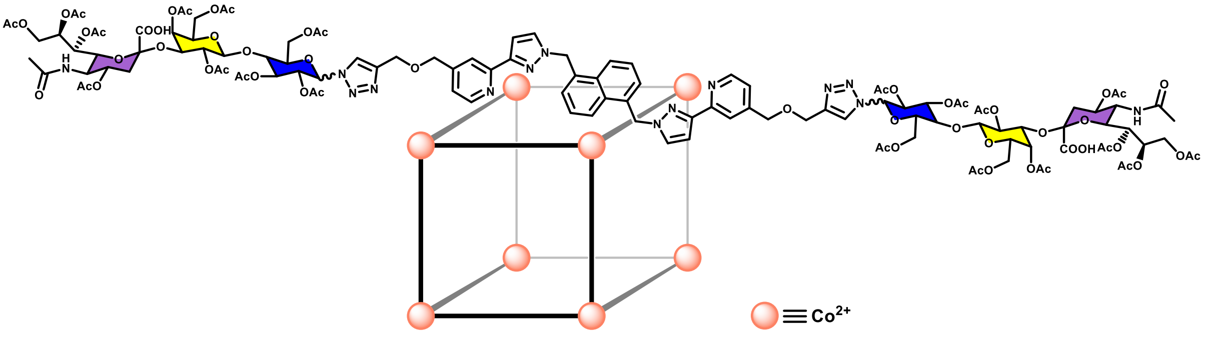

Same procedure as for **32** and **33** using ligand **63**. Yield: 40 mg, 77%.

[$Co_8(L^{15\text{-}3SL})_{12}(BF_4)_{16}$] [$Co_8^{3SL}$] (75)

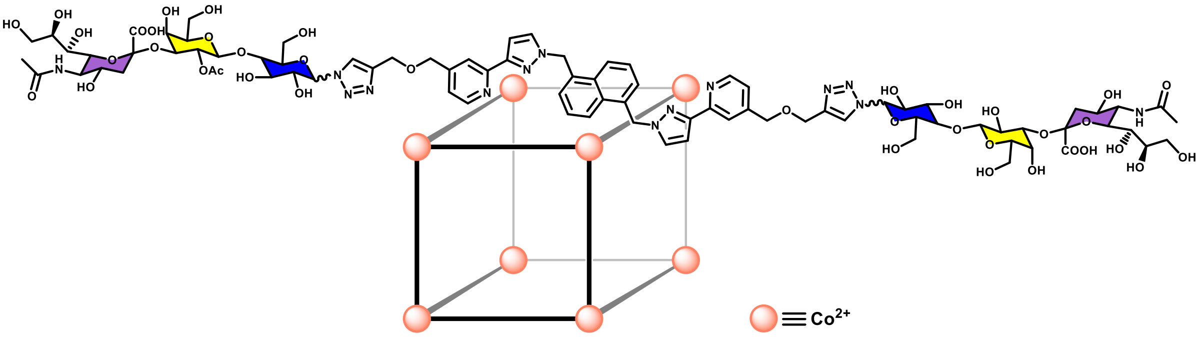

Same procedure as for **34** and **35** using ligand **67**. Yield: 34 mg, 85%

[$Co_8(L^{15\text{-}6SL})_{12}(BF_4)_{16}$] [$Co_8^{6SL}$] (76)

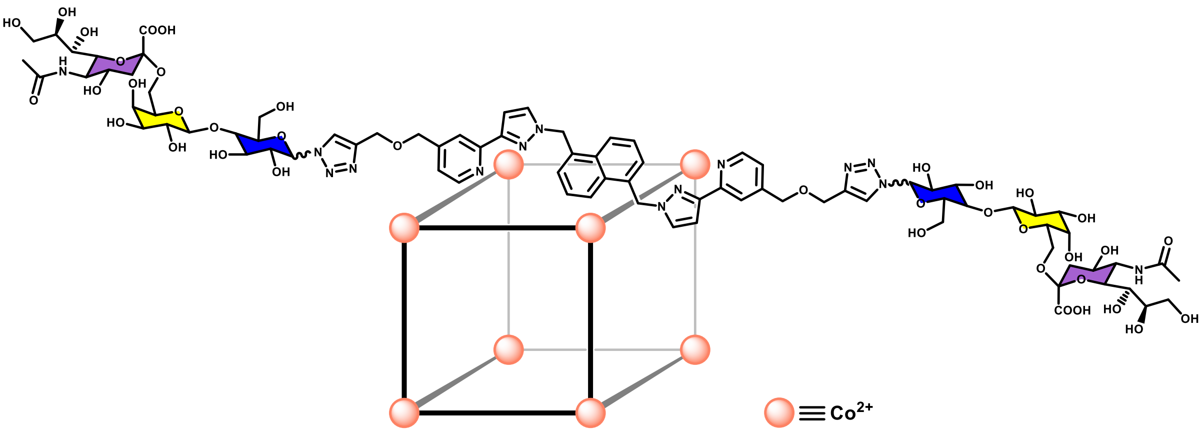

Same procedure as for **34** and **35** using ligand **68**. Yield: 45 mg, 88%.

$[Co_8(L^{15\text{-}3SL\text{-}Me})_{12}(BF_4)_{16}]$ $[Co_8{}^{3SL\text{-}Me}]$ (77)

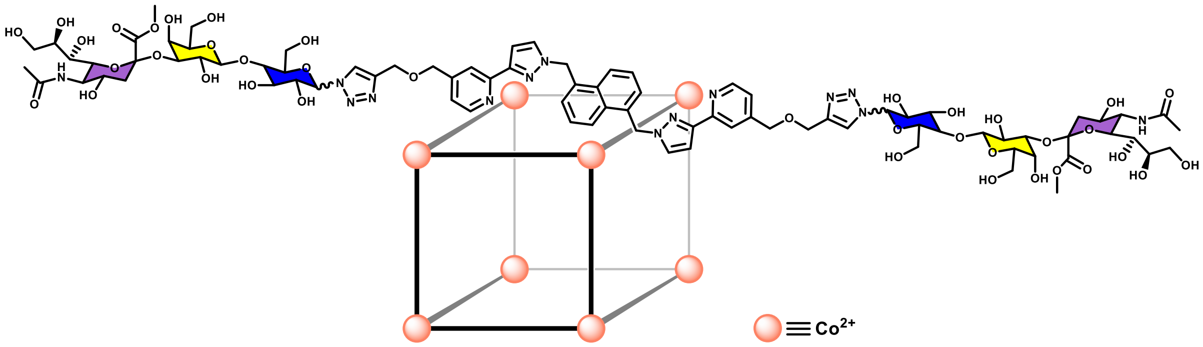

Same procedure as for **34** and **35** using ligand **69**. Yield: 46 mg, 87%.

Synthesis of sialyllactose-pendant tetrahedral cages

[$Co_4(L^{23\text{-}3SL})_6(BF_4)_8$] [$Co_4{}^{3SL}$] (78)

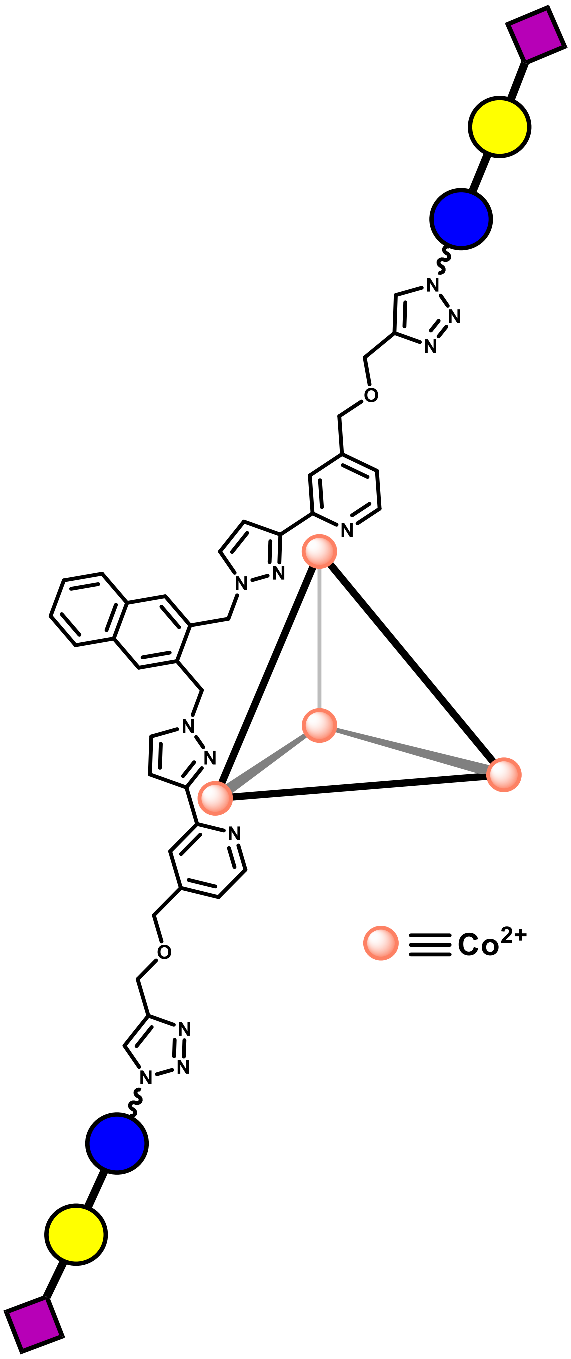

Same procedure as for **34** and **35** using ligand **72**. Yield: 66 mg, 61%

[$Co_4(L^{23\text{-}6SL})_6(BF_4)_8$] [$Co_4^{6SL}$] (79)

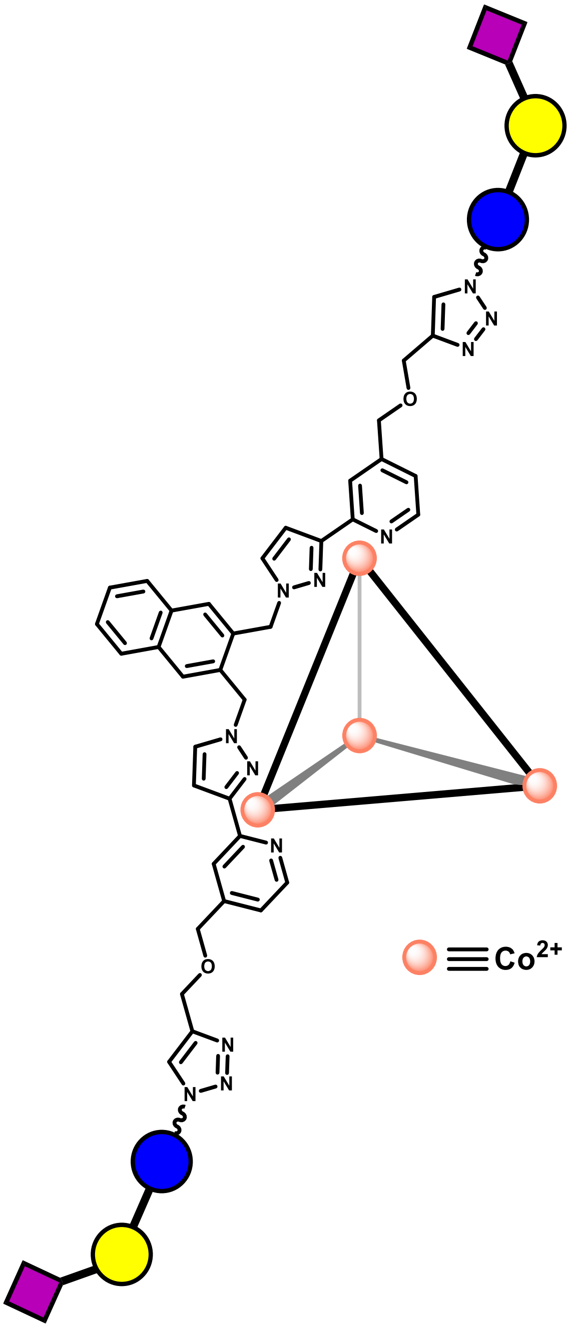

Same procedure as for **34** and **35** using ligand **73**. Yield: 72 mg, 67%

Selected NMR spectra Figures

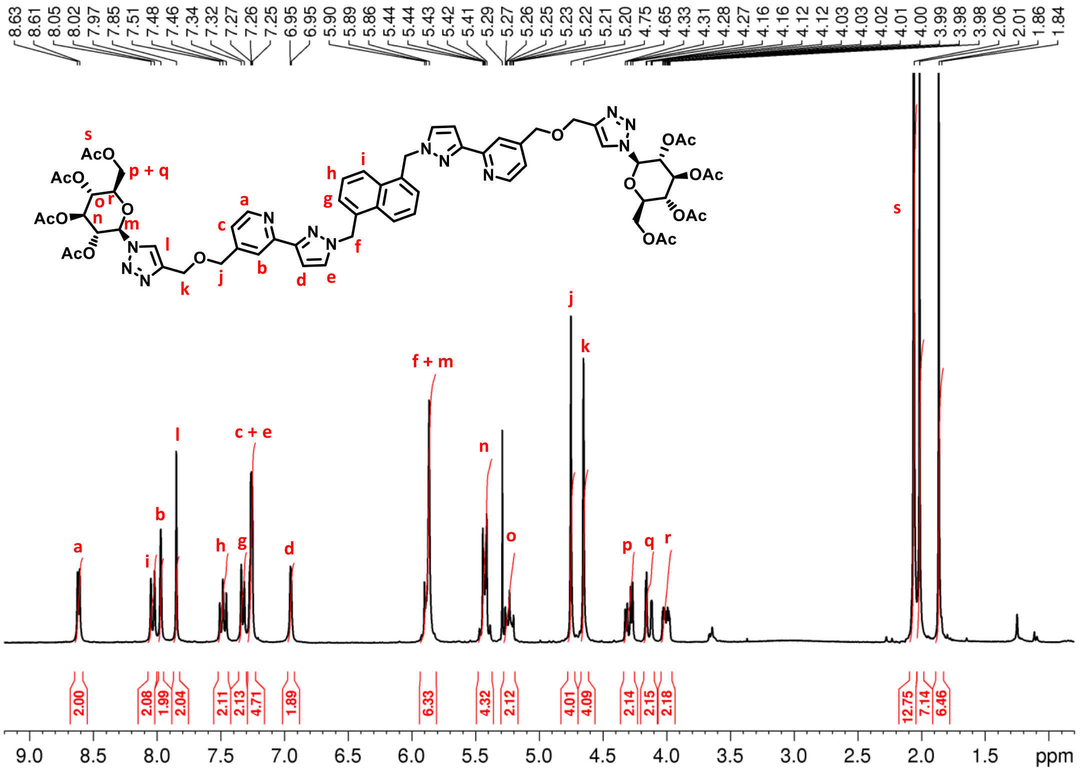

Figure S87. ^{1}H NMR spectrum (300 MHz, $CDCl_3$, 298 K) of $\mathbf{L^{15\text{-}Glu\text{-}Ac}}$

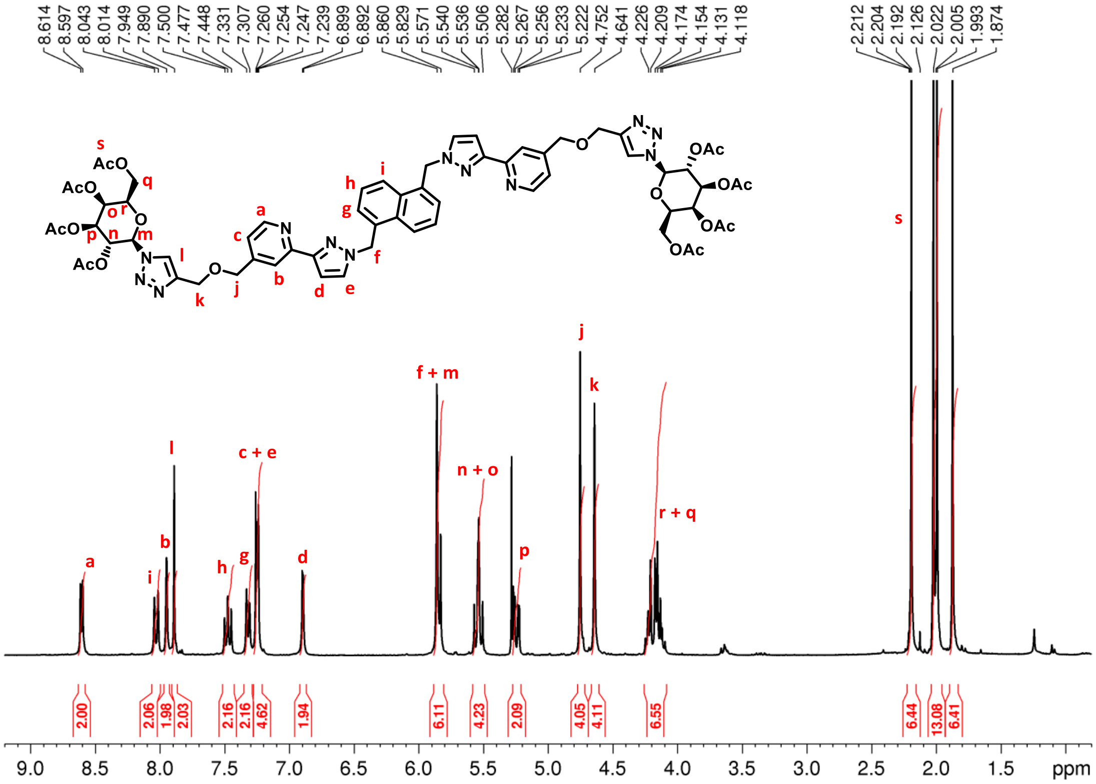

Figure S88. ^{1}H NMR spectrum (300 MHz, $CDCl_3$, 298 K) of $\mathbf{L^{15\text{-}Gal\text{-}Ac}}$

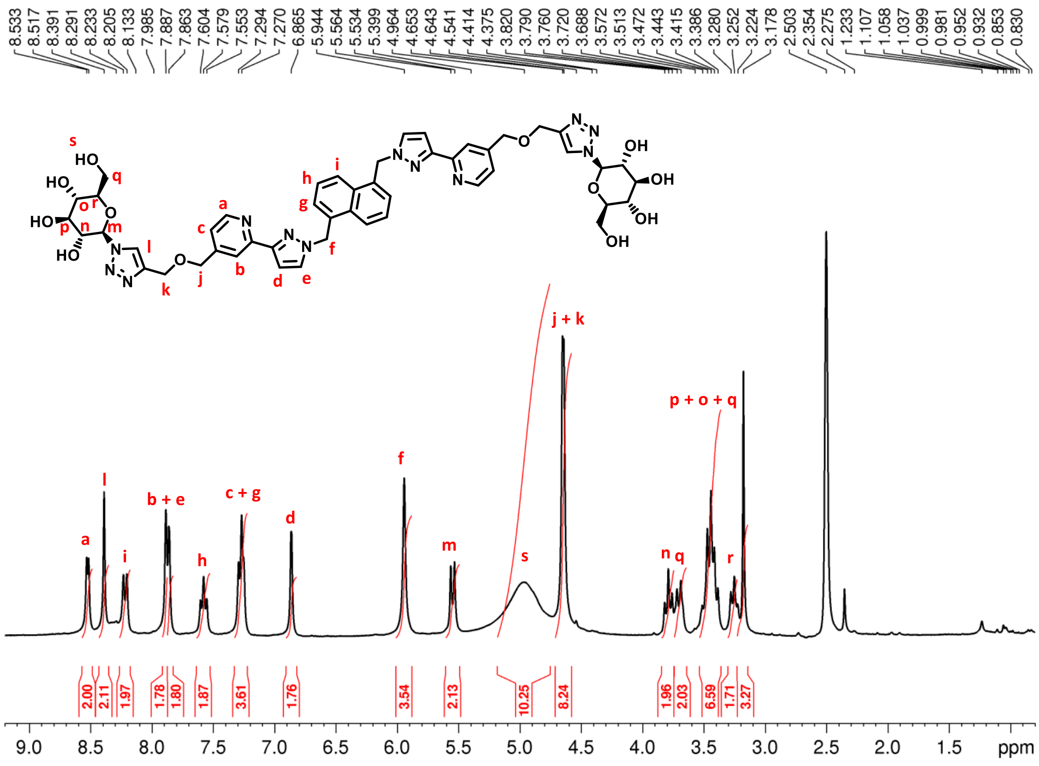

Figure S89. ^{1}H NMR spectrum (300 MHz, DMSO-d_6, 298 K) of **L$^{15\text{-}Glu}$**

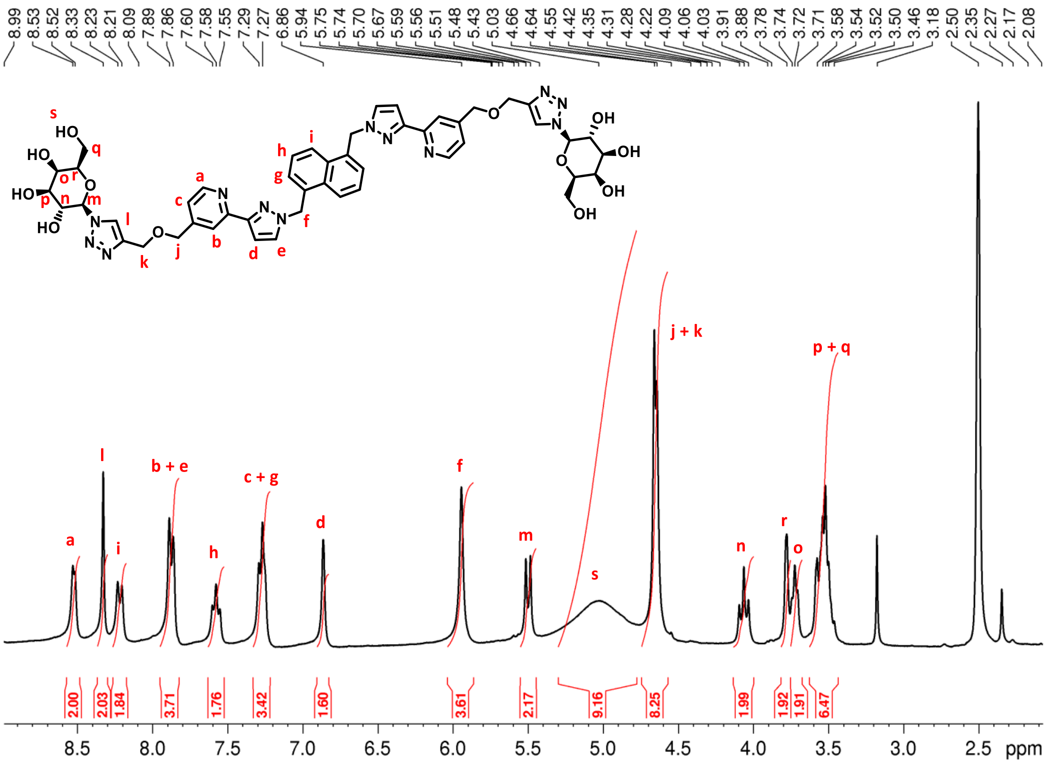

Figure S90. ^{1}H NMR spectrum (300 MHz, DMSO-d_6, 298 K) of **L$^{15\text{-}Gal}$**

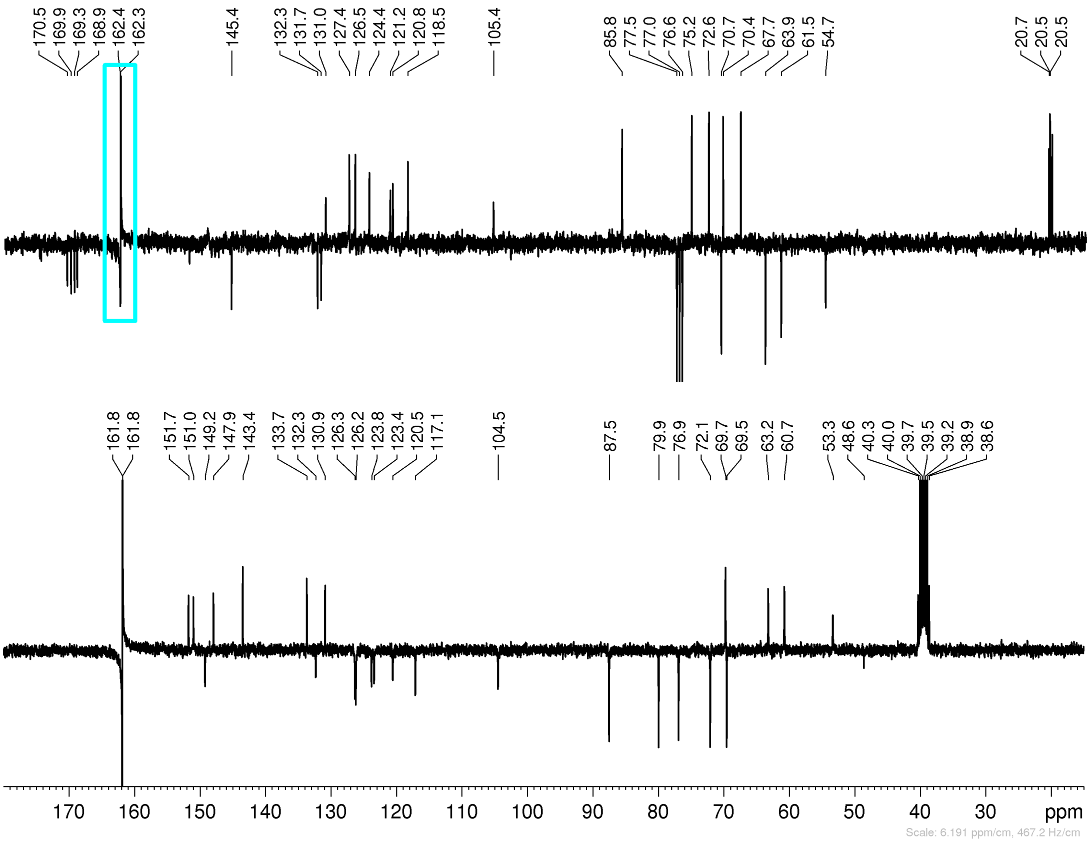

Figure S91. ^{13}C NMR (75 MHz, 298 K) spectra of $\mathbf{L^{15\text{-}Gal\text{-}Ac}}$ ($CDCl_3$) vs $\mathbf{L^{15\text{-}Gal}}$ (DMSO-d_6). Highlighted area shows a background peak associated with this particular NMR instrument which can be ignored.

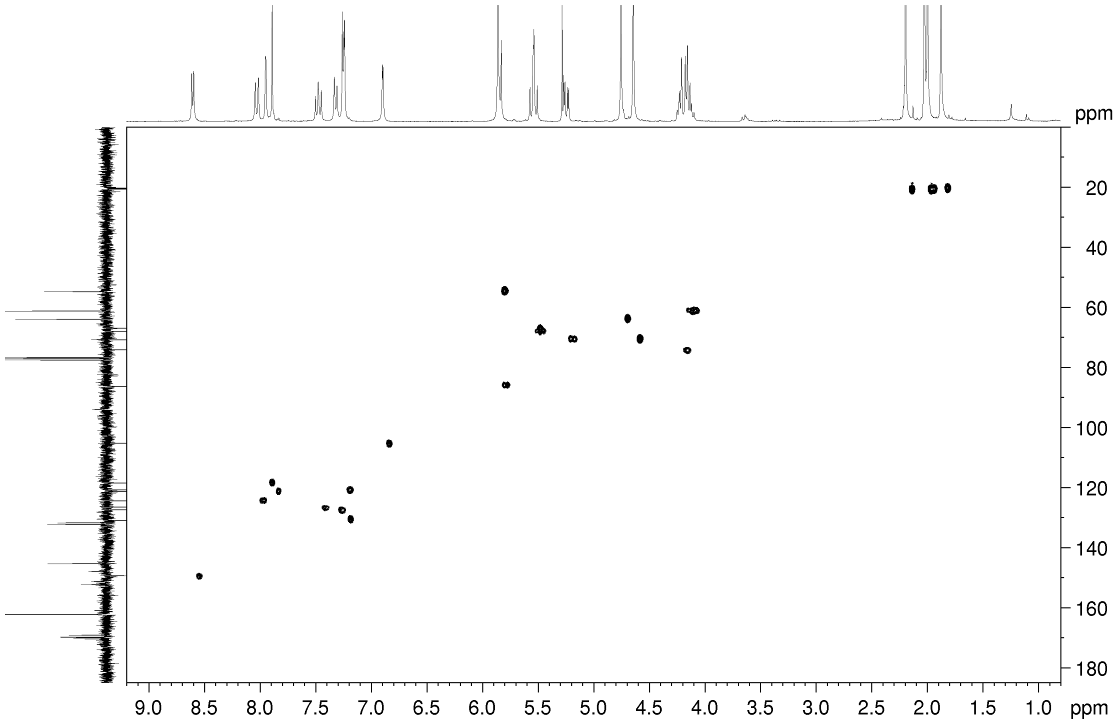

Figure S92. 2D-HSQC NMR spectra ($CDCl_3$) of $\mathbf{L^{15\text{-}Gal\text{-}Ac}}$

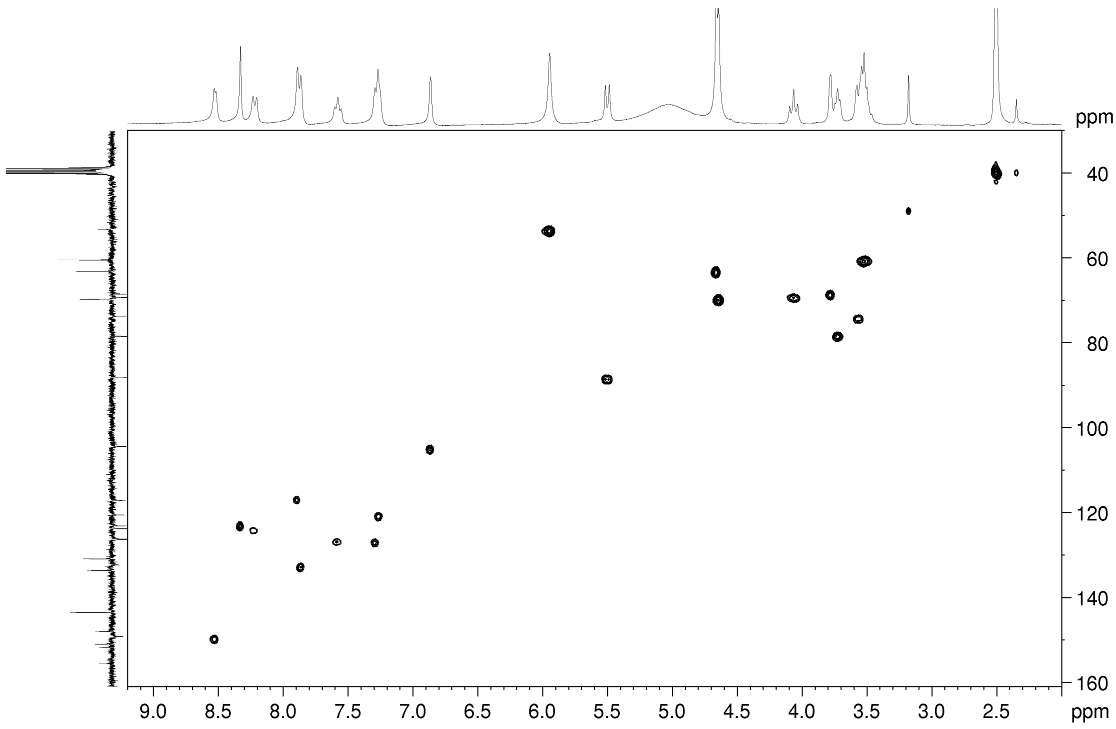

Figure S93. 2D-HSQC NMR spectra (DMSO-d_6) of **L$^{15\text{-Gal}}$**

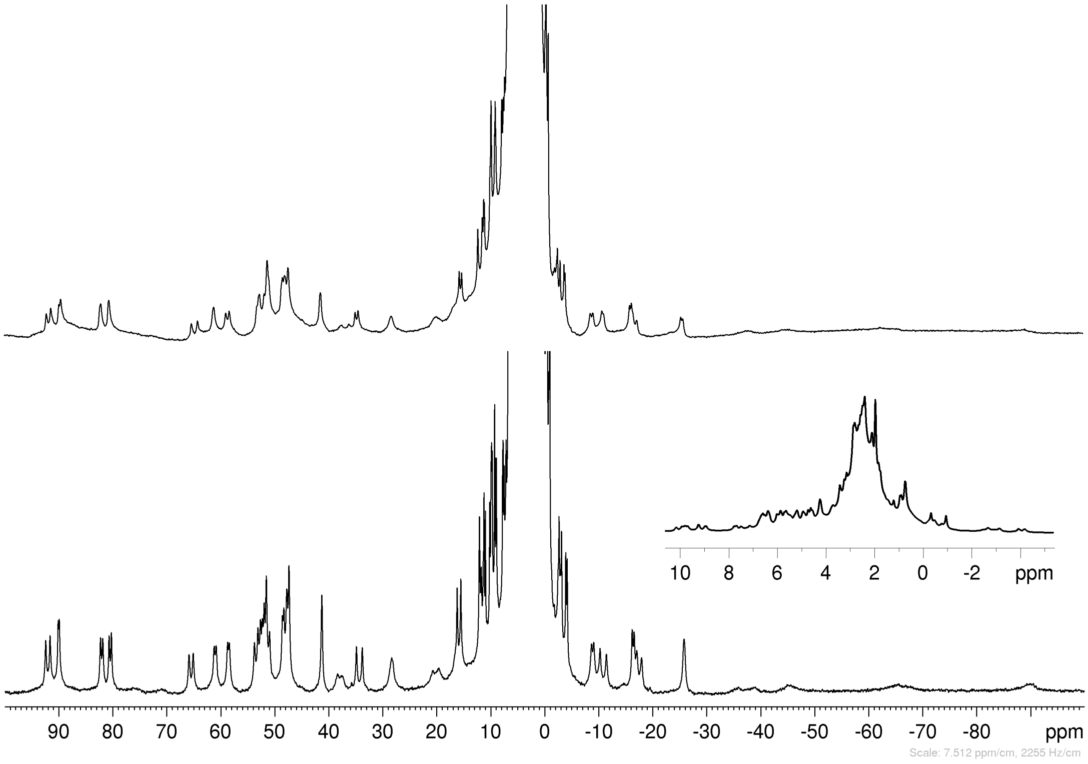

Figure S94. (Top to bottom) ^{1}H NMR spectra (300 MHz, CD_3CN, 298 K) of **[$Co_8(L^{15\text{-}Gal\text{-}Ac})_{12}(BF_4)_{16}$]** vs **[$Co_8(L^{15\text{-}Glu\text{-}Ac})_{12}(BF_4)_{16}$]**. Inset: expansion of **$Co_8^{Glu\text{-}Ac}$** at 10 ppm to -5 ppm.

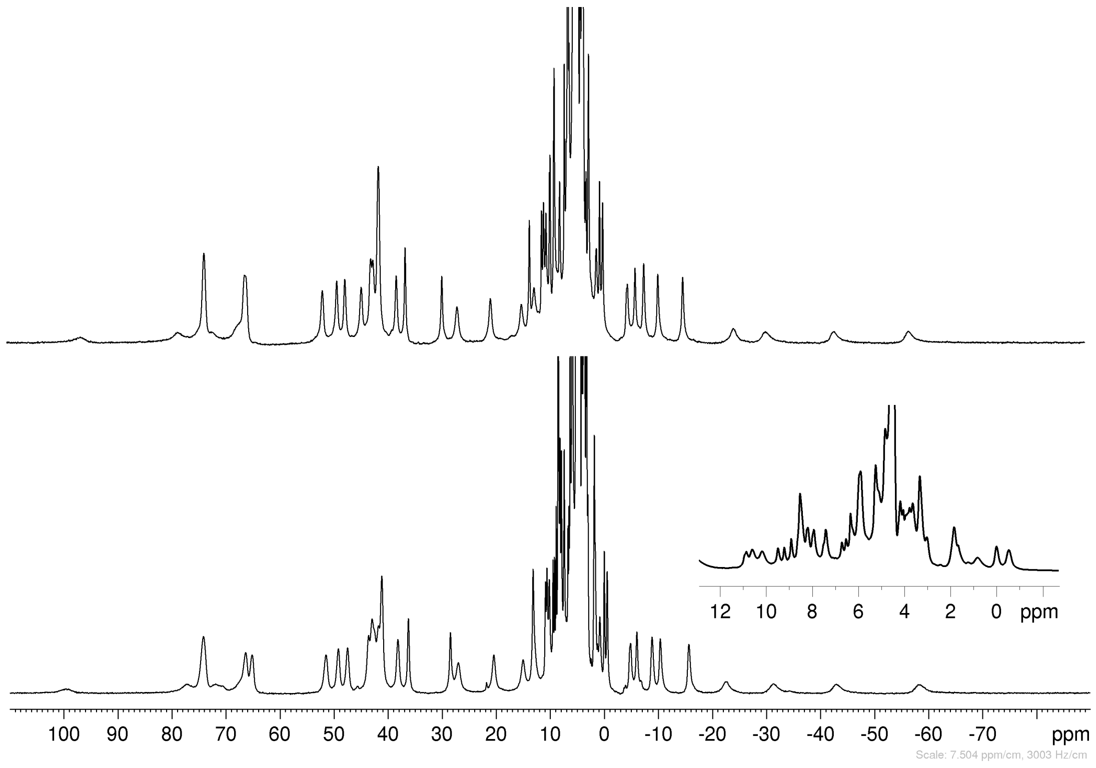

Figure S95. (Top to bottom) ^{1}H NMR spectra (300 MHz, D_2O, 363K) of **[$Co_8(L^{15\text{-}Gal})_{12}(BF_4)_{16}$]** vs **[$Co_8(L^{15\text{-}Glu})_{12}(BF_4)_{16}$]**. Zoom in of **$Co_8^{Glu}$** at 10 ppm to -5 ppm.

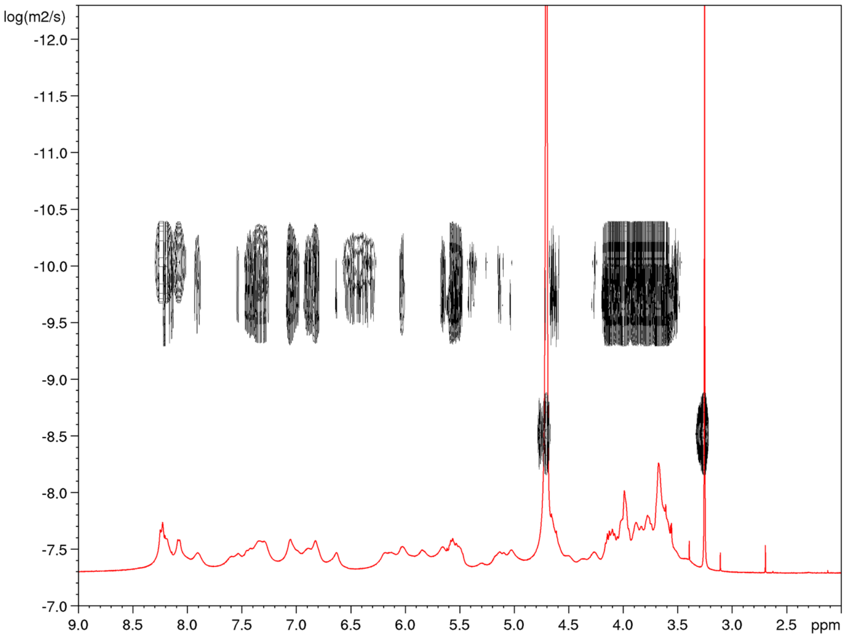

Figure S96. 2D-DOSY NMR spectra (500 MHz, D_2O) of the **$[Zn_8(L^{15\text{-}Gal})_{12}(BF_4)_{16}]$** cubic cage at 298K, which confirms a single species is present.

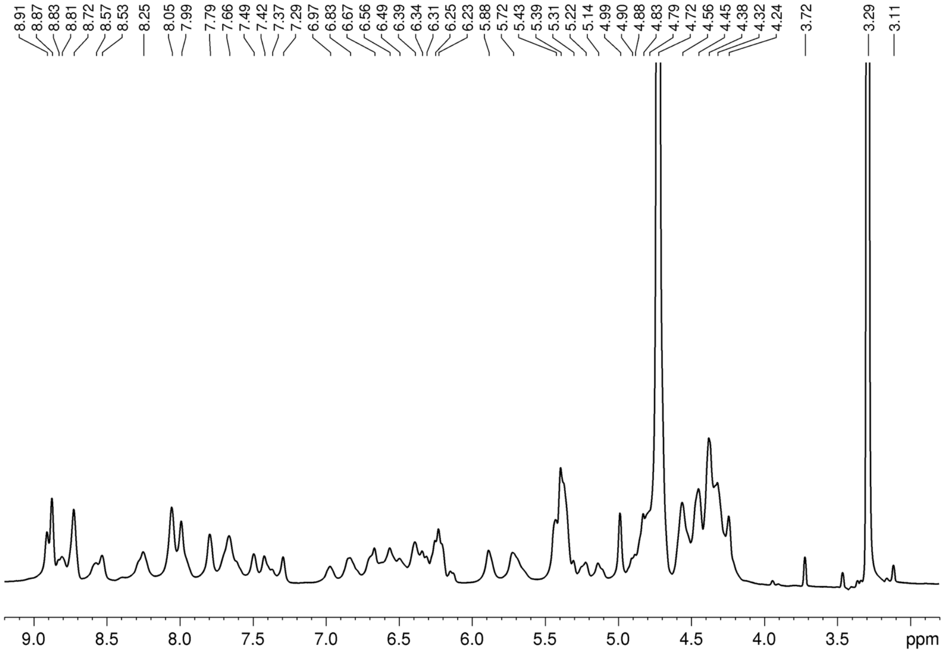

Figure S97. ^{1}H NMR spectrum (400 MHz, D_2O) of the **$[Zn_8(L^{15\text{-}Gal})_{12}(BF_4)_{16}]$** cubic cage at 363K, with the high temperature slightly reducing the broadness of the peaks.

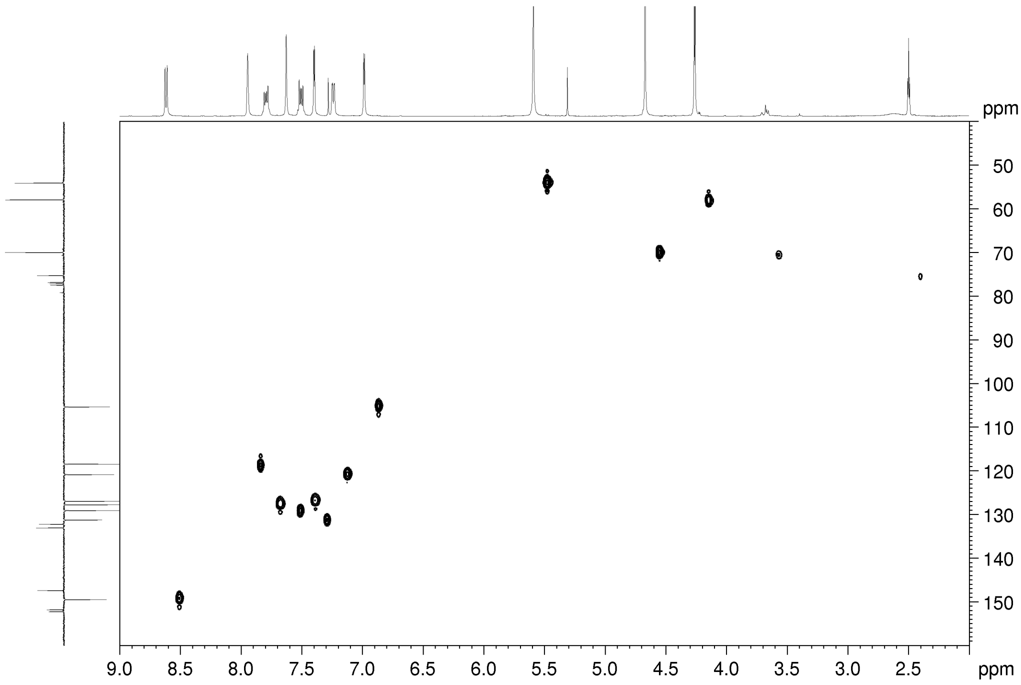

Figure S98. 2D-HSQC NMR spectra ($CDCl_3$) of **L^{23CC}**.

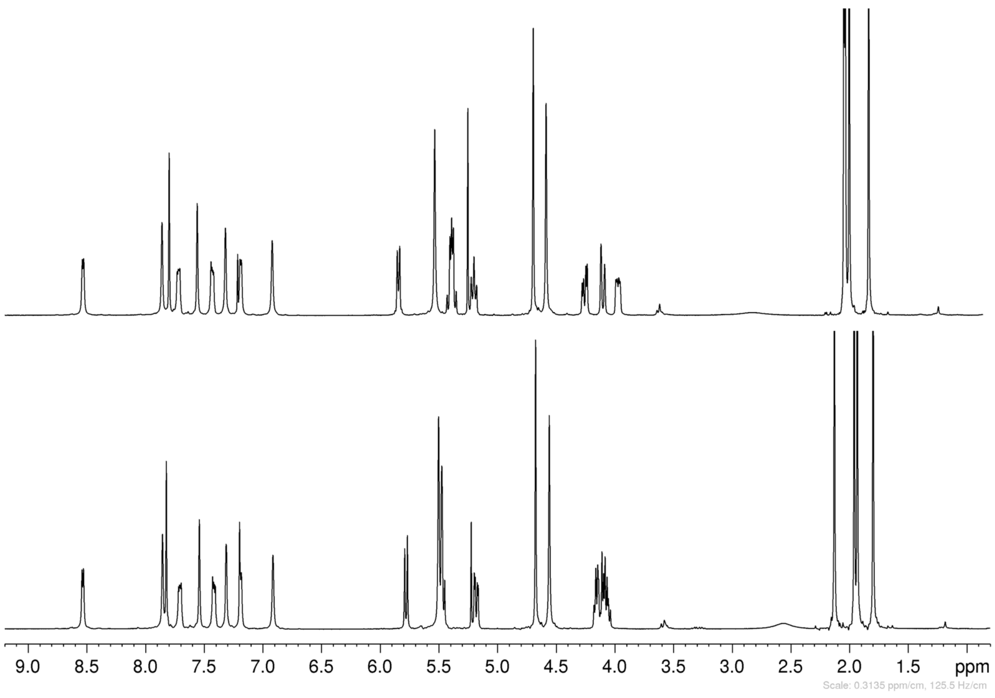

Figure S99. (Top to bottom) ^{1}H NMR spectra (400 MHz, $CDCl_3$, 298 K) of **L$^{23\text{-}Glu\text{-}Ac}$** and **L$^{23\text{-}Gal\text{-}Ac}$**

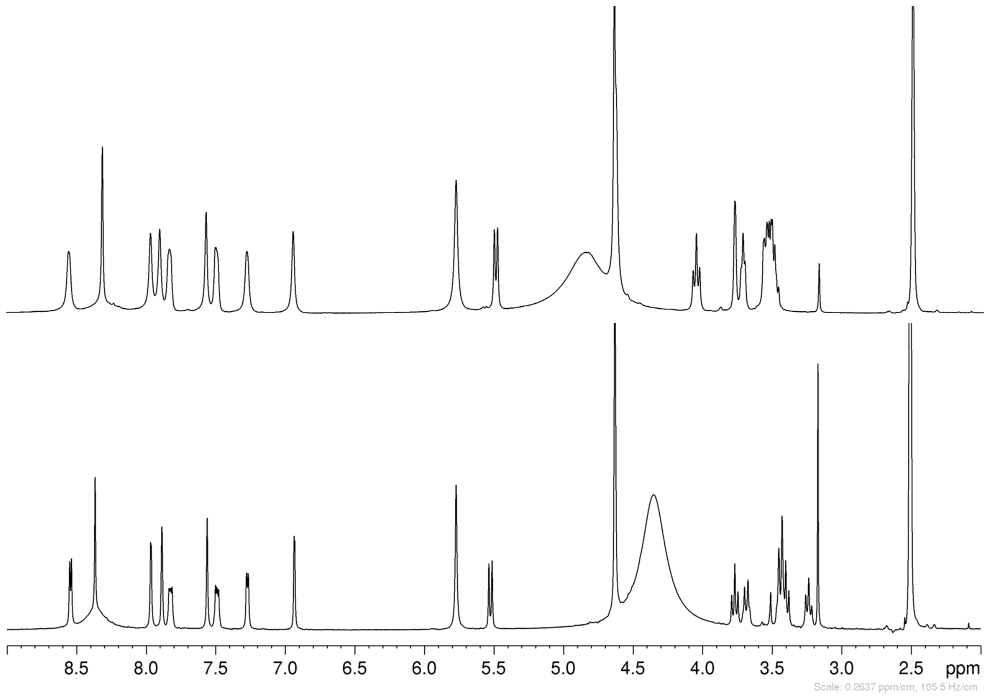

Figure S100. (Top to bottom) ^{1}H NMR spectra (400 MHz, DMSO-d_6, 298 K) of $\mathbf{L^{23\text{-}Gal}}$ and $\mathbf{L^{23\text{-}Glu}}$

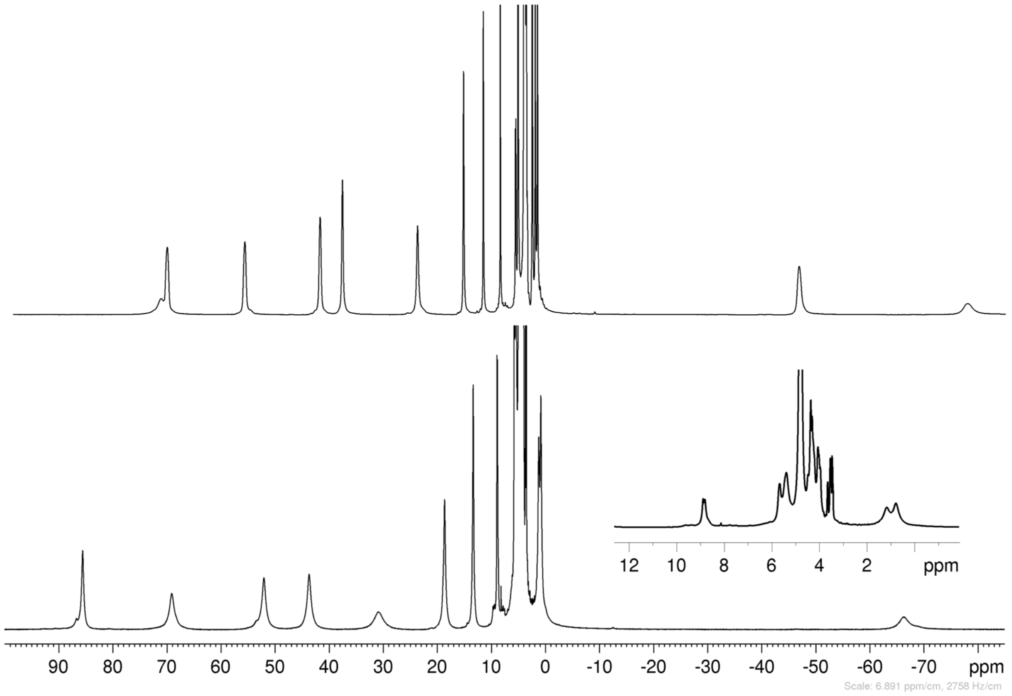

Figure S101. (Top to bottom) ^{1}H NMR spectra (400 MHz, D_2O) of $\mathbf{[Co_4(L^{23\text{-}Glu})_6(BF_4)_8]}$ and $\mathbf{[Co_4(L^{23\text{-}Gal)})_6(BF_4)_8]}$ at 363K. Zoom in of $\mathbf{Co_4^{Gal}}$ at 12 ppm to -2 ppm.

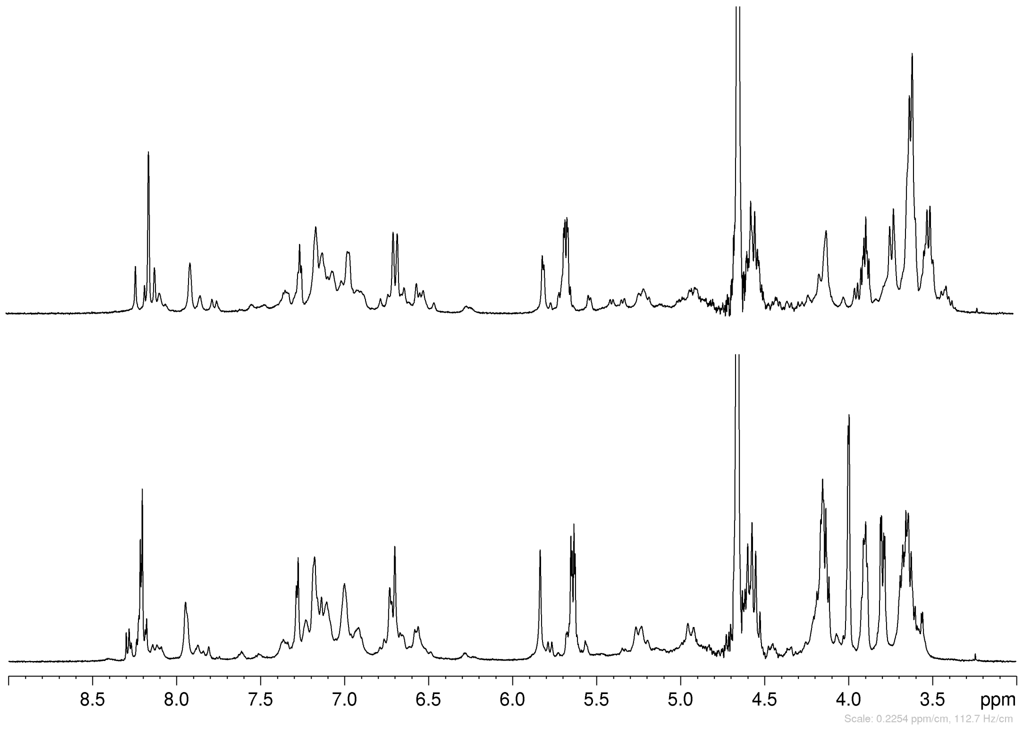

Figure S102. (Top to bottom) ^{1}H NMR spectra (500 MHz, D_2O, 298 K) of **[Zn$_4$(L$^{23\text{-Glu}}$)$_6$(BF$_4$)$_8$]** and **[Zn$_4$(L$^{23\text{-Gal)}}$)$_6$(BF$_4$)$_8$]**.

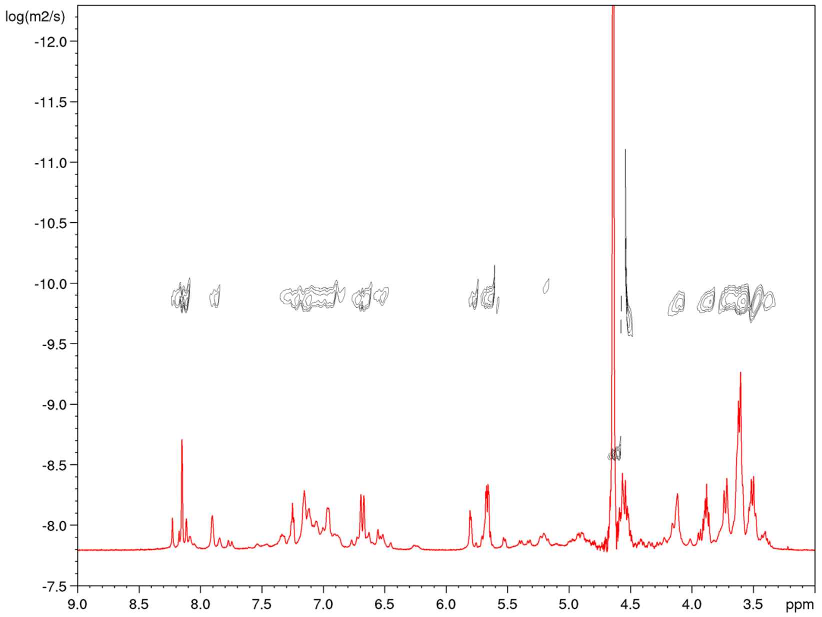

Figure S103. 2D-DOSY NMR spectra (500 MHz, D_2O) of the **[Zn$_4$(L$^{23\text{-Glu}}$)$_6$(BF$_4$)$_8$]** tetrahedral cage at 298K, which confirms that a single species is present.

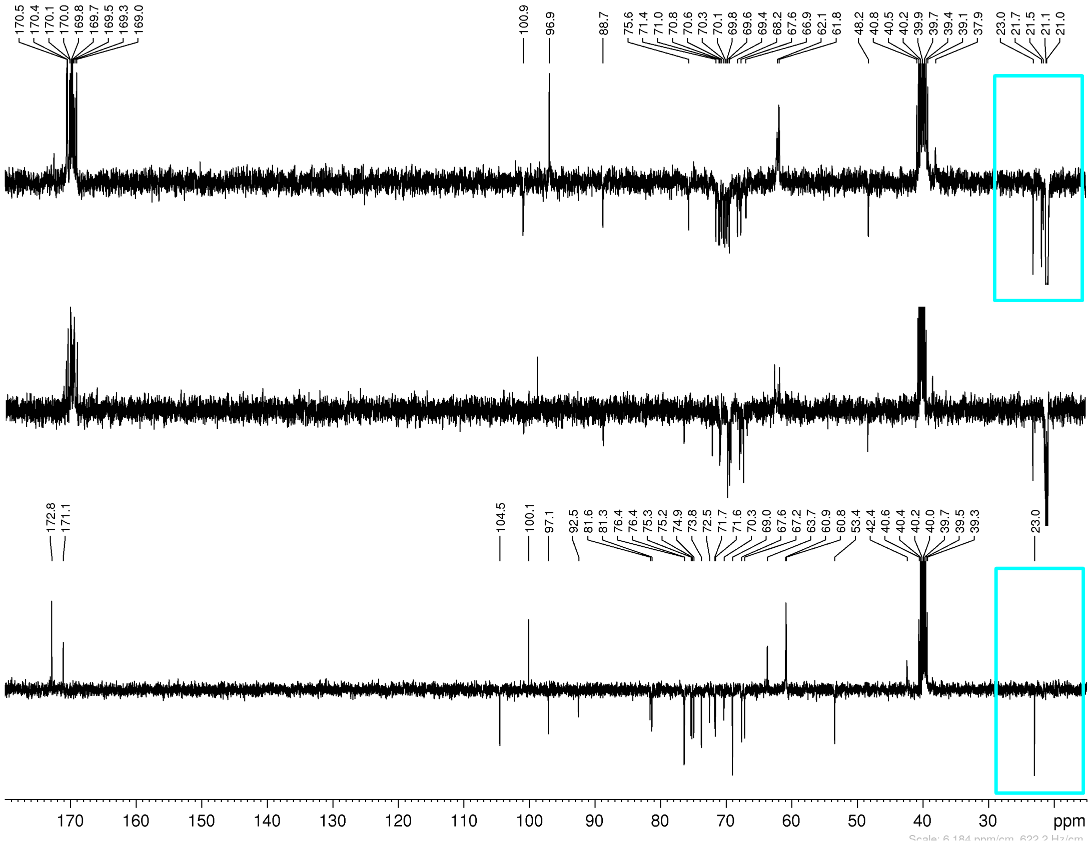

Figure S104. (From the top) ^{13}C NMR spectra (75 MHz, DMSO-*d*$_6$, 298 K) of 3'-sialyllactose (Ac), 6'-sialyllactose (Ac) and 3'-sialyllactose sodium salt. Highlighted areas show the CH_3 peaks associated with the acetyl protecting groups and the amide group.

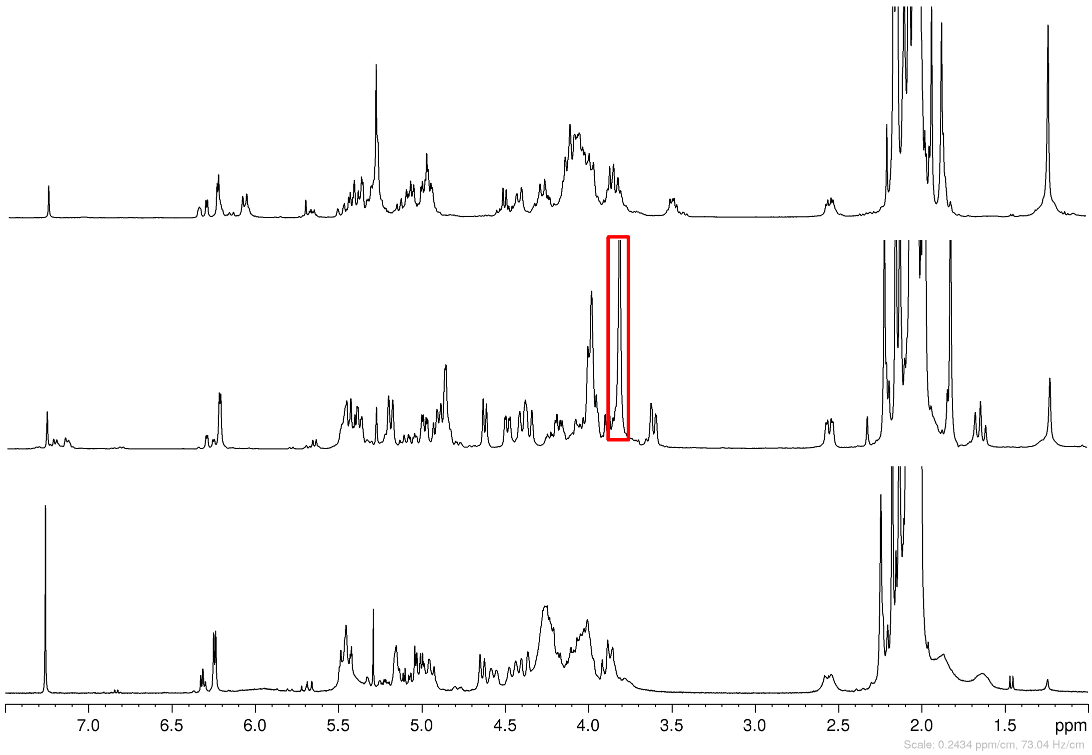

Figure S105. (From the top) ^{1}H NMR spectra (400 MHz, $CDCl_3$, 298 K) of 6'-sialyllactose (Ac), 3'-sialyllactose (Ac/Me) and 3'-sialyllactose (Ac). Highlighted area shows the methyl ester protons.

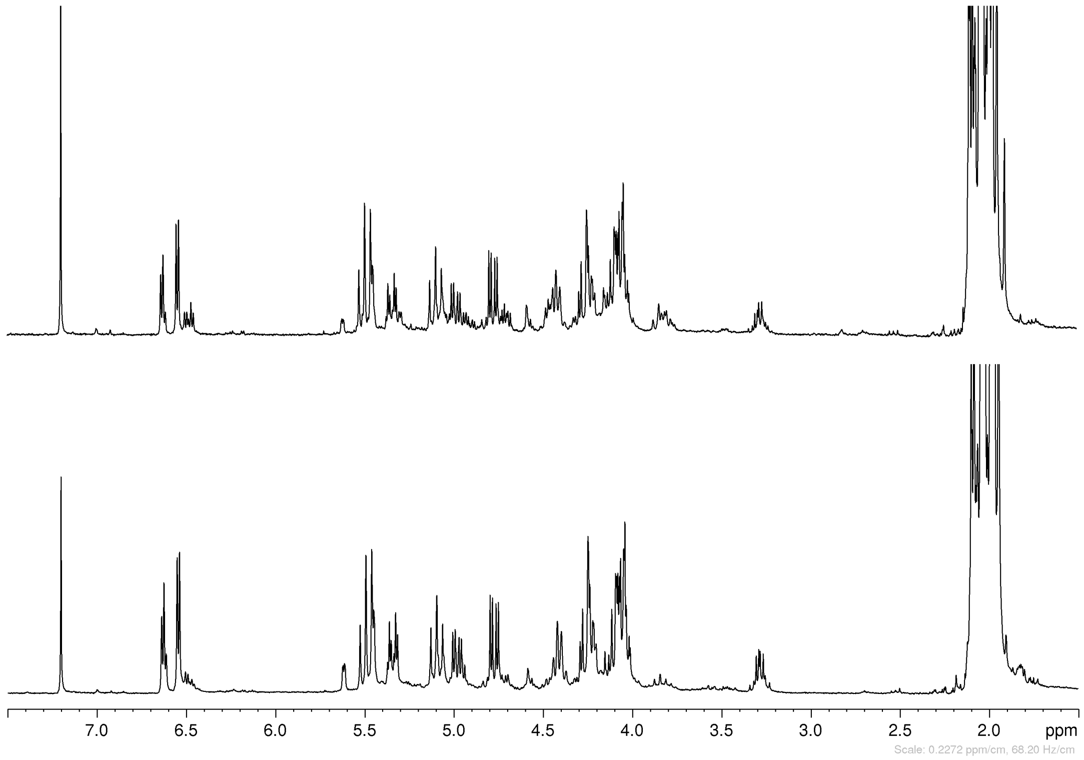

Figure S106. ^{1}H NMR spectra (300 MHz, $CDCl_3$, 298 K) of 1-bromo-6’-sialyllactose(Ac) and 1-bromo-3’-sialyllactose(Ac).

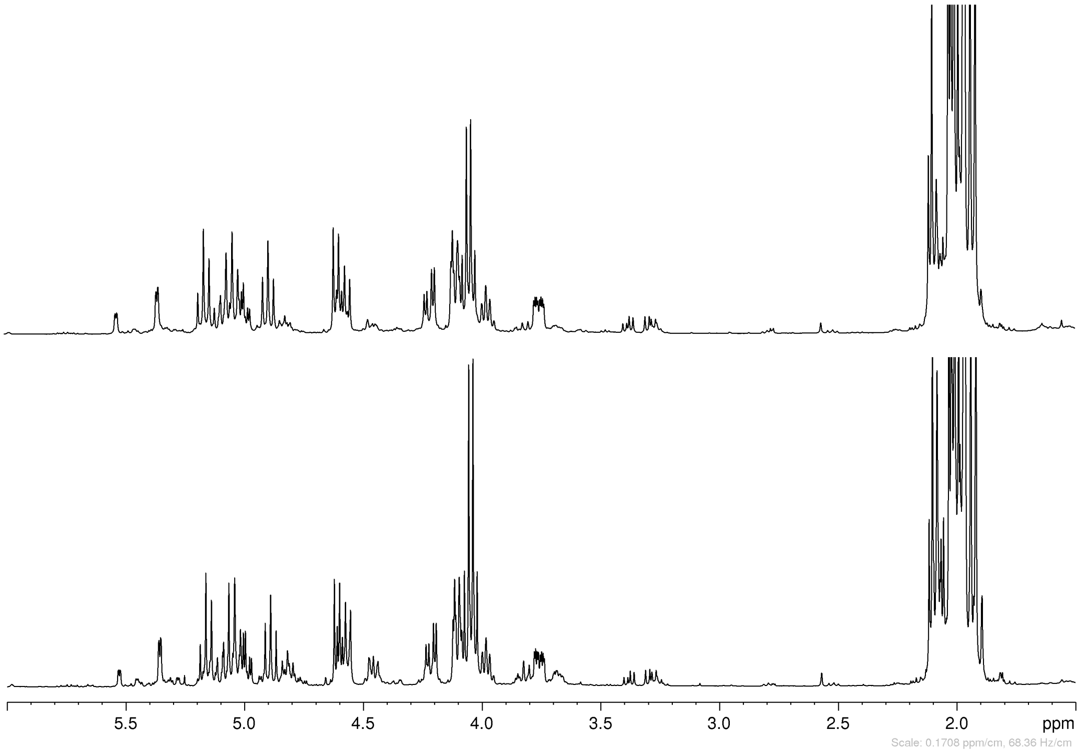

Figure S107. ^{1}H NMR spectra (300 MHz, $CDCl_3$, 298 K) of 1-azido-6’-sialyllactose(Ac) and 1-azido-3’-sialyllactose(Ac).

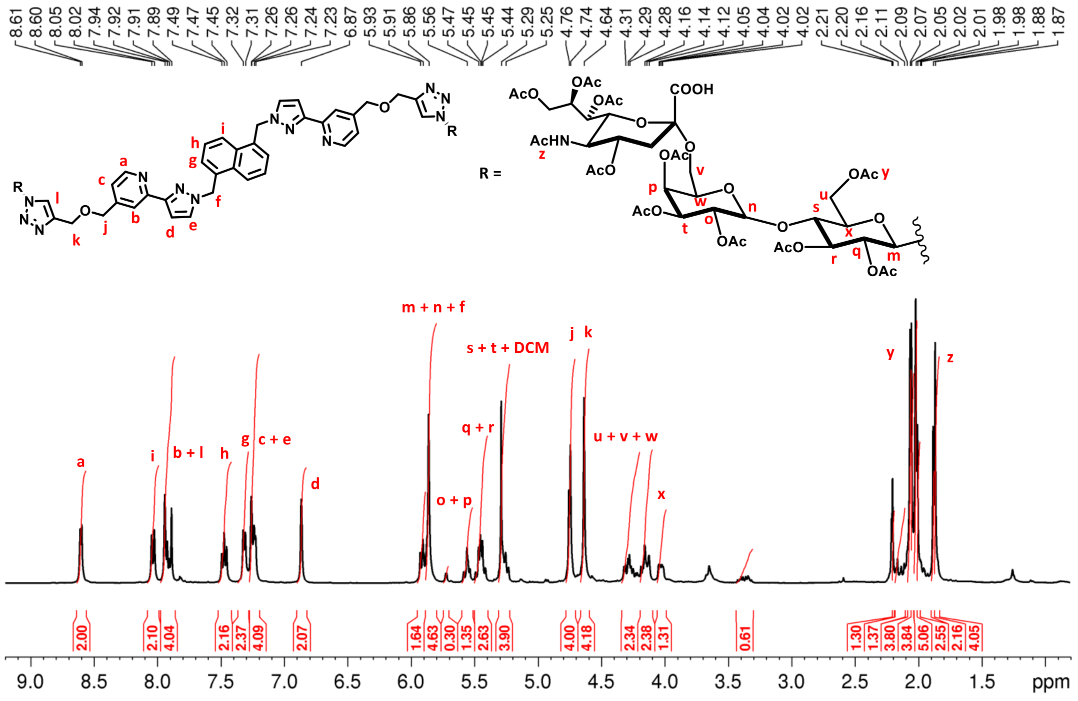

Figure S108. ^{1}H NMR spectrum (400 MHz, $CDCl_3$, 298 K) of **$L^{15\text{-}6SL\text{-}Ac}$** where HSQC was used to help assign the majority of the ligand signals. The triazole proton (l) integrates to 2H, which is indicative of a di-functionalised pure ligand; not mono-functionalised.

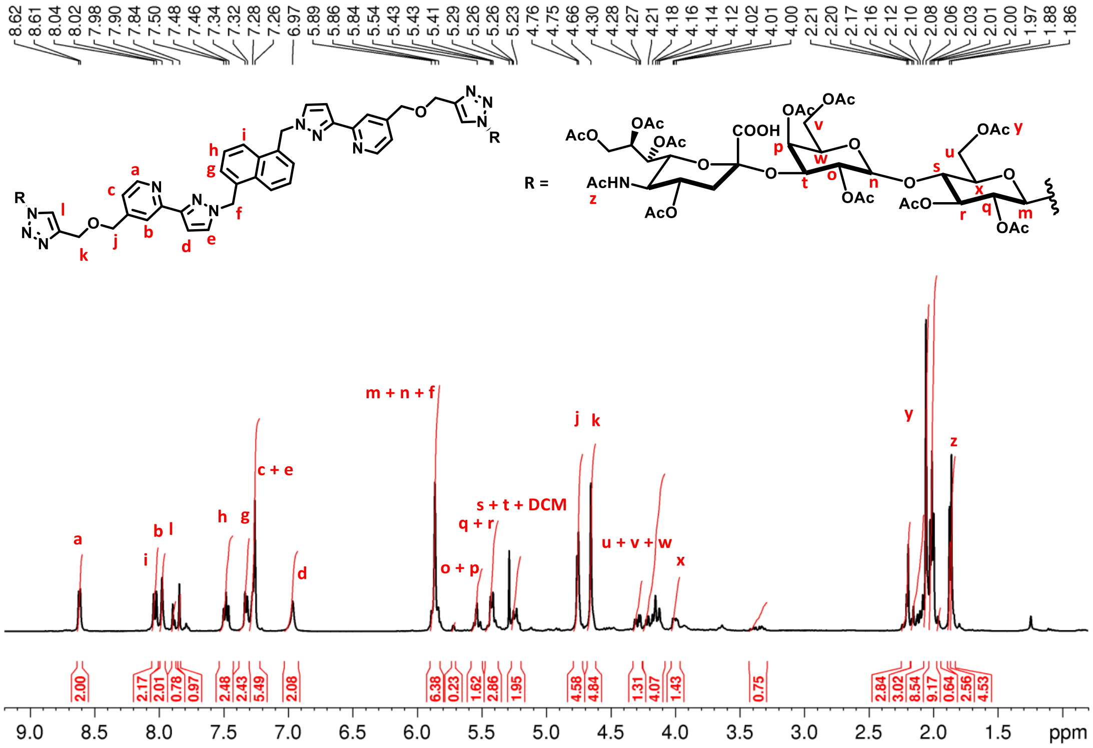

Figure S109. ^{1}H NMR spectrum (400 MHz, $CDCl_3$, 298 K) of $\mathbf{L^{15\text{-}3SL\text{-}Ac}}$ where HSQC was used to help assign the majority of the ligand signals. The triazole proton (l) integrates to 2H, which is indicative of a di-functionalised pure ligand; not mono-functionalised.

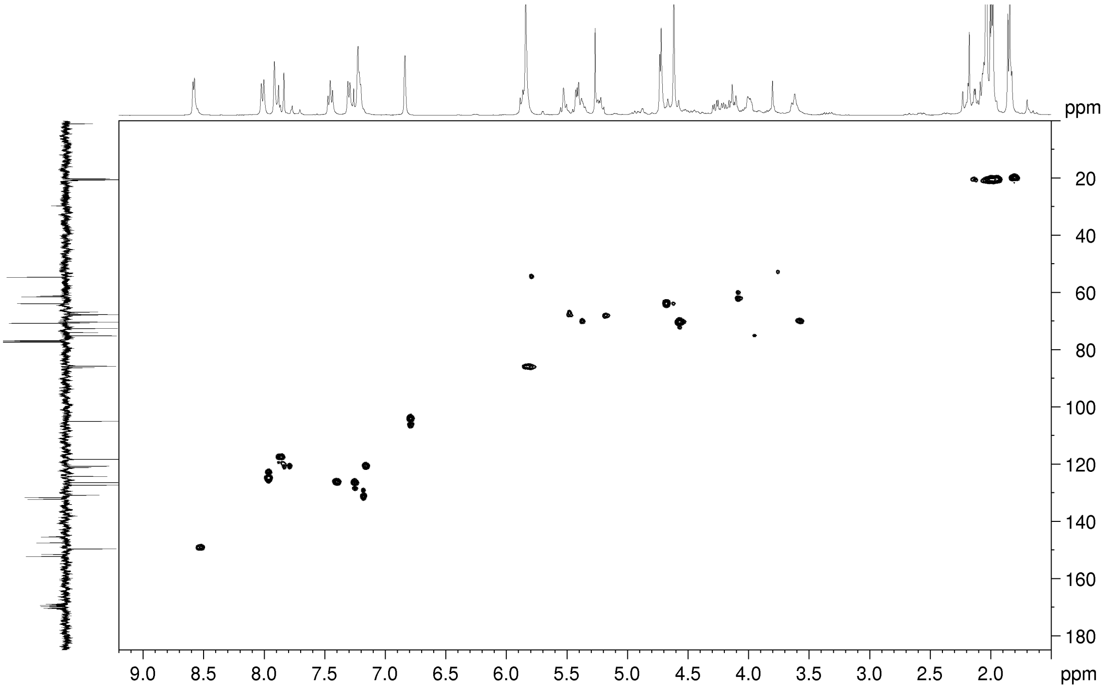

Figure S110. 2D-HSQC NMR spectrum ($CDCl_3$) of **L$^{15\text{-}3SL\text{-}Ac\text{-}Me}$**

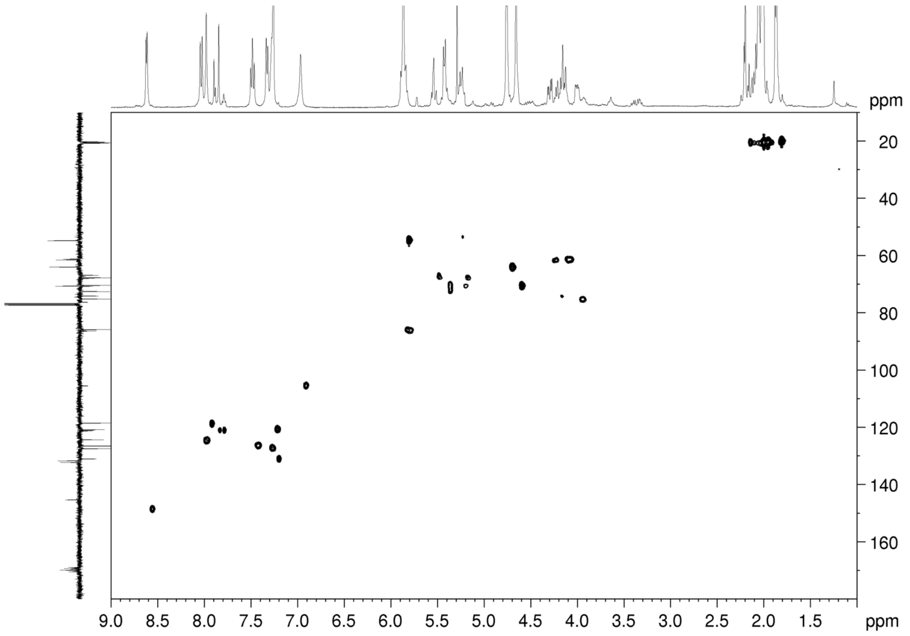

Figure S111. 2D-HSQC NMR spectrum ($CDCl_3$) of **L$^{15\text{-}3SL\text{-}Ac}$**

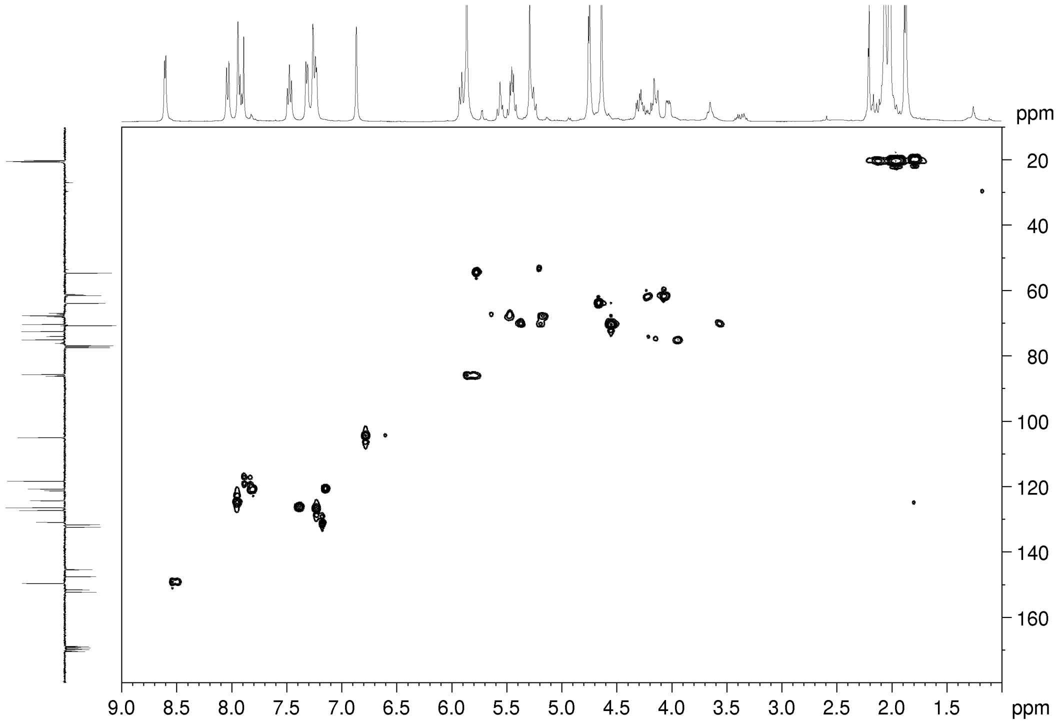

Figure S112. 2D-HSQC NMR spectrum ($CDCl_3$) of **L[15-6SL-Ac]**

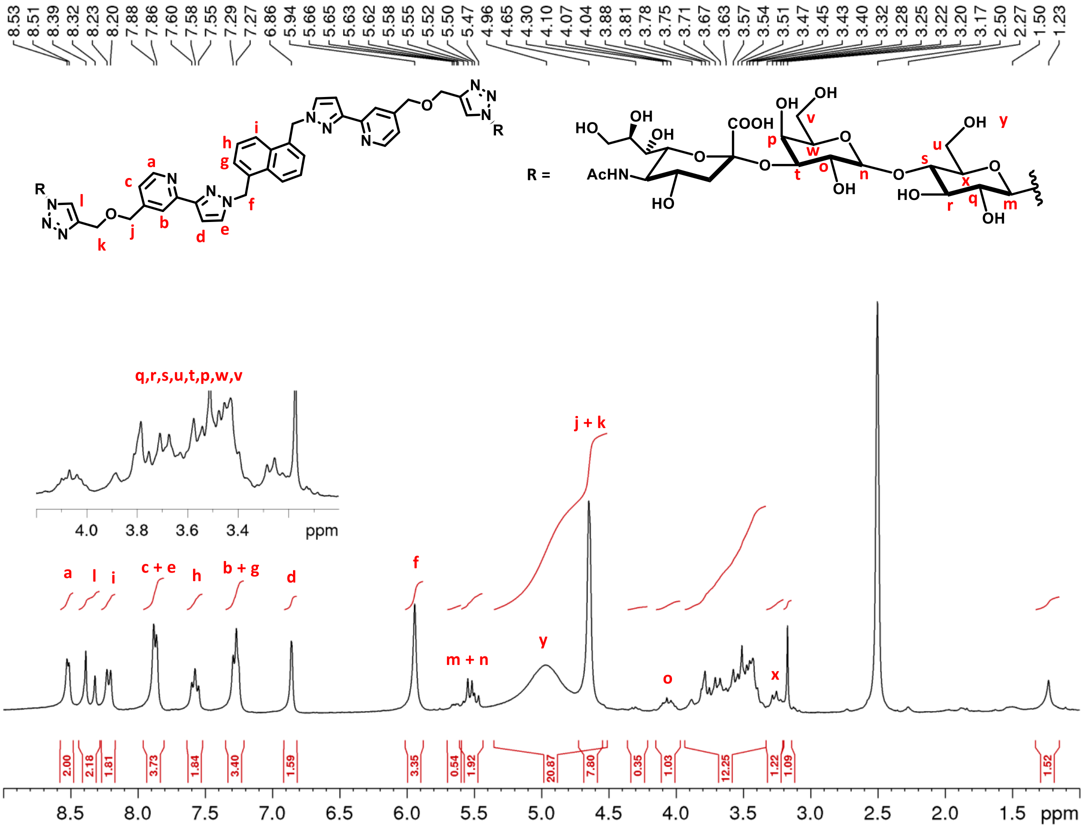

Figure S113. ^{1}H NMR spectrum (300 MHz, DMSO-d_6, 298 K) of $\mathbf{L^{15\text{-}3SL}}$ where HSQC was used to help assign the majority of the ligand signals. An expansion of the region from 4.2 ppm to 3.0 ppm is included. The triazole proton(s) (l) integrates to 2H, which is indicative of a di-functionalised pure ligand; not mono-functionalised.

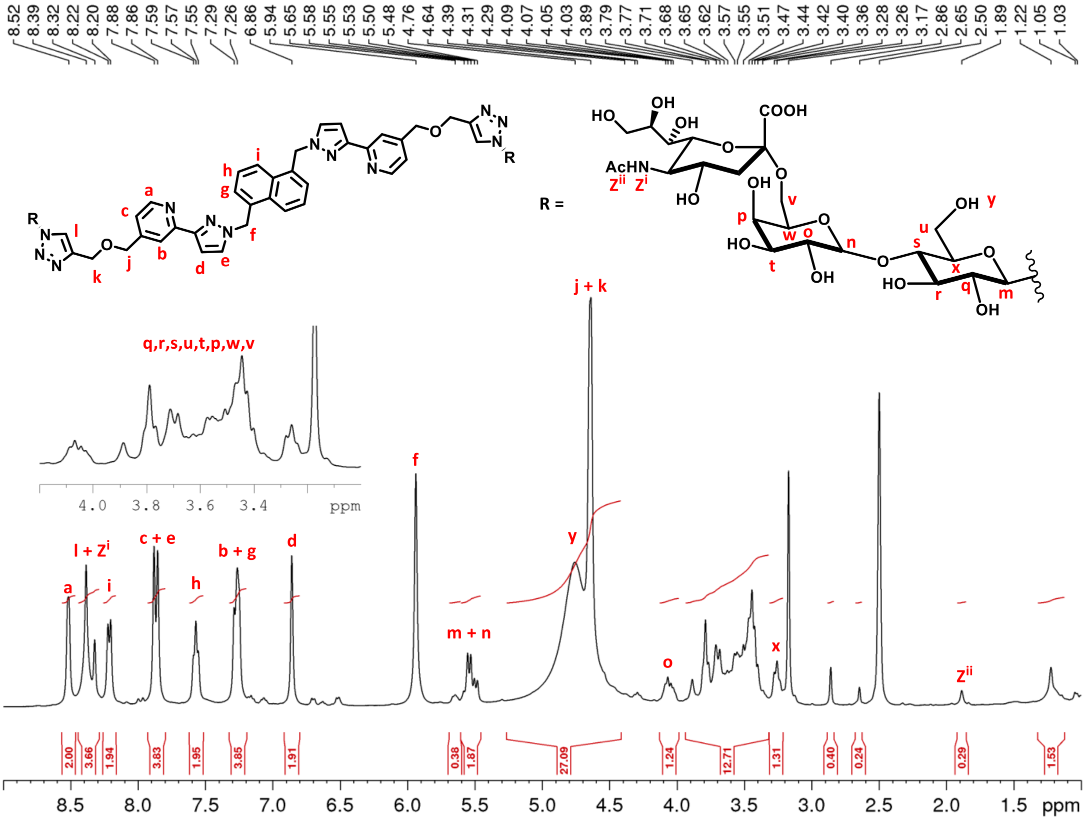

Figure S114. ^{1}H NMR spectrum (300 MHz, DMSO-d_6, 298 K) of $\mathbf{L}^{\mathbf{15\text{-}6SL}}$ where HSQC was used to help assign the majority of the ligand signals. An expansion of the region from 4.2 ppm to 3.0 ppm is included. The triazole proton (l) integrates to 2H, which is indicative of a di-functionalised pure ligand; not mono-functionalised. The amide N**H** proton (Z^{i}) can also be seen in this NMR spectrum.

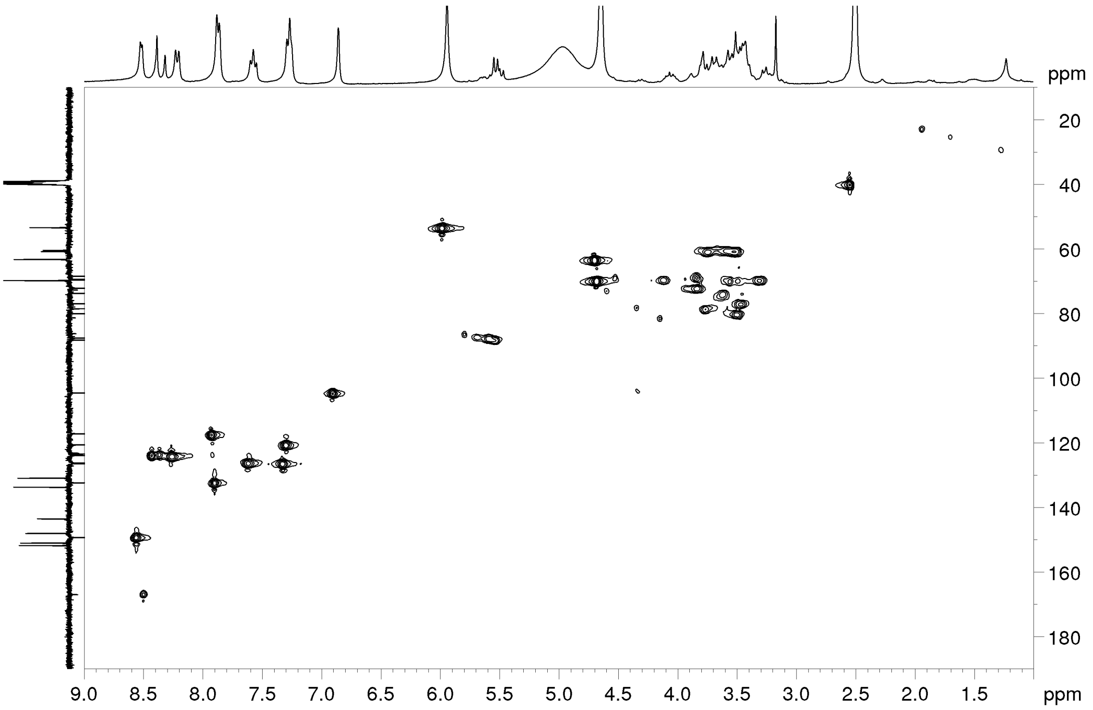

Figure S115. 2D-HSQC NMR spectrum (DMSO-d_6) of $\mathbf{L}^{\mathbf{15\text{-}3SL}}$

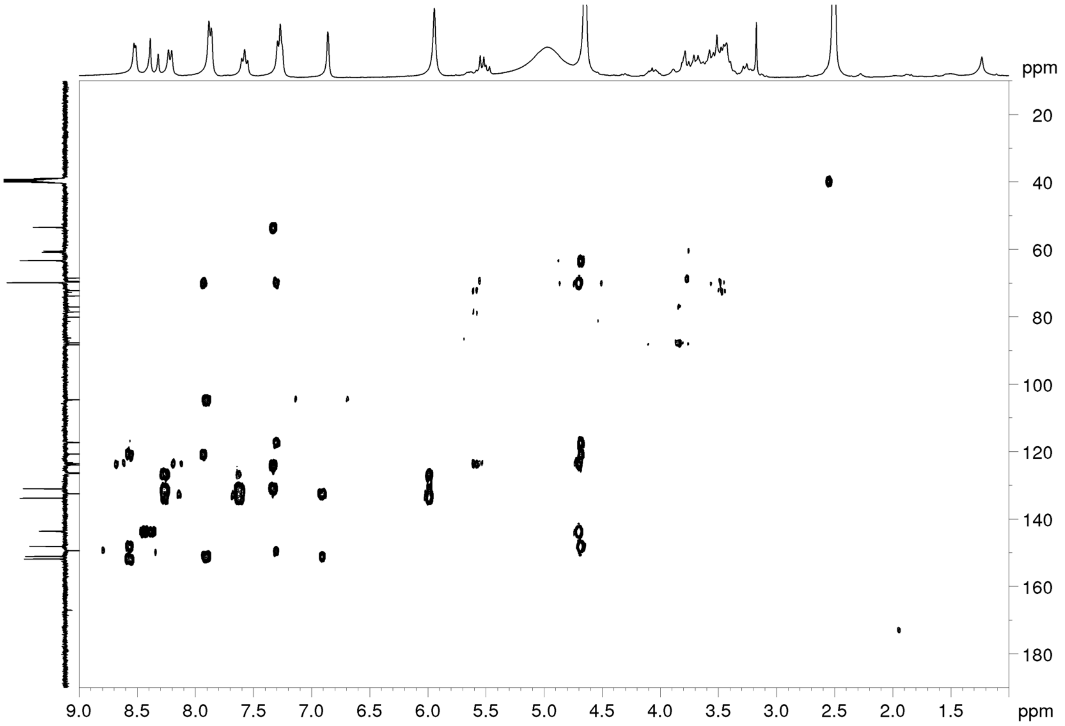

Figure S116. 2D-HMBC NMR spectrum (DMSO-d_6) of $\mathbf{L}^{\mathbf{15\text{-}3SL}}$

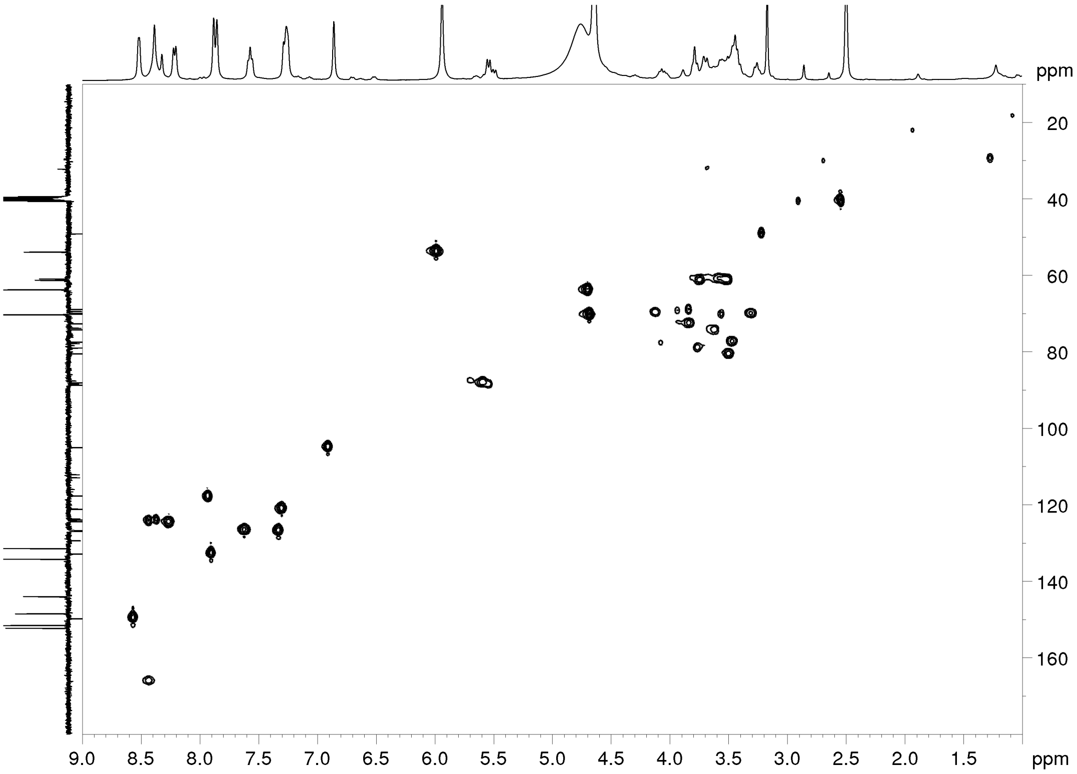

Figure S117. 2D-HSQC NMR spectrum (DMSO-*d*$_6$) of $\mathbf{L^{15\text{-}6SL}}$

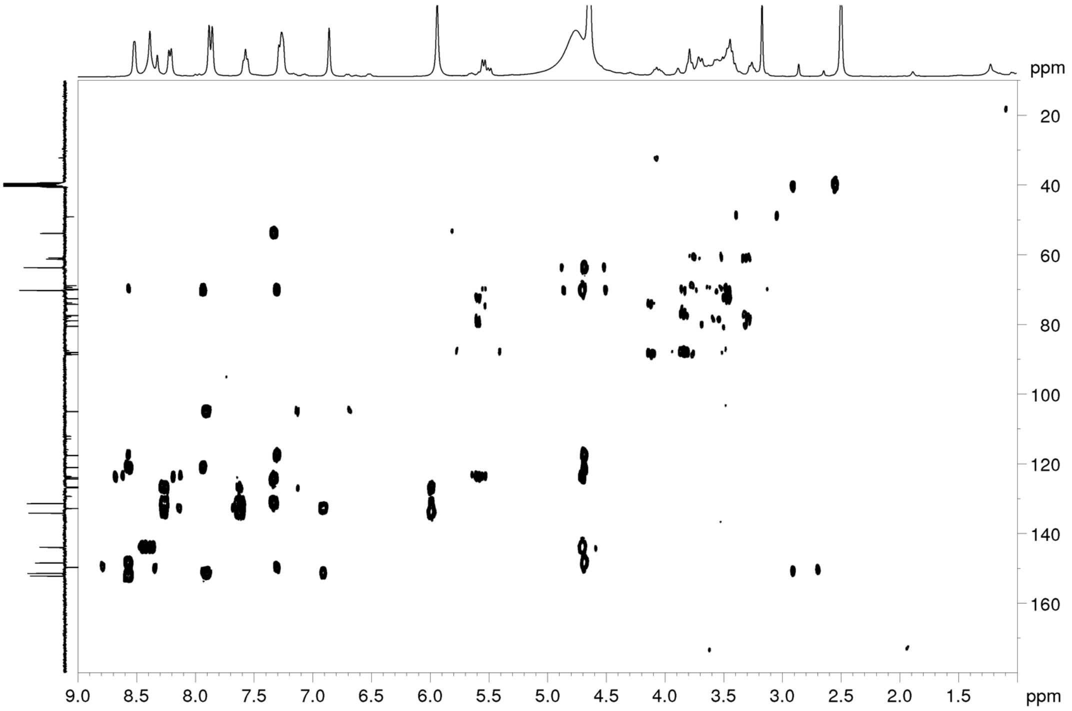

Figure S118. 2D-HMBC NMR spectrum (DMSO-*d*$_6$) of $\mathbf{L^{15\text{-}6SL}}$

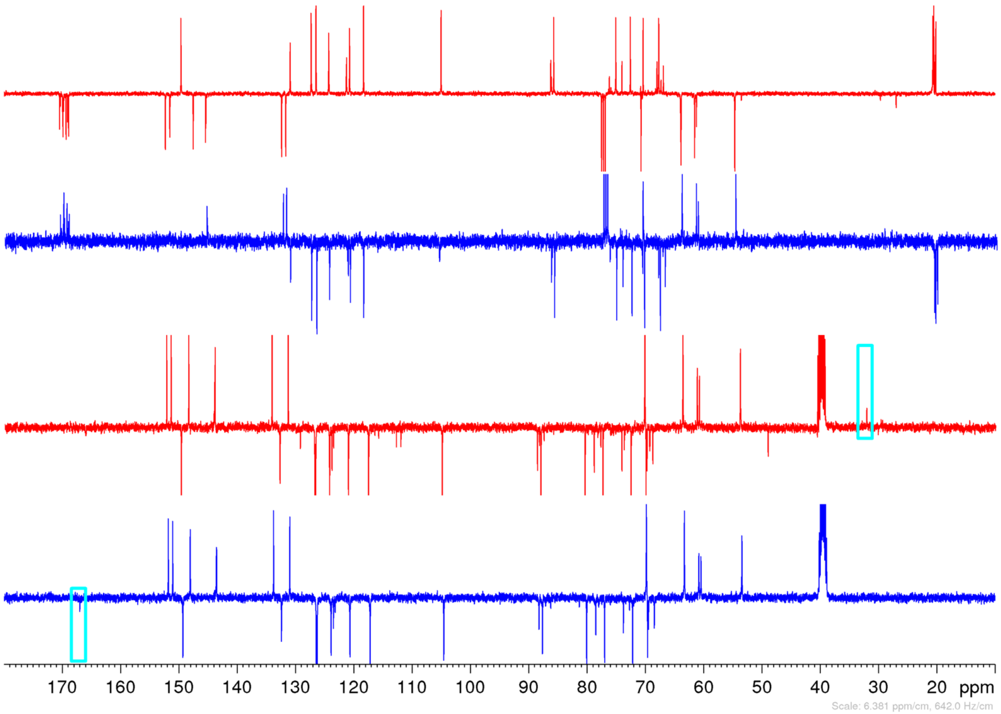

Figure S119. ^{13}C NMR spectra (DMSO-d_6 and $CDCl_3$) of $\mathbf{L^{15\text{-}3SL}}$ and $\mathbf{L^{15\text{-}3SL\text{-}Ac}}$ (blue) and $\mathbf{L^{15\text{-}6SL}}$ and $\mathbf{L^{15\text{-}6SL\text{-}Ac}}$ (red). Highlighted areas (cyan boxes) show the signals associated with the amide group that are still retained after deprotection with *Zemplen* conditions.

**It is noted that the concentration of NMR samples ranges from 30 mM to 50 mM (in most cases) so the absence of some peaks are seen in the weaker samples, yet the majority of the ligand core is observed.*

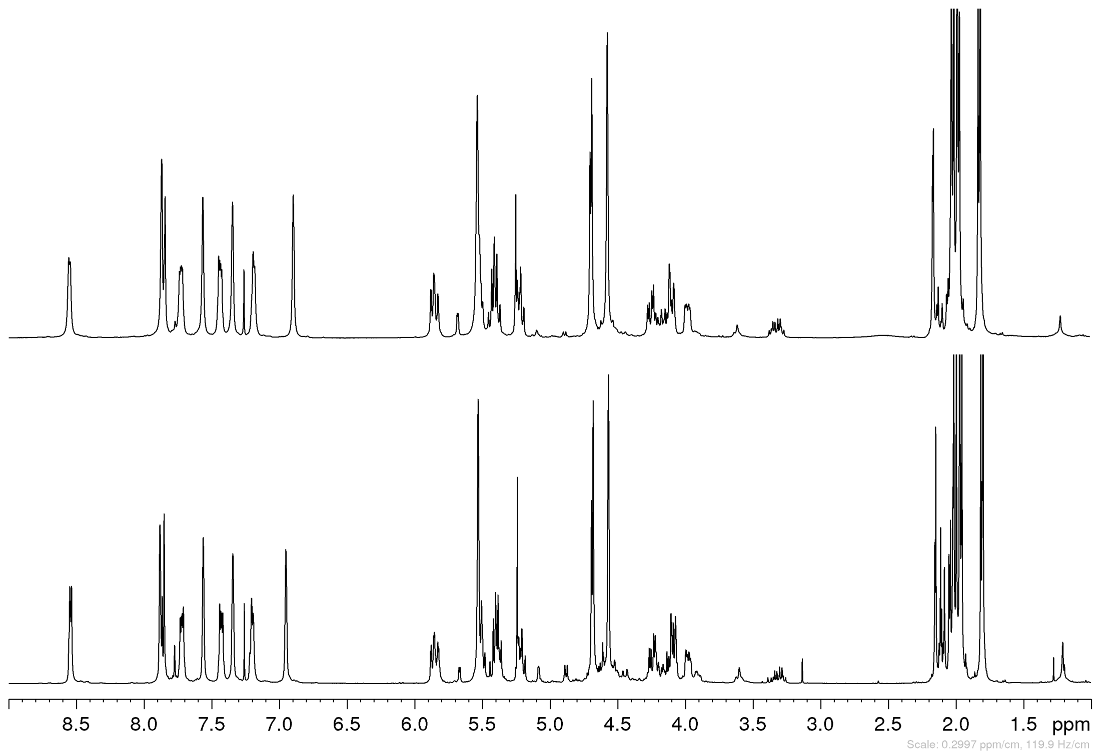

Figure S120. ^{1}H NMR spectra (400 MHz, $CDCl_3$, 298 K) of $\mathbf{L^{23\text{-}3SL\text{-}Ac}}$ vs $\mathbf{L^{23\text{-}6SL\text{-}Ac}}$

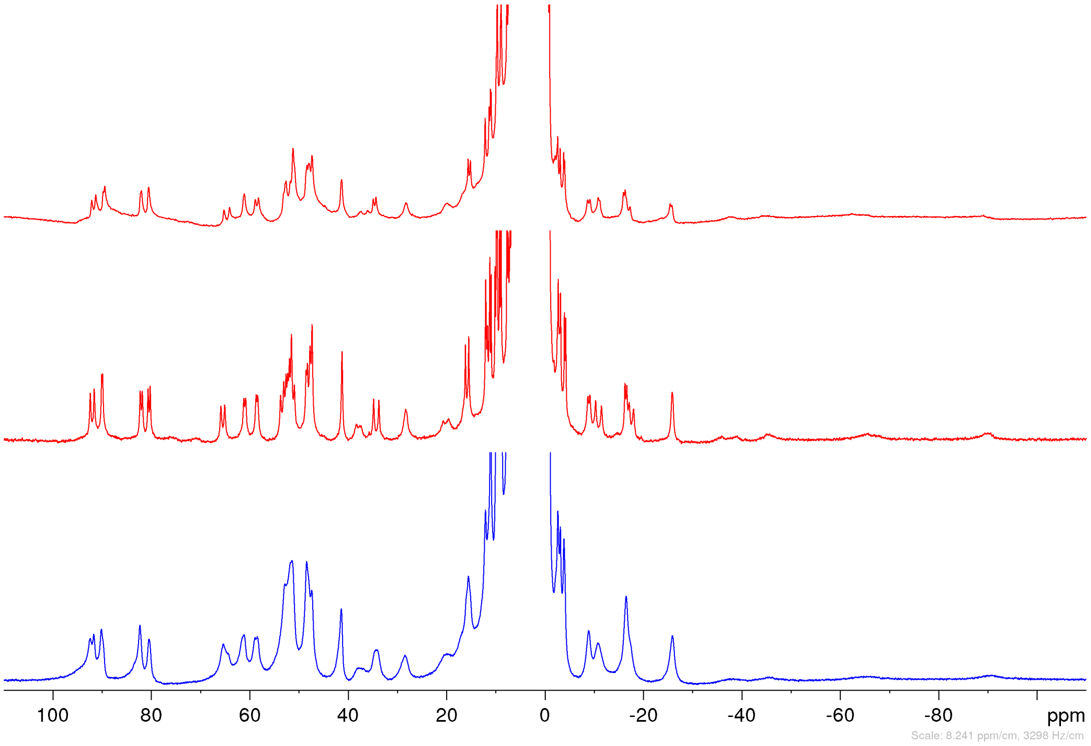

Figure S121. ^{1}H NMR spectra (400 MHz, CD_3CN, 298 K) of **$[Co_8(L^{15\text{-}3SL\text{-}Ac})_{12}(BF_4)_{16}]$** (blue) compared to the cages with monosaccharide glucose (top) and galactose (middle) pendants (red). There is enhanced broadness of the cage peaks with sialyllactose pendant groups compared to monosaccharide pendant groups due to the increase in size and therefore slower tumbling in solution.

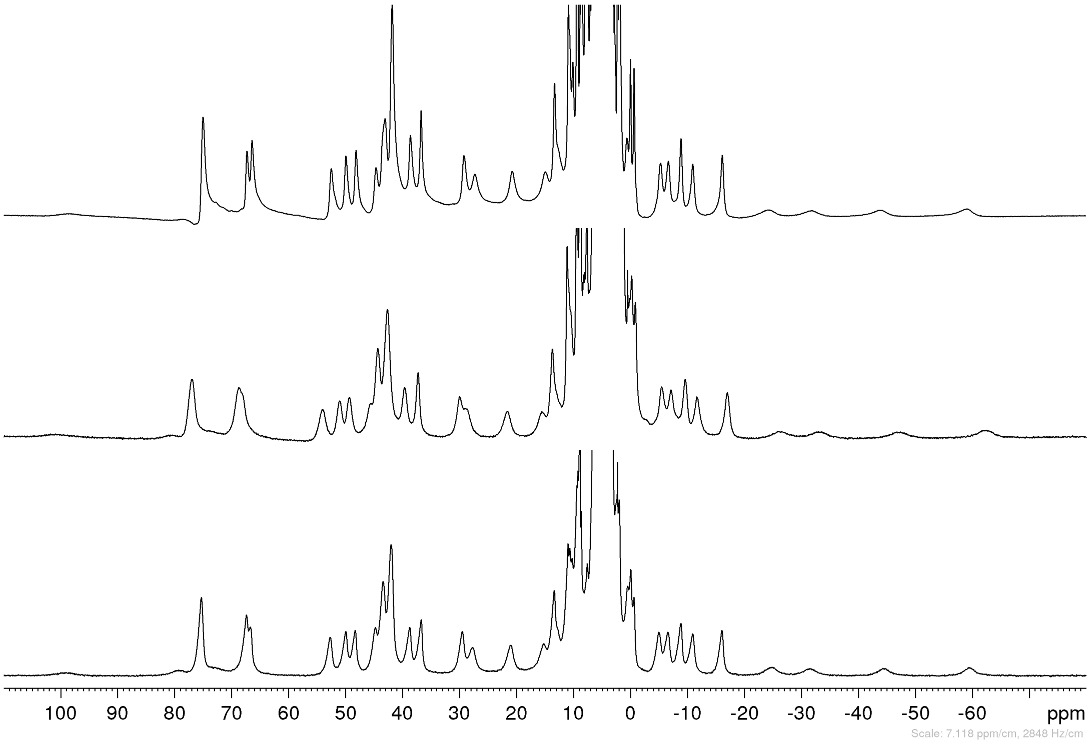

Figure S122. (Top to bottom) ^{1}H NMR spectra (400 MHz, D_2O, 363 K) of **[Co$_8$(L$^{15\text{-}6SL}$)$_{12}$(BF$_4$)$_{16}$]**, **[Co$_8$(L$^{15\text{-}3SL\text{-}Me}$)$_{12}$(BF$_4$)$_{16}$]** and **[Co$_8$(L$^{15\text{-}3SL}$)$_{12}$(BF$_4$)$_{16}$]**.

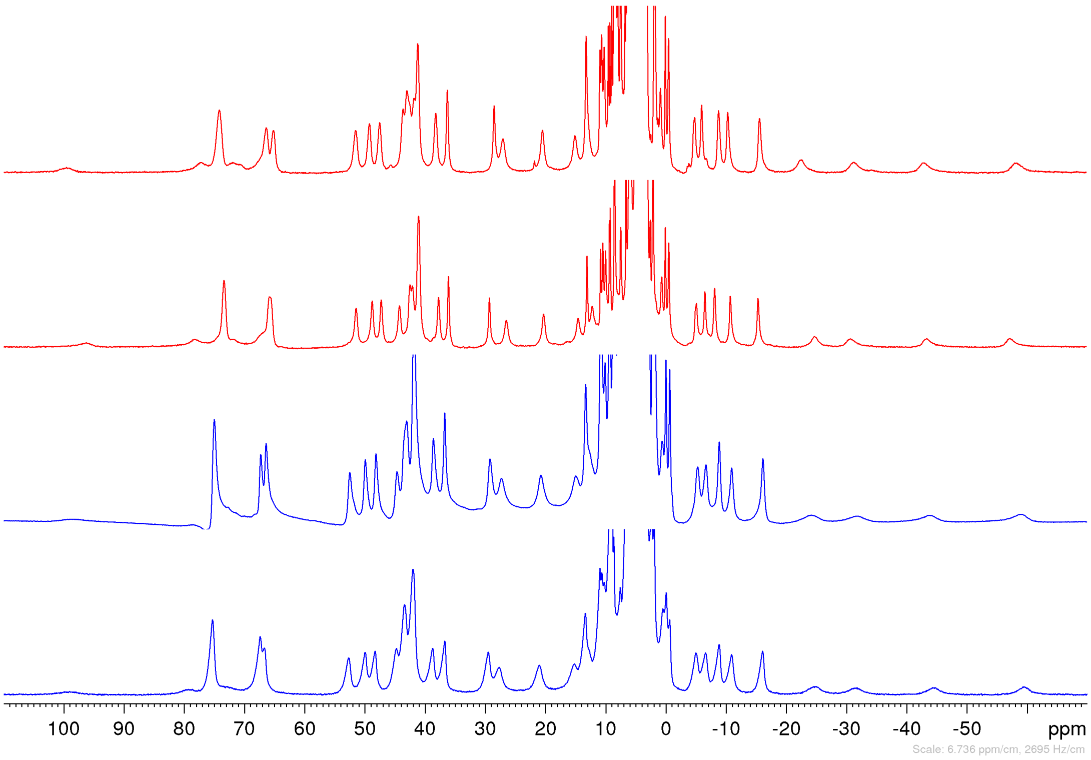

Figure S123. ^{1}H NMR spectra (400 MHz, D_2O, 363 K) of **[$Co_8(L^{15\text{-}6SL})_{12}(BF_4)_{16}$]** and **[$Co_8(L^{15\text{-}3SL})_{12}(BF_4)_{16}$]** (blue – top and bottom respectively) and the monosaccharide-pendant cubic cages with galactose and glucose groups (red – top and bottom respectively). There is enhanced broadness of the sialyllactose-pendant cage peaks due to the increase in size and therefore slower tumbling in solution (even at 363K).

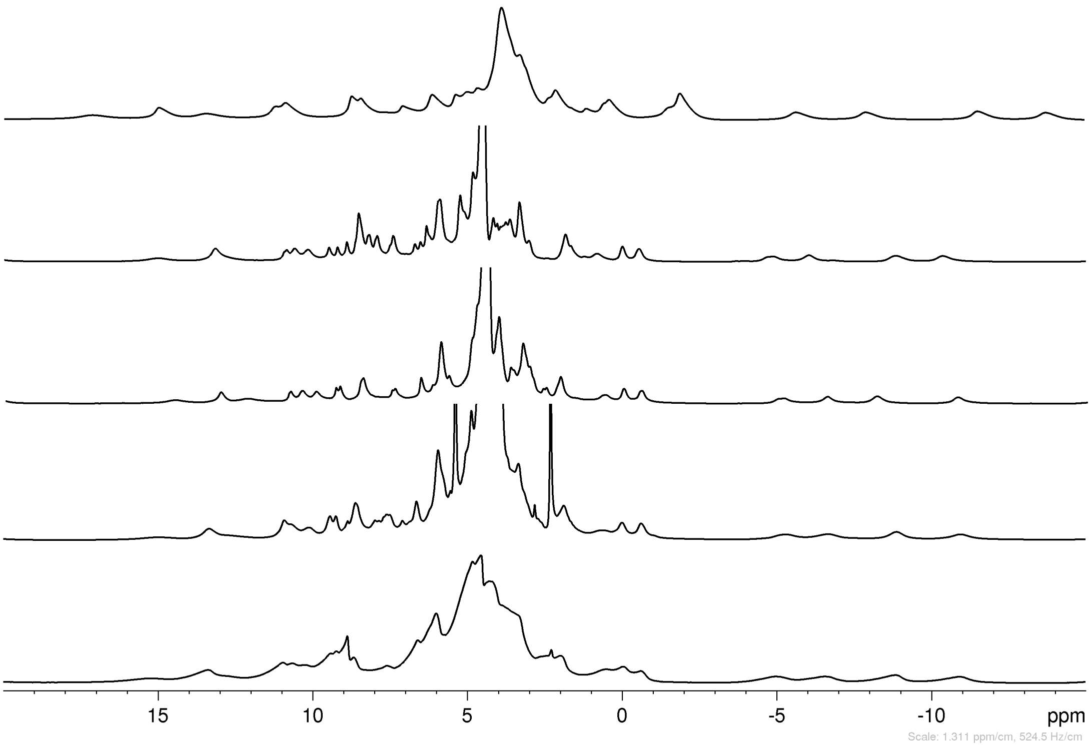

Figure S124. ^{1}H NMR spectra of (from top to bottom): **[$Co_8(L^{15CC})_{12}(BF_4)_{16}$]**, **[$Co_8(L^{15\text{-}Gal})_{12}(BF_4)_{16}$]**, **[$Co_8(L^{15\text{-}Glu})_{12}(BF_4)_{16}$]**, **[$Co_8(L^{15\text{-}6SL})_{12}(BF_4)_{16}$]** and **[$Co_8(L^{15\text{-}3SL})_{12}(BF_4)_{16}$]** between 20 and -15 ppm, which highlights the difference in the terminal functional groups of each ligand, furthest away from the Co(II) centres. For the **SL**-pendant cages, there is enhanced broadness around the 12 to -2 ppm region, associated with slower tumbling of the larger complex in solution. The -5 to -14 ppm region is very characteristic of the cubic cage symmetry, with 2 ligand environments having an internal symmetry which leads to four copies of each chemical type of signal.

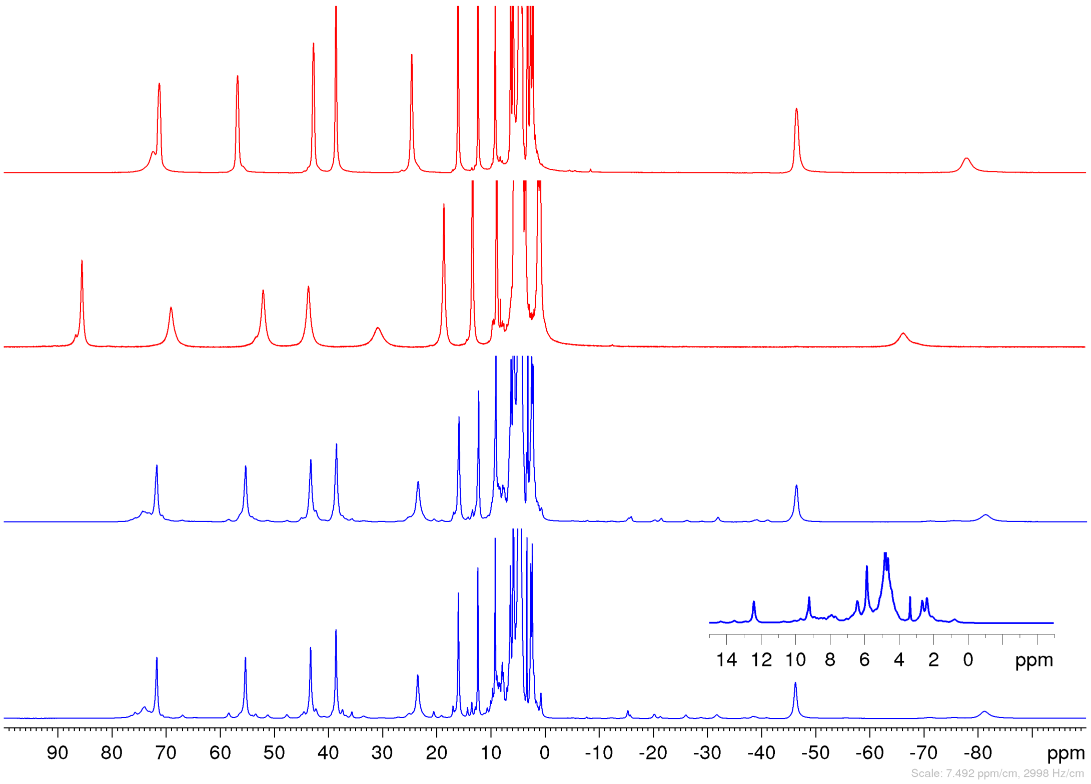

Figure S125. ^{1}H NMR spectra (400 MHz, D_2O, 363K) of **[Co$_4$(L$^{23\text{-}6SL}$)$_6$(BF$_4$)$_8$]** and **[Co$_4$(L$^{23\text{-}3SL}$)$_6$(BF$_4$)$_8$]** (blue – top to bottom) compared to the monosaccharide cubic cages of glucose and galactose (red – top to bottom). The inset shows an expansion of the spectrum of **[Co$_4$(L$^{23\text{-}3SL}$)$_6$(BF$_4$)$_8$]** in the region 15 ppm to -5 ppm.

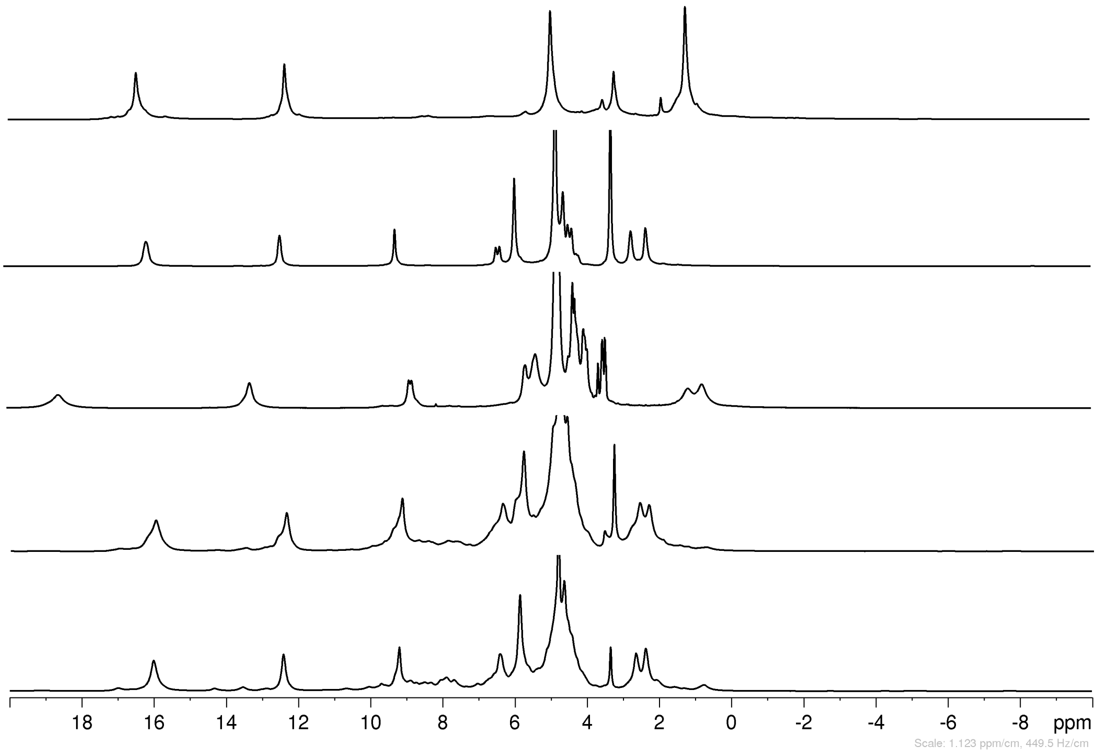

Figure S126. ^{1}H NMR spectra of (from top to bottom): **[Co$_4$(L^{23CC})$_6$(BF$_4$)$_8$]**, **[Co$_4$(L$^{23\text{-}Glu}$)$_6$(BF$_4$)$_8$]**, **[Co$_4$(L$^{23\text{-}Gal}$)$_6$(BF$_4$)$_8$]** and **[Co$_4$(L$^{23\text{-}6SL}$)$_6$(BF$_4$)$_8$]** and **[Co$_4$(L$^{23\text{-}3SL}$)$_6$(BF$_4$)$_8$]** between 20 and -10 ppm, which highlights the difference in the terminal functional groups of each ligand, furthest away from the cobalt centres. For the **SL** cages, there is enhanced broadness around the 7 to 4 ppm region, associated with slower tumbling of the larger complex in solution.

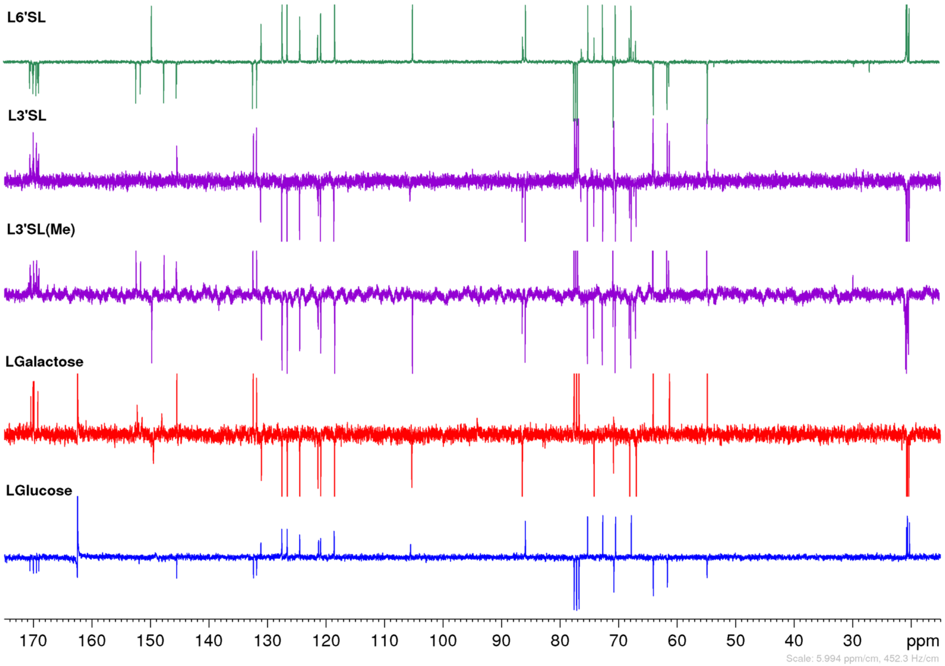

Figure S127. Comparison of the ^{13}C NMR spectra for all types of 1,5 acetyl protected ligands (100 MHz for the **SL** ligands and 75 MHz for glucose and galactose, $CDCl_3$, 298 K).

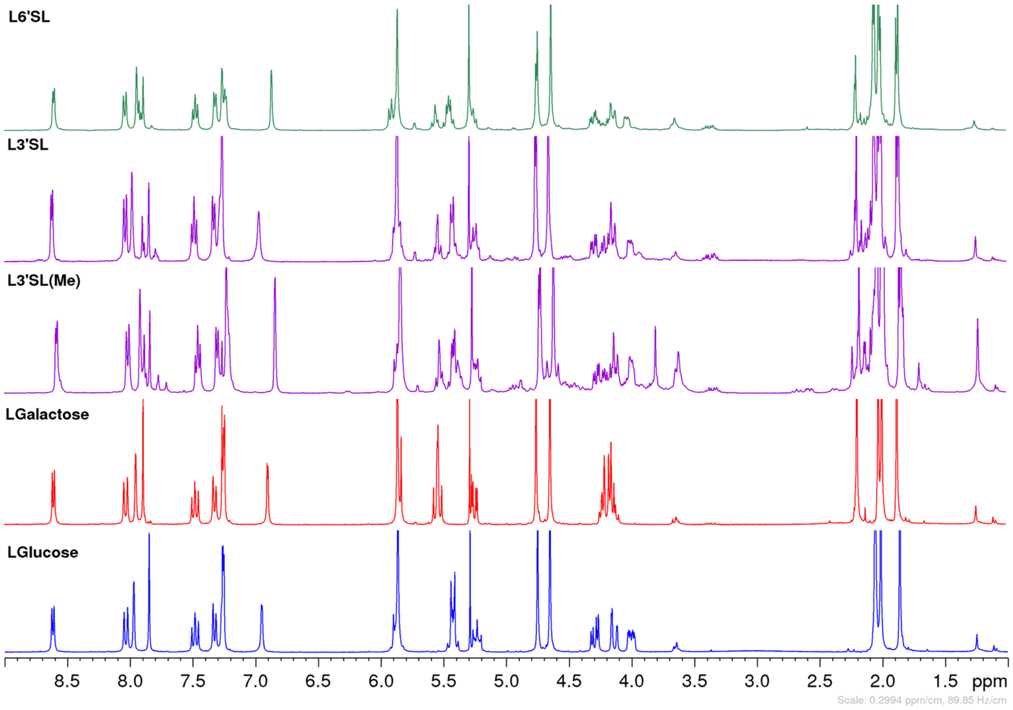

Figure S128. Comparison of the ^{1}H NMR spectra for all types of 1,5 acetyl protected ligand (400 MHz for the **SL** ligands and 300 MHz for glucose and galactose, $CDCl_3$, 298 K).

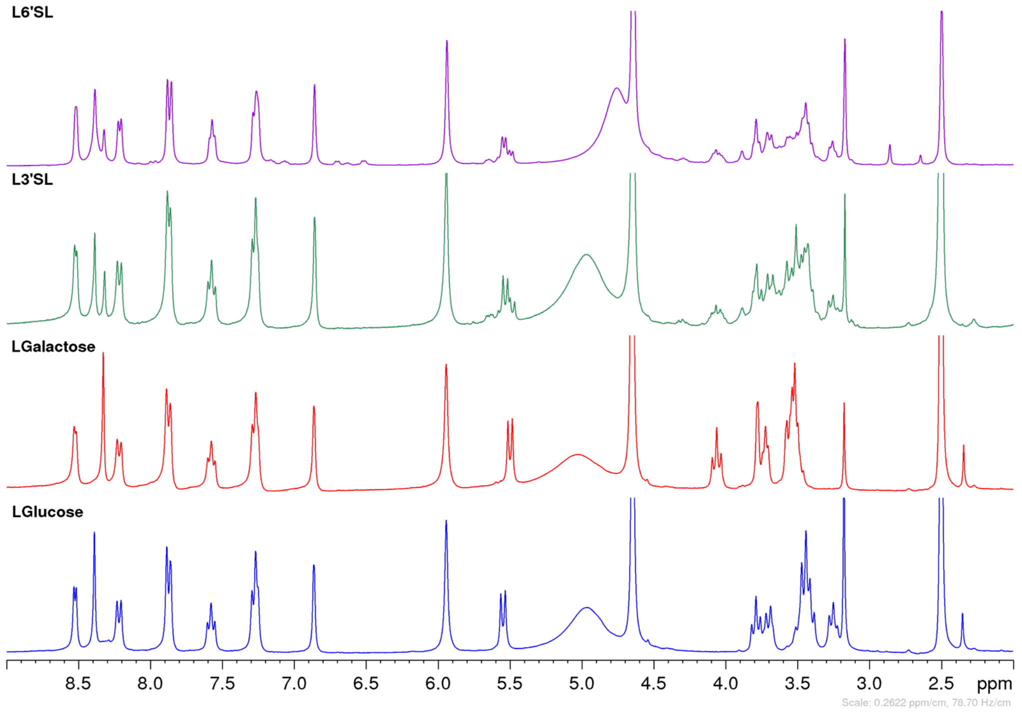

Figure S129. Comparison of the ^{1}H NMR spectra for 1,5 deprotected ligands (400 MHz for the **SL** ligands and 300 MHz for glucose and galactose, DMSO-d_6, 298 K).

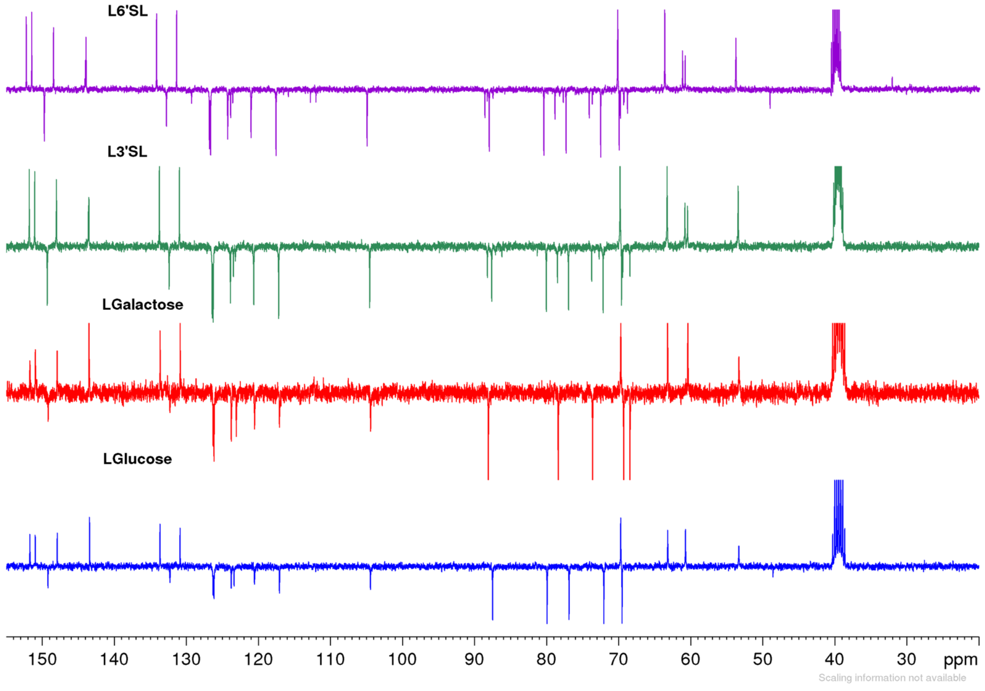

Figure S130. Comparison of ^{13}C NMR spectra for 1,5 deprotected ligands (100 MHz for the **SL** ligands and 75 MHz for glucose and galactose, DMSO-d_6, 298 K).

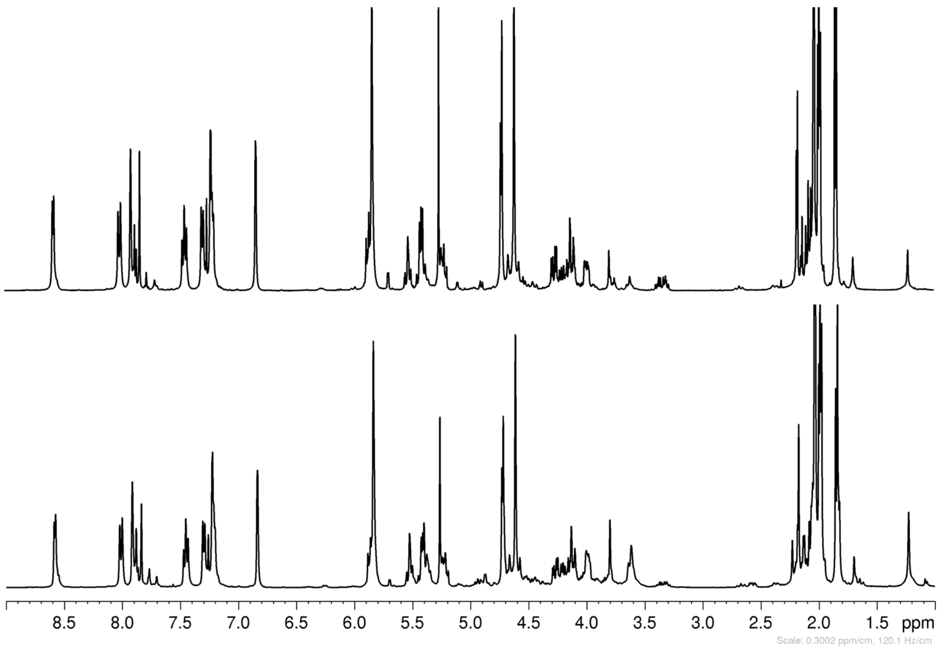

Figure S131. Comparison of the ^{1}H NMR spectra for $\mathbf{L^{15\text{-}3SL\text{-}Ac\text{-}Me}}$ and $\mathbf{L^{15\text{-}6SL\text{-}Ac\text{-}Me}}$ (400 MHz, $CDCl_3$, 298 K).

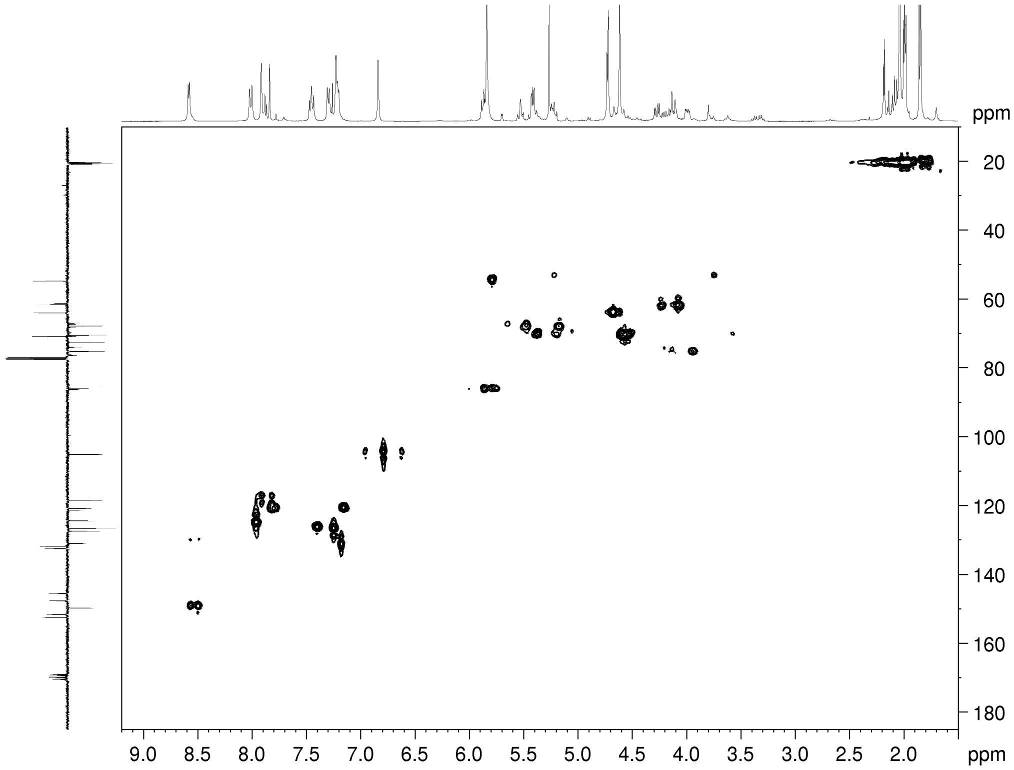

Figure S132. 2D-HSQC NMR spectrum ($CDCl_3$) of $\mathbf{L^{15\text{-}6SL\text{-}Ac\text{-}Me}}$.

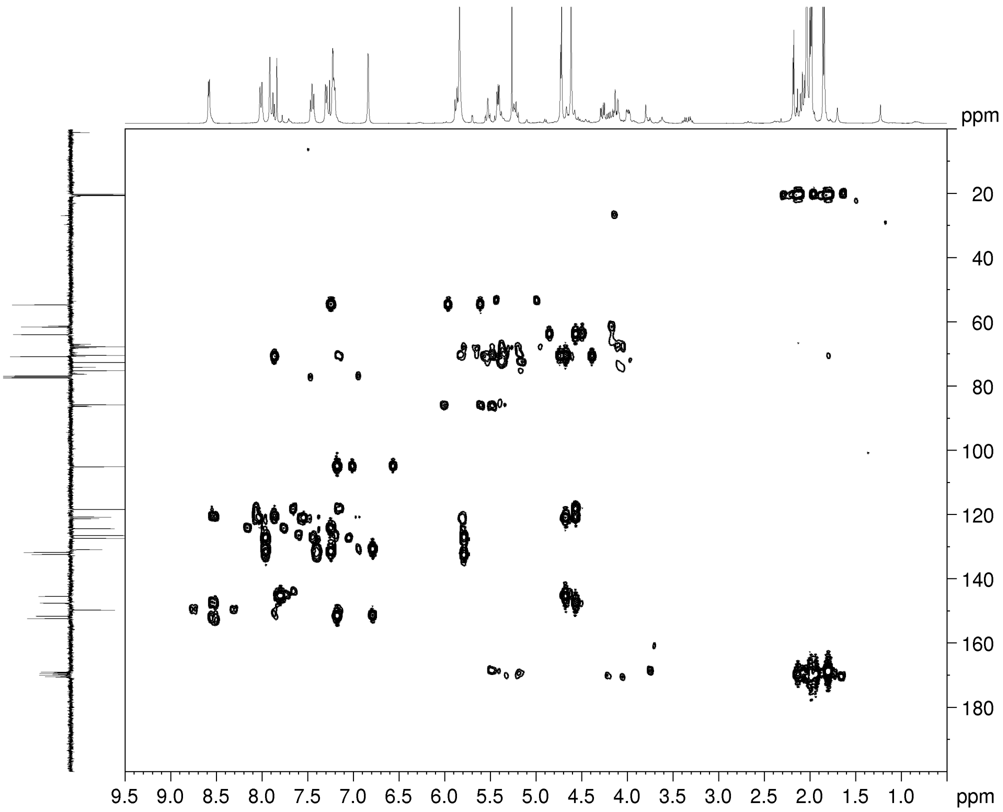

Figure S133. 2D-HMBC NMR spectrum ($CDCl_3$) of **L$^{15\text{-}6SL\text{-}Ac\text{-}Me}$**.

Stability study of the Zn_4 tetrahedral and Zn_8 cubic cages

Water stability of **[$Zn_4(L^{23\text{-}Gal})_6(BF_4)_8$]** and **[$Zn_8(L^{15\text{-}Gal})_{12}(BF_4)_{16}$]** as analysed by ^1H NMR spectroscopy. The samples were kept at RT inside standard NMR tubes for ~2 months.

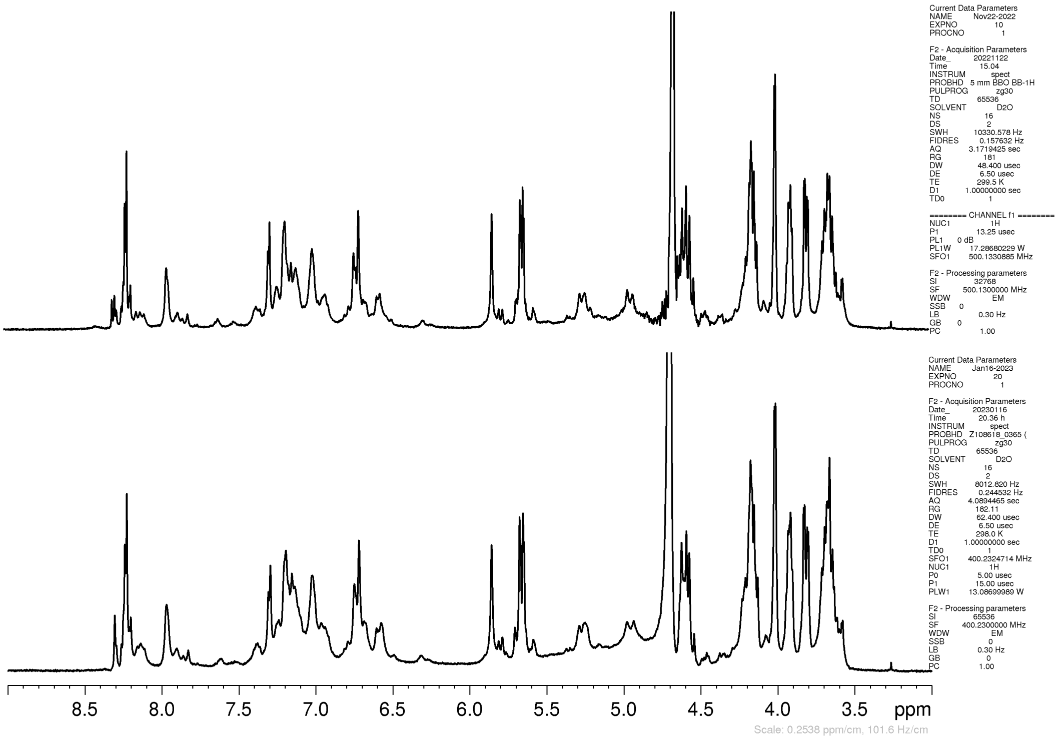

Figure S134. ^1H NMR spectra (D_2O) of **[$Zn_4(L^{23\text{-}Gal})_6(BF_4)_8$]** at 298K. The NMR spectra indicate no change in the cage structure over a ~2 month period whilst remaining in solution.

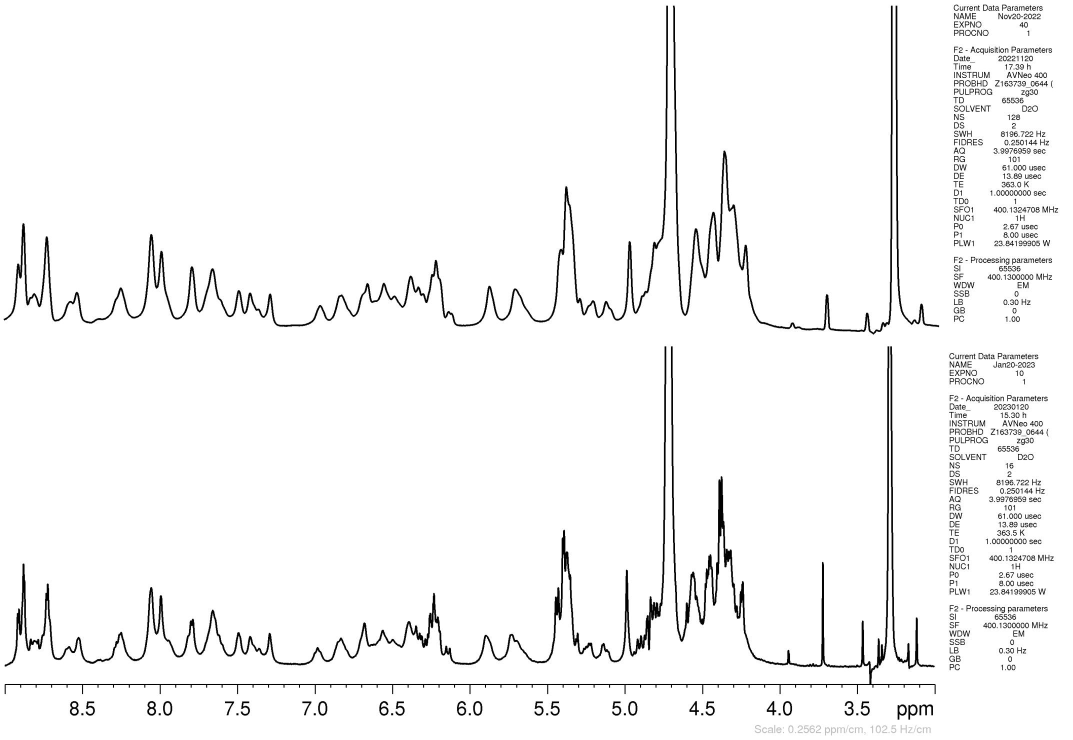

Figure S135. ^{1}H NMR spectra (D_2O) of **$[Zn_8(L^{15\text{-}Gal})_{12}(BF_4)_{16}]$** at 363K. The NMR spectra indicate no change in the cage structure over a ~2 month period whilst remaining in solution.

Aggregation assays

Turbidimetry experiments

Soybean Agglutinin (SBA) was dissolved in HEPES buffer (10 mM HEPES, 0.15 M NaCl, 2 mM $CaCl_2$, 0.2 mM $MnCl_2$, pH 7.4)

Jacalin and Wheat Germ Agglutinin were dissolved in HEPES buffer (10 mM HEPES, 0.1 mM $CaCl_2$, pH 8.5)

In a 96-well plate half-area flat bottom, lectin (50 µL, 10 µM) and an aqueous solution of metal complex (5 µL, 500 µM) were quickly mixed and the absorbance was recorded at 420, 500 and 600 nm for 30 minutes every 60 seconds. A solution of free sugar (D-galactose, 3'-sialyllactose or 6'-sialyllactose) (2 µL, 1 M) was added and the absorbance was recorded every 60 seconds for a further 30 minutes.

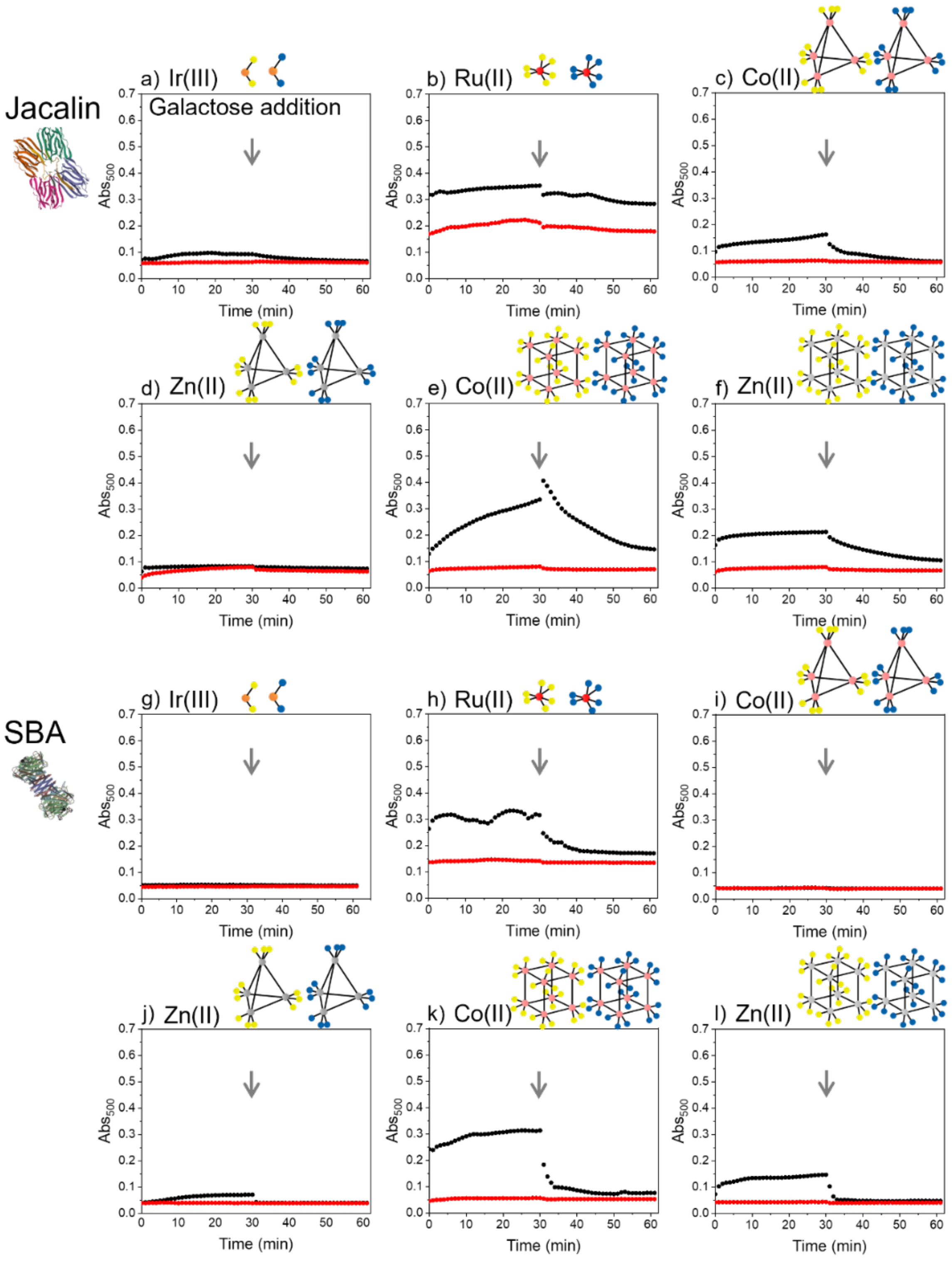

Figure S136. Turbidity monitored by A_{500} of Jacalin with: (a) **Ir^{Glu}** (red) and **Ir^{Gal}** (black), (b) **Ru^{Glu}** (red) and **Ru^{Gal}** (black), (c) **Co$_4^{Glu}$** (red) and **Co$_4^{Gal}$** (black), (d) **Zn$_4^{Glu}$** (red) and **Zn$_4^{Gal}$** (black), (e) **Co$_8^{Glu}$** (red) and **Co$_8^{Gal}$** (black), (f) **Zn$_8^{Glu}$** (red) and **Zn$_8^{Gal}$** (black); and SBA with with (g) **Ir^{Glu}** (red) and **Ir^{Gal}** (black), (h) **Ru^{Glu}** (red) and **Ru^{Gal}** (black), (i) **Co$_4^{Glu}$** (red) and **Co$_4^{Gal}$** (black), (j) **Zn$_4^{Glu}$** (red) and **Zn$_4^{Gal}$** (black), (k) **Co$_8^{Glu}$** (red) and **Co$_8^{Gal}$** (black), (l) **Zn$_8^{Glu}$** (red) and **Zn$_8^{Gal}$** (black).

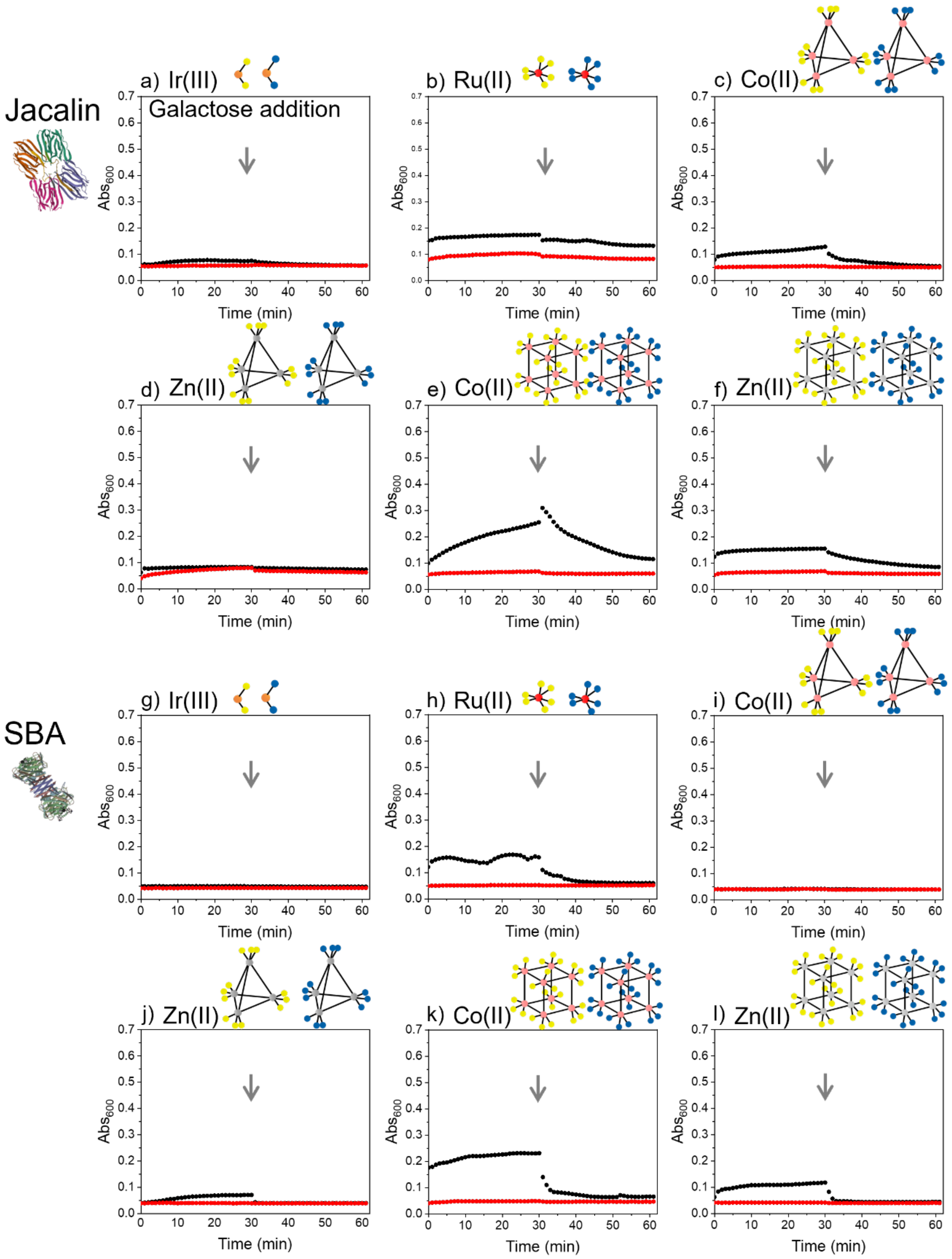

Figure S137. Turbidity monitored by A_{600} of Jacalin with (a) **Ir^{Glu}** (red) and **Ir^{Gal}** (black), (b) **Ru^{Glu}** (red) and **Ru^{Gal}** (black), (c) **Co$_4^{Glu}$** (red) and **Co$_4^{Gal}$** (black), (d) **Zn$_4^{Glu}$** (red) and **Zn$_4^{Gal}$** (black), (e) **Co$_8^{Glu}$** (red) and **Co$_8^{Gal}$** (black), (f) **Zn$_8^{Glu}$** (red) and **Zn$_8^{Gal}$** (black); and SBA with with (g) **Ir^{Glu}** (red) and **Ir^{Gal}** (black), (h) **Ru^{Glu}** (red) and **Ru^{Gal}** (black), (i) **Co$_4^{Glu}$** (red) and **Co$_4^{Gal}$** (black), (j) **Zn$_4^{Glu}$** (red) and **Zn$_4^{Gal}$** (black), (k) **Co$_8^{Glu}$** (red) and **Co$_8^{Gal}$** (black), (l) **Zn$_8^{Glu}$** (red) and **Zn$_8^{Gal}$** (black).

Competition experiments

Soybean agglutinin (SBA) was dissolved in HEPES buffer (10 mM HEPES, 0.15 M NaCl, 2 mM $CaCl_2$, 0.2 mM $MnCl_2$, pH 7.4)

Jacalin was dissolved in HEPES buffer (10 mM HEPES, 0.1 mM $CaCl_2$, pH 8.5)

In a 96-well plate half-area flat bottom, 20 µL serial dilution of D-galactose starting from 1 M and 20 µL of lectin (40 µM) were incubated at room temperature for 1 hour. An aqueous solution of glycan-appended metal complex (5 µL, 500 µM) was added to each well and the absorbance at 670, 700 and 750 nm was recorded every 60 seconds for 30 minutes.

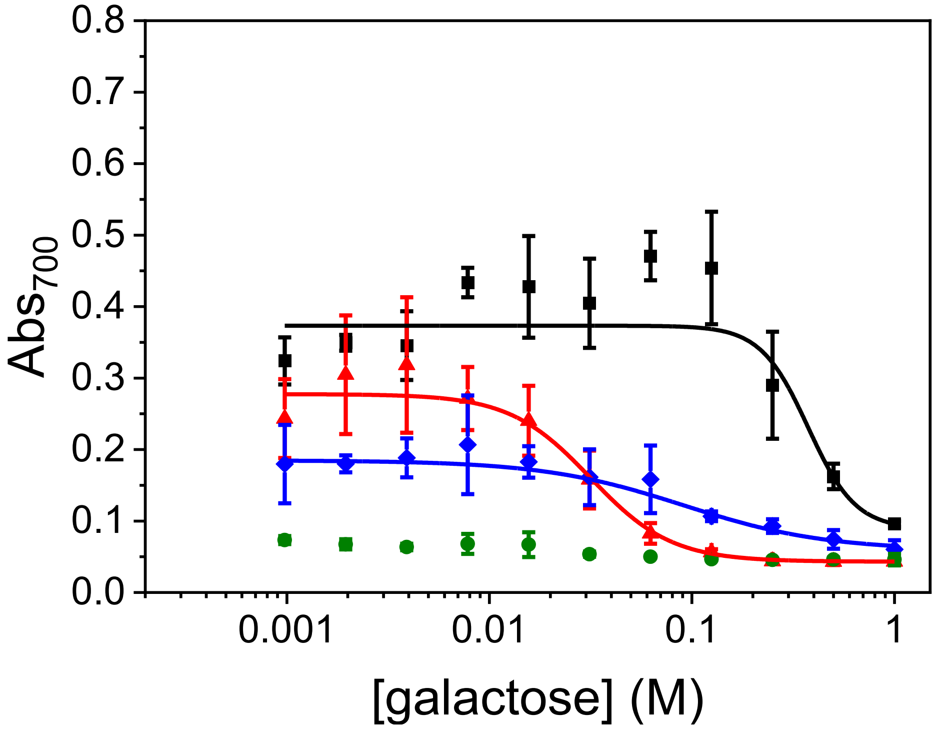

Figure S138. Competitive binding assay for Jacalin towards **Co_8^{Gal}** (black), **Co_4^{Gal}** (red), **Ru^{Gal}** (blue) and **Ir^{Gal}** (green). Monitored by A_{700}. Values shown are the average of 3 measurements.

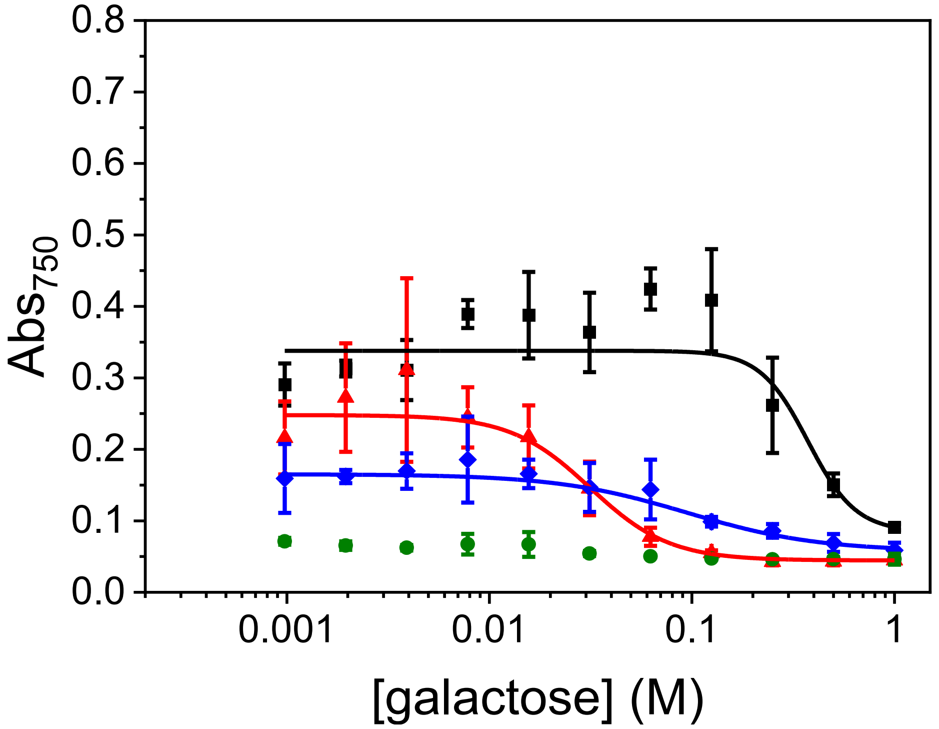

Figure S139. Competitive binding assay for Jacalin towards **Co8**^{Gal} (black), **Co4**^{Gal} (red), **Ru**^{Gal} (blue) and **Ir**^{Gal} (green). Monitored by A_{750}. Values shown are the average of 3 measurements.

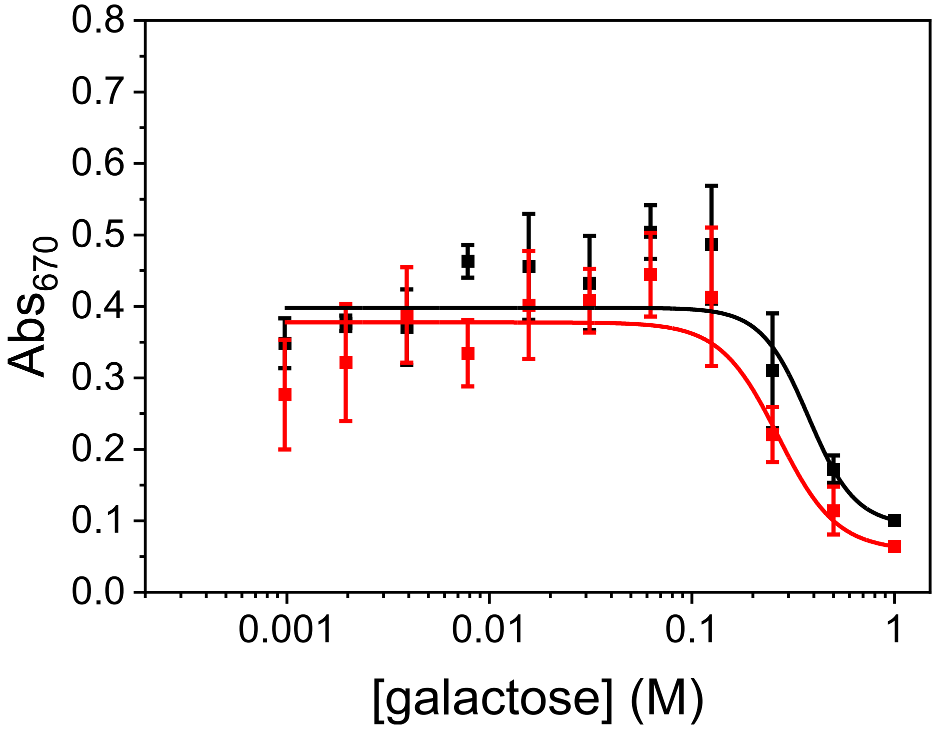

Figure S140. Competitive binding assay for Jacalin towards $\mathbf{Co_8}^{\mathbf{Gal}}$ (black) and $\mathbf{Zn_8}^{\mathbf{Gal}}$ (red). Fitting to Hill Equation provides IC50 values of 0.38 M for $\mathbf{Co_8}^{\mathbf{Gal}}$ and 0.27 M for $\mathbf{Zn_8}^{\mathbf{Gal}}$. Monitored by A_{670}. Values shown are the average of 3 measurements.

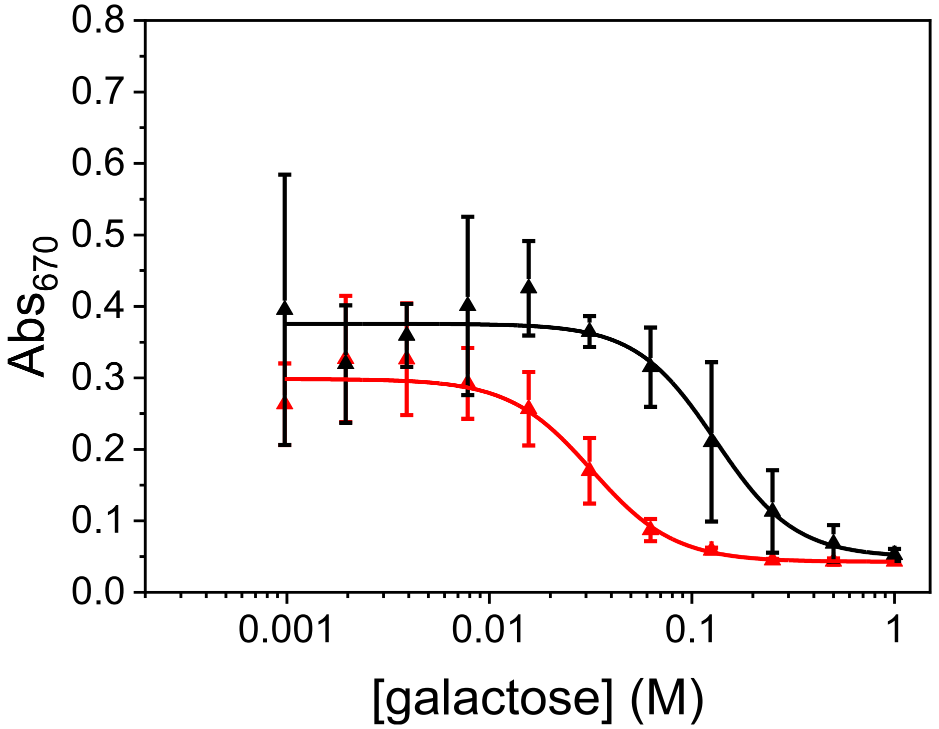

Figure S141. Competitive binding assay for Jacalin towards **Zn4**$^{\mathbf{Gal}}$ (black) and **Co4**$^{\mathbf{Gal}}$ (red). Fitting to Hill Equation provides IC50 values of 0.13 M for **Zn4**$^{\mathbf{Gal}}$ and 0.03 M for **Co4**$^{\mathbf{Gal}}$. Monitored by A_{670}. Values shown are the average of 3 measurements.

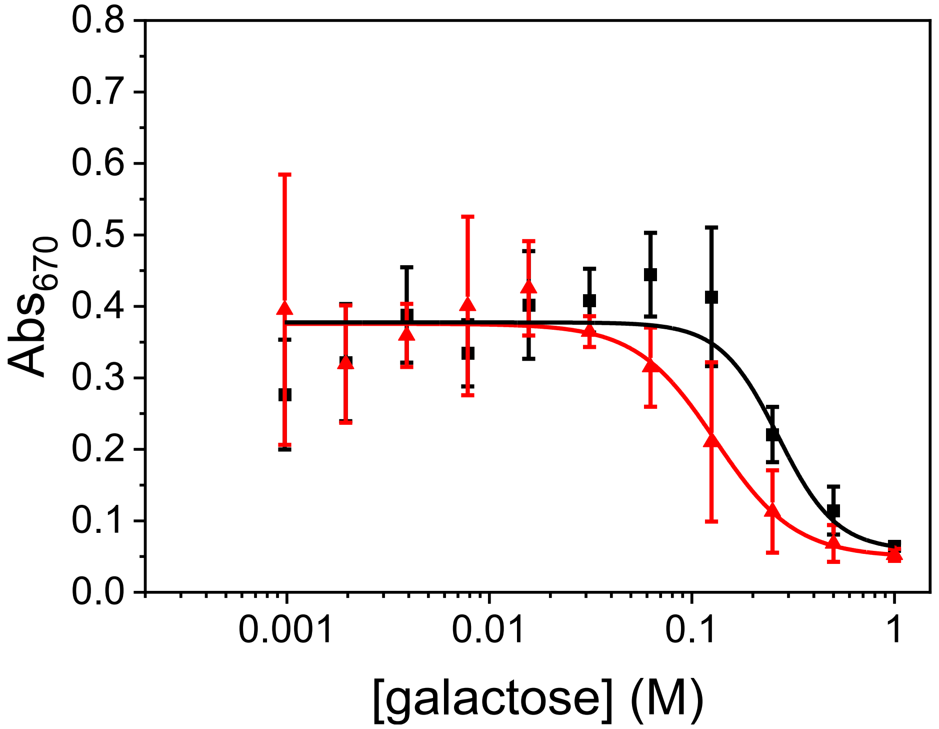

Figure S142. Competitive binding assay for Jacalin towards **Zn_8**^{Gal} (black) and **Zn_4**^{Gal} (red). Fitting to Hill Equation provides IC50 values of 0.27 M for **Zn_8**^{Gal} and 0.13 Mfor **Zn_4**^{Gal}. Monitored by A_{670}. Values shown are the average of 3 measurements.

Haemagglutination inhibition assay[13]

As an additional assay to demonstrate the availability of the sialyllactose motifs on the co-ordination metal complexes, an erythrocyte agglutination assay was undertaken. The lectin WGA can agglutinate erythrocytes giving a disperse solution, but in the absence of WGA the cells clump. If the complex inhibits the binding the cells can clump. To a U-bottom 96-well plate, a solution of WGA (25 µL, 0.05 mg/mL in PBS) and serial dilutions of **Co_8**^{3SL} and **Co_8**^{6SL} (starting from 4 mM in PBS) were incubated at room temperature for 1 hour. A 5% ovine erythrocyte solution in PBS (50 µL) was added to each well and the plate was incubated for few hours before being imaged. As expected, **Co_8**^{3SL} was a more potent inhibitor of WGA-induced agglutination than **Co_8**^{6SL}, with the formation of pellets of blood at the bottom of the well.

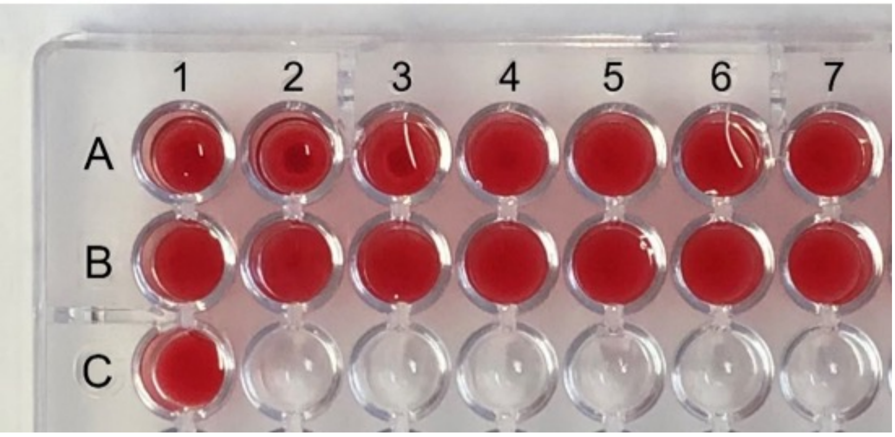

Figure S143. Haemagglutination by WGA, inhibition by **Co_8**^{3SL} (row A) and **Co_8**^{6SL} (row B). In column 1, complexes are 4mM, and serial (half) dilution across the plate (left to right). Well C1 serves as a control, where only WGA and erythrocytes were incubated.

Haemolysis[14]

Aqueous solutions of **Co_8**^{3SL} and **Co_8**^{6SL} (40 µL, serial dilutions starting from 0.2 mM in PBS) and sheep blood (40 µL, 5% solution in PBS) were mixed in Eppendorf tubes and incubated at room temperature. The erythrocytes solution was also incubated with PBS (40 µL) as a negative control and with MilliQ water (40 µL) as a positive control. After 1 hour, the solutions were centrifuged (6000 rpm, 3 minutes), 10 µL of supernatant were added to 90 µL of PBS in a 96-well plate, and the absorbance at 450 nm was monitored. Haemolysis registered for the negative control (PBS) was 4.5% and for the positive control (Milli-Q water) was 26%.

Table S1. Percentage of haemolysis caused by **Co_8**^{3SL} and **Co_8**^{6SL} at different concentrations of metal cage.

	**Haemolysis (%)**	
**Concentration (mM)**	**Co_8**^{3SL}	**Co_8**^{6SL}
**0.2**	5.1	4.6
**0.1**	5.1	4.9
**0.05**	5.6	5.1
**0.025**	5.5	4.8

References

[1] Huang, T.; Yu, Q.; Liu, S.; Zhang, K. Y.; Huang, W.; Zhao, Q. Rational Design of Phosphorescent Iridium(III) Complexes for Selective Glutathione Sensing and Amplified Photodynamic Therapy. *ChemBioChem* **2019**, *20* (4), 576–586. https://doi.org/10.1002/cbic.201800507.

[2] Hammarström, L.; Norrby, T.; Stenhagen, G.; Mårtensson, J.; Åkermark, B.; Almgren, M. Two-Dimensional Emission Quenching and Charge Separation Using a Ru(II)-Photosensitizer Assembled with Membrane-Bound Acceptors. *J. Phys. Chem. B* **1997**, *101* (38), 7494–7504. https://doi.org/10.1021/jp9710805.

[3] He, W. Y.; Fontmorin, J. M.; Hapiot, P.; Soutrel, I.; Floner, D.; Fourcade, F.; Amrane, A.; Geneste, F. A New Bipyridyl Cobalt Complex for Reductive Dechlorination of Pesticides. *Electrochim. Acta* **2016**, *207*, 313–320. https://doi.org/10.1016/j.electacta.2016.04.170.

[4] Arezzini, B.; Ferrali, M.; Ferrari, E.; Frassineti, C.; Lazzari, S.; Marverti, G.; Spagnolo, F.; Saladini, M. Synthesis, Chemical and Biological Studies on New Fe3+-Glycosilated β-Diketo Complexes for the Treatment of Iron Deficiency. *Eur. J. Med. Chem.* **2008**, *43* (11), 2549–2556. https://doi.org/10.1016/j.ejmech.2008.02.045.

[5] Andreeva, O. V.; Garifullin, B. F.; Sharipova, R. R.; Strobykina, I. Y.; Sapunova, A. S.; Voloshina, A. D.; Belenok, M. G.; Dobrynin, A. B.; Khabibulina, L. R.; Kataev, V. E. Glycosides and Glycoconjugates of the Diterpenoid Isosteviol with a 1,2,3-Triazolyl Moiety: Synthesis and Cytotoxicity Evaluation. *J. Nat. Prod.* **2020**, *83* (8), 2367–2380. https://doi.org/10.1021/acs.jnatprod.0c00134.

[6] Sylvie, B.; Huteau, V.; Zarantonelli, M.-L.; Pires, R.; Ughetto-Monfrin, J.; Taha, M.-K.; England, P.; Lafaye, P. Phosphorylcholine-Carbohydrate- Protein Conjugates Efficiently Induce Hapten-Specific Antibodies Which Recognize Both Streptococcus Pneumoniae and Neisseria Meningitidis: A Potential Multitarget Vaccine against Respiratory Infections. *J. Med. Chem.* **2004**, *47*, 3916–3919.

[7] Simerska, P.; Christie, M. P.; Goodwin, D.; Jen, F. E. C.; Jennings, M. P.; Toth, I. A1,4-Galactosyltransferase-Catalyzed Glycosylation of Sugar and Lipid Modified Leu-Enkephalins. *J. Mol. Catal. B Enzym.* **2013**, *97*, 196–202. https://doi.org/10.1016/j.molcatb.2013.08.018.

[8] Coppo, R. L.; Zanoni, K. P. S.; Murakami Iha, N. Y. Unraveling the Luminescence of New Heteroleptic Ir(III) Cyclometalated Series. *Polyhedron* **2019**, *163*, 161–170. https://doi.org/10.1016/j.poly.2019.01.058.

[9] Jackson, G.D.; Tipping, M.B.; Taylor, C.G.P.; Piper, J.R.; Pritchard, C.; Mozaceanu, C.; Ward, M.D. A family of externally-functionalised coordination cages. *Chemistry*, **2021**, *3*, 1203-1214. https://doi.org/10.3390/chemistry3040088.

[10] Sherman, A. A.; Yudina, O. N.; Komarova, B. S.; Tsvetkov, Y. E.; Iacobelli, S.; Nifantiev, N. E. Preparative Route to Per-O-acetylated N-Acetyl-and N-(tert-Butoxycarbonyl) neuraminyl-α-(2→ 3)-galactosyl Disaccharide Glycosyl Donors by Regioselective Acetolysis of Sialyl-α-(2→ 3′)-lactose. *Synthesis*, **2005**, 11, 1783-1788. http://doi.org/ 10.1055/s-2005-869957.

[11] a) Adesoye, O. G.; Mills, I. N.; Temelkoff, D. P.; Jackson, J. A.; Norris, P. Synthesis of a d-glucopyranosyl azide: Spectroscopic evidence for stereochemical inversion in the SN2 reaction. *J. Chem. Educ.*, **2012**, 89, 943-945; b) Whitehead, M.W.; Khanzhin, N.; Borsig, L.; Hennet, T. Custom glycosylation of cells and proteins using cyclic carbamate-derivatized oligosaccharides. *Cell Chemical Biology*, **2017**, 24, 1336–1346. https://doi.org/10.1016/j.chembiol.2017.08.012.

[12] Lorpitthaya, R.; Suryawanshi, S. B.; Wang, S.; Pasunooti, K. K.; Cai, S.; Ma, J.; Liu, X. W. Total Synthesis of Sialic Acid by a Sequential Rhodium-Catalyzed Aziridination and Barbier Allylation of D-Glycal. *Angew. Chem. Int. Ed.* **2011**, 50, 12054-12057. https://doi.org/10.1002/ange.201104516.

[13] Mammen M.; Dahmann G.; Whitesides GM. Effective inhibitors of hemagglutination by influenza virus synthesized from polymers having active ester groups. Insight into mechanism of inhibition. *J. Med. Chem.* **1995**, 38 4179-90. https://doi.org/10.1021/jm00021a007

[14] Richards, S.-J.; Isufi, K.; Wilkins, L. E.; Lipecki, J.; Fullam, E.; Gibson, M. I. Multivalent Antimicrobial Polymer Nanoparticles Target Mycobacteria and Gram-Negative Bacteria by Distinct Mechanisms. *Biomacromolecules* **2018**, *19* (1), 256–264. https://doi.org/10.1021/acs.biomac.7b01561.
